# A revision of *Dissochaeta* (Melastomataceae, Dissochaeteae)

**DOI:** 10.3897/phytokeys.107.26548

**Published:** 2018-08-09

**Authors:** Abdulrokhman Kartonegoro, Jan Frits Veldkamp, Peter Hovenkamp, Peter van Welzen

**Affiliations:** 1 Naturalis Biodiversity Center, P.O. Box 9517, 2300 RA Leiden, The Netherlands Naturalis Biodiversity Center Leiden Netherlands; 2 Research Center for Biology, Indonesian Institute of Sciences (LIPI), Jl. Jakarta-Bogor KM.46, Cibinong 16911, Indonesia Research Center for Biology, Indonesian Institute of Sciences Cibinong Indonesia; 3 Institute of Biology (IBL), Leiden University, P.O. Box 9505, 2300 RA Leiden, The Netherlands Leiden University Leiden Netherlands

**Keywords:** *
Dissochaeta
*, *
Macrolenes
*, Melastomataceae, revision, South–East Asia, taxonomy

## Abstract

*Dissochaeta* is a plant genus of woody climbers, classified in the tribe Dissochaeteae (Melastomataceae). The taxonomic history of the genus is complicated and includes some allied genera like *Dalenia*, *Diplectria*, *Macrolenes* and *Omphalopus*. Most of them are already regarded as synonyms of *Dissochaeta* except for *Macrolenes* which is considered a separate genus here as well. *Dissochaeta* is characterised by its scrambling habit, interpetiolar outgrowths, 4-merous flowers, dimorphic stamens and berry-like fruits. A taxonomic revision of *Dissochaeta* is presented, which includes references, a complete list of synonyms, detailed morphological descriptions of the species and an identification key, as well as information on the distribution, habitat and ecology, vernacular names, notes and lists of examined specimens. Fifty four species and two varieties of *Dissochaeta* are recognised. We designate several lectotypes, propose eleven new combinations and we describe one new species and one new variety.

## Introduction

*Dissochaeta* Blume is a genus of woody climbers found in South–East Asia. The plants typically grow in open or secondary habitats, where they climb in small trees or shrubs. The genus is characterised by its scrambling growth habit, opposite phyllotaxy with interpetiolar outgrowths, terminal or rarely axillary inflorescences, 4-merous flowers, 2-whorls of dimorphic stamens and berry-like fruits. Some species are restricted both geographically and elevationally, while others are widespread. The genus *Macrolenes* Naudin closely resembles *Dissochaeta* and also consists of woody climbers with a scrambling habit, but differs in some vegetative and flowering aspects. Together with *Macrolenes*, *Dissochaeta* is included within Tribe Dissochaeteae (Naudin) Triana (Triana 1872, Cogniaux 1891, Bakhuizen van den Brink *f.*1943, Maxwell 1984, Clausing and Renner 2001). On the other hand, the genus is also considered as part of tribe Miconieae (Blume 1831a, 1831b, Don 1832, Endlicher 1840, Naudin 1851, Miquel 1855, Renner 1993). Taxonomic revisions for parts of the genus and its allies can be found in Bakhuizen van den Brink *f.* (1943), Veldkamp et al. (1979), Nayar (1980) and Renner et al. (2001).

## Taxonomic history

*Dissochaeta* was first proposed by Blume (1831a) and consisted of 15 species with eight of them split off from *Melastoma* L. (in its wide sense: Jack 1823, Blume 1826, De Candolle 1828). The word *Dissochaeta* is derived from the Greek words “dissos”, meaning double and “chaitè”, meaning hair or bristle and alludes to the two filiform appendages at the base of the anthers (Backer 1936, Maxwell 1980a, Kartonegoro and Veldkamp 2010). This feature is present in most of the species, but absent in a few. Blume (1831a) proposed two sections in the genus, section Dissochaeta and section Diplectria Blume, which differ in the shape of the calyx tube, the appendages at the base of the anthers and the indumentum of the ovary apex. Section Dissochaeta has a cyathiform calyx tube, 4-dentate calyx lobes and an apically pubescent ovary, while section Diplectria has a cylindric calyx tube, truncate lobes and an ovary with a glabrous apex (Blume 1831a, 1831b). Section Dissochaeta was subdivided by Blume (1831a) into three informal groups, a) Tetrandrae, flowers with 4 stamens, without any staminodes; b) Octandrae flowers with 4 stamens, alternating with 4 staminodes; and c) Octandrae flowers with 8 fertile stamens (Maxwell 1980a, Kartonegoro and Veldkamp 2010).

Blume (1831a) also described the new genus *Aplectrum* Blume, comprising three species, which have an ovate-globose calyx tube and four stamens alternating with four staminodes. The anthers of *Aplectrum* were said to be inappendiculate, unlike the appendiculate anthers of *Dissochaeta* (Blume 1831a). Blume did not indicate the similarity/difference between *Aplectrum* and Dissochaetasect.Diplectria, which also has four staminodes alternating with four stamens. He also did not mention the position of the fertile and sterile stamens in relation to the position of the petals, a character later used to separate genera (Maxwell 1980a). Later, Reichenbach (1841) raised section Diplectria to genus level as *Diplectria*. Simultaneously with the establishment of *Dissochaeta* and *Aplectrum*, Blume (1831a) established *Marumia* Blume (=*Macrolenes*), also a woody climber, but different in having axillary inflorescences, persistent and long calyx lobes, eight fertile stamens and several filiform appendages at the base of the anthers.

Korthals (1844) accepted Blume’s *Dissochaeta* and *Aplectrum* as distinct groups of woody climbing genera in Melastomataceae in his Netherland Indies (Indonesia) Melastomataceae account. He proposed a new woody climber genus, *Dalenia* Korth., which has similarities with *Dissochaeta*, but instead has a deciduous calyptra which encloses the petals before anthesis. Naudin (1851) included *Diplectria* in *Dissochaeta* and made a new division of the genus into two groups without any nomenclatural status, *Inermes* Naudin and *Bisetosae* Naudin, which differ from each other in lacking or having bristle appendages at the base of the anthers, respectively. The *Inermes* group has similarities with Blume’s *Diplectria* and *Bisetosae* with Blume’s *Dissochaeta*. Furthermore, Naudin (1851) maintained the genera *Aplectrum* and *Dalenia* and he also proposed a new genus, *Omphalopus* Naudin, with 3 species defined by having filaments attaching to the anthers in the middle (medifixed) and a tessellate surface of the locules (Naudin 1851).

The name *Aplectrum* is a later homonym of *Aplectrum* (Nutt.) Torr., already proposed by Torrey (1826) for a subgenus of *Corallorrhiza* (Orchidaceae) by Nuttal (1818). Therefore, Gray (1854) introduced the new name *Anplectrum* A.Gray as a valid genus name for Blume’s *Aplectrum*, which was followed by Triana (1872) in his World Melastomataceae account by uniting all species of *Diplectria* and *Aplectrum* within *Anplectrum*.

Baillon (1877) divided *Dissochaeta* into nine sections: *Anoplodissochaeta* Baill., *Anplectrum* (A.Gray) Baill., *Creochiton* (Blume) Baill., *Dalenia* (Korth.) Baill., *Dicellandra* (Hook.*f.*) Baill., *Eudissochaeta* Blume ex Endl. (invalid name for section Dissochaeta), *Omphalopus* (Naudin) Baill., *Oxyotandra* Baill. and *Sakersia* (Hook.*f.*) Baill. His broad circumscription of the genus also included the non-woody climbing genera *Creochiton* Blume (woody epiphyte), *Dicellandra* Hook.*f.* (herb to woody) and *Sakersia* Hook.*f.* (woody = *Dichaetanthera* Endl.) with *Dissochaeta*. The distribution of the genus also became wider, because *Dicellandra* and *Dichaetanthera* are African genera.

Cogniaux (1890, 1891), in his monograph of the family, accepted Triana’s (1872) concept and rejected Baillon’s generic classification (1877). He reinstated several genera and divided Dissochaeta into three sections, sect. Diplostemones Cogn. (invalid name, should have been section Dissochaeta; Kartonegoro and Veldkamp 2010), with a truncate or obscurely lobed calyx and eight stamens with elongate appendages; sect. Isostemones Cogn., with a similar calyx but with four stamens with elongate appendages; and sect. Dissochaetopsis Cogn., with long, linear to lanceolate, caducous calyx lobes and four straight stamens with short appendages. Cogniaux’s classification of *Dissochaeta* and allied genera were adopted by Krasser (1893) except that he synonymised *Anplectrum* with the older genus *Diplectria*. The number of infrageneric taxa increased when Merrill (1917) proposed the new species *Dissochaetaglabra* Merr. and placed it in a new section Disparistemones Merr.

Bakhuizen van den Brink *f.* (1943), in his comprehensive work on the Melastomataceae of the Malay Archipelago (Malesian Region), did not adopt an infrageneric classification for *Dissochaeta*, quite unlike previous authors. Thus, he described some new species in *Dissochaeta* and established two new woody-climbing genera, *Backeria* Bakh.*f.* and *Neodissochaeta* Bakh.*f.*, based on the small size of the calyx tube and the presence of narrow extra-ovarian chambers, respectively. Like Cogniaux (1890, 1891), he also maintained the genera *Dalenia* and *Omphalopus* as distinct genera. He discussed the possible illegitimate character of the name *Anplectrum*, which he considered to be a superfluous orthographic variant of *Aplectrum* (both bad Greek) and he preferred to regard *Diplectria* and *Backeria* as accepted names instead (Bakhuizen van den Brink *f.* 1943, 1964, Veldkamp et al. 1979).

Nayar (1966, 1969b) considered *Neodissochaeta* as a distinct genus and added some new species to it. The genus *Backeria* was also maintained by Raizada (1968), but he synonymised all species of *Diplectria* with it. However, since *Diplectria* is an older name than *Backeria*, *Diplectria* is the correct generic name (Veldkamp et al. 1979) in this circumscription.

Maxwell (1980a, 1980b) divided *Dissochaeta* into only three sections: sect. Dissochaeta, sect. Anoplodissochaeta and sect. Omphalopus. This separation is mostly based on floral characters, especially the stamens. Section Dissochaeta has well-developed calyx lobes (>2 mm long) and curved stamens, while sections *Anoplodissochaeta* and *Omphalopus* have undeveloped calyx lobes (<2 mm long) and straight stamens. Section Omphalopus differs from sect. Anoplodissochaeta by having tessellate-reticulate locules (vs. smooth ones) and medifixed anthers (vs. basifixed). In agreement with Bakhuizen van den Brink *f.* (1943) and Veldkamp et al. (1979), he also maintained *Diplectria* as a distinct genus allied to *Dissochaeta* with *Backeria* synonymised under it and he included *Dalenia*, *Neodissochaeta* and *Omphalopus* in *Dissochaeta*.

Results of molecular phylogenetic studies by Clausing and Renner (2001) showed that a woody climbing or scrambling growth habit evolved only once in the Asian Melastomataceae. Based on that result, Renner et al. (2001) recognised only the single genus *Dissochaeta*, with two other genera, *Diplectria* and *Macrolenes*, as synonyms. Renner et al. (2001) ignored the differences in floral characters. *Macrolenes*, sister to *Dissochaeta* (Clausing and Renner 2001), differs from *Dissochaeta* in a unique combination of vegetative and floral characters (presence of a pair of hair cushions at the base of the lower leaf surface, axillary inflorescences, long and persistent calyx lobes and the anthers with several basal filiform appendages) and is, therefore, considered to be a distinct genus, separate from *Dissochaeta* (Bakhuizen van den Brink *f.* 1943, Nayar 1980, Maxwell 1984).

### Circumscription of *Dissochaeta* proposed in this study

*Diplectria* is here considered to be a synonym of *Dissochaeta* since both genera have correlating floral characters with intermediates between the extreme forms. This concept of *Dissochaeta*, including *Diplectria*, was already pointed out by Backer (Bakhuizen van den Brink *f.* 1943), following Naudin’s concept (Naudin 1851). *Dissochaeta* and *Diplectria* show a strong morphological similarity (Bakhuizen van den Brink *f.* 1943, Veldkamp et al. 1979, Maxwell 1984) in their the scrambling habit and terminal inflorescences with 2‒5 ramifications, but were distinguished based on floral characters like the position of the stamens and staminodes on the hypanthium (Veldkamp et al. 1979, Maxwell 1980b). According to Maxwell (1984), *Diplectria* differs from *Dissochaeta* in having four fertile stamens opposite the petals (oppositipetalous) and four staminodes alternate to the petals (alternipetalous). In contrast, in *Dissochaeta* the alternipetalous stamens are always fertile, while the oppositipetalous stamens are either fertile, staminodes or absent. Based on these differences, these two genera were even classified in two different subtribes, *Diplectrinae* J.F.Maxwell and *Dissochaetinae* Naudin (Maxwell 1980b, 1984). However, there are strong similarities between *Dissochaeta* and *Diplectria* in the structure of the stamens: their position in bud, connective appendages and the direction and shape of the alternipetalous stamens. The oppositipetalous stamens of *Diplectria* also similar to those of *Dissochaeta*. The shape and orientation of the oppositipetalous stamens in *Dissochaetabeccariana* Cogn., *Dissochaetaglandulosa* Merr., *Dissochaetalaevis* Ohwi ex J.F.Maxwell and *Dissochaetasarawakensis* (M.P.Nayar) J.F.Maxwell are similar to those of *Diplectria*. These four *Dissochaeta* species also have a pair of glandular patches abaxially on the base of the leaf blades, which is also found in several species of *Diplectria* and, therefore, they are considered as intermediate between the two genera and which are here regarded congeneric because of the resulting continuous morphological variation.

*Dalenia* was distinguished from *Dissochaeta* based on the presence of a calyptra enclosing the petals in bud (Korthals 1844, Naudin 1851, Miquel 1855, Triana 1872, Cogniaux 1890, 1891, Krasser 1893, Bakhuizen van den Brink *f.* 1943, Nayar 1966). The calyptra is in fact the hypanthium/calyx and it falls off when flowers are mature. Despite this calptriform hypanthium, the habit, position of the inflorescences, stamen characters and the baccate fruits are highly similar to those of *Dissochaeta* and *Diplectria* within the tribe *Dissochaeteae* (Nayar 1966). As the inflorescence position and the stamen characters are considered to be more important characters for the recognition of genera, the presence of the calyptra is regarded as a variation within the genus and *Dalenia* is considered as congeneric with *Dissochaeta* in this revision following Maxwell (1980b, 1984) and Renner et al. (2001).

*Omphalopus* was also distinguished from *Dissochaeta* by its tessellate reticulate anthers with medifixed filament attachments (Naudin 1851, Miquel 1855, Triana 1872, Cogniaux 1890, 1891, Krasser 1893, Bakhuizen van den Brink *f.* 1943). This unusual insertion seems to be insufficient for separating the genus and since the habit, leaves arrangement, inflorecences, calyx tube and fruits resemble those of *Dissochaeta*, it may by considered as a synonym (Maxwell 1980b, Renner et al. 2001, Kartonegoro and Veldkamp 2010).

*Macrolenes* was also known to have similar habit and ecological aspects with *Dissochaeta*. The genus also grows as woody climbers with a scrambling habit, but differs in some vegetative and flowering aspects with *Dissochaeta*. *Macrolenes* can be distinguished from *Dissochaeta* by a combination of some characters, e.g. axillary inflorescences (vs. mainly terminal in *Dissochaeta*), a pair hair cushion domatia on the base of abaxial leaves (vs. cushion domatia absent), longer and distinct calyx lobes (vs. mainly shorter and often indistinct calyx lobes) and several fimbriate, filiform appendages on the alternipetalous anthers (vs. only a pair of filiform, non-fimbriate appendages on the alternipetalous anthers). Some species of *Dissochaeta* have long calyx lobes, similar to those of *Macrolenes*, but they are usually erect, not reflexed and mostly fall off when fruiting. Based on those constant differences in morphological characters between two genera, here we agree to keep *Macrolenes* as a separate genus from *Dissochaeta*.

Fifty four species and two varieties are recognised in this revision. Species delimitations are based on clear morphological discontinuities in more than a single character. Specific characters used for recognition are shown in the descriptions, notes and the key. We have not recognised subspecies, because no allopatric forms were found, but instead either described the infraspecific variation without any taxonomic categories or we recognised varieties when a character shows a discontinuity. An infrageneric classification is not (yet) included in this revision, a future better resolved phylogeny should form the basis for that.

## Materials and methods

This revision is based on the analysis of gross morphological characters of *Dissochaeta* for which more than 2000 herbarium specimens were studied and for which the following herbaria are thanked for loans/facilities: ANDA, BM, BO, E, K, L, SING and U (abbreviations follow Thiers 2018). Additionally, the *Dissochaeta* collections in the databases and specimen images from A (http://kiki.huh.harvard.edu/databases/specimen_index.html), AAU, BISH (http://nsdb.bishopmuseum.org), BK, BR, BRI, C, CAS, CM, F, FI, G, GH, HBG (http://www.herbariumhamburgense.de/Data_Spermatophyta/index.php), KEP, MCU, MICH, MO, MPU, NY (http://sweetgum.nybg.org/science/vh), P, PH, PNH, S, TCD, US (http://collections.nmnh.si.edu/search/botany) and JSTOR Global Plants (http://plants.jstor.org) were also used. Investigation of morphological characters including indumentum, flowers and fruits was performed with binocular stereomicroscopes. The types of almost all names were examined either as actual specimens or as images. Morphological descriptions and measurements were made from dried specimens and fresh material with terminology following Bakhuizen van den Brink *f.* (1943), Nayar (1966, 1980), Veldkamp et al. (1979), Maxwell (1980a, 1984) and Renner et al. (2001). Distribution maps were prepared using DIVA-GIS (http://www.diva-gis.org/). A list of selected examined specimens was prepared and listed under each species per country and, secondarily, per province or island. All examined specimens are also alphabetically listed together in a separate index.

## Results

### Morphology

***Habit***

**Figure 1.**
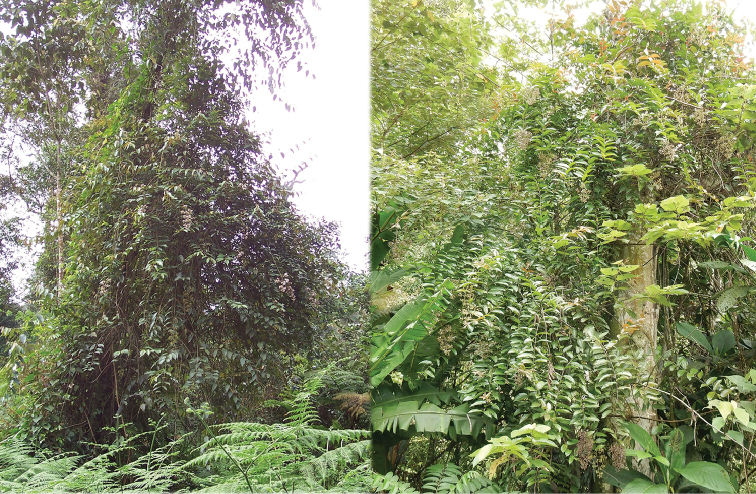
Habit of *Dissochaeta*. Photographs by A. Kartonegoro (left) and D. Penneys (right).

All species of *Dissochaeta* are essentially woody climbers with a scrambling growth habit. This scrambling growth is also known for *Macrolenes* and some species of *Creochiton* (Kartonegoro and Veldkamp 2013), but otherwise unknown within Old World Melastomataceae (Clausing and Renner 2001). Most of the known species are reported to climb on to the branches of mainly small trees or shrubs, though sometimes they reach up to 30 m high (Fig. 1). The species do not scramble into big canopy trees, because they need open space to germinate and grow rather than dense shade. Due to their scrambling growth, individuals usually have thin branches with non-self supporting, long internodes and pendent flowering and fruiting branches (Maxwell 1984, Clausing and Renner 2001, Kartonegoro and Veldkamp 2010). In some species, adventitious roots are also common, which lignify and become hook-shaped structures after desiccation (Clausing and Renner 2001).

***Branchlets***

The branchlets are usually terete and rarely angular, though, in some taxa. angular branchlets become terete when older. The indumentum of the branchlets is variable, ranging from subglabrous, glabrescent or covered with sparse or dense stellate hairs with a punctate, furfuraceous, tomentose, or floccose appearance. In addition, some species also have short or long, dense, simple, glandular or eglandular bristle hairs. Mature branches are usually glabrescent.

***Nodes***

**Figure 2.**
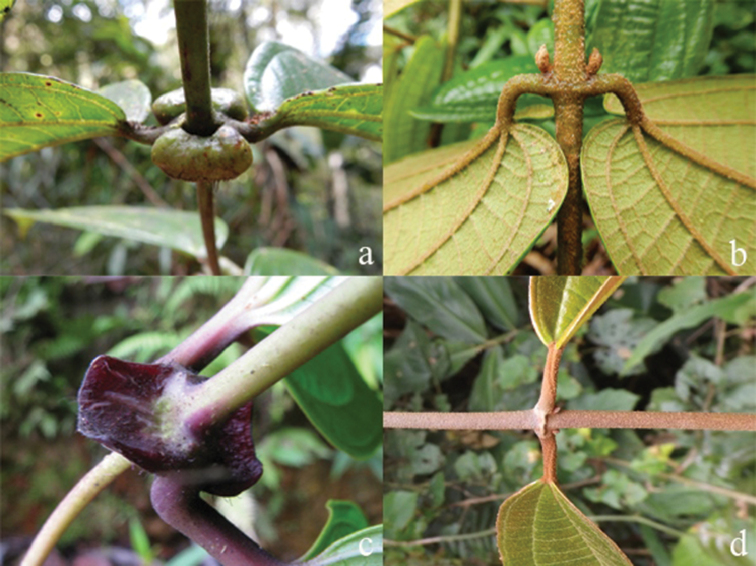
Nodes of *Dissochaeta***a***D.glabra***b***D.macrosepala***c***D.pulchra***d***D.viminalis*. Photographs by D. Penneys (**a, b**), J. Henrot (**c**), A. Kartonegoro (**d**).

Nodes of all species bear some kind of large and swollen interpetiolar outgrowths (stipules are unknown in the family), which vary from just lines and ridges to crest-like and often annular outgrowths (Fig. 2). In some species, such a *D.glabra* Merr., *D.glandiformis* J.F.Maxwell, *D.pulchra* (Korth.) J.F.Maxwell, *D.sarawakensis* and *D.stipularis* (Blume) Clausing, the interpetiolar outgrowth is conspicuous and wide, which may help climbing and stabilisation in the same way thorns or hooks do in other scramblers (Clausing and Renner 2001). The indumentum of the nodes is similar to that of the branchlets, but denser.

***Leaves***

Like in most Melastomataceae, the phyllotaxis in *Dissochaeta* is opposite in one row (distichous, never decussate) with equal-sized (isophyllous) leaves. The shape is quite variable from ovate, elliptic to oblong or combinations of these within species. The apex usually is acuminate with a varying tip length. The margin is generally entire and becomes wavy when dry except for *D.pulchra* and *D.rectandra* Karton., which have a slightly serrulate margin. The leaf base varies between rounded, subcordate to shallowly cordate with distinct sinuses. The venation of the leaves is acrodromal with a midrib at the base and one or two pairs of major secondary (lateral) veins. Another pair of lateral veins also arises from the base and runs along or merges with the leaf margin and forms an intramarginal vein. In general, there are numerous secondary veins and a reticulate pattern of finer, higher order veins (Maxwell 1984). The main veins are usually sunken adaxial and raised on abaxial. Most species have a dark glossy, glabrous adaxial leaf surface except in some species, e.g. *D.hirsutoidea* Furtado, *D.porphyrocarpa* Ridl. and *D.rostrata* Korth., which are hispid and covered by sparse or dense bristle hairs. On the abaxial surface, the indumentum varies amongst the species from glabrous to stellate puberulous to furfuraceous, tomentose, floccose or setose with glandular or eglandular bristle hairs. Unfortunately, the leaves are usually not sufficient for definitive determinations and many species of *Dissochaeta* require flowers or fruits for identification because many vegetative characters are generally shared by two or more taxa.

A pair of peculiar thin walled corky cushions at the base of the leaf blades on the abaxial surface, called “glandular patches”, are found in species like *D.beccariana*, *D.glabra*, *D.glandulosa* and *D.laevis*. This feature resembles a pair of hair cushions at the base of the leaf blades on the lower surface in *Macrolenes*. Their function, if any and homology with domatia, are unknown (Maxwell 1984).

***Petiole***

The petioles are well developed in all species and are terete with a dorsal groove, which may give the impression of a flattened petiole. The indumentum is similar to that found on the branchlets except for being setose in *D.sarawakensis* and *D.stipularis*.

***Inflorescences***

The inflorescences are cymose and, in most species, they are terminal, multi-flowered raceme-like thyrses or panicles. Axillary inflorescences with few flowers are found in a few species: *D.acmura* Stapf & M.L.Green, *D.axillaris* Cogn. and *D.laevis*. *Dissochaetaannulata* Hook.*f.* ex Triana, *D.conica* (Bakh.*f.*) Clausing, *D.atrobrunnea* G.Kadereit and *D.viminalis* (Jack) Clausing sometimes have terminal and axillary inflorescences. Terminal inflorescences are usually panicles with reduced leaves on the proximal nodes of the axis (Maxwell 1984). The length of the terminal inflorescences varies from 10-16 cm (*D.biligulata* Korth.) to up to 90 cm (*D.glabra*). The rachis is usually angular or 4-angled instead of being terete and the nodes and indumentum are similar to those on the branchlets except in *D.sarawakensis* and *D.stipularis*. Ramifications of the panicles can be up to 5 orders and the branching within it is decussate with 3-flowered cymules terminating each terminal ramification (Maxwell 1984). The central flower of the terminal cymules usually has a longer pedicel than the two lateral ones, which are, in fact, the last order of ramification. In most species, the central flower of the cymules will mature and open first, followed by the two lateral ones, which bloom simultaneously. Likewise, for most members of the family, the flowers are actinomorph and epigynous. Here, they are also 4-merous with similar size of the petals.

***Bracts and bracteoles***

Bracts and bracteoles are present in all species even though some of them fall off before anthesis (Maxwell 1984). In this revision, bracts are pairs of leaf-like organs opposite at each node at every ramification level of the inflorescences, whereby the bracts on the primary axes nodes are the largest and the size gradually diminishes with each higher node. Bracteoles are recognised as an appendage or leaf-like structure subtending the base of each pedicel and are found only basal to the lateral flowers of the terminal 3-flowered cymules. The shape of bracts and bracteoles is also of taxonomic value, because it varies between minute, subulate, linear, lanceolate, ovate and suborbicular. Distinct bracteoles are found in *D.beccariana*, *D.bracteata* (Jack) Blume and *D.glandulosa*, which sometimes enclose flowers buds.

***Hypanthium and calyx***

Like in most Melastomataceae, the receptacle forms a tube, the hypanthium, with calyx lobes at the apex, alternating with whorls of petals and stamens (Hansen 1984). The shape of the hypanthium varies from campanulate, urceolate, tubular to cyathiform or funnelform and can be terete or angular. The size of the hypanthium also varies from small (2–4 mm long) in *D.biligulata*, D.glabravar.glabra and *D.gracilis* (Jack) Blume to large (8–10 mm long) in *D.axillaris*. The indumentum of the hypanthium ranges from glabrous to stellate-furfuraceous to tomentose to floccose with or without scattered to dense bristle hairs. This indumentum is important to identify certain species (Maxwell 1984). The presence of eight vertical ridges on the hypanthium is typical for species like *D.leprosa* (Blume) Blume, *D.pallida* (Jack) Blume and *D.spectabilis* J.F.Maxwell.

There are four calyx lobes, but these are not always visible as the calyx of most species may be truncate, undulate or have four small points. Calyx lobes can be rounded or triangular as in *D.annulata*, *D.atrobrunnea* and *D.leprosa* or can be linear to long, lanceolate as in *D.johorensis* Furtado, *D.macrosepala* Stapf and *D.porphyrocarpa*. The lobes are important for identification. The indumentum of the calyx lobes is similar to that of the hypanthium.

***Petals***

As the flowers are 4-merous, four free petals are commonly present. The petals are usually contorted in bud and overlap. The petal bud is always conical with an acute or acuminate tip, but some are rounded in *D.fallax* (Jack) Blume. The petals are thin, conspicuous, symmetric and colourful. Even though the colour of the petals generally has very little taxonomic value, in some cases the constant colour of the petals is useful to distinguish the species. The most frequent shapes are ovate, obovate and suborbicular with rounded or obtuse to acute tips and a clawed base. In a few species, the margin and tip of the petals are somewhat bristly, e.g. *D.hirsutoidea*, *D.johorensis*, *D.malayana* Furtado and *D.porphyrocarpa*. The petals are reflexed or erect.

***Stamens***

The stamens provide the best taxonomic characters for identification (Kartonegoro and Veldkamp 2010). In most species, 8 heterantherous stamens are usually present in two, dimorphic staminal whorls, an outer, alternipetalous and an inner, oppositipetalous one (Maxwell 1984, Kartonegoro and Veldkamp 2010). The alternipetalous stamens are known as the pollinating stamens and the oppositipetalous (alternisepalous) ones are the feeding stamens (Kadereit 2006, Kartonegoro and Veldkamp 2010). Most species have 8 fully developed and complete fertile stamens; in some species only 4 fertile stamens developed with the other 4 stamens being staminodial or absent.

**Figure 3.**
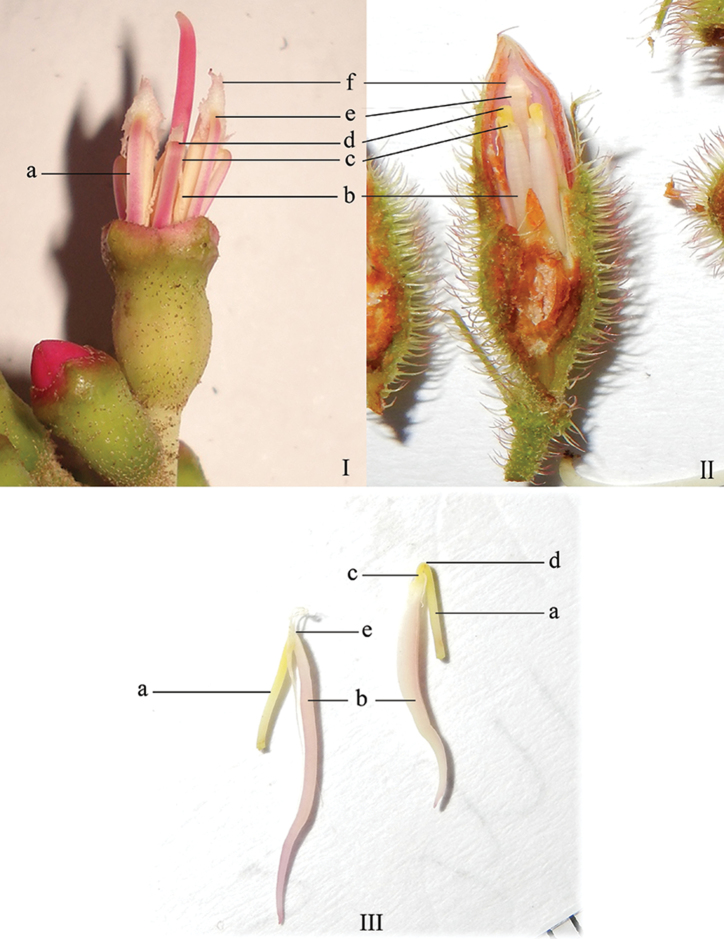
Stamens of *Dissochaeta* in bud. **I** facing outside **II** facing inside **III** separated stamens, alternipetalous (left); oppositipetalous (right). **a** filaments **b** thecae **c** point of attachment oppositipetalous stamens with filaments **d** stipopodium **e** point of attachment alternipetalous stamens with filaments **f** basal crest. Photographs by A. Kartonegoro (**I**) and D. Penneys (**II, III**).

The filaments are well-developed, flattened, glabrous and uniform in shape. Their length and orientation vary with the stage of maturity of the stamens. The filaments originate at the same level below the inner margin of the hypanthium. In both anther types (alternipetalous and oppositipetalous) before anthesis, the filaments are abaxial (facing outside) and the anthers adaxial (facing towards the inside) (Fig. 3I, II). The filaments alternating with the petals are straight and the point of attachment with the anthers distinct, while those opposite the petals are sharply bent and incurved before reaching the rather indistinct point of attachment with the anthers. Distally there is a sharp bend shortly below the attachment to the anther, the stipopodium (Fig. 3d) (Veldkamp et al. 1979). When dissecting the filament, it disarticulates and breaks here easily, although the “natural” point of breakage is apparently between the stipopodium and the connectival area of the basal crest and lateral appendages (Veldkamp et al. 1979). The attachment of the filament to the anther is usually near the base (basifixed) except in *D.fallax* where the filament is inserted in the middle part of the anther (medifixed). In bud, the stamens are inserted at the inner margin of the hypanthium, either in the extra-ovarial chambers or not.

**Figure 4.**
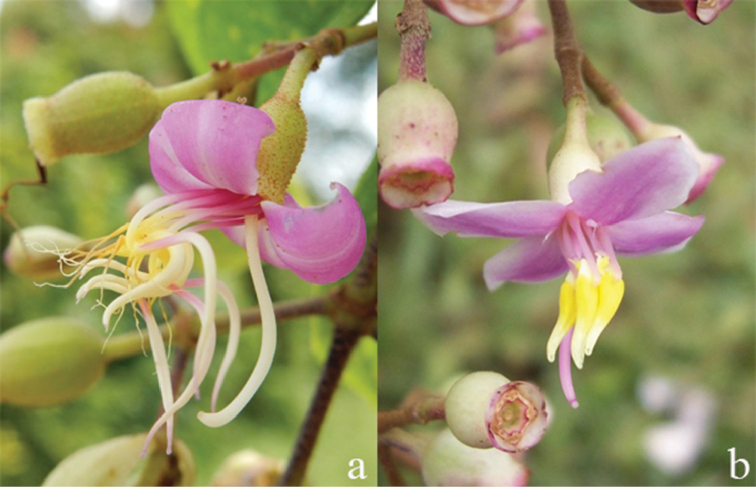
Mature flowers with mature stamens. **a** curved anthers (*D.bracteata*) **b** straight anthers (*D.inappendiculata*). Photographs by D. Penneys (**a**) and A. Kartonegoro (**b**).

The anthers are elongate, subulate and glabrous and open distally with a single pore. In mature flowers, they reverse their orientation by bending upwards and become less apical to the filaments. Filaments become longer and curve sideways or straight upwards. The stipopodium of mature oppositipetalous stamens becomes flexed to sinuate and leaves a scar-line, thus the filament and anther are not in parallel alignment (Maxwell 1984). The anthers here are more or less hook- to S-shaped, while the alternipetalous anthers are usually curved, sickle-shaped (Fig. 4a). In a few species, the orientation of all anthers is straight, e.g. *D.bakhuizenii* Veldkamp, *D.inappendiculata* Blume and *D.vacillans* (Blume) Blume (Fig. 4b). The oppositipetalous anthers are usually thicker and shorter than the alternipetalous ones. Their thecae are smooth and glabrous except in *D.fallax* where they are tessellate-reticulate.

The connective of the alternipetalous anthers can be sterile, without thecae, in the basal part. This sterile zone is the pedoconnective and is found in some Melastomataceae and varies in size relative to the size of the stamens (Kadereit 2006, Wong 2016). In the oppositipetalous anthers, a pedoconnective is rare or not developed. The base of the pedoconnective usually has basal appendages (basal crest), which are membranous and triangular, hastate, oblong or ligular in shape. Lateral appendages are solitary or paired, filiform to ribbon-like and sometimes divided at the tip (Kartonegoro and Veldkamp 2010). The two appendages in oppositipetalous anthers extend from the lower part of the thecae and are adaxially bifid, ligular, or have spuriform appendages and, laterally or basally, there may or may not be a pair of filiform appendages.

***Pollen***

Although the stamens of *Dissochaeta* are diverse and display many different shapes and orientations, the pollen is uniform and is not of much taxonomic use. It has been described as 3-colpate with the colpi alternating with three pseudocolpi, prolate, 14‒20 × ca. 11 µm, with a psiIate or smooth exine (Maxwell 1984).

***Staminodes***

Staminodes are found in several species of *Dissochaeta*. Species included in *Diplectria* by Bakhuizen van den Brink *f.* (1943), Veldkamp et al. (1979) and Maxwell (1984) have staminodes in alternipetalous stamen whorl. They have anthers with undeveloped thecae, which are terete, ligular or triangular and infertile and lack the pedoconnective. However, the filaments, basal crest and lateral appendages are well developed, similar to the fertile alternipetalous stamens that are present in many species. These staminodes are functional in order to increase the attraction of the flowers by their colourful appendages and they might signal a large amount of available pollen (Kadereit, person. comm.). Oppositipetalous staminodes are different and have small thecae, ± ⅓ of the length of the alternipetalous ones with minute or well developed connective appendages and with or without lateral appendages. Differing from those previously, it seems that these staminodes are just stamen rudiments without function (Kadereit, person. comm.).

***Gynoecium***

The height of the ovary ranges from about ⅓ to nearly the length of the hypanthium. The ovary is glabrous, villous or has several bristly hairs at the tip where it joins with the style. The ovary apex is usually rounded or conical to mammiform in a few species, like *D.bakhuizenii* and *D.nodosa* Korth. The placentation in *Dissochaeta* is similar to that of the other genera in the tribe (except for a few *Creochiton* species, Kartonegoro and Veldkamp 2013), with a single placenta in each of the four locules, axillary attached to the middle of the central column. The style in bud is straight, but slightly curved at maturity, especially at the apex. The curved orientation of the mature style is usually opposite to that of the filaments. In the heterantherous species like *D.divaricata* (Willd.) G.Don, *D.glabra* and *D.viminalis*, the filaments of the two whorls are bent differently (Kadereit, person. comm.). The style is glabrous except in a few species where it is pubescent. The stigma of all species is capitate, but minute and inconspicuous.

***Extra-ovarial chambers***

Between the hypanthium and the ovary, there are usually septa which form between the chambers. These chambers are known as extra-ovarial chambers and the stamens develop from here (Bakhuizen van den Brink *f.* 1943, Hansen 1984, Maxwell 1984, Kartonegoro and Veldkamp 2010). The number and depth of these extra-ovarial chambers depend on the number and size of the fertile stamens. Usually, there are 4 or 8 chambers, which vary from shallow to reaching the base of the ovary (Kartonegoro and Veldkamp 2010). The depth of the chambers was used by Bakhuizen van den Brink *f.* (1943) to separate *Backeria* and *Neodissochaeta* from *Dissochaeta*.

***Fruits***

The fruit in Tribe Dissochaeteae, including *Dissochaeta*, is baccate (berry) with mainly a subglobose, ovoid to urceolate shape. The indumentum resembles that of the hypanthium. The colour is green at first, then becomes dark blue to purple when ripe. Some species like *D.biligulata* and *D.gracilis* have 8 distinct lines on the surface of the fruits, while in *D.leprosa* and *D.spectabilis*, 8 ridges are also common. When fruiting, the remnants of the calyx lobes are sometimes persistent in an erect or downward reflexed position or they fall off. Seeds have a cuneate shape, are smooth and flat-topped.

### Distribution and ecology

*Dissochaeta* is distributed in South China to South–East Asia, mainly the Malesian region, including the Nicobar Islands (India). It is found north and south of the equator along the South–East Asian tropical rainforest belt but is absent in the eastern part of the Lesser Sunda Islands (Flores, Sumba and Timor). Borneo is the centre of diversity of the genus with 26 species of which 17 are endemic. From the Philippine Islands to New Guinea, the number of taxa and their abundance declines. The occurrence of the genus in mainland India is questionable (see note under *D.divaricata*).

*Dissochaeta* is found mostly in tropical evergreen and perpetually wet forest with little or no seasonal variation in temperature and rainfall (Maxwell 1984). The species are found predominantly in secondary vegetation or more open places within the primary vegetation, such as tree fall gaps, river margins and roadsides. They climb several metres high and produce their flowering and fruiting branches over the tops of trees and larger shrubs. The genus has nodes which bear large interpetiolar outgrowths, which may help climbing and stabilisation in the same way thorns or hooks do in other scramblers (Clausing and Renner 2001). According to Clausing and Renner (2001), *Dissochaeta* has a faster growth rate than other scramblers and often outcompetes them. The climbing habit is reflected in the very wide wood vessels for hydraulic conductivity and thin-walled fibres for limited mechanical support (Van Vliet 1981). These woody climbers apparently only flower when mature and only on the branchlets which are in an exposed, open position. Branchlets that are not exposed to direct sunlight, regardless of their maturity or height in the forest, do not produce flowers (Maxwell 1984).

The majority of species and varieties revised here are confined to lowland and hilly areas up to 1500 m elevation; however, some taxa are restricted to lowland or montane forest. Some species from the lowland forest are usually found in mixed dipterocarp forest, heath forest or swampy forest. Species occuring in montane forest are *D.alstonii* M.P.Nayar, *D.celebica* Blume, *D.intermedia* Blume, *D.leprosa*, *D.marumioides* Cogn., *D.nodosa*, *D.rectandra* and *D.spectabilis*, which can reach from 1200 to ca. 2500 m altitude. There is no specific flowering and fruiting season, the species flower and fruit throughout the year. Some taxa, like *D.biligulata* and *D.gracilis*, sometimes have flowers and fruits together in the same inflorescence. Individual mature plants that reach the canopy or another suitable open area, regularly flower and fruit, but concurrently with many other individuals of the same species. This suggests that flowering and fruiting may be random, but is perhaps cyclic and may, therefore, be regulated by various environmental factors (Maxwell 1984).

### Taxonomic treatment

#### Dissochaeta

Taxon classificationPlantaeMyrtalesMelastomataceae

Blume, Flora 14: 492. 1831

Map 1

Dissochaeta Blume, Flora 14: 492. 1831. Dissochaetasect.Dissochaeta Blume, Flora 14: 493. 1831. Dissochaetasect.Eudissochaeta Blume ex Endl., Gen. Pl. 1219. 1840, *nom. inval.*Dissochaetasect.Diplostemones Cogn. in Boerl., Handl. Fl. Ned. Ind. 2: 533. 1890, *nom. superfl.* Type: Dissochaetavacillans (Blume) Blume (lectotype, designated by Kartonegoro and Veldkamp 2010, pg. 128).
Dissochaeta Blume sect. Diplectria Blume, Flora 14: 501. 1831. Diplectria (Blume) Rchb., Deut. Bot. Herb.-Buch.: 174. 1841. Type: Diplectriacyanocarpa (Blume) Kuntze (lectotype, designated by Veldkamp et al. 1979, pg. 410) [= Dissochaetadivaricata (Willd.) G.Don].
Aplectrum Blume, Flora 14: 502. 1831 [non Torr. 1826], *nom. inval.* Type: Aplectrumviminale (Jack) Blume (lectotype, designated by Veldkamp et al. 1979, pg. 410) [= Dissochaetaviminalis (Jack) Clausing].
Dalenia Korth. in Temminck, Verh. Nat. Gesch. Ned. Bezitt., Bot.: 243. 1844. Dissochaetasect.Dalenia (Korth.) Baill., Hist. Pl. 7: 51. 1877. Type: Daleniapulchra Korth. [= Dissochaetapulchra (Korth.) J.F.Maxwell].
Omphalopus Naudin, Ann. Sci. Nat., Bot. sér. 3, 15: 277. 1851. Dissochaetasect.Omphalopus (Naudin) Baill., Hist. Pl. 7: 51. 1877. Type: Omphalopusfallax (Jack) Naudin (lectotype, designated by Bakhuizen van den Brink *f.* 1943, pg. 118) [= Dissochaetafallax (Jack) Blume].
Anplectrum A.Gray, U. S. Expl. Exped., Phan. 1: 597. 1854. *nom. nov.* for Aplectrum Blume, non. Torr. 1826. Type: Anplectrumviminale (Jack) Triana (designated by Veldkamp et al. 1979, pg. 410) [= Dissochaetaviminalis (Jack) Clausing].
Backeria Bakh.*f.*, Contr. Melastom.: 130. 1943, *nom. superfl.* Type: Backeriaviminalis (Jack) Bakh.*f.* (lectotype, designated by Veldkamp et al. 1979, pg. 410) [= Dissochaetaviminalis (Jack) Clausing].
Neodissochaeta Bakh.*f.*, Contr. Melastom.: 134. 1943, *nom. superfl.* Type: Neodissochaetagracilis (Jack) Bakh.*f.* (lectotype, designated by Kartonegoro and Veldkamp 2010, pg. 128) [= Dissochaetagracilis (Jack) Blume].
Melastoma auct. non Burm. ex L.: Jack, Trans. Linn. Soc. London 14: 3. 1823; Blume, Bijdr. Fl. Ned. Ind. 17: 1067. 1826. *p.p.*, excl.type.

##### Description.

Woody climbers, scrambling; bark greyish, tan to light brown, finely fissured. Branchlets terete or subangular; glabrous to tomentose or floccose with minute stellate or simple glandular or eglandular bristly hairs; sometimes with adventitious roots; nodes swollen with an interpetiolar annular line, ridge or crest, annular or pectinate. Leaves opposite; petioles terete, rarely flattened with a dorsal groove, glabrous to tomentose or with bristly hairs; blades membranous, subcoriaceous or rarely chartaceous with acrodromal venation, ovate to lanceolate, rarely suborbicular, base rounded to cordate, margins entire, rarely serrulate, apex acute to acuminate, midnerve with 1 or 2 pairs of lateral veins and 1 pair of intramarginal veins, secondary venation reticulate; nerves typically sunken adaxially, raised abaxially; adaxial side glabrous, rarely with scattered simple bristle hairs, abaxial side glabrous to densely brown tomentose or with dense bristle hairs. Inflorescences terminal, or rarely axillary, many-flowered, thyrses with 2 to 5 ramifications, decussate, ending with 3-flowered cymules; main axis quadrangular, indumentum similar to that of the branchlets; bracts and bracteoles distinct or minute, linear to ovate, glabrous to densely tomentose, mostly inconspicuous and early caducous; pedicels glabrous to tomentose, sometimes with bristle hairs, longer in central flower, shorter in lateral ones. Flowers 4–merous. Hypanthium campanulate, urceolate, tubular or cyathiform, glabrous to densely tomentose, with or without bristle hairs, often with 4 or 8 vertical ridges; calyx lobes truncate or with distinct rounded, triangular or lanceolate lobes, glabrous or with scattered bristle hairs to nearly densely tomentose; petals in bud conical or rounded, tubular or angular, tip rounded to acute or acuminate, contorted; mature petals ovate, obovate or suborbicular, reflexed or not, apex acute or obtuse, base thin, truncate to clawed, symmetric, glabrous, sometimes with appressed hairs at base and apex or margins puberulous. Stamens 4 or 8, heterantherous when 8, alternipetalous and oppositipetalous or 4 alternipetalous only, smooth or tessellate-reticulate, beaked or not, with terminal pore; filaments flattened, straight or curved sideways; anthers basifixed, sometimes medifixed; the alternipetalous ones thinner, when mature straight or curved and sickle-shaped, at base forming a pedoconnective, sometimes locule not developed and of being staminodal, basal crest membranous, triangular, sagittate, hastate or ligular, with or without paired filiform lateral appendages; the oppositipetalous ones thicker, when mature straight or curved with hooked or S-shaped, connective ridge with erose, bifid or spur-like appendages, basally with or without filiform appendages, sometimes reduced and staminodial less than ⅓ as long as the alternipetalous or absent. Ovary ⅓ to nearly as long as the hypanthium, apex glabrous to densely villous, sometimes with scattered bristle hairs, 4-locular; style straight or curved and hooked at the tip when mature; stigma minute, capitate; ovary concrescent with the hypanthium, with or without 4 or 8 longitudinal septa forming extra-ovarial chambers for the anthers, shallow to reaching to the base of the ovary. Fruits baccate, globose, ovoid to urceolate, dark blue or purple when mature, sometimes with four prominent erect or reflexed calyx remnants, glabrous to floccose; some with distinct vertical ridges. Seeds numerous, cuneate, smooth, flat-topped.

##### Distribution.

**Map 1.**
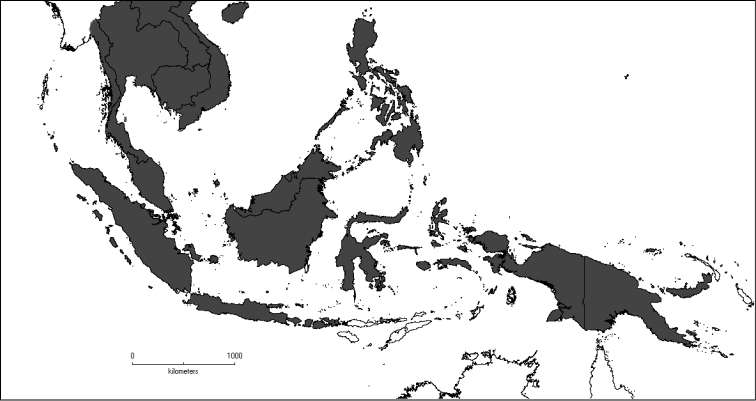
Distribution of *Dissochaeta* (grey).

The genus has 54 species and two varieties which are distributed in South China to South–East Asia, mainly in the Malesian Region (Map 1). It is present in South China (Hainan; Chen and Renner 2007), Myanmar, Indochina (Cambodia, Laos and Vietnam), the Nicobar Islands, Thailand and throughout Malesia except for the eastern part of the Lesser Sunda Islands (Flores, Sumba and Timor). Borneo is the centre of its distribution with almost 50% of the species. Some species also have a restricted distribution.

##### Ecology.

The genus is found predominantly in secondary vegetation or more open places within the primary vegetation, such as tree fall gaps, river margins and roadsides (Kadereit 2006) in evergreen forest, mixed dipterocarp forest, heath forest, hilly forest, swamp forest and montane forest. The plants climb several metres high and produce their flowering and fruiting branches over the tops of small trees and larger shrubs at the end of branches that are in the open.

##### Notes.

From its inception in 1831, several authors created infrageneric classifications (Blume 1831a, 1831b, Endlicher 1840, Naudin 1851, Miquel 1855, Baillon 1877, Cogniaux 1890, 1891, Krasser 1893, Merrill 1917, Maxwell 1980b), which were based on floral characters that are highly variable. Therefore, we here refrain from using an infrageneric classification until a phylogenetic analysis shows the various clades that can be recognised morphologically.

#### Key to species of *Dissochaeta*

**Table:** 

1	Nodes of branchlet swollen with a raised prominent annular crest-like, collar-shaped, pulvinate or pectinate interpetiolar ridge, more than 2 mm in length from base	**2**
–	Nodes of branchlet swollen with a line or raised interpetiolar ridge, less than 2 mm in length from base	**16**
2	Interpetiolar ridge with pectinate appendages, apex acute, up to 10 mm long from base and ca. 1 mm wide, pointing upwards and downwards from base (Borneo)	*** D. latifolia ***
–	Interpetiolar ridge with annular crest-like, collar-shaped or pulvinate appendages, pointing horizontally, 3–6 mm long from base and as wide as nodes	**3**
3	Branchlets and leaf blades abaxially glabrous to stellate-puberulous or –punctate	**4**
–	Branchlets and leaf blades abaxially densely stellate-furfuraceous to tomentose	**15**
4	Leaf blades underneath glabrous, with a pair of glandular patches at base in the axil of the lateral nerve	**5**
–	Leaf blades underneath glabrous or stellate-puberulous or -punctate, without a pair of glandular patches at base in the axil of the lateral nerve	**11**
5	Inflorescences 30–90 cm long; anthers of alternipetalous stamens with undeveloped thecae, staminodes flattened	**6**
–	Inflorescences 10–25 cm long; anthers of alternipetalous stamens with developed thecae, fertile, rostrate (Borneo)	**7**
6	Inflorescences up to 90 cm long with 4 or 5 ramifications; hypanthium cyathiform-tubular; staminodes with a triangular basal crest, base emarginate or hastate, up to 2 mm long (Borneo)	*** D. glabra ***
–	Inflorescences 30–35 cm long with 3 or 4 ramifications; hypanthium suburceolate; staminodes with a triangular basal crest, base emarginate or hastate, ca. 1 mm long (New Guinea)	*** D. papuana ***
7	Leaf blades membranous, ovate or suborbicular, margin slightly serrulate; petal bud enclosed within a calyptra showing constriction with hypanthium	*** D. pulchra ***
–	Leaf blades subcoriaceous, ovate to elliptic, margin entire; petal bud not enclosed within a calyptra	**8**
8	Bracts linear; bracteoles subulate, stellate-furfuraceous, 0.5–1 mm long	**9**
–	Bracts elliptic to oblong; bracteoles ovate to oblong, glabrous, whitish, 6–14 mm long	**10**
9	Branchlets and interpetiolar crest densely covered with simple 1–2 mm long bristle hairs; leaf base subcordate; inflorescences terminal; hypanthium campanulate, 2–3 mm long	*** D. sarawakensis ***
–	Branchlets and interpetiolar crest glabrous; leaf base rounded; inflorescences terminal and axillary; hypanthium cyathiform, cup-shaped, 3–4 mm long	*** D. laevis ***
10	Bracteoles ovate, 6–8 mm long; hypanthium tubular 4–7 × 2–3 mm, calyx lobes truncate with undulate tip; fruits subglobose to ovoid, 5–8 × 3–6 mm	*** D. beccariana ***
–	Bracteoles oblong to lanceolate, ca. 14 × 3–4 mm; hypanthium campanulate 5–6 × 4–5 mm, calyx lobes truncate, level; fruits urceolate, ca. 10 × 6 mm	*** D. glandulosa ***
11	Leaf blades abaxially stellate puberulous or punctate; calyx lobes truncate with widened undulate tip; alternipetalous stamens fertile, anther thecae lanceolate, pedoconnective developed	**12**
–	Leaf blades abaxially glabrous; calyx lobes truncate with erect apiculate tip, 0.3–0.5 mm long; alternipetalous stamens staminodial, thecae undeveloped, flattened, 0.5–4 mm long, pedoconnective not developed	**13**
12	Leaf blades underneath stellate-puberulous, 3–5.25 cm wide; calyx lobes ca. 1 mm long; alternipetalous stamens with thecae 4–5 mm long, pedoconnective ca. 0.5 mm long, lateral appendages absent; ovary with puberulous apex	*** D. bakhuizenii ***
–	Leaf blades underneath stellate-punctate, 5–9.2 cm wide; calyx lobes 1–1.5 mm long; alternipetalous stamens with thecae 7–8 mm long, pedoconnective 1.5–2 mm long, lateral appendages paired, filiform, 2–2.5 mm long; ovary with pubescent and bristly apex (Peninsular Malaysia)	*** D. rectandra ***
13	Leaf blades ovate; petiole glabrous and dorsally with bristle hairs; hypanthium urceolate, glabrous (Borneo)	*** D. micrantha ***
–	Leaf blades elliptic to elliptic-oblong; petiole densely brown stellate-furfuraceous and covered with scattered or dense bristle hairs; hypanthium cyathiform, tubular or campanulate, densely furfuraceous or pubescent	**14**
14	Interpetiolar ridge and petioles densely covered with erect, thick, 4–6 mm long bristle hairs; leaf blades membranous; bracts oblong-lanceolate, margin ciliate; hypanthium tubular-campanulate (Borneo)	*** D. maxwellii ***
–	Interpetiolar ridge and petioles sparsely covered with slender, ca. 2 mm long bristle hairs; leaf blades subcoriaceous; bracts linear, margin not ciliate; hypanthium tubular-cyathiform	*** D. stipularis ***
15	Leaf blades subcoriaceous, ovate to suborbicular, nervation with 1–2 pairs of lateral nerves; calyx lobes truncate with indistinct apex; petal bud enclosed with calyptra showing constriction with hypanthium (Borneo). ***D.pubescens***
–	Leaf blades membranous, ovate-elliptic, nervation with 1 pair of lateral nerves; calyx lobes distinct with 2–2.5 mm long triangular or rounded apex; petal bud not enclosed with calyptra (Sumatra)	*** D. glandiformis ***
16	Branchlets, petioles, inflorescence axes, hypanthium and fruits covered with dense or scattered simple erect or curved bristle hairs	**17**
–	Branchlets, petioles, hypanthium and fruits not covered with dense or scattered simple erect or curved bristle hairs	**27**
17	Calyx lobes triangular or truncate with a slightly triangular apex, 1–3 mm long	**18**
–	Calyx lobes linear-lanceolate, 3–11 mm long	**25**
18	Leaf blades densely covered with bristle hairs on both sides	**19**
–	Leaf blades devoid of bristle hairs on both sides or covered only on midnerve underneath	**23**
19	Bracts and bracteoles oblong, sparsely stellate-puberulous, densely bristly only along the margin (Borneo)	*** D. rostrata ***
–	Bracts and bracteoles linear, densely stellate-furfuraceous and densely bristly on both surfaces	**20**
20	Hypanthium campanulate or tubular, 4–5.5 mm long; fruits subglobose, 4–6 mm long	**21**
–	Hypanthium suburceolate, 6–10 mm long; fruits subglobose, 8–10 mm long	**22**
21	Bristles on branchlets capitate (apex glandular); leaf blades coriaceous; calyx lobes 2.5–3 mm long; petal buds glabrous at tip; calyx remnant in fruits reflexed (Sumatra)	*** D. alstonii ***
–	Bristles on branchlets not capitate (apex eglandular); leaf blades membranous; calyx lobes ca. 1 mm long; petal buds bristly at tip; calyx remnant in fruits erect (Borneo)	*** D. hirsutoidea ***
22	Hypanthium 5–6 × 2–2.5 mm, calyx lobes with acute apex, up to 2 mm long; petal buds glabrous at tip (Peninsular Malaysia & Sumatra)	*** D. johorensis ***
–	Hypanthium 8–10 × 3–4 mm, calyx lobes with undulate apex, up to 1.5 mm long; petal buds bristly at tip (Peninsular Malaysia)	*** D. malayana ***
23	Branchlets covered with minute stellate hairs and dense dark red-brown bristle hairs; nodes thickly covered with stellate hairs and thick brown bristle hairs; bracteoles subulate, densely bristly; stamens with minute basal crest, less than 1 mm long (Borneo) –Inflorescences terminal and axillary	*** D. atrobrunnea ***
–	Branchlets covered with minute stellate hairs and scattered bristle hairs; nodes without bristle hairs; bracteoles oblong or lanceolate, lacking bristle hairs; stamens with triangular or ligular basal crest, 1–4 mm long –Inflorescences terminal	**24**
24	Leaf blades subcoriaceous, with prominent nervation; bracteoles 10–15 × ca. 2 mm; hypanthium campanulate, 8–10 × 5–7 mm, calyx lobes with acute apex, ca. 3 mm long; petal buds without bristle hairs at apex; fruits urceolate, 13–15 × 5–10 mm (Peninsular Malaysia)	*** D. griffithii ***
–	Leaf blades membranous, without prominent nervation; bracteoles ca. 3.5 × 1 mm; hypanthium tubular, ca. 4 × 2 mm, calyx lobes with obtuse apex, ca. 1 mm long; petal buds bristly at apex; fruits ovoid, 5–6 × 3–3.5 mm (Borneo)	*** D. densiflora ***
25	Bristle hairs on branchlets, petioles and hypanthium curved; leaf blades membranous, hirsute, covered with curved bristle hairs on both surfaces, 9.7–12 cm long; hypanthium campanulate, 5–6 mm long, calyx lobes 3–4 mm long; petal buds with bristly apex (Borneo)	*** D. porphyrocarpa ***
–	Bristle hairs on branchlets, petioles and hypanthium straight; leaf blades subcoriaceous, above glabrous, below densely pubescent or floccose, 13–20 cm long; hypanthium tubular or suburceolate, 7–9 mm long, calyx lobes 6–11 mm long; petal buds with glabrous apex (Sumatra)	**26**
26	Branchlets, petioles and hypanthium axes pubescent and with 4–5 mm long bristle hairs; bracteoles subulate, ca. 2 mm long	*** D. horrida ***
–	Branchlets, petioles and hypanthium axes floccose and with 1–2 mm long bristle hairs; bracteoles subulate, 5–10 mm long	*** D. floccosa ***
27	Inflorescences axillary, up to 10 cm long, thyrse with 1–20 flowers	**28**
–	Inflorescences terminal, 12–57 cm long; thyrse with more than 20 flowers	**31**
28	Branchlets, leaf underneath and hypanthium glabrous; alternipetalous stamens staminodal, anther locules undeveloped, flattened, pedoconnective not developed	**29**
–	Branchlets, leaf blades underneath and hypanthium stellate-furfuraceous to tomentose; alternipetalous stamens fertile, thecae developed, C-shaped, pedoconnective developed	**30**
29	Leaf blades subcoriaceous, petioles glabrous; hypanthium 7–8 × ca. 5 mm, calyx lobes with irregularly cracked or rounded tip, 1–2 mm long; extra-ovarial chambers extending almost to base of ovary; fruits 7–8 × 5–6 mm	*** D. conica ***
–	Leaf blades membranous, petioles densely covered with red-brown bristles at lateral groove at attachment with blade; hypanthium 3–4 × 2–2.5 mm, calyx lobes truncate, ca. 0.5 mm long; extra-ovarial chambers extending only to ⅓ of upper part of ovary; fruits 4–5 × 3–4 mm	*** D. viminalis ***
30	Branchlets glabrescent; leaf blades membranous, base subcordate; inflorescences with 3–10 flowers; hypanthium tubular or funnelform; alternipetalous stamens with 3–4 mm long pedoconnective (Philippines)	*** D. acmura ***
–	Branchlets densely stellate-tomentose; leaf blades subcoriaceous, base rounded; inflorescences with 15–20 flowers; hypanthium campanulate; alternipetalous stamens with ca. 5 mm long pedoconnective	*** D. axillaris ***
31	Flowers with 4 alternipetalous stamens; oppositipetalous stamens absent or not developed	**32**
–	Flowers with 8 stamens, both alternipetalous and oppositipetalous well developed or staminodial	**42**
32	Calyx lobes truncate with acute or triangular tip	**33**
–	Calyx lobes slightly triangular or lanceolate with acute tip	**40**
33	Leaf base subcordate; anthers medifixed, with tessellate-reticulate thecae, basal crest triangular, orbicular or ligular, 2–3 mm long, lateral appendages absent or not developed; pedoconnective not developed	*** D. fallax ***
–	Leaf base rounded; anthers basifixed, with smooth thecae, basal crest triangular or hastate, 0.5–1 mm long, lateral appendages paired, filiform or ribbon-like; pedoconnective developed	**34**
34	Hypanthium suburceolate or urceolate; stamens with straight or curved anthers when mature	**35**
–	Hypanthium campanulate; stamens with curved anthers when mature	**38**
35	Hypanthium robust, 5–8 × 3–4 mm; stamens with curved anthers	*** D. leprosa ***
–	Hypanthium small, 3–5 × 2–2.5 mm; stamens with straight anthers	**36**
36	Petiole 6–8 mm long; inflorescences short, 10–15 cm long; pedicels those of central flowers 1–2 mm long, those of lateral flowers ca. 0.5 mm long; fruits subglobose with 8 lines	*** D. biligulata ***
–	Petiole 10–17 mm long; inflorescences long, 15–57 cm long; pedicels those of central flowers 2–4 mm long, those of lateral flowers 1–3 mm long; fruits ovoid-urceolate without lines	**37**
37	Bracteoles minute, less than 1 mm long; calyx lobes distinctly triangular, erect persistent when fruiting, 1–2 mm long (Borneo)	*** D. rubiginosa ***
–	Bracteoles linear, 1–2 mm long; calyx lobes truncate with triangular point, widened or reflexed persistent when fruiting, less than 1 mm long (Moluccas & New Guinea)	*** D. angiensis ***
38	Leaf blades abaxially, petioles and inflorescence axes glabrous or sparsely puberulous (Java & Lesser Sunda)	*** D. vacillans ***
–	Leaf blades abaxially, petioles and inflorescence axes stellate-furfuraceous or tomentose	**39**
39	Leaf blades underneath densely tomentose; hypanthium campanulate-angular with 4 ridges; petal buds 3–7 mm long; mature petals ovate to oblong, 6–10 × 3–5 mm, pink; extraovarial chambers deep, extending to base of ovary (Java)	*** D. intermedia ***
–	Leaf blades underneath stellate-furfuraceous; hypanthium campanulate-terete without ridges; petal buds 2–2.5 mm long; mature petals obovate, 3–4 × ca. 2 mm, dark purple; extraovarial chambers shallow, extending to upper ⅓ of ovary (Sulawesi & Philippines)	** D. celebica var. celebica **
40	Bracteoles minute, subulate, 1–2 mm long; hypanthium tubular or funnel-form; stamens with straight filaments and anthers when mature, basal crest ligular, ca. 2 mm long (New Guinea)	*** D. brassii ***
–	Bracteoles linear, 2–5 mm long; hypanthium campanulate; stamens with curved filaments and anthers when mature, basal crest triangular or rounded, ca. 0.5 mm long	**41**
41	Leaf blades underneath stellate-furfuraceous; central flowers with 1–2 mm long pedicels, in lateral flowers ca. 0.5 mm long; calyx lobes persistent when fruiting (Sulawesi)	** D. celebica var. longilobata **
–	Leaf blades underneath stellate-tomentose; central flowers with 3–4 mm long pedicels, in lateral flowers 1–2 mm long; calyx lobes caducous when fruiting (New Guinea)	*** D. schumannii ***
42	Alternipetalous stamens fertile, well developed; oppositipetalous fertile or staminodal	**43**
–	Alternipetalous stamens staminodal, not well developed; oppositipetalous ones fertile, well developed	**56**
43	Alternipetalous and oppositipetalous stamens distinctly unequal; oppositipetalous ones less than half the length of alternipetalous ones	**44**
–	Alternipetalous and oppositipetalous stamens subequal or equal in length; oppositipetalous ones more than half the length of the alternipetalous ones	**45**
44	Leaf blades underneath glabrous; hypanthium glabrous; filaments curved sideways, alternipetalous and oppositipetalous anthers curved, whitish, with a pair of wavy filiform lateral appendages	*** D. gracilis ***
–	Leaf blades underneath stellate-furfuraceous; hypanthium glabrescent to stellate-furfuraceous; filaments straight, alternipetalous and oppositipetalous anthers straight, yellow, without lateral appendages	*** D. inappendiculata ***
45	Leaf blades subcoriaceous, coriaceous or chartaceous, underneath stellate punctate	**46**
–	Leaf blades membranous, underneath glabrous, stellate-furfuraceous or tomentose	**50**
46	Hypanthium densely stellate-tomentose; calyx lobes slightly 4-triangular with acute apex	**47**
–	Hypanthium glabrous to stellate-furfuraceous; calyx lobes truncate with acute apex	**49**
47	Hypanthium tubular to suburceolate, calyx lobes lanceolate, 4–4.5 mm long, reflexed when mature (Borneo)	*** D. macrosepala ***
–	Hypanthium campanulate, calyx lobes triangular, 1–3 mm long, erect when mature	**48**
48	Bracteoles linear, 2–3 mm long; hypanthium at early buds urceolate or subglobose, enclosing the petal bud; petal buds 2–4 mm long; mature petals ca. 10 × 5–7 mm; stamens with a fimbriate basal crest; fruits subglobose to urceolate, 8–10 × 4–7 mm (Peninsular Malaysia & Sumatra)	*** D. punctulata ***
–	Bracteoles lanceolate or oblong, up to 10 mm long; hypanthium at early buds campanulate, not enclosing the petal bud; petal buds 7–8 mm long; mature petals 12–20 × 10–14 mm; stamens with triangular or erose basal crest; fruits ovoid to urceolate, 13–15 × 5–10 mm	*** D. annulata ***
49	Hypanthium stellate-furfuraceous; bracteoles conspicous, ovate to oblong, 5–9 mm long, enclosing the hypanthium	*** D. bracteata ***
–	Hypanthium glabrous; bracteoles inconspicous, linear or lanceolate, 2–4 mm long, caducous	*** D. pallida ***
50	Calyx lobes triangular or rounded with obtuse or acute tip, 2–4 mm long. **51**
–	Calyx lobes truncate with 4 acute or triangular points, 0.5–1 mm long	**52**
51	Hypanthium densely stellate-furfuraceous and with scattered, thickened, 1–2 mm long bristles; calyx lobes rounded, apex obtuse, margin ciliate, ca. 2 mm long; petal buds 3–5 mm long (Sumatra)	*** D. marumioides ***
–	Hypanthium densely brown stellate-tomentose, slightly 8-ridged and without bristles; calyx lobes triangular, apex acute, margin not ciliate, 3–4 mm long; petal buds 8–10 mm long (Peninsular Malaysia & Sumatra)	*** D. spectabilis ***
52	Branchlets, leaf blades underneath, inflorescence axes and hypanthium glabrous, glabrescent to sparsely puberulous	**53**
–	Branchlets, leaf blades underneath, inflorescence axes and hypanthium densely furfuraceous to tomentose	**54**
53	Hypanthium narrowly campanulate to suburceolate, 1–3 mm in width, calyx lobes with undulate or rounded apex, 0.5–1 mm long; stamens with a pair of filiform lateral appendages (Java)	*** D. vacillans ***
–	Hypanthium broadly campanulate, ca. 3 mm in width, calyx lobes with acute, triangular apex, ca. 1 mm long; stamens lacking lateral appendages (Sumatra)	*** D. nodosa ***
54	Bracteoles linear, ca. 1.5 mm long; hypanthium suburceolate; thecae tesselate-reticulate, oppositipetalous stamens with ligular basal appendages and lacking lateral appendages	*** D. fallax ***
–	Bracteoles linear-lanceolate, 3–6 mm long; hypanthium campanulate; thecae smooth, oppositipetalous stamens with spuriform basal appendages and a pair of filiform lateral appendages	**55**
55	Leaf blade base emarginate or subcordate; petioles 8–10 mm long; bracteoles linear, 3–4 mm long; hypanthium campanulate-angular, ca. 2 mm wide (Philippines)	*** D. cumingii ***
–	Leaf blade base rounded; petioles 10–15 mm long; bracteoles lanceolate, 5–6 mm long; hypanthium campanulate-terete, 3–5 mm wide (Java)	*** D. sagittata ***
56	Leaf blades membranous or subcoriaceous; hypanthium cyathiform-tubular or cup-shaped; fruits subglobose	**57**
–	Leaf blades chartaceous; hypanthium campanulate-angular to suburceolate, slightly 4- or 8-lined; fruits urceolate	**58**
57	Leaf blades subcoriaceous, petioles glabrous; hypanthium 7–8 × ca. 5 mm, calyx lobes with irregularly cracked tip or rounded apex, 1–2 mm long; extra-ovarial chambers extending almost to base of ovary	*** D. conica ***
–	Leaf blades membranous, petioles densely covered with red-brown bristles at lateral groove near the attachment with base of leaf blade; hypanthium 3–4 × 2–2.5 mm, calyx lobes indistinct, ca. 0.5 mm long; extra-ovarial chambers extending only to upper ⅓ of ovary	*** D. viminalis ***
58	Leaf blades underneath glabrous but with sparsely stellate hairs along the nerves, with a pair of glandular patches at base; petiole glabrous to stellate-puberulous, covered with bristle hairs at dorsal line, 5–8 mm long; calyx lobes with minutely pointed tips, ca. 0.5 mm long	*** D. barbata ***
–	Leaf blades underneath glabrous or stellate-furfuraceous, without a pair of glandular patches at base; petiole sparsely to densely covered with stellate hairs and often with dense bristle hairs, 10–15 mm long; calyx lobes without distinct tip, ca. 1 mm long	*** D. divaricata ***

#### 1. Dissochaeta acmura

Taxon classificationPlantaeMyrtalesMelastomataceae

Stapf & M.L.Green, Bull. Misc. Inform. Kew: 42. 1913.

Map 2

##### Type.

Philippines. Luzon: Province of Albay, H. Cuming 2838 (lectotype, designated here: K [K000859613]!).

**Map 2.**
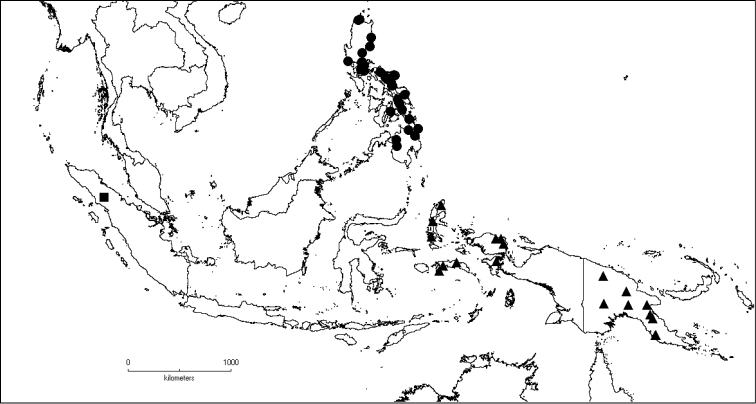
Distribution of *D.acmura* (●), *D.alstonii* (■) and *D.angiensis* (▲).

##### Description.

Climbing up to 8 m in height. Branchlets terete, 2.5–4 mm in diameter, covered with stellate hairs, dense on young branches and nodes, glabrescent; nodes swollen, with interpetiolar ridge; internodes 5–10 cm long. Leaves: petioles flattened, 10–23 mm long, densely stellate-furfuraceous; blades ovate to elliptic, 8.3–17.2 × 3.7–6.8 cm, membranous, rarely subcoriaceous, base rounded or subcordate, margin entire, apex acute or acuminate, tip 0.5–2 cm long; nervation prominent above, with 1 pair of lateral nerves, 1 pair of intramarginal nerves; adaxially glabrous, abaxially brown stellate-tomentose, dense on midrib. Inflorescences axillary, 5.5–10 cm long, 3–10 flowers; main axis terete, densely stellate-furfuraceous and with simple glandular hairs; primary axes 11–13 cm long, with 3 or 4 nodes; secondary axes 1–1.4 cm long, with 1 or 2 nodes; tertiary axes 0.8–1 cm long, with 1 node or undeveloped; bracts linear, ca. 5 mm long, densely stellate-furfuraceous, caducous; bracteoles linear, 1–2 mm long, densely stellate-furfuraceous, caducous; pedicel densely stellate-furfuraceous, 5–7 mm long in central flowers, 2–3 mm long in lateral flowers. Hypanthium tubular or slightly funnel-shaped, 7–11 × 3–5.5 mm, densely stellate-tomentose, sparsely covered with scattered simple glandular bristle hairs; calyx lobes truncate with 4 minute tips, 1–3 mm long; petal buds conical, 8–13 × 3–9 mm; mature petals obovate to suborbicular, 18–20 × 13–15 mm, reflexed or not, base clawed, apex obtuse, glabrous, white or white with pinkish hue, hairy at edge. Stamens 8, unequal, glabrous, filaments curved sideways; alternipetalous stamens with white-creamy filaments, 10–12 mm long, apex yellow, anthers slightly curved, sickle-shaped, slender, thecae 10–12 mm long, pink, pedoconnective 3–4 mm long, basal crest bifid up to 2 mm long, lateral appendages paired, filiform, white, 3–3.5 mm long; oppositipetalous stamens with white filaments, ca. 10 mm long, anthers S-shaped, thecae 10–12 mm long, pink, basal crest minute or ligular, ca. 0.5 mm long, margin erose, lateral appendages paired, filiform, 5–6 mm long, bright white. Ovary ¾ of hypanthium in length, apex glabrous, 8-ridged; style 17–22 mm long, glabrous, curved sideways, opposite to the filaments, slightly curved at the end when mature, whitish; stigma minute; extra-ovarial chambers 8, the 4 alternipetalous ones extending to near the base of the ovary, the 4 oppositipetalous ones extending to about the middle of the ovary. Fruits urceolate, ellipsoid, 8–15 × 5–10 mm, stellate-furfuraceous, brownish green when unripe; calyx remnants persistent, erect, widened. Seeds ca. 0.5 mm long.

##### Distribution.

Philippines.

##### Ecology and habitat.

Open shade, along ridges, on wet or less sandy ground in forest or secondary forest at 220–300 m elevation.

##### Note.

*Dissochaetaacmura* resembles *D.axillaris* in having axillary inflorescences, but differs in its membranous leaf blades and triangular mature calyx lobes, while *D.axillaris* has coriaceous leaf blades and truncate calyx lobes.

##### Specimens examined.

**PHILIPPINES. Biliran**: Mt. Suiro, 30 Apr 1954, M.D. Sulit PNH 21567 (K, L, PNH). **Catanduanes**: M. Ramos BS 30366 (BM, P); San Miguel, 21 Nov 1991, Barbon, Garcia & Alvarez PPI 2425 (L). **Cebu**: Cebu Central, 800 m, May 1998, D. Bicknell 1490 (L). **Leyte**: 1 May 1914, C.A. Wenzel 414 (BM); 14 May 1914, C.A. Wenzel 662 (BM); Baybay, Villa Solidaridad, 150 m, H.O. Gutierrez PNH 119985 (PNH). **Luzon**: Aurora, Diteki, 550 m, 9 May 1996, R.U. Fuentes & L. Fernando PPI 37255 (L); *Ibid.*, 450 m, 19 May 1996, R.U. Fuentes & L. Fernando PPI 37459 (L); *Ibid.*, Casiguran, Bianoan, 19 Mar 1993, Barbon, Garcia & Fernando PPI 9293 (L); Cagayan, Claveria, 467 m, 3 Aug 1995, Garcia et al. PPI 18314 (L); *Ibid.*, Santa Praxedes, 520 m, 11 Aug 1995, Garcia, Fuentes & Romero PPI 18494 (L); Camarines Norte, Basud, Mount Nilisan, 9 Sep 1991, Reynoso, Romero & Fuentes PPI 1317 (L); Camarines Sur, Iriga, Mt. Asog, 14 Jun 1992, Barbon, Romero & Fuentes PPI 8440 (L); Laguna, Jun-Aug 1915, R.C. MacGregor BS 22859 (BM, P); Zambales, San Antonio, Aug 1910, M. Ramos BS 437 (U); *Ibid.*, Sep–Oct 1912, M. Ramos BS 16612 (BM, L); San Mariano, SO. Agal, 200 m, 2 Jul 1994, Barbon, Romero R.U. Fuentes PPI 13023 (L); Sorsogon, Irosin, Mt. Bulusan, 300 m, Dec 1915, A.D.E. Elmer 15257 (BM, BO, K, L, P, PNH, U), *Ibid.*, 300 m, Aug 1916, A.D.E. Elmer 16844 (BM, BO, K, L, P, PNH, U); *Ibid.*, Lake Polog, Aug 1915, M. Ramos BS 23644 (BM, BO, K, P, PNH); Albay Province, H. Cuming 2838 (K); Quezon Province, Lalawinan, Tipuan, 2 Sep 1991, Barbon, Alvarez & Garcia PPI 2212 (K, L); *Ibid.*, Burdeos, Kinabuawan, 24 Aug 1991, Barbon, Alvarez & Garcia PPI 2037 (K, L); *Ibid*., Tayabas, H. Cuming 815 (BM, K); *Ibid.*, H. Cuming 2840 (K); *Ibid.*, Lucban, May 1907, A.D.E. Elmer 8236 (BM, BO, K, L, P, PNH, U); *Ibid*., Mt. Tulaog, May 1917, M. Ramos & G.E. Edano BS 29114 (BO). **Mindanao**: Agusan Del Norte, Butuan, San Mateo, Tungao, 250 m, 31 May 1961, D. Mendoza PNH 41847 (BO, K, L, PNH); *Ibid.*, 8 Jun 1961, D. Mendoza PNH 42217 (L, PNH); *Ibid.*, 85 m, 25 Aug 1966, S. Jurane PNH 98404 (PNH); Agusan del Sur, Dinagat, Binahanan, 80 m, 9 Oct 1991, Gaerlan, Sagcal & Fernando PPI 4855 (L); Lanao del Sur, Lake Lanao, Camp Keithley, 1907, M.S. Clemens 1155 (BO); Surigao, 3 Jul 1927, C.A. Wenzel 3043 (BO, K); Surigao del Sur, Aras-Asan, 250 m, 17 May 1975, University of San Carlos 819 (L); Cotabato, Mabuhay Mining Camp, 25 May 1950, Aňonuevo PNH 13453 (BM, PNH). **Samar**: Oquendo, Mt. Mahagna, 220 m, 17 Apr 1951, M.D. Sulit PNH 14463 (K, L, PNH); *Ibid.*, 30 Apr 1951, M.D. Sulit PNH 14514 (BM, K, L, PNH); Paranas, 17 Oct 1992, Reynoso, Sagcal & Garcia PPI 7448 (L); *Ibid.*, 400 m, 1 May 1996, Reynoso & Majaducon PPI 21956 (L).

#### 2. Dissochaeta alstonii

Taxon classificationPlantaeMyrtalesMelastomataceae

M.P.Nayar, Bull. Bot. Surv. India 11: 188. 1969.

Map 2

Dissochaeta rostrata Korth. var. alstonii (M.P.Nayar) J.F.Maxwell, Gard. Bull. Singapore 33: 318. 1980.

##### Type.

Indonesia. North Sumatra: Tapanuli, between Sidikalang and Pongkolan, 1200 m elev., 27 Mar 1954, A.H.G. Alston 14813 (holotype: BM [BM000944479]!).

##### Description.

Branchlets terete, 3–4 mm in diameter, covered with stellate hairs and glandular bristles; nodes swollen, with an interpetiolar ridge; internodes 4–7 cm long. Leaves: petioles terete, 5–8 mm long, densely stellate-furfuraceous; blades ovate, 6–8.5 × 3–4 cm, subcoriaceous, base subcordate, margin entire, ciliate, apex acuminate, tip ca. 1 cm long; nervation prominent above, with 1 or 2 pairs of lateral nerves and 1 pair of intramarginal nerves; adaxially glabrous and with scattered bristle hairs on midrib, abaxially punctate, midrib with dense stellate hairs, brown furfuraceous and bristle hairs. Inflorescences terminal, many-flowered, 20–30 cm long; main axis angular, densely setose with glandular bristles and stellate-furfuraceous hairs; primary axes up to 28 cm long with 6–8 nodes, secondary axes 1.5–4 cm long with 1–3 nodes, tertiary axes up to 1 cm long with 1 node; bracts linear, 3–6 mm long, densely setose with glandular bristles; bracteoles linear, 3–4 mm long, densely setose with glandular bristles; pedicels densely setose with glandular bristles, 2–3 mm long in central flowers, 1–2 mm long in lateral flowers. Hypanthium campanulate, tubular, 4.5–5.5 × 2–3 mm, densely setose and covered with glandular bristles and stellate-furfuraceous hairs; calyx lobes triangular, 2.5–3 mm long, densely setose; petal buds conical, 3–4 mm long; mature petals obovate to ovate-oblong, ca. 4.5 × 2.5 mm, base clawed, apex acuminate, glabrous but glandulose-setose at margin, pink. Stamens 8, unequal, glabrous, filaments curved sideways; alternipetalous stamens with 4–4.5 mm long filaments, anthers rostrate, sickle-shaped, thecae 4.5–5 mm long, pedoconnective ca. 0.5 mm long, basal crest erose, up to 0.4 mm long, lateral appendages subulate, paired, ca. 2 mm long; oppositipetalous stamens with ca. 3.5 mm long filaments, anthers hook-shaped, thecae 3.5–4 mm long, basal crest minute, erose, ca. 0.2 mm long, lateral appendages minute, ca. 0.2 mm long. Ovary half as long as hypanthium, apex stellate-furfuraceous; style 9–11 mm long, glabrous, apex curved; stigma punctiform; extra-ovarial chambers 8, extending to near the base of ovary. Fruits subglobose, 4.5–5.5 × ca. 4 mm, covered with dense glandular bristles and stellate-furfuraceous hairs; calyx lobe remnants persistent, reflexed. Seeds ca. 0.5 mm long.

##### Distribution.

Sumatra.

##### Ecology and habitat.

Montane forest at ca. 1200 m elevation.

##### Note.

*Dissochaetaalstonii* is known only from the type from Northern Sumatra. The species resembles *D.rostrata*, by having a setose and bristly appearance on branchlets and hypanthium. It differs by having glandular bristles in most parts, like branches, leaves and hypanthium while, in *D.rostrata*, the bristles are simple, not glandular.

#### 3. Dissochaeta angiensis

Taxon classificationPlantaeMyrtalesMelastomataceae

Kaneh. & Hatus. ex Ohwi, Bot. Mag. (Tokyo) 57: 5. 1943.

Map 2

##### Type.

Indonesia. West Papua: Arfak Mts., track to Lake Gita from Momi, 1300 m elev., 4 Apr 1940, R. Kanehira & S. Hatusima 13374 (lectotype, designated here: FU *n.v.*; isolectotype: L [L0537256]!).

##### Description.

Climbing up to 7 m in height. Branchlets terete, 3–6 mm in diameter, covered with densely stellate-furfuraceous hairs, rarely pubescent; nodes swollen, interpetiolar ridge slightly raised; internodes 5–12.3 cm long. Leaves: petioles terete, 10–17 mm long, densely stellate-furfuraceous; blades ovate, elliptic to oblong, 8.2–18 × 4–7 cm, membranous to nearly subcoriaceous, base rounded or emarginate, margin entire, rarely subserrulate, apex acuminate, tip 1–1.5 cm long; nervation with 1 (rarely 2) pairs of lateral nerves and 1 pair of intramarginal nerves; adaxially glabrous, dark glossy green, abaxially brown stellate-furfuraceous, dense on midrib. Inflorescences terminal and in the upper leaf axils, many-flowered, up to 57 cm long; main axis angular, densely stellate-furfuraceous; primary axes up to 54 cm long with 6–8 nodes, secondary axes up to 18.5 cm long with 1–3 nodes, tertiary axes 1–4 cm long with 1 or 2 nodes or sometimes undeveloped; bracts linear, ovate or elliptic, 1.7–3.5 × ca. 1.2 cm long, densely brown stellate-furfuraceous, caducous; bracteoles linear, 1–2 mm long, densely stellate-furfuraceous, caducous; pedicels densely stellate-furfuraceous, 3–4 mm long for central flowers, 2–3 mm long for lateral flowers. Hypanthium urceolate, 4–5 × 2–2.5 mm, densely stellate-furfuraceous; calyx lobes truncate with 4 triangular tips, ca. 1 mm long; petal buds conical, 3–4 mm long, blades ovate, base clawed, apex acute; mature petals oblong, 7–9 × 4–6 mm, base clawed, margin ciliate, apex obtuse, glabrous, white to pinkish. Stamens 4, equal, alternipetalous, filaments straight; alternipetalous stamens with 5–7 mm long filaments, anthers oblong or lanceolate, thecae slightly straight, 5–6 mm long, yellow, pedoconnective short or slightly undeveloped, basal crest hastate or triangular, 0.75–1 mm long, lateral appendages ligular, ribbon-like or paired and filiform with irregular margins, 0.75–2 mm long. Ovary ¾ of hypanthium in length, apex villous; style 10–12 mm long, glabrous; stigma capitate, minute; extra-ovarial chambers 4, shallow, extending ca. ⅓ of ovary. Fruits ovoid-urceolate, 5–8 × 2.5–7 mm, glabrous to glabrescent, green when young, calyx remnants persistent, widened. Seeds ca. 0.5 mm long.

##### Distribution.

Moluccas and New Guinea.

##### Ecology and habitat.

Lowland hill forest to lower montane forest at 50–1300 m elevation.

##### Vernacular names.

Moluccas: *siri utan* (Ambon); *arendong* (Bacan). New Guinea: *tsoin* (Kutubu); *johnihoeveke* (middle Waria).

##### Note.

*Dissochaetaangiensis* resembles *D.celebica* Blume but differs by having a much larger urceolate hypanthium and short lateral appendages (0.75–2 mm) on the alternipetalous stamens. The mature anthers are straight rather than curved.

##### Specimens examined.

**INDONESIA. Moluccas**: Bacan, Mt. Damar, Masurung, 200 m, 12 Aug 1937, Nedi 28 (BO); Ceram, Honitetu–Wae Tuba, 4 Feb 1938, P.J. Eyma 2771 (BO, L); *Ibid*., Between Raniki and Manusela, 1000 m, 24 Jun 1918, Kornassi 1403 (BO). **North Moluccas**: Halmahera, Mt. Sembilan, 600 m, 28 Sep 1951, D.R. Pleyte 299 (BO, K, L, PNH); Morotai, Mt. Pare-Pare, Rawa Panjang, 1000 m, 28 May 1949, A.J.G.H. Kostermans 1322 (BO, K, L, PNH). **West Papua**: Vogelkop Peninsula, Ije River Valley, Bamfot Village, 850 m, 2 Nov 1961, P. van Royen & H. Sleumer 7646 (BO, K, L); *Ibid.*, Isjon River Valley, Son Village, 650 m, 28 Oct 1961, P. van Royen & H. Sleumer 7574 (BO, K, L); Arfak Mountains, Angi, 1300 m, 4 Apr 1940, R. Kanehira & S. Hatusima 13374 (L); Bomberai Peninsula, Tangguh, 50 m, 21 Feb 2002, W. Takeuchi, E.N. Sambas & R. Maturbongs 16004 (BO). **PAPUA NEW GUINEA. Central Division**: Sogeri, Subitana, 22 Jun 1954, J.S. Womersley & P. van Royen NGF 5815 (BO, K), *Ibid.*, 1885, H.O. Forbes 459 (BM). **Chimbu**: Haia, 640 m, 16 Sep 1996, W. Takeuchi 11200 (K). **East Sepik**: Waskuk Hills, between Garuka and Waskuk, 60 m, 28 Jun 1995, W. Takeuchi & J.C. Regalado 10198 (L). **Madang**: Bismarck Range, Gulno Village, 1050 m, 15 Oct 1995, W. Takeuchi 10790 (L). **Morobe**: Kipu, Tiaura, 800 m, 7 Jan 1966, H. Streimann NGF 26113 (BO, K, L); Wampit, Bupu Village, 760 m, 3 Mar 1964, A.N. Millar NGF 23243 (L); *Ibid.*, 1310 m, 13 Jul 1967, A.N. Millar NGF 22928 (L); Wareo, 600 m, 25 Dec 1935, J. Clemens & M.S. Clemens 1395 (L). **Southern Highlands**: Tari, Bosavi Mission-Mulimia Govt. Stn., 700 m, 2 Sep 1986, O. Gideon LAE 57470 (K, L).

#### 4. Dissochaeta annulata

Taxon classificationPlantaeMyrtalesMelastomataceae

Hook .f. ex Triana, Trans. Linn. Soc. London 28: 83, tab. 7, fig. 89a. 1872.

Fig. 5 , Map 3

Diplectria annulata (Hook.*f.* ex Triana) Kuntze, Revis. Gen. Pl. 1: 246. 1891.
Dissochaeta robinsonii Merr., Philipp. J. Sci., C 11: 298. 1916. Type: Indonesia. Moluccas: Ambon, Hitoemesen, 100 m elev., 5 Nov 1913, C.B. Robinson 2024 (lectotype, designated here: BO [BO1747982]!; isolectotypes: BM [BM000944486]!, GH [GH00072242, image seen]!, K [K000859510]!, L [L0537257]!, NY [NY00228565, image seen]!, P [P02274818]!, US [US00120532, image seen]!).
Dissochaeta johannis-winkleri O.Schwartz, Mitt. Inst. Allg. Bot. Hamburg 7: 251. 1931. Type: Indonesia. West Kalimantan: Lebang Hara 160 m elev., 5 Dec 1924, J. Winkler 590 (lectotype, designated here: HBG [HBG522821, image seen]!; isolectotypes: BO [BO1865970]!, HBG [HBG522822, image seen]!).
Dissochaeta deusta Ohwi, Bot. Mag. (Tokyo) 57: 6. 1943. Type: Indonesia. West Papua: Nabire, Dallman, 400 m elev., 1 Mar 1940, R. Kanehira & S. Hatusima 11999 (lectotype, designated here: FU, *n.v.*; isolectotypes: BO [BO1747983]!, L [L0537258]!).
Dissochaeta simalurensis Bakh.*f.*, Contr. Melastom.: 228. 1943. Type: Indonesia. Aceh: Simaloer Eiland, 24 Jun 1919, Achmad 1197 (holotype: L [L0537260]!; isotype: BO!).
Dissochaeta annulata Hook.*f.* ex Triana var. robinsonii (Merr.) Bakh.*f.*, Contr. Melastom.: 231. 1943.
Dissochaeta annulata Hook.*f. ex* Triana var. setosa Bakh.*f.*, Contr. Melastom.: 231. 1943. Type: Indonesia. West Sumatra: Mentawai Eilanden, Siberoet Eiland, 8 Sep 1924, Iboet 12 (holotype: L [L0537259]!; isotypes: BO [BO1865981, BO1865982]!).
Dissochaeta ramosii auct. non Merr.: Furtado, Gard. Bull. Singapore 20: 112, 1963. *p.p.*, excl. type.
Dissochaeta annulata Hook.*f.* ex Triana var. johannis-winkleri (O.Schwartz) J.F.Maxwell, Gard. Bull. Singapore 33: 313. 1980.

##### Type.

Malaysia. Penang: Penang Hill, W. Griffith KD 2268 (lectotype, designated here: K [K000859545]!; isolectotype: K [K000859544]!).

**Figure 5.**
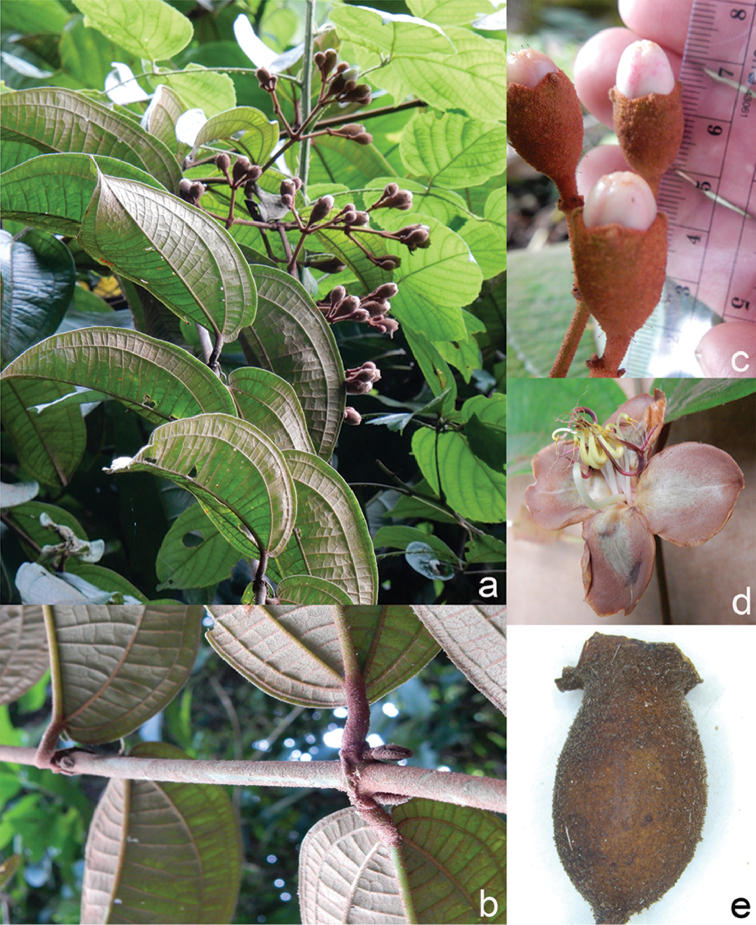
*Dissochaetaannulata***a** habit **b** branchlet **c** hypanthium **d** flower **e** fruit. Photographs by D. Penneys, vouchers: Penneys 2506 (WNC).

**Map 3.**
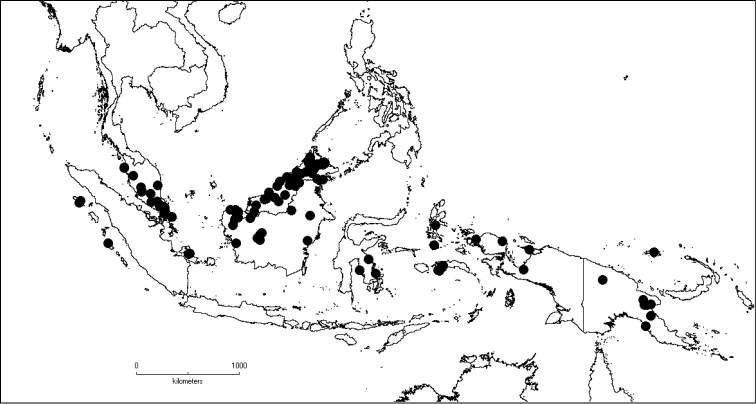
Distribution of *D.annulata* (●).

##### Description.

Climbing up to 30 m in height. Branchlets terete, 3–6 mm in diameter, densely to sparsely brown stellate-furfuraceous to glabrescent, often with dense or scattered bristles; nodes swollen with interpetiolar ridges, internodes 5.5–8 cm long. Leaves: petioles terete, 7–28 mm long, densely stellate-tomentose; blades ovate, 6–15 × 3.5–8 cm, subcoriaceous to coriaceous, rarely membranous, base broadly cordate, rarely rounded, margin entire, apex acuminate, tip 1–1.5 cm long; nervation with 1 or 2 pairs of lateral nerves and 1 pair of intramarginal nerves; adaxially glabrous, bright green with prominent nervation, abaxial greyish-brown, stellate-punctate to tomentose. Inflorescences terminal or in the upper leaf axils, up to 25 cm long, many-flowered; main axis terete to subangular, brown stellate-furfuraceous; primary axis 16–20 cm long with 4–5 nodes, secondary axis 2.5–3 cm long with 1–2 nodes, tertiary axis 0.8–1 cm long with 1 node; bracts lanceolate to oblong, rarely linear, 1–3 × 0.5–1 cm, densely brown stellate-tomentose, caducous; bracteoles lanceolate to oblong, rarely linear, ca. 10 × 5 mm, densely tomentose; pedicels densely tomentose, 3–5 mm long in central flowers, 1–3 mm long in lateral flowers. Hypanthium campanulate, (6–)10–15 × (3–)7–8 mm, densely stellate-tomentose, sometimes with few scattered gland-tipped bristles up to 1 mm long; calyx lobes truncate with rounded, triangular or acute tips, erect, 1–3 mm long; petal buds conical, 7–8 mm long, bright pink; mature petals ovate to obovate, 12–20 × 10–14 mm, reflexed, base clawed, apex rounded, glabrous with ciliate margin, white or white pinkish. Stamens 8, unequal, filaments curved sideways, light yellow; alternipetalous stamens with (10–)12–14 mm long filaments, anthers slender, sickle-shaped, curved, thecae (12–)16–18 mm long, maroon, pedoconnective 4–7 mm long, basal crest entire, erose or bifid, up to 2 mm long, lateral appendages paired, filiform, 6–8 mm long, yellow; oppositipetalous stamens with (8–)10–12 mm long filaments, anthers S-shaped, thecae (10–)12–14 mm long, light yellow, basal crest ligular, obtuse or erose, 1–4 mm long, lateral appendages paired, filiform, 10–12 mm long, yellow. Ovary half as long as hypanthium, apex villous; style (12–)18–22 mm long, curved sideways in direction opposite to the filaments, curved at the tip, glabrous, white; stigma minute; extra-ovarial chambers 8, extending to near the base of the ovary. Fruits ovoid to urceolate, sometimes subglobose, (10–)13–15 × 5–10 mm, densely stellate tomentose, green brownish; calyx lobes persistent. Seeds ca.0.75 mm long.

##### Distribution.

Malay Peninsula, Sumatra (Simeuleu, Bangka, Mentawai & Riau Archipelago), Borneo, Sulawesi (South-East), Moluccas and New Guinea.

##### Ecology and habitat.

Lower montane, evergreen forest on granite, old secondary forest, Kerangas forest, open places at 50–1550 m elevation.

##### Vernacular names.

Peninsular Malaysia: *akar sendudok* (Malay). Sumatra: *olor sigepu bala* (Simeuleu). Borneo: *akar kemunting* (Iban); *gelagan akar* (Dayak); *kelawit* (Ti); *ulur-ulur bukit* (Brunei); *apeh talah* (Apokayan).

##### Notes.

1. One of the most widespread species in the Malesian Region. Surprisingly never found in mainland Sumatra, only on Simeuleu Island, Mentawai Islands, Riau Archipelago and Bangka Island. The species also does not occur in the Philippines and the southern part of Malesia (Java to the Lesser Sunda Islands); in Sulawesi, it was only found in the South-eastern Peninsula.

2. The appearance of *D.annulata* resembles that of *D.axillaris* and *D.bracteata* in the shape of the leaf blades and the stamens. It differs from *D.axillaris* by its terminal inflorescence (instead of axillary) and slightly triangular calyx lobes (instead of truncate). It is distinct from *D.bracteata* by its densely brown stellate-tomentose indumentum in most parts and the campanulate hypanthium, while *D.bracteata* is mostly glabrous with a more tubular hypanthium.

3. The establishment of varieties was initiated by Bakhuizen van den Brink *f.* (1943) and Maxwell (1980b) and it was mostly based on inconstant characters. The variety *robinsonii* was based on the rather small size of the inflorescences, including the size of hypanthium and bracts compared to typical *D.annulata*; we consider size too variable to support the separation of taxa, especially when all the other characters are similar, e.g. indument, hypanthium shape, stamen shape. Moreover, other varieties, *johannis-winkleri* and *setosa*, are only based on a simple character such as a more setose indumentum with additional bristles on the part of flowers and fruits. Since the variation in these characters is continuous, it is better to merge these varieties into the synonymy of *D.annulata*.

##### Selected specimens examined.

**MALAYSIA. Johor**: Gunung Blumut, 900 m, 24 Mar 1923, R.E. Holttum SFN 10657 (BM, BO, K); Gunung Ledang, 900 m, 17 Jul 1969, T.C. Whitmore FRI 12371 (K, L); Kota Tinggi, 50 m, 22 Apr 1978, J.F. Maxwell 78-207 (L); Labis forest Reserve, Endau River, 24 Jul 1977, J.F. Maxwell 77-359 (L). **Pahang**: Fraser’s Hill, 4 Aug 1967, J.C. Carrick 1613 (K, L); Kuantan, Panching, 130 m, 8 Jun 1968, H. Ogata 10513 (L); Tasek Bera, 16 Oct 1930, M.R. Henderson SFN 24057 (K). **Penang**: Penang Hill, W. Griffith KD 2268 (K); Government Hill, A.C. Maingay KD 788 (K, L). **Perak**: B. Scortechini 235 (L, P). **Selangor**: Genting Highlands, Gunong Ulu Kali, 1500 m, 9 Apr 1978, J.F. Maxwell 78-81a (L); Ulu Gombak, 26 May 1966, J.C. Carrick 1474 (K, L). **Terengganu**: Jerteh, Ulu Besut, Bukit Tangga, 900 m, 19 Jul 1984, M. Shah & Mahmud 4944 (L). **Sabah**: Keningau, Nabawan, 21 Aug 1976, Dewol SAN 83879 (K, L); Lamag, Gunong Lotong, Inarat, 400 m, 22 May 1976, P.F. Cockburn SAN 83349 (K, L); Ranau, Lohan to Mamut Copper Mine, 1000 m, 9 Jul 1984, J.H. Beaman et al. 10647 (K, L); *Ibid.*, Mount Kinabalu, Eastern Shoulder, 1066 m, 14 Jun 1961, W.L. Chew, E.J.H. Corner & A. Stainton RSNB 69 (BO, K, L); Tenom, Kemabong-Katubu, 29 Apr 1972, P.F. Cockburn & Saikeh SAN 70032 (K, L); Sandakan, Sep–Dec 1920, M. Ramos BS 1207 (BO, PNH); Tawau, Tawau Hill, 300 m, 15 Jul 1974, Aban & George SAN 79761 (K, L). **Sarawak**: O. Beccari PB 3282 (K, P); Kuching, Matang, 760 m, G.D. Haviland 546 (K); Baram, Kelabit Highland 1066 m, 6 Nov 1974, P.K. Chai S.35320 (K, L); Bintulu, Nyabau, 90 m, 15 Jun 1966, Sibat ak Luang S.24556 (BO, K, L); Marudi, Tinjar, Ulu Dapoi, 14 Apr 1965, E. Wright S.23062 (K, L); Balingian, Ulu Sg. Arip, Bukit Iju, 24 Jul 1965, Sibat ak Luang S.23617 (BO, K, L); Niah, Gunung Subis, Jan 1961, Mohidin S.21636 (K, L); Betong, Bukit Sadok, 15 Oct 1982. Banyeng & Ilias Paie S.45094 (K, L). **SINGAPORE.** Bukit Timah, 1893, H.N. Ridley 5087 (BM). **BRUNEI. Temburong**: Bukit Belalong, 21 Jul 1989, K.M. Wong 1434 (K). **INDONESIA. Aceh**: Simeuleu Island, 24 Jun 1919, Achmad 1197 (BO, L). **Bangka Belitung**: Bangka, Lobok Besar, G. Mangkol, 50 m, 12 Sep 1949, A.J.G.H. Kostermans & Anta 632 (BO, K, L). **Riau Archipelago**: Bintan Island, Gunung Bintan, 350 m, 13 Jun 1919, H.A.B. Bünnemeijer 6158 (BO). **West Sumatra**: Mentawai Islands, Siberut Island, 8 Sep 1924, Iboet 12 (BO, L). **Central Kalimantan**: Katingan-Seruyan Logging Area, 212 m, 27 Jul 2011, R. Susanti et al. 276 (BO). **East Kalimantan**: G. Beratus, 700 m, 18 Jul 1952, A.J.G.H. Kostermans 7595 (BO, K, L); Long Sungai Barang, 750 m, 6 May 1993, J. Van Valkenburg 1252 (BO, L, P); Muan Region, Sungai Riko, 20 m, Dec 1950, A.J.G.H. Kostermans 4386 (BO, L). **North Kalimantan**: Long Bawan, Krayan, 1150 m, 17 Jul 1981, M. Kato, H. Okamoto & E.B. Walujo B-9017 (BO, L). **West Kalimantan**: Pontianak, Bentiang, Gunung Mayung, 800 m, 28 Oct 1980, G. Shea 26643 (BO, L); Sintang, Bukit Baka National Park, 310 m, 9 Nov 1993, A.C. Church et al. 637 (L); Lebang Hara, 160 m, 5 Dec 1924, J. Winkler 590 (BO , HBG); Ketapang, Gunung Palung National Park, Cabang Panti, 930 m, 20 Oct 1997, T.G. Laman et al. 1357 (BO, L). **South East Sulawesi**: North Kolaka, Mt. Mekongga, 931 m, 30 Jun 2011, E.A. Widjaja et al. 9718 (BO). **Moluccas**: Ambon, Hitumesen, 100 m, 5 Nov 1913, C.B. Robinson 2024 (BM, BO, GH, K, L, NY, P, US); Waai, Bukit Pompule, 450 m, 28 Dec 1984, Ramlanto 461 (BO, L); Ceram, Honitetu-Wae Tuba, 4 Feb 1938, P.J. Eyma 2770 (BO); Obi, Anggai, Gunung Batu Putih, 300 m, 20 Nov 1974, E.F. de Vogel 4181 (BO, L). **North Moluccas**: Halmahera, Weda Bay, Tolu Blewen Camp, 475 m, 3 Feb 2013, Gushilman, Haris & Lasut 382 (BO, L). **Papua**: Yapen Island, Nyora Uta, 750 m, 14 Aug 1997, E.A. Widjaja, T. Partomihardjo & A. Ruskandi 6894 (BO, K, L). **West Papua**: Nabire, Dallman, 400 m, 1 Mar 1940, R. Kanehira & S. Hatusima 11999 (BO, L); Sorong, Remu River, 8 May 1954, P. van Royen 4081 (L). **PAPUA NEW GUINEA. Central Province**: Bereina, between Kubuna and Bakoiudu, 14 Jan 1981, N.A. Vinas & Nagari UPNG 4849 (L). **East Sepik**: Hunstein Range 440 m, 18 Jul 1990, W. Takeuchi 6192 (BO, K, L). **Manus**: Lorengau, Buyang, 530 m, 7 Mar 1981, J. Kerenga & J.R. Croft LAE 77282 (K, L). **Morobe**: Buko Creek, Gurakor, 487 m, 11 Jan 1962, A.N. Millar NGF 14452 (K, L); Wampit River, Bupu River, 762 m, 3 Mar 1964, A.N. Millar NGF 23241 (BO, K, L); Lae, 200 m, 21 Nov 1973, M. Jacobs 9676 (BO, L).

#### 5. Dissochaeta atrobrunnea

Taxon classificationPlantaeMyrtalesMelastomataceae

G.Kadereit, Edinburgh J. Bot. 63(1): 4, fig. 1. 2006.

Map 5

##### Type.

Indonesia. Central Kalimantan: Barito Ulu, Project Barito Ulu Base Camp, 1 Jun 1990, K. Sidiyasa PBU 229 (holotype: E [E00225106]!; isotypes: BO [BO0009659]!, K [K001089634]!, L [L.2542233]!).

##### Description.

Climbing up to 20 m in height. Branchlets terete, 4–6 mm in diameter, densely covered with stellate hairs and dark red-brown bristle hairs; nodes swollen, with interpetiolar ridge, thickly covered with stellate hairs and dark-red bristle hairs thickened at base; internodes 3.5–6 cm long. Leaves: petioles terete, 5–9 mm long, densely covered with stellate hairs and bristles; blades ovate, 8–11 × 4–7 cm, subcoriaceous, base cordate, margin entire, apex acuminate, densely bristly, tip 0.5–1 cm long; nervation with 2 pairs of lateral nerves and 1 pair of intramarginal nerves; adaxially glabrous with prominent nerves, abaxially with sparsely brown stellate hairs, more dense on midrib and with bristle hairs. Inflorescences terminal (many-flowered) and axillary (9–15 flowers), up to 20 cm long, up to 7 cm long when axillary; main axis angular, flattened at upper side, densely covered with stellate hairs and bristles; when terminal with primary axes up to 16 cm long with 6 or 7 nodes, secondary axes 5–6 cm long with 2 or 3 nodes, tertiary axes 0.8–1 cm long with 1 node; when axillary with primary axes, up to 5 cm long with 2 or 3 nodes, secondary axes up to 1 cm long with 1 node, tertiary axes not developed; bracts elliptic, 10–15 × 3–5 mm, densely covered with bristle hairs; bracteoles subulate, 7–9 × 1–2 mm, densely covered with bristle hairs; pedicels densely covered with stellate hairs and bristles, 2–3 mm long in central flowers, ca. 1 mm long or subsessile in lateral flowers. Hypanthium campanulate, 6–8 × 3–4 mm, densely covered with stellate hairs and bristle hairs; calyx lobes triangular, 1–2 mm long, densely covered with bristle hairs; petal bud conical, 5–6 mm long, apex bristly; mature petals ovate, 10–12 × 5–6 mm, glabrous, reflexed, base clawed, apex obtuse, white with purple flush or pinkish. Stamens 8, subequal, filaments curved sideways; alternipetalous stamens with ca. 9 mm long filaments, anthers slightly curved, sickle-shaped, thecae 8–9 mm long, pedoconnective 3–4 mm long, basal crest minute, lateral appendages paired, filiform, up to 6 mm long; oppositipetalous stamens with ca. 7 mm long filaments, anthers S-shaped, thecae 8–9 mm long, basal crest absent, lateral appendages paired, filiform, 5–6 mm long. Ovary ⅔ of hypanthium in length, apex thickened, bristly; style slightly curved when mature, 7–8 mm long; stigma minute; extra-ovarial chambers 8, the 4 alternipetalous ones extending to the base of the ovary, the 4 oppositipetalous ones extending to about the lower third of the ovary. Fruits urceolate, ca. 12 × 5–6 mm, covered with stellate hairs and dark red-brown bristles; calyx lobe remnants persistent. Seeds ca. 0.5 mm long.

##### Distribution.

Borneo (Central Kalimantan).

##### Ecology and habitat.

Primary and secondary lowland dipterocarp forest on swampy soil and in open areas at ca. 150 m elevation.

##### Note.

*Dissochaetaatrobrunnea* is known only from three collections from lowland dipterocarp forest in Central Kalimantan, Indonesia. The species resembles *D.alstonii* from North Sumatra by its dense bristles found all over the branchlets, petioles and hypanthium, but differs by having longer bristles and a larger hypanthium and fruits. The appearance of the vegetative organs sometimes resembles *Macroleneshirsuta* (Cogn.) J.F.Maxwell, which is different in its flowering organs (Kadereit 2006).

##### Specimens examined.

**INDONESIA. Central Kalimantan**: Barito Ulu, 1 Jun 1990, K. Sidiyasa PBU 229 (BO, E, K, L); *Ibid.*, Trail Jalang Babang, 18 Jun 1990, C.E. Ridsdale PBU 81 (L); Kahayan River, South of Tumbang Sian, 150 m, 1 May 1988, J.S. Burley & Tukirin 852 (BO, K, L).

#### 6. Dissochaeta axillaris

Taxon classificationPlantaeMyrtalesMelastomataceae

Cogn. in H.J.P.Winkl., Bot. Jahrb. Syst. 48: 108. 1913.

Map 4

Dissochaeta ramosii Merr., J. Straits Branch Roy. Asiat. Soc. 86: 340. 1922. Type: Malaysia. Borneo, Sabah, Sebuga near Sandakan, Dec 1920, M. Ramos BS 1758 (lectotype, designated here: PNH [PNH32282, image seen]!; isolectotypes: A [A00072206, image seen]!, K [K000859509]!, US [US00120531, image seen]!).
Dissocaheta acmura *auct. non* Stapf & M.L.Green: Bakh.*f.*, Contr. Melastom.: 227. 1943. *p.p.*, excl. type.

##### Type.

Indonesia. Central Kalimantan: Semurung, Sungei Tarik, 18 Jul 1908, H.J.P. Winkler 3033 (lectotype, designated here: L [L0652534]!; isolectotypes: BM [BM00094485]!, BO!, BR [BR-518825, image seen]!, K [K000859508]!, WRSL *n.v.*).

**Map 4.**
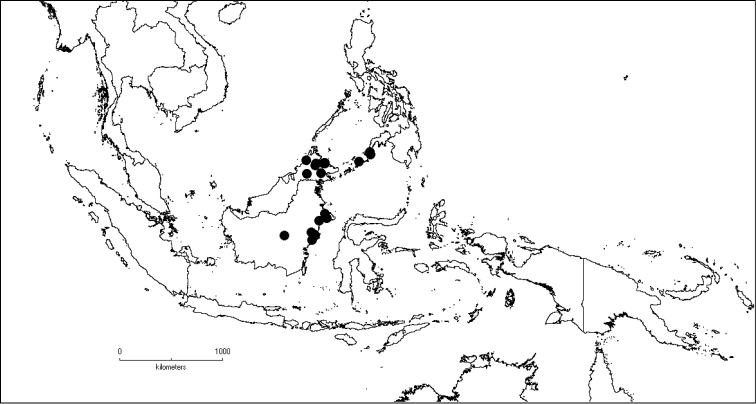
Distribution of *D.axillaris* (●).

##### Description.

Climbing up to 15 m in height. Branchlets terete, 4–5 mm in diameter, covered with brown stellate-tomentose hairs; nodes swollen, with interpetiolar ridges; internodes 6–9 cm long. Leaves: petioles terete, 9–18 mm long, densely stellate-tomentose; blades ovate or ovate-elliptic, 9–20 × 4–8.7 cm, subcoriaceous, base rounded, margin entire, apex acuminate, tip up to 1 cm long; nervation with 1 pair of lateral nerves and 1 pair of intramarginal nerves; adaxially glabrous, abaxially densely stellate-tomentose. Inflorescences axillary, up to 9 cm long, 15–20 flowers; main axes densely stellate-tomentose; primary axes 4–11 cm long with 2 or 3 nodes, secondary axes 1–4.5 cm long with 1 or 2 nodes, tertiary axes ca. 1.5 cm long with 1 node or not developed; bracts linear, ca. 5 mm long, stellate-tomentose, caducous; bracteoles linear, 3–4 mm long, stellate-tomentose, caducous; pedicels densely stellate-tomentose, 4–5 mm long in central flowers, 1–2 mm long in lateral flowers. Hypanthium campanulate, 8–10 mm × ca. 6 mm, densely covered with stellate-tomentose hairs, sometimes with capitate bristles; calyx lobes truncate, without distinct tip, ca. 1.5 mm long, sometimes with 4 minute acute tips; petal bud conical, 5–10 mm long mature petals ovate to suborbicular, 15–20 × 10–20 mm, base clawed, apex rounded, glabrous, white or pinkish white. Stamens 8, unequal, filaments curved sideways; alternipetalous stamens with 10–11 mm long filaments, anthers curved, sickle-shaped, thecae ca. 10 mm long, apex rostrate, pedoconnective ca. 5 mm long, basal crest erose, ca. 1 mm long, lateral appendages paired, filiform, 5–10 mm long; oppositipetalous stamens with ca. 10 mm long filaments, anthers S-shaped, thecae 10–12 mm long, basal crest ligular, ca. 0.5 mm long, sometimes with a pair of capillary appendages, ca. 1 mm long, lateral appendages paired, filiform, 7–8 mm long. Ovary ¾ of hypanthium in length, apex villous; style glabrous, curved at tip, 10–12 mm long; stigma minute; extra-ovarial chambers 8, extending almost to the base of the ovary. Fruits urceolate, ca. 15 × 12 mm, sparsely covered with stellate hairs or glabrous; calyx remnant truncate, persistent. Seeds ca. 0.75 mm long.

##### Distribution.

Borneo and Philippines (South-western Islands).

##### Ecology and habitat.

Primary open lowland forest or on limestone at 10–930 m elevation.

##### Vernacular name.

Borneo: *rinsim* (Kinabatangan).

##### Note.

*Dissochaetaaxillaris* is easy to distinguish from all other species by its tomentose indumentum and axillary inflorescences. Differences with *D.acmura*, another species with axillary inflorescences species, are the more subcoriaceous leaf blades and the small petal buds which are sunken inside the calyx lobes. The distribution of those two species does not overlap. Sometimes this species is misidentified as *D.annulata*, which has leaf blades with a subcordate base and terminal inflorescences.

##### Selected specimens examined.

**MALAYSIA. Sabah**: Beluran, Bidu-Bidu Forest Reserve, 1 Mar 1991, Maikin & Lideh SAN 131046 (L); Bongol, 365 m, G.D. Haviland 1385 (K); Sandakan, Long Manis, 9 Aug 1962, G. Mikil SAN 31570 (BO, K, L); *Ibid.*, Myburgh, Oct-Dec 1921, A.D.E. Elmer 20106 (BM, BO, K, L, P, U); *Ibid.*, Kabili-Sepilok FR., 26 Jun 1937, Enggoh BNB 7270 (K, L); *Ibid*., Sebuga, M. Ramos BS 1758 (K, PNH); Beluran, Bongaya FR, 45 m, 17 Jul 1975, Aban & Kodoh SAN 81978 (K, L); Lahad Datu, Danum Valley, 22 Jul 1986, Leopold et al. SAN 114565 (K, L). **INDONESIA. Central Kalimantan**: Semurung, Sungai Tarik, 18 Jul 1908, H.J.P. Winkler 3033 (BM, BO, BR, K, L). **East Kalimantan**: Samarinda, Loa Haur, 60 m, 12 May 1952, A.J.G.H. Kostermans 6846 (BO, K, L, PNH); East Kutai, Sungai Susuk Region, 26 Jun 1951, A.J.G.H. Kostermans 5452 (BO, K, L); West Bengalon, Sebongkok Utara, 102 m, 6 Apr 1996, Ambriansyah & Arbainsyah AA 1667 (BO, K, L, P); Samboja, 50 m, 6 Jul 1995, Ambriansyah et al. AA 1280 (K, L, P); Sangkulirang, Mangapu, 10 m, 19 Jun 1937, Aet 739 (BO, L); Sebulu, 50 m, 1 Dec 1980, M. Kato & H. Wiriadinata B-6995 (BO, L). **West Kalimantan**: Ketapang, Gunung Palung National Park, Cabang Panti, 930 m, 20 Oct 1997, T.G. Laman et al. 1357 (BO, K). **PHILIPPINES. Basilan**: Nov 1912, D.P. Miranda FB 17872 (BM, K, L, P). **Mindanao**: St. Cruz Island, Sapamoro, 20 Dec 1961, Olsen 989 (L). **Sulu**: Jolo, Mt. Daho, Sep 1924, M. Ramos & G.E. Edano BS 43902 (L, P).

#### 7. Dissochaeta bakhuizenii

Taxon classificationPlantaeMyrtalesMelastomataceae

Veldkamp, Blumea 24: 443. 1979.

Fig. 6 , Map 5

Dissochaeta microplectrosa J.F.Maxwell, Gard. Bull. Singapore 33: 313, fig. 3. 1980. Type: Indonesia. North Sumatra: Karoland, Mount Sinabung, 1400 m elev., 19 Aug 1928, J.A. Lörzing 13673 (holotype: L [L0537283]!; isotype: BO!).
Neodissochaeta reticulata auct. non Bakh.*f.*: Bakh.*f.*, Contr. Melastom.: 143. 1943. *p.p*., excl. type.
Dissochaeta sagittata auct. non Blume: Bakh.*f.*, Contr. Melastom.: 233. 1943. *p.p*., excl. type.

##### Type.

Indonesia. West Sumatra: Ophir District, Tanang Taloe, 1100 m elev., 15 Jun 1917, H.A.B. Bünnemeijer 1053 (holotype: L [L0537231]!; isotypes: BO [BO1744599, BO1747935]!, PNH *n.v.*).

**Figure 6.**
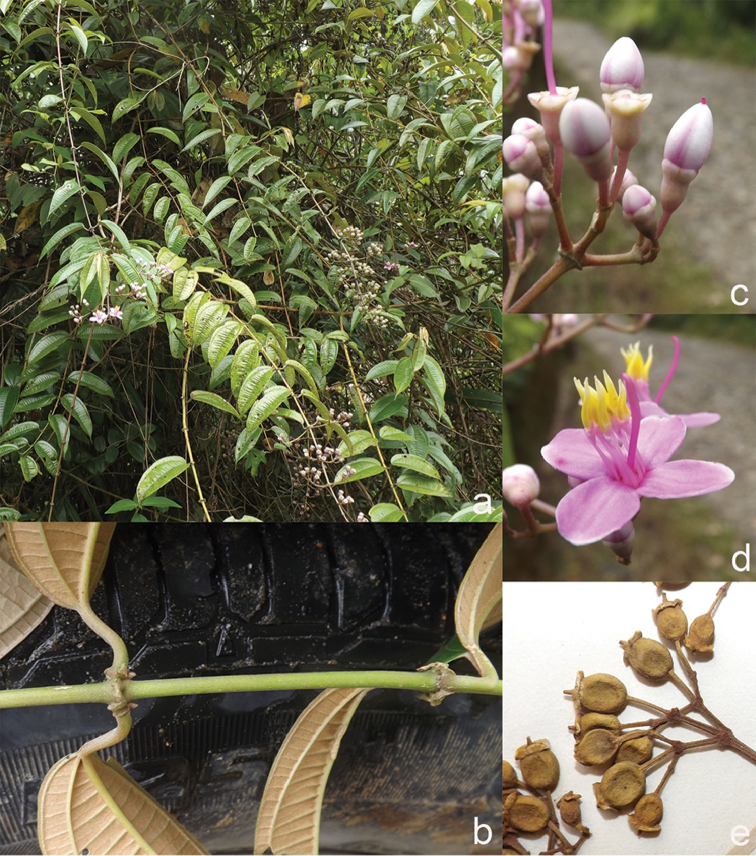
*Dissochaetabakhuizenii*. **a** habit **b** branchlet **c** hypanthium **d** flower **e** fruits. Photographs by A. Kartonegoro; vouchers: Kartonegoro 1103 (BO, L).

**Map 5.**
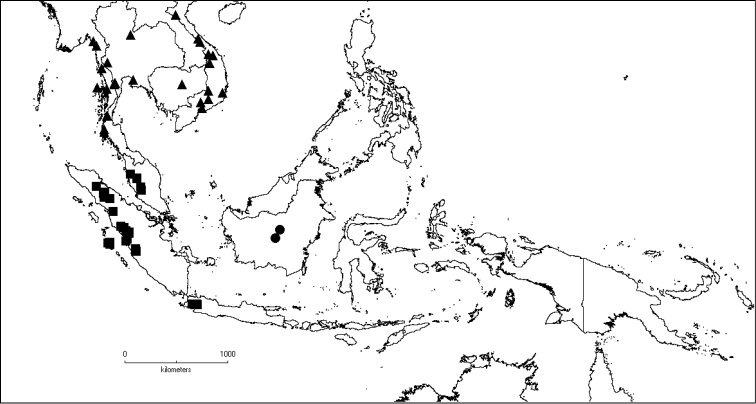
Distribution of *D.atrobrunnea* (●), *D.bakhuizenii* (■) and *D.barbata* (▲).

##### Description.

Climbing up to 30 m in height. Branchlets terete, 3–5 mm in diameter, greyish or brown stellate-puberulous with small bristle enations; nodes swollen, interpetiolar ridge distinct with collar-shaped ridge or crest-like; internodes 5–10 cm long. Leaves: petioles flattened, 10–15 mm long, stellate-furfuraceous; blades elliptic or ovate-elliptic, 7.5–13.5 × 3–5.25 cm, membranous, base rounded, margin entire, apex acuminate, tip ca. 0.5–1 cm long; nervation with 1 pair of lateral nerves and 1 pair of intramarginal nerves; adaxially glabrous, glossy green, abaxially densely, brown, short stellate-puberulous. Inflorescences terminal, up to 25 cm long, many-flowered; main axis glabrous to sparsely stellate-puberulous, rarely bristly; primary axes up to 14 cm long with 4 or 5 nodes, secondary axes up to 5 cm long with 2 or 3 nodes, tertiary axes up to 2 cm long with 1 or 2 nodes; bracts and bracteoles minute, inconspicuous, caducous; pedicels sparsely stellate-furfuraceous, 3–4 mm long in central flowers, 1–2 mm long in lateral flowers. Hypanthium campanulate-tubular, 2–5 × 1–3 mm, sparsely stellate-puberulous or nearly glabrous, somewhat 8-ridged; calyx truncate with 4 undulate lobes, widened, ca. 1 mm long, glabrous; petal bud conical, 3–5 × 2–3 mm; mature petals ovate, 4–5 × ca. 4 mm, base clawed, apex rounded, glabrous with ciliate margin, pale pink to violet. Stamens 8, equal or subequal, filaments straight; alternipetalous stamens with 4–6 mm long filaments, anthers oblong or lanceolate, straight, thecae 4–5 mm long, yellow, pedoconnective ca. 0.5 mm long, basal crests triangular with a small pair of acute auricles, 1.5–2 mm long, lateral appendages absent or prolonged from basal crest, 1–2 mm long; oppositipetalous stamens with 2.5–3 mm long filaments; anther oblong-lanceolate, straight, thecae 3–4 mm long, yellow, basal crest ligular, 1–1.5 mm long, lateral appendages a minute pair of auricles or absent. Ovary half as long as hypanthium, apex puberulous; style glabrous, curved at top, 10–12 mm long; stigma minute; extra-ovarial chambers 8, extending to the middle of the ovary. Fruits ovoid or subglobose, 4–5 × 3–4 mm, glabrous, often with 8 lines, apex mammiform; calyx lobe remnants persistent. Seeds ca. 0.4 mm long.

##### Distribution.

Peninsular Malaysia, Sumatra and Java (West).

##### Ecology and habitat.

Secondary forest, montane forest or near a crater in open forest, 700–1550 m elevation.

##### Vernacular names.

Sumatra: *sanduduk* (Batak); *pulutu* (Mentawai). Java: *harendong areuy* (Sundanese).

##### Note.

This species can easily be distinguished by the presence of only fertile stamens without any lateral appendages and fruits with a mammiform apex. The mammiform apex on the fruits resembles that of *D.nodosa* from Sumatra and *D.rectandra* from Peninsular Malaysia. In the indumentum of the lower leaf surface, it resembles *D.inappendiculata* Blume and it is sometimes misidentified when vegetative only.

##### Selected specimens examined.

**MALAYSIA. Pahang**: Cameron Highlands, Robinson’s Falls, 1600 m, 16 Apr 1978, J.F. Maxwell 78-197 (L); *Ibid.*, 1400 m, 20 Mar 1992, J. Klackenberg & R. Lundin 673 (L); Fraser’s Hill, 1550 m, 27 Sep 1978, J.F. Maxwell 78-368 (L). **Perak**: Bukit Larut, Dec 1883, King’s collector 5284 (L); *Ibid*., Gunung Hijau, 1320 m, 13 Jul 2006, M.K. Hisham et al. FRI 52047 (BO, L). **Selangor**: Genting Highlands, Gunong Ulu Kali, 1200 m, 9 Apr 1978, J.F. Maxwell 78-83 (L). **INDONESIA. Aceh**: Mt. Leuser, Gunung Bandahara, 800–1000 m, 20 Mar 1975, W.J.J.O. de Wilde & B.E.E. de Wilde-Duyfjes 15596 (BO, K). **Jambi**: Kerinci, Kayu Aro, 850 m, 21 Oct 1954, W. Meijer 3007 (BO); *Ibid.*, Sungai Kumbang, 1400 m, 4 Apr 1914, H.C. Robinson & C. Boden-Kloss s.n. (BM). **Mentawai Islands**: Siberut, 10 Sep 1924, C. Boden-Kloss SFN 12282 (BO, K). **North Sumatra**: Karo, Mount Sinabung, 1400 m, 19 Aug 1928, J.A. Lörzing 13673 (BO, L); *Ibid.*, Road from Siantar to Berastagi, 1000 m, 21 Feb 1932, W.N. Bangham & C.N. Bangham 951 (K); Tapanuli, Between Sidikalang and Pongkolan, 1200 m, 27 Mar 1954, A.H.G. Alston 14790 (BM, BO, PNH); Prapat, Gunung Batu Lopang, 1400 m, 8 Jul 1972, W.J.J.O. de Wilde & B.E.E. de Wilde-Duyfjes 13528 (BO, K); Sibolangit, Bandar Baru, 800 m, 17 Jun 1916, J.A. Lörzing 4349 (BO); Sipirok, Dolok Sibual-Buali, 1200 m, 8 Mar 1983, Zahro 69 (BO). **West Sumatra**: Ophir, Tanang Talu, 1100 m, 15 Jun 1917, H.A.B. Bünnemeijer 1053 (BO, L) Lubuk Sikaping, Mt. Gadang, 700 m, 15 Jun 1953, J. van Borssum-Waalkes 1893 (BO, K); Batu Sangkar, Mount Sago, Puncak Pato, 1200 m, 10 Mar 1989, H. Nagamasu 3782 (ANDA). **Banten**: Between Citorek & Muncang, 800 m, 22 Jun 1911, C.A. Backer 1839 (BO). **West Java**: Mt. Salak, Gunung Bunder to Kawah Ratu, 1300 m, 8 Jan 1941, C.N.A. de Voogd & S. Bloembergen s.n. (BO, L); *Ibid.*, Cangkuang, 1000 m, 16 Sep 1985, M.M.J. van Balgooy 5161 (BO, L, P); Mt. Halimun, Malasari, 1055 m, 10 Oct 2017, A. Kartonegoro 1103 (BO, L); Mt. Sembung, C.A. Backer 12256 (BO).

#### 8. Dissochaeta barbata

Taxon classificationPlantaeMyrtalesMelastomataceae

(Triana ex C.B.Clarke) Karton. comb. nov.

urn:lsid:ipni.org:names:60476833-2

Map 5

Anplectrum stellulatum Geddes, Bull. Misc. Inform. Kew: 236. 1928. Type: Thailand. Phitsanulok: Nakhon Thai, 200 m elev., 17 Apr 1922, A.F.G. Kerr 5870 (lectotype, designated here: K [K000859555]!; isolectotypes: BK [BK-257160, image seen]!, BM!).
Anplectrum cyanocarpa auct. non. Triana: Kurz, Forest Fl. Burma 1: 508. 1877. *p.p.* excl. type.
Anplectrum glaucum auct. non. Triana: C.B.Clarke in Hook.*f.*, Fl. Brit. India 2: 545. 1879. *p.p.*, excl. type.
Backeria barbata (Triana ex C.B.Clarke) Raizada, Indian Forester 94: 435. 1968.
Diplectria barbata (Triana ex C.B.Clarke) Franken & M.C.Roos in Veldkamp *et al*., Blumea 24: 415, fig. 3A. 1979.
Dissochaeta divaricata auct. non. G.Don: Clausing in Renner *et al*., Fl. Thailand 7(3): 423. 2001. *p.p.*, excl. type.

##### Basionym.

*Anplectrumbarbatum* Triana ex C.B.Clarke in Hook.*f.*, Fl. Brit. India 2: 546. 1879.

##### Type.

Myanmar. Martaban: Chappedong, N. Wallich 4082 (holotype: K [K000859568]!).

##### Description.

Climbing up to 10 m in height. Branchlets terete, 3–5 mm in diameter, glabrous or with scattered minute stellate hairs; nodes swollen, with interpetiolar ridge; internodes 4–6.5 cm long. Leaves: petioles terete, 10–15 mm long, glabrous to stellate-puberulous, covered with bristle hairs at dorsal side; blades elliptic-oblong to oblong, 6.5–13 × 2.5–5 cm, chartaceous, base rounded, margin entire, apex acuminate, tip ca. 1 cm long; nervation with 1 pair of lateral nerves and 1 pair of intramarginal nerves; adaxially glabrous, abaxially with sparsely stellate hairs at nerves, with a pair of basal glandular patches. Inflorescences terminal, up to 30 cm long, many-flowered; main axis terete, glabrous or stellate-puberulous; primary axes up to 28 cm long with 8 or 9 nodes, secondary axes up to 10 cm long with 4 or 5 nodes, tertiary axes up to 2 cm long with 1 or 2 nodes, quarternary axes up to 1 cm long with 1 node; bracts linear, 2–4 mm long, stellate puberulous; bracteoles linear, 3–4 mm long, stellate puberulous; pedicels stellate-puberulous, 4–6 mm long in central flowers, 2–4 mm long in lateral flowers. Hypanthium campanulate-angular to suburceolate, 5–6 × 2–3 mm, slightly 4-ridged, glabrous or stellately puberulous; calyx lobes truncate, without 4 distinct tips, ca. 1 mm long; petal bud conical, 4–5 mm long; mature petals ovate-elliptic, ca. 7 × 4–4.5 mm, not reflexed, base clawed, apex acute, pink to purplish. stamens 8, unequal, filaments straight; alternipetalous stamens staminodial, with 3–4 mm long filaments, thecae rudimentary, slender, terete, ca. 4 mm long, pedoconnective undeveloped, basal crest triangular, ca. 2 mm long, acute or erose at tip, thin, lateral appendages in a pair, linear to filiform, ca. 2 mm long; oppositipetalous stamens with 5–6 mm long filaments, anthers thick, curved, S-shaped, thecae 8–9 mm long, yellow, apex rostrate, basal crest triangular, ca. 1 mm long, basal appendages bifid, 1–2 mm long. Ovary ⅔ of hypanthium in length, apex glabrous; style glabrous, 13–15 mm long, curved at the end, slender, pink; stigma minute; extra-ovarial chambers 4, oppositipetalous, extending almost to the base of the ovary. Fruits urceolate, 7–8 × 4–5 mm, glabrous; calyx lobe remnants persistent. Seeds numerous, ca. 0.75 mm long, cuneate.

##### Distribution.

Myanmar, Indo-China, South China (Hainan; fide Chen & Renner 2007) and Thailand.

##### Ecology and habitat.

Mixed deciduous and disturbed evergreen forest to mountainous forest at 90–1500 m elevation.

##### Vernacular name.

*kyar ma naing* (Myanmar), 藤牡丹, *teng mu dan* (China)..

##### Note.

*Dissochaetabarbata* resembles *D.divaricata* in the hypanthium and fertile stamens, with the alternipetalous stamens staminodal and the oppositipetalous ones fertile. *Dissochaetabarbata* differs in having a subglabrous indumentum in most parts and in possessing a pair of glandular patches on the base of leaf blades underneath. This is the only known species that is completely distributed outside the Malesian region.

##### Selected specimens examined.

**MYANMAR. Ban Han**: Bon s.n. (P). **Martaban**: Chappedong, N. Wallich 4082 (K). **Tanintharyi**: Yaphyu, Alel Taung, Kyaukshut Village, 297 m, 24 Mar 2015, K. Armstrong et al. 627 (NY). **Tenasserim**: 31 May 1869, J.W. Helfer 2290 (K), Kallin Kwan Chaung, 60 m, 10 Feb 1926, C.E. Parkinson 1691 (K); Mergui, W. Griffith KD 2289 (K). **CAMBODIA.** Chuo Chan, L. Pierre s.n. (BM, K, L). **VIETNAM. Bien Hoa**: Chiao Pang, Mar 1877, L. Pierre s.n. (P). **Bum Mo**: 18 May 1921, B. Hayata 337 (P). **Haut Donnai**: Blao, 1000 m, 21 Feb 1933, E. Poilane 22026 (L, P). **Khanh Hoa**: Dien Khanh, Hon Ba, 900 m, 24 Jun 2004, D.D. Soejarto & T.N. Ninh 13310 (L, P). **Kontum**: Dakto, Ngok Guga, 1000 m, 27 Nov 1946, E. Poilane 35651 (L, P). **Lang Go Rum**: Cu Bi, 600 m, 28 Jul 1925, E. Poilane 12234 (P). **Nghe An**: Con Cuong, Ye Khe, 16 Oct 2008, Du et al. 3009 (K). **Quang Duc**: Dak Song, 800 m, 19 Mar 1953, E. Schmid s.n. (P). **Quang Nam**: Moi Se Go, 1500 m, 25 Feb 1941, E. Poilane 31700 (P). **Quang Tri**: Lang Vieng Ap, 400 m, 13 Jun 1924, E. Poilane 10854 (L, P). **THAILAND. Chonburi**: Kao Re Chan, 600 m, 21 Apr 1931, H.S.H. Lakshnakara 739 (K). **Kanchanburi**: Thong Paphum, 19 Apr 1967, B. Nimanong 89 (L). **Petchaburi**: Khao Cong, 8 Jan 1929, A.F.G. Kerr 16573 (BM, K, L); Kaeng Krachan, Panoen Thung, 100 m, 27 Jan 2005, K. Williams et al. 1148 (L). **Phang Nga**: Kapong, 100 m, 17 Feb 1929, A.F.G. Kerr 17113 (BM, K); Kao Paw Ta Luang Keo, 29 Apr 1974, K. Larsen & S.S. Larsen 33470 (K, L, P). **Phitsanulok**: Nakhon Thai, 200 m, 17 Apr 1922, A.F.G. Kerr 5870 (BK, BM, K). **Ranong**: Khlong Kam Puan, 100 m, 1 May 1973, R. Geesink & T. Santisuk 5098 (L); Muang, 400 m, 25 Apr 2005, R. Pooma et al. 5259 (L).

#### 9. Dissochaeta beccariana

Taxon classificationPlantaeMyrtalesMelastomataceae

Cogn. in A.DC. & C.DC., Monogr. Phan. 7: 559. 1891.

Map 8

Neodissochaeta magnibracteata Bakh.*f.*, Contr. Melastom.: 142. 1943. Type: Indonesia. West Kalimantan: Soengei Kenepai, 1893, J.G. Hallier 2013 (holotype: L [L0537263]!; isotypes: BO [BO1865993, BO1865994, BO1865995]!, K [K000859505]!, SING *n.v.*).
Neodissochaeta beccariana (Cogn.) M.P.Nayar, Kew Bull. 20: 159. 1966.

##### Type.

Malaysia. Sarawak: Santubong, O. Beccari PB 2190 (lectotype, designated here: FI [FI007928, image seen]!; isolectotype: K [K000859504]!).

##### Description.

Climbing up to 30 m in height. Branchlets terete, 3–5 mm in diameter, glabrous, sometimes with scattered bristles; nodes swollen, with a raised interpetiolar, crest-like ridge, sparsely covered with stellate hairs; internodes 8–9 cm long. Leaves: petioles terete, 1.3–1.5 cm long, glabrous except small bristles abaxiallyly; blades broadly ovate, 11–16 × 6.2–7.5 cm, subcoriaceous, base rounded, margin entire, apex acuminate, tip up to 2 cm long; nervation with 1 pair of lateral nerves and 1 pair of intramarginal nerves; adaxially glabrous, abaxially glabrous with sparsely stellate hairs on the midrib, with a basal pair of glandular patches. Inflorescences terminal, up to 22 cm long, many-flowered; main axis glabrous to sparsely stellate-puberulous; primary axes up to 18 cm long with 4 or 5 nodes, secondary axes up to 4 cm long with 1 or 2 nodes, tertiary axes ca. 1 cm with 1 node; bracts elliptic, ca. 3 × 1.2 cm, conspicuous, glabrous, white; bracteoles ovate, conspicuous, 6–8 × 6–7 mm, glabrous, white; pedicels glabrous or sparsely stellate-puberulous, 5–6 mm long in central flowers, 2–3 mm long in lateral flowers. Hypanthium tubular, 4–7 × 2–3 mm, glabrous or stellately-puberulous; calyx lobes truncate, with or without 4 small undulate tips, ca. 0.5 mm long; petal bud conical, up to 4 mm long, apex with narrow acute tip; mature petals broadly ovate, 6–7 × 5–6 mm, reflexed, purple, base clawed to slightly cordate, apex acute. Stamens 8, subequal, filaments straight, light yellow; alternipetalous stamens with 4–5 mm long filaments, anthers curved, sickle-shaped, thecae 4–5 mm long, apex acute, purple, pedoconnective ca. 1 mm long, basal crest triangular, thin, erose, irregular margin 1–2 mm long, lateral appendages paired, filiform, 3–5 mm long; oppositipetalous stamens with 5–6 mm long filaments, bent at the attachment to anthers, anthers thick, slightly curved, hook-shaped, thecae 5–7 mm long, yellow, apex obtuse, basal crest thin, ligular, 1–2 m long, lateral appendages paired, filiform, 1–2 mm long. Ovary half as long as hypanthium, apex glabrous; style glabrous, 16–18 mm long, curved at the end, slender, pale yellow; stigma minute, pinkish; extra-ovarial chambers 8, shallow to nearly undeveloped. Fruits subglobose to ovoid, 5–8 × 3–6 mm, glabrous, with 8 slight lines along the surface; calyx lobe remnants persistent. Seeds ca. 0.75 mm long.

##### Distribution.

Borneo.

##### Ecology and habitat.

Lowland to montane dipterocarp forest in open shade at 30–1400 m elevation.

##### Vernacular name.

*Akar* (Iban).

##### Note.

The species is easily recognised because all parts are usually glabrous, the leaf blades abaxiallyly have a pair of glandular patches at the base and the bracts and bracteoles are white. The most similar species is *D.glandulosa*, also with glabrous parts and glandular patches, but it has a larger, campanulate hypanthium.

##### Selected specimens examined.

**MALAYSIA. Sabah**: Beaufort, Beaufort Rail-Line, 10 m, 22 Mar 1962, G. Mikil SAN 34540 (K, L); Keningau, Pensiangan Kayu, 17 Oct 1985, Sumbing SAN 110362 (L); *Ibid.*, Crocker Range, 1400 m, 17 Oct 1999, S.J. Davies et al. 99128 (L); Tawau, Road Kalabakan, 26 Jul 1962, Aban Gibot SAN 30555 (K, L); Ranau, Mt. Kinabalu, Dallas, 900 m, 5 Aug 1931, J. Clemens & M.S. Clemens 30342 (BM, K, L). **Sarawak**: O. Beccari PB 2190 (FI, K); Sibu, Rejang, Jul 1893, G.D. Haviland 3628 (BM, K); Bario, Bukit Lawi, 1500 m, 2 Aug 1985, D. Awa & B. Lee S.50537 (L); Kuching, Gunung Penrissen, 2 May 1962, Ilias Paie S.16326 (K); *Ibid.*, Semenggoh FR., 19 Oct 1966, Banyeng ak Nyudong S.26207 (BO, K, L); Belaga, Linau Balui, Sg. Nawai, 800 m, 5 Sep 1978, B. Lee S.40000 (K, L); Kapit, Balleh, Menyiong, Sungai Sebatu, 500 m, 12 Nov 1979, Othman bin Haron et al. S.41368 (K, L); *Ibid.*, Wong Kijang, 25 Oct 1988, Othman et al. S.56049 (L); Limbang, Sungai Ensungei, Tg. Long Amok, 11 Sep 1980, R. George et al. S.42845 (K, L); Miri, Gunung Mulu National Park, 1400 m, 9 Mar 1990, P.C. Yii & Abu Talib S.58277 (K, L). **BRUNEI. Belait**: Melilas, Ulu Ingei, 30 m, 5 Mar 1996, Said BRUN 17315 (K). **Temburong**: Ulu Belalong, 500 m, 21 Jan 1994, J. Dransfield et al. 7392 (K, L). **INDONESIA. East Kalimantan**: West Kutai, Long Ibut, 130 m, 16 Aug 1925, F.H. Endert 2552 (BO, L); *Ibid.*, Mount Kemul, 1200 m, 26 Sep 1925, F.H. Endert 3572 (BO, L). **West Kalimantan**: Pontianak, Gunung Bentuang, 200 m, 24 Jun 1989, J.S. Burley & Tukirin 2857 (BO, L); Sungai Kenepai, 1893, J.G. Hallier 2013 (BO, K, L); Sintang, 120 m, 18 Apr 1994, A.C. Church et al. 1000 (BO, L); Kapuas Hulu, Mendalam Rivers, 170 m, 15 Mar 2000, Albertus 45 (L).

#### 10. Dissochaeta biligulata

Taxon classificationPlantaeMyrtalesMelastomataceae

Korth. in Temminck, Verh. Nat. Gesch. Ned. Bezitt., Bot.: 240. 1844.

Fig. 7 , Map 6

Dissochaeta microcarpa Naudin, Ann. Sci. Nat., Bot. sér. 3, 15: 72. 1851. Type: Singapore. Singapore Island, Feb 1837, C. Gaudichaud-Beaupré 80 (holotype: P [P02274814, image seen]!).
Dissochaeta bancana Miq., Fl. Ned. Ind. 1(1): 529. 1855. Type: Indonesia. Bangka, T. Horsfield 19 (lectotype, designated here: K [K000859500]!; isolectotype: K [K000859501]!).
Anplectrum biligulatum (Korth.) Triana, Trans. Linn. Soc. London 28: 85. 1872.
Dissochaeta celebica auct. non Blume: Triana, Trans. Linn. Soc. London 28: 83. 1872; C.B.Clarke in Hook.*f.*, Fl. Brit. India 2: 544. 1879; Cogn. in Boerl., Handl. Fl. Ned. Ind. 2: 533. 1890; in A.DC. & C.DC., Monogr. Phan. 7: 561. 1891; in H.J.P.Winkl., Bot. Jahrb. Syst. 48: 108. 1913; King, J. Asiat. Soc. Bengal, Pt. 2, Nat. Hist. 69(2): 54. 1900; Ridl., Fl. Malay Penins. 1: 798. 1922; Merr., Univ. Calif. Publ. Bot. 15: 224. 1929; Craib, Fl. Siam. Enum. 1, 4: 697. 1931; Bygrave & A.P.Davis, Checkl. Fl. Pl. Gymnosperms Brunei Darussalam: 186. 1996; Cellin. in J.H.Beaman & C.E.Anderson, Pl. Mt. Kinabalu 5: 99. 2004. *p.p.*, excl. type.
Dissochaeta intermedia auct. non Blume: C.B.Clarke in Hook.*f.*, Fl. Brit. India 2: 544. 1879. *p.p.*, excl. type.
Diplectria biligulata (Korth.) Kuntze, Revis. Gen. Pl. 1: 246. 1891.
Dissochaeta celebica Blume var. contracta King, J. Asiat. Soc. Bengal, Pt. 2, Nat. Hist. 69(2): 54. 1900. Type: Malaysia. Perak: Larut, Apr 1882, King’s collector 2911 (lectotype, designated here: K [K000859535]!; isolectotypes: BM!, CAL *n.v.*).
Dissochaeta scortechinii King, J. Asiat. Soc. Bengal, Pt. 2, Nat. Hist. 69(2): 55. 1900. Type: Malaysia. Perak: B. Scortechini 23 (lectotype, designated here: K [K000859536]!; isolectotype: SING *n.v.*).
Neodissochaeta biligulata (Korth.) Bakh.*f.*, Contr. Melastom.: 140. 1943.
Neodissochaeta celebica (Blume) Bakh.*f.*, Contr. Melastom.: 141. 1943. *p.p.*, excl. type.
Dissochaeta monticola auct. non Blume: Clausing in S.S.Renner *et al*., Fl. Thailand 7(3): 428. 2001. *p.p.*, excl. type.
Dissochaeta intermedia Blume var. sagittata auct. non. J.F.Maxwell: Cellin. in J.H.Beaman & C.E. Anderson, Pl. Mt. Kinabalu 5: 99. 2004. *p.p.*, excl. type.

##### Type.

Indonesia. West Sumatra: Gunung Paauw, P.W. Korthals s.n. (lectotype, designated here: L [L0537285]!; isolectotype: L [L0537284]!).

**Figure 7.**
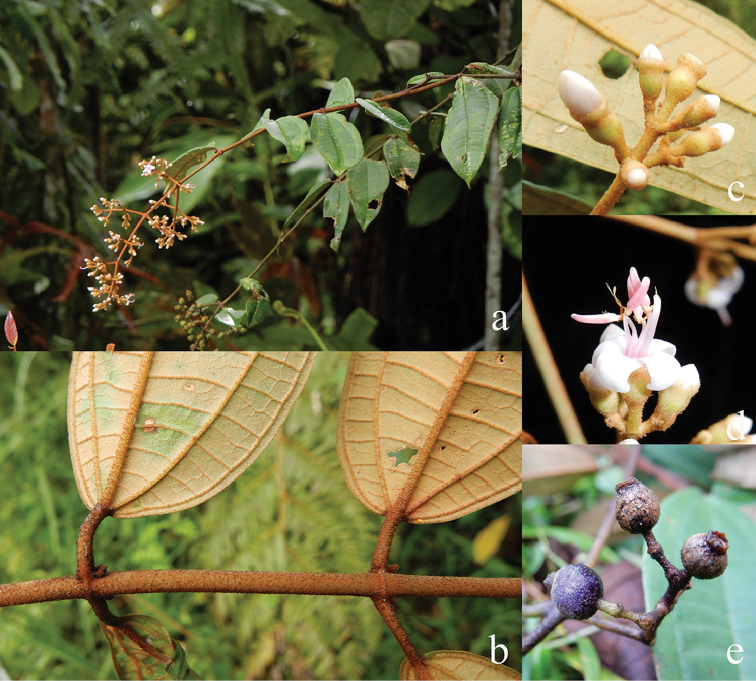
*Dissochaetabiligulata***a** habit **b** branchlet **c** hypanthium **d** flower **e** fruits. Photographs by D. Penneys; vouchers: Penneys 2488 (WNC) & Penneys 2509 (WNC).

**Map 6.**
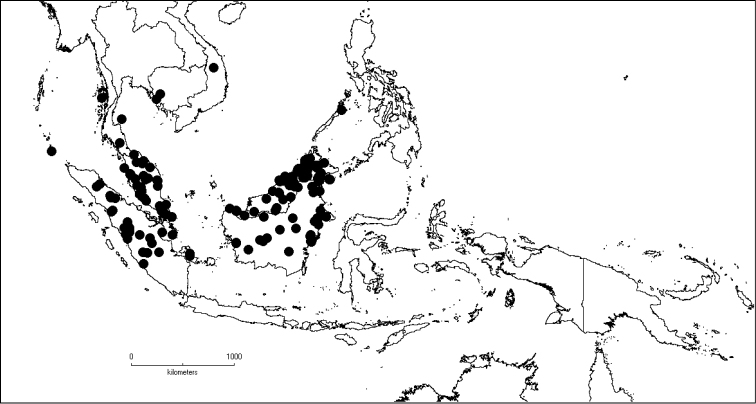
Distribution of *D.biligulata* (●).

##### Description.

Climbing up to 5 m height. Branchlets terete, 3–4 mm in diameter, densely brown stellate-furfuraceous; nodes swollen, with interpetiolar line; internodes 4–6.5 cm long. Leaves: petioles terete, 6–8 mm long, brown stellate-furfuraceous; blades ovate-oblong or elliptic-oblong, 7–15 × 2.5–5.4 cm, membranous, base rounded, margin entire, apex acuminate, tip ca. 1 cm long; nervation with 1 pair of lateral nerves and 1 pair of intramarginal nerves; adaxially glabrous, yellowish-green, abaxially densely covered with minute brown stellate-furfuraceous hairs. Inflorescences terminal, 10–16 cm long, many-flowered; main axis angular, brown stellate-furfuraceous; primary axes up to 15 cm long with 6–8 nodes, secondary axes 2–4 cm long with 2 or 3 nodes, tertiary axes 0.5–1 cm long with 1 node; bracts linear, ca. 3 mm long, brown stellate-furfuraceous; bracteoles linear, 2–2.5 mm long, brown stellate-furfuraceous; pedicel densely stellate-furfuraceous, 1–2 mm long in central flowers, ca. 0.5 mm long or absent in lateral flowers. Hypanthium suburceolate, 3–4 × 2–2.5 mm, brown stellate-furfuraceous; calyx lobes truncate with 4 undulate tips, ca. 1 mm long; petal buds conical, 2–3 mm long, glabrous; mature petals ovate to elliptic, 5–6 × 3–3.5 mm, reflexed, base clawed, apex obtuse, glabrous, white or pinkish-white, sometimes transparent. Stamens 4, equal, alternipetalous, filaments curved sideways, ca. 5 mm long, white, pinkish at base, anthers oblong-lanceolate, thecae 3.5–5 mm long, straight, pink, pedoconnective ca. 1 mm long, basal crest triangular or erose with irregular margin, yellow, ca. 0.5 mm long, lateral appendages paired, filiform, 2–3 mm long. Ovary ¾ of hypanthium in length, apex villous; style glabrous, ca. 9 mm long, pointing in direction opposite to filaments, curved at tip; stigma minute; extra-ovarial chambers 4, shallow, reaching to upper third of ovary. Fruits globose, ca. 6 × 4–5 mm, glabrous, bright green, slightly 8 vertical lines present; calyx lobes remnant persistent. Seeds ca. 0.5 mm long.

##### Distribution.

India (Nicobar Islands), Myanmar, Indochina (Cambodia and Vietnam), southwards through Thailand to western Malesia (Malay Peninsula, Sumatra and Borneo) and Philippines (Palawan).

##### Ecology and habitat.

Secondary lowland forest, opened primary forest or disturbed forest, dipterocarp forest or montane forest from sea level to 1500 m elevation.

##### Vernacular names.

Sumatra: *akar kemunting* (Bintan). Borneo: *kemanti Omang* (Iban); *luka kalapat* (Penan).

##### Note.

Generally recognised as *D.celebica* by most authors because of the similarity in the small flowers with only four fertile stamens. *Dissochaetabiligulata* differs from *D.celebica* by its dark brown furfuraceous indumentum on the leaves below, a truncate calyx with lobes with undulate tips, a white or pinkish-white corolla and globose fruits with distinct lines. In *D.celebica*, the indumentum underneath the leaf blades is less dense, the calyx lobes are glabrous, purple and have a triangular apex, the corolla is dark purple and the fruit ovoid without distinct lines. This species is very common in the open dipterocarp forest in western Malesia.

##### Selected specimens examined.

**INDIA. Nicobar Islands**: East West Road, 20 Jul 1976, M.S. Balakrishnan 3899 (L). **MYANMAR. Tenasserim**: Domel Island, 14 Jan 1829, J.W. Helfer 2286 (K). **CAMBODIA. Kah Kong**: Lhuom Rovaal, 600 m, 27 Feb 1966, Anon 758 (P); Mount Rodam Meong Daeum, 650 m, 23 Feb 1966, Martin 348 (P). **VIETNAM. Ha Tay**: Mount Bavi, Tonkin River, Jun 1908, C. dÁlleizette 2420 (L). **THAILAND. Narathiwat**: Sungei Kolok, 4 Mar 1974. K. Larsen & S.S. Larsen 32923 (K, L). **Pattalung**: Tamote Falls, 225 m, 9 Aug 1986, J.F. Maxwell 86-552 (L). **Pattani**: Bukit, 25 Jan 1931, P. Put 3635 (K). **Surat Thani**: Ko Panghan, 3 Jun 1927, P. Put 778 (BM, K). **Trang**: Khao Pap Pa, 13 Mar 1974, K. Larsen & S.S. Larsen 33279 (P). **Yala**: Nikhom Kulong, 500 m, 31 Dec 1972, T. Santisuk & B. Nimanong BKF 54194 (K, L). **MALAYSIA. Johor**: Kluang, 24 Nov 1967, Alphonso, Sanusi & Sidek S.200 (K, L); Gunung Pulai, 550 m, 29 Jan 1978, J.F. Maxwell 78-24 (L). **Malacca**: W. Griffith KD 2288 (BO, K). **Negri Sembilan**: Gunung Angsi, 730 m, 25 Nov 1923, M. Nur SFN 11708 (BO, K); Nilai, near Seremban, Jinderam Estate, 90 m, 22 Sep 1957, M. Shah 129 (BO, K, L). **Pahang**: Sungai Tahan, 27 Jul 1936, Kiah SFN 31912 (BO, K, PNH); Fraser’s Hill, Girdle Road, 1200 m, 26 Aug 1959, M. Shah & Kadim 680A (BO, K, L). **Penang**: N. Wallich 4050 (K, P); Government Hill, 20 Jan 1903, C. Curtis 3806 (BM, BO). **Perak**: B. Scortechini 23 (K); Larut, King’s collector 2911 (BM, K); Trolak, 22 Mar 1967, S. Chelliah KEP 104685 (K, L); Ulu Bubong, 120–180 m, Jun 1886, King’s collector 10290 (BM, K). **Selangor**: Ulu Gombak, 21 Aug 1968, Y. Teo & J.W. Purseglove 165 (K, L); Kuala Lumpur, 5 Mar 1915, H.N. Ridley s.n. (BM, K, L). **Terengganu**: Kemaman, Sungai Nipah, 24 Jun 1932, E.J.H. Corner SFN 25848 (K). **Sabah**: Beaufort, Klas FR, 23 Jul 1973, Dewol & Abdul Karim SAN 77821 (K, L); Keningau, Kitau, 8 Sep 1982, Amin SAN 95456 (K, L); Kalabakan, Seranum, 21 Sep 1983, Fidilis & Suali SAN 101240 (K, L); Lahad Datu, Danum Valley, 238 m, 6 Jul 2006, Rosalia et al. SAN 145986 (K); Lamag, Sungai Tangkulap, 30 Jun 1983, Amin SAN 97451 (L); Nabawan, Jalan Nabawan-Pandewan, 12 Feb 1990, Sumbing SAN 128120 (K); Pensiangan, Pensiangan Kayu FR., 28 Jan 1994, Fidilis SAN 136931 (K); Tawau, Oct 1922–Mar 1923, A.D.E. Elmer 21840 (BM, BO, K, L, P, PNH, U); Ranau, Kampung Kiau, 30 Nov 1915, M.S. Clemens 10122 (PNH); *Ibid*., Kampung Merungin, 300 m, 18 Nov 1975, Leopold & Saikeh SAN 82620 (K, L); Mt. Kinabalu, 1060 m, 14 Jun 1961, W.L. Chew, E.J.H. Corner & A. Stainton RSNB 68 (K, L); *Ibid.*, Kelawat, near Tamparuli, 900 m, 2 Mar 1954, S. Darnton 230 (BM, L); Sipitang, Mesapol FR, 30 m, 25 Nov 1968, K. Ogata 11671 (L). **Sarawak**: Baram, Kelabit Highland, 1066 m, 6 Nov 1974, P.K. Chai S.35335 (K, L); Belaga, Sungai Murum, 600 m, 13 May 1994, S.H. Lai et al. S.68539 (K); Kuching, Oct 1892, G.D. Haviland 1901 (BM, K); *Ibid.*, Selang FR., 30 m, 25 Jul 1957, Ilias Paie S.8460 (BO, K, L); Limbang, Bukit Pagon, Sungai Sipayan, 540 m, 3 Aug 1984, D. Awa & B. Lee S.47645 (L); Marudi, Bario, 1100 m, 12 Apr 1995, T.E. Beaman & R. Repin 164 (K); Miri, Lambir National Park, 5 Apr 1966, Sibat ak Luang S.25075 (BO, K, L); *Ibid.*, Riam Road, 30 m, 3 Dec 1962, Joseph Au S.17254 (BO, K, L); Betong, Bukit Sadok, 15 Oct 1982, Banyeng & Ilias Paie S.45085 (K, L); Dataran Tinggi Merurong, Sungai Jelalong, 330 m, 9 Oct 1984, Othman & P.C. Yii S.48814 (K, L); Baram, Ulu Koyan, Mount Dulit, 900 m, 15 Sep 1932, P.W. Richards 1818 (K, L). **SINGAPORE.** Feb 1837, C. Gaudichaud-Beaupré 80 (P); Oct 1861, T. Anderson 67 (BM, K, P); Seletar, 27 Mar 1889, H.N. Ridley 2025 (BM); MacRitchie Reservoir 10 m, 15 Jul 1982, J.F. Maxwell 82-194 (L); Pierce Reservoir, 60 m, 20 Oct 1957, H.M. Burkill HMB 1227 (K, L, PNH). **BRUNEI. Belait**: Jalan Merangking-Buau, 10 Aug 1991, N. Nangkat 265 (K, L); Bukit Teraja, 160 m, 27 Sep 1957, P.S. Ashton BRUN 671 (BO, K, L). **Seria**: Andulau FR, 25 m, 31 Jul 1963, H.P. Fuchs & S. Muller 21150 (K, L). **Temburong**: Batu Apoi, 350 m, 29 Oct 1991, D.A. Simpson & S. Marsh 2502 (BO, K, L). **INDONESIA. Aceh**: Mt. Leuser, Gunung Bandahara, 1350 m, 20 Feb 1980, S. Prawiroatmodjo 2386 (BO, K); *Ibid.*, Klut Nature Reserve, Pucuk Lembang, 40 m, 8 Jul 1985, W.J.J.O. de Wilde & B.E.E. de Wilde-Duyfjes 19788 (BO). **Bangka-Belitung**: Bangka, T. Horsfield 19 (K); *Ibid.*, Menumbing, J.E. Teijsmann s.n. (BM, BO, K, U). **Bengkulu**: Lebong Tandai, Apr 1922, C.J. Brooks 6675 (K). **Jambi**: Bangko, 160 m, 10 Aug 1925, O. Posthumus 687 (BO, L); Harapan Rain forest, 31 Mar 2013, Wardi et al. BOHK 390 (BO, K). **North Sumatra**: Asahan, Dolok Tomuan, 1000 m, 20 Aug 1936, Rahmat Si Boeea 9964 (L); Padang Sidempuan, Padang Lawas, Huta Imbaru, 20-21 Jun 1933, Rahmat Si Toroes 4634 (K, L); South Tapanuli, Batang Toru, 640 m, 6 Jun 2003, W. Takeuchi & E.N. Sambas 18228 (BO); Pematang Siantar, 6 Apr 1954, A.H.G. Alston 15233 (BM, L); Besitang, Sikundur, 100 m, 15 Aug 1971, K. Iwatsuki et al. S-401 (BO, L). **Riau**: Indragiri Hulu, Berapit, 13 Apr 1939, P. Buwalda 6535 (BO, K, L, PNH); Rengat, Bukit Tigapuluh, 100 m, 14 Nov 1988, J.S. Burley & Tukirin 1472 (BO, K, L). **Riau Archipelago**: Bintan Island, Gunung Bintan, 40 m, 12 Jun 1919, H.A.B. Bünnemeijer 6114 (BO, L). **West Sumatra**: Tanah Datar, Lembah Anai, 600 m, 24 Dec 1983, M. Rahayu & Maskuri 473 (BO, K); Batu Sangkar, Mt. Sago, 1200 m, 10 Mar 1989, H. Nagamasu 3773 (BO, L); Lima Puluh Kota, Gunung Malintang, 1100 m, 18 Jul 1918, H.A.B. Bünnemeijer 3587 (BO, K, L, P); *Ibid.*, Harau Valley, Sarasah Bonta, 700 m, 12 Sep 2017, A. Kartonegoro 1088 (BO, L); Lubuk Sikaping, Mt. Gadang, 700 m, 15 Jun 1953, J. van Borssum-Waalkes 1888 (BO); Paauw, P.W. Korthals s.n. (L). **Central Kalimantan**: Barito Ulu, 25 Jun 1990, C.E. Ridsdale PBU 692 (BO, L); Bukit Raya, Tumbang Tapi, 100 m, 18 Jan 1983, J.F. Veldkamp 8297 (BO, L). **East Kalimantan**: West Kutai, Besilau Tuwa, 20 m, 22 Jun 1925, F.H. Endert 1593 (BO, L); Sangkulirang, Sampayau, 25 m, 9 Jun 1937, Aet 669 (BO, K, L); East Kutai, Sungai Menubar Region, 5 m, 5 Jun 1951, A.J.G.H. Kostermans 4960 (BO, K, L); Wanariset Research Area, Semoi Road, 20 m, 10 Mar 1992, Ambriansyah & Z. Arifin AA 468 (K, L); Between Papadi & Pamilau, 700–800 m, 9 Aug 1981, R. Geesink 9295 (L, P); Long Iram, Maruwai, Lampunut, 310 m, 19 Mar 1999, P.J.A. Kessler et al. 2657 (L); Wain River, 24 Jun 1910, L.M.R. Rutten 170 (U). **North Kalimantan**: Tarakan, 25 Oct 1953, W. Meijer 1866 (BO, K, L). **South Kalimantan**: Hayup, 28 Jun 1908, HJ.P. Winkler 2627 (BM, BO, K, L, P). **West Kalimantan**: Sungai Blu’u, 1896, Jaheri 704 (BO); Ketapang, Gunung Palung National Park, Cabang Panti, 20 m, 24 Oct 1996, T.G. Laman et al. 110 (BO, K, L). **PHILIPPINES. Palawan**: Taytay, Ibangley, Pagdanan Range, 50–70 m, 31 Jan 1991, D.D. Soejarto & E. Fernando, 7458 (L); *Ibid.*, B.C. Stone et al. PPI 368 (L).

#### 11. Dissochaeta bracteata

Taxon classificationPlantaeMyrtalesMelastomataceae

(Jack) Blume, Flora 14: 495. 1831.

Figure 8 , Map 7

Melastoma bracteatum Jack, Trans. Linn. Soc. London 14: 9. 1823 (“bracteata”).
Dissochaeta bracteata Korth. in Temminck, Verh. Nat. Gesch. Ned. Bezitt., Bot.: 237, tab. 55. 1844, *nom. illeg.*, non Blume (1831). Type: Indonesia. West Sumatra: Doekoe, P.W. Korthals s.n. (lectotype, designated here: L [L0822680]!; isolectotype: L [L0822679]!).
Dissochaeta bracteosa Naudin, Ann. Sci. Nat., Bot. sér. 3, 15: 76. 1851. Type: Malaysia. Pulo Pinang, Mar 1837, C. Gaudichaud-Beaupré 97 (lectotype, designated here: P [P02274815, image seen]!; isolectotype P [P02274816, image seen]!).
Dissochaeta korthalsii Miq., Fl. Ned. Ind. 1(1): 528. 1855, *nom. superfl*. Type: Based on Dissochaetabracteata Korth.

##### Type.

Malaysia. Penang, Jack s.n. (lost); Malaysia. Penang, N. Wallich 4044 (neotype, designated by Kartonegoro and Veldkamp 2010, pg. 129): K [K000859538]!; isoneotypes: BM!, K [K000859537]!).

**Figure 8.**
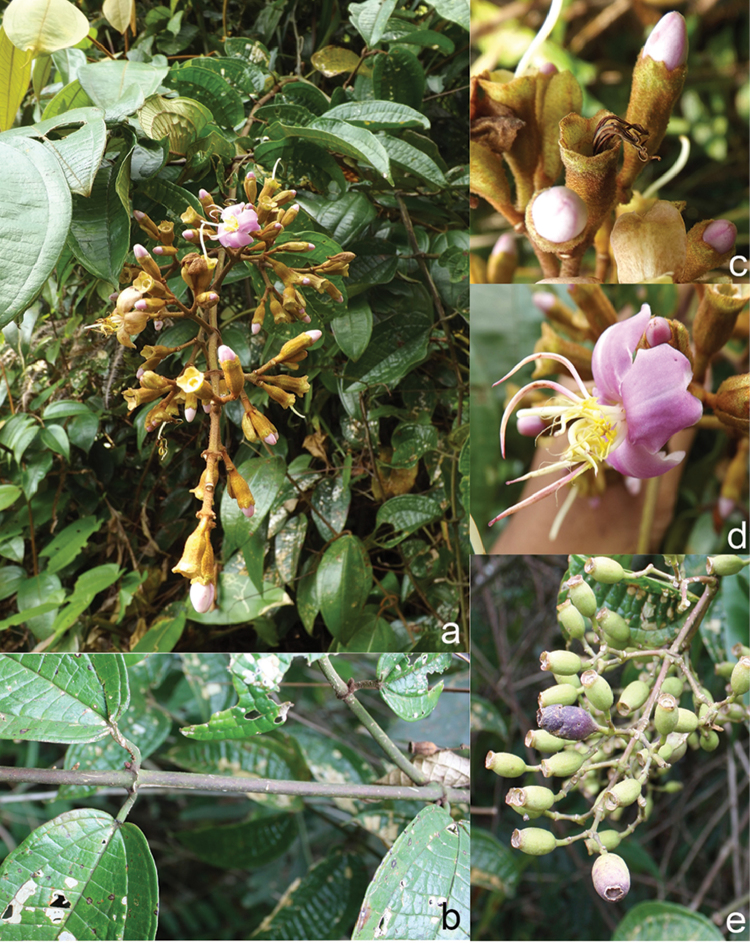
*Dissochaetabracteata***a** habit **b** branchlet **c** hypanthium **d** flower **e** fruits. Photographs by A. Kartonegoro; vouchers Kartonegoro 1074 (BO, L).

**Map 7.**
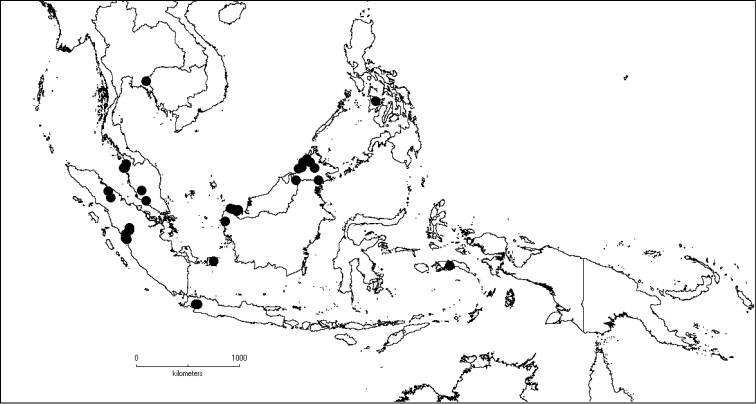
Distribution of *D.bracteata* (●).

##### Description.

Climbing up to 15 m in height. Branchlets terete, 3–5 mm in diameter, stellate-puberulous; nodes swollen, interpetiolar ridge undulate, densely brown pubescent; internodes 8–11 cm long. Leaves: petioles terete, 5–10 mm long, densely brown stellate-furfuraceous; blades ovate, 6–13 × 3–7 cm, chartaceous, base cordate to subcordate, margin entire, apex acuminate, tip 0.3–0.5 cm long; nervation with 1 or 2 pairs of lateral nerves and 1 pair of intramarginal nerves; adaxially glabrous, glossy, with prominent nervation, abaxially glabrous to sparsely stellate-punctate, young leaves densely stellate-furfuraceous. Inflorescences terminal, up to 20 cm long, many-flowered; main axis stellate-puberulous; primary axes up to 15 cm long with 5 or 6 nodes, secondary axes 2.5–3.5 cm long with 1 or 2 nodes, teriary axes 0.8–1 cm long with 1 node; bracts ovate, 10–12 × 5–6 mm, densely brown stellate-furfuraceous; bracteoles ovate or ovate-oblong, 5–9 × 2–4 mm, reflexed inside, enclosing the flower bud, densely stellate-furfuraceous; pedicels brown stellate-furfuraceous, 4–5 mm long in central flowers, 1–2 mm long in lateral flowers. Hypanthium tubular, 3–8 × 2–4 mm, densely brown stellate-furfuraceous, ridge conspicuous; calyx lobes truncate with rounded or triangular tip, 1–2 mm long, stellate-furfuraceous; petal bud conical, 1–4 mm long; mature petals ovate, ca. 10 × 5–7 mm, reflexed, base clawed, apex rounded, glabrous or with appressed hairs at base inside, bright purple with white lines. Stamens 8, unequal, filaments curved sideways, whitish; alternipetalous stamens with 6–8 mm long filaments, anthers linear or lanceolate, curved, sickle-shaped, thecae 13–15 mm long, pink, pedoconnective 3–4 mm, basal crests erose or triangular, irregular, ca. 1 mm long, yellow, lateral appendages paired, filiform, 3–5 mm long, sometimes divided at the apex; oppositipetalous stamens with 5–6 mm long filaments, slightly bent at apex, stipopodium ca. 1 mm long, anthers lanceolate, S-shaped, locule 8–10 mm long, thick, bright white or yellow with pink apex, basal crest hastate, 1–2 mm long, yellow, lateral appendages paired, filiform, 4–5 mm long. Ovary half as long as the hypanthium, apex pubescent; style glabrous or subglabrescent, 6–10 mm long, curved at top; stigma minute; extra-ovarial chambers 8, extending to the middle and the base of the ovary. Fruits urceolate, elongate, 6–10 × 3–5 mm, stellate-puberulous to nearly glabrous, yellowish-green when unripe; calyx lobes caducous. Seeds ca. 0.5 mm long.

##### Distribution.

Thailand (Chanthaburi), Peninsular Malaysia (Kedah, Malacca and Penang), Sumatra (North, West and Belitung), Java (West), Borneo (Sabah and Sarawak), Philippines (Panay) and Moluccas (Ceram).

##### Ecology and habitat.

Secondary montane forest or on the edge of the forest and open area at 600–900 m elevation.

##### Vernacular names.

Peninsular Malaysia: *oosa* (Malay). Sumatra: *andor si ramu dalik* (Batak).

##### Note.

*Dissochaetabracteata* has distinct ovate bracteoles, which cover the hypanthium when in bud. It is sometimes confused with *D.annulata*, which has similar robust chartaceous leaves and inflorescences, but differs in having a much more glabrous or puberulous abaxially leaf surface. The hypanthium of *D.bracteata* is urceolate, more campanulate in *D.annulata*. Even though the species has a wider distribution than any other species, its occurrence is rather scattered in each region with low numbers of samples.

##### Specimens examined.

**THAILAND. Chanthaburi**: Khao Soi Dao, 1800 m, 28 Apr 1930, A.F.G. Kerr 19192 (BM, K). **MALAYSIA. Kedah**: Gurun, Gunung Jerai, 16 Jul 1994, Zainudin et al. 5131 (K, L). **Malacca**: A.C. Maingay KD 791 (1217) (K). **Penang**: N. Wallich 4044 (BM, K); *Ibid.*, Mar 1837, C. Gaudichaud-Béaupre 97 (P); Government Hill, Apr 1890, C. Curtis 2298 (K); Penang Hill, 730 m, 14 Sep 1966, Ding Hou 839 (K, L); Batu Pulau, 1905, W. Fox s.n. (BM). **Selangor**: Genting Highlands, Gunong Ulu Kali, 1600 m, 3 Jun 1978, J.F. Maxwell 78-308 (L). **Sabah**: Beaufort, Quary, 12 Sep 1970, Aban Gibot SAN 66948 (K); Beluran, Ulu Tungud Forest Reserve, 600 m, 24 Jul 2005, Joanes et al. SAN 146905 (K); Papar, Keningau Road, 20 Feb 1975, Abdul Karim SAN 78421 (K, L); Penampang, Inobong, 22 Jul 2010, Aloysius et al. SAN 152101 (L); Ranau, Mount Kinabalu, Kota Belud to Kibayo, 28 Oct 1915, M.S. Clemens 9816 (BO, PNH); *Ibid.*, Dallas, 900 m, Sep 1931, J. Clemens & M.S. Clemens 30339 (BO, K, L); *Ibid.*, Between Kota Belud and Kaliau, 450 m, 11 Mar 1954, S. Darnton 505 (BM); *Ibid.*, Nosurong, 19 May 1986, Amin & Jarius SAN 114333 (L); Tawau, Silimpopon, St. Lucia, 22 m, 5 Jun 1940, P. Orolfo 22 (K, L). **Sarawak**: Kuching, 19 Apr 1893, G.D. Haviland 151 (BM, K, L); *Ibid.*, Mount Santubong, May 1961, Hj. Bujang S.13494 (K); *Ibid.*, Belvedere 15 m, 12 Sep 1955, J.W. Purseglove P.4353 (K, L); Lundu, Sematan, Gunung Pueh, 820 m, 23 Jun 1974, James et al. S.34495 (K, L); *Ibid.*, Pandan, 25 May 1986, Abang Mohtar et al. S.53018 (AAU, L). **INDONESIA. Bangka-Belitung**: Belitung Island, Manggar, J.E. Teijsmann s.n. (BO). **North Sumatra**: Deli Serdang, Bangun Purba, 175 m, 14 Mar 1925, J.A. Lörzing 11439 (BO); Asahan, Dolok Tomouan, 1000 m, 10–15 Jun 1936, Rahmat Si Boeea 9075 (L). **West Sumatra**: Padang, Limau Manis, 400 m, 5 Sep 2017, A. Kartonegoro 1056 (BO, L); Lima Puluh Kota, Pangkalan Koto Baharu, 100 m, Apr 1915, E. Jacobson 2412 (BO); *Ibid.*, Harau Valley, Sarasah Bonta, 500 m, 11 Sep 2017, Kartonegoro 1074 (BO, L); Padang Panjang, 550 m, 1 Aug 1957, W. Meijer 7180 (L); Pariaman, Duku, P.W. Korthals s.n. (L). **West Java**: Bogor, Bolang, Cirangsad, 600 m, 19 Jul 1912, C.A. Backer 4139 (BO). **Moluccas**: Ceram, Masohi, Wae Ruwata, 150-200 m, 2 Dec 1990, J.S. Burley & Tukirin 4325 (BO, K, L). **PHILIPPINES. Panay**: Capiz, Oct-Nov 1925, G.E. Edano BS 46108 (BO, P).

#### 12. Dissochaeta brassii

Taxon classificationPlantaeMyrtalesMelastomataceae

(M.P.Nayar) Karton. comb. nov.

urn:lsid:ipni.org:names:60476834-2

Map 8

Dissochaeta angiensis auct. non Ohwi: Veldkamp, Blumea 24: 441. 1979. *p.p.* excl. type.

##### Basionym.

*Neodissochaetabrassii* M.P.Nayar, Kew Bull. 20: 160. 1966.

##### Type.

Papua New Guinea. Milne Bay: Woodlark Island, Kulumadau, 100 m elev., 14 Nov 1956, L.J. Brass 28743 (holotype: K [K000859607]!; isotype: L [L0537255]!).

**Map 8.**
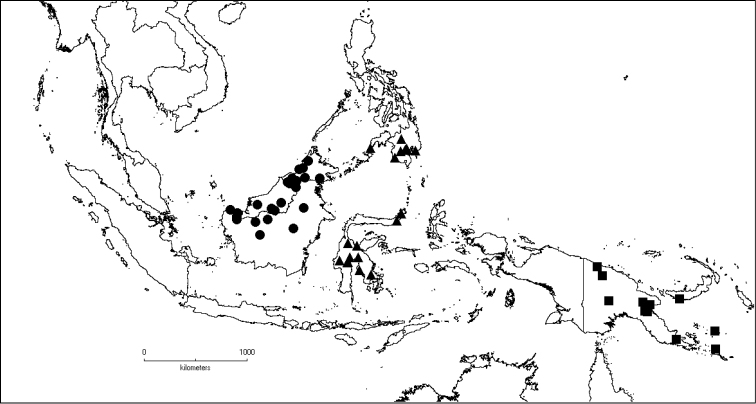
Distribution of *D.beccariana* (●), *D.brassii* (■) and D.celebicavar.celebica (▲).

##### Description.

Climbing up to 10 m in height. Branchlets terete, 3–4 mm in diameter, glabrescent to densely covered with brown stellate-furfuraceous hairs; nodes swollen, with interpetiolar line; internodes 4–6.5 cm long. Leaves: petioles flattened, 1.2–1.6 cm long, densely stellate-furfuraceous; blades elliptic, 9–13.4 × 3.4–6.4 cm, membranous, base rounded or emarginate, margin entire, apex acuminate, tip 1.5–1.8 cm long; nervation with 1 or 2 pairs of lateral nerves and 1 pair of intramarginal nerves; adaxially glabrous, dark glossy green, abaxially densely brown stellate-furfuraceous, rarely rugose. Inflorescences terminal, 15–22 cm long, many-flowered; main axis densely brown stellate-furfuraceous; primary axes up to 15.5 cm long with 3 or 4 nodes, secondary axes 3–6 cm long with 2 or 3 nodes, tertiary axes 0.6–1.8 cm long with 1 node; bracts linear, 1.5–2 cm long, densely tomentose; bracteoles minute, 1–2 mm long, caducous, densely brown stellate-furfuraceous; pedicels stellate-furfuraceous, 2–3 mm long in central flowers, 1–2 mm in lateral flowers. Hypanthium tubular or funnelform, 4–6 × 2–3 mm, densely brown stellate-tomentose; calyx lobes slightly triangular, 2–2.5 mm long; petal bud conical, 5–7 × 2–3 mm, glabrous; mature petals obovate, 7–8 × ca. 4 mm, base clawed, apex rounded, glabrous, white or pale pink. Stamens 4, equal, alternipetalous, filaments straight, 5–6 mm long; anthers oblong-lanceolate, thecae 5–6 mm long, straight, yellow, pedoconnective bent, ca. 1 mm long, basal crest ligular, up to 2 mm long, lateral appendages erose or paired, filiform with irregular margin, 1.5–2.5 mm long. Ovary ⅔ of hypanthium in length, apex pubescent; style 8–9 mm long, glabrous; stigma minute; extra-ovarial chambers 4, extending to the middle of the ovary. Fruits urceolate, obpyriform, 6–8 × 5–6 mm, glabrescent; calyx remnants caducous. Seeds ca. 0.5 mm long.

##### Distribution.

New Guinea (Papua New Guinea).

##### Ecology and habitat.

On the edge of forests, secondary forest or road banks at 100–700 m elevation.

##### Note.

Veldkamp (1979) regards this species as a synonym of *D.angiensis*, which is also distributed in New Guinea and also has only 4 stamens. *Dissochaetabrassii* differs in having slightly triangular, 2–2.5 mm long calyx lobes, while *D.angiensis* has truncate, ca. 1 mm long calyx lobes. The lobes of this species will fall off when fruiting and the shape of the fruits is then sometimes similar to resembling species.

##### Specimens examined.

**PAPUA NEW GUINEA. East Sepik**: Ambunti, Waskuk Hills, 100 m, 28 Jun 1995, J.C. Regalado & W. Takeuchi 1426 (K, L). **Gulf**: Lakekamu, Avi Avi River, 105 m, 25 Oct 1996, W. Takeuchi & J. Kulang 11438 (K, L, P). **Milne Bay**: Maneau Range, Mt. Dayman, 700 m, 17 Jul 1953, L.J. Brass 23485 (L); Woodlark Island, Kulumadau, 100 m, 14 Nov 1956, L.J. Brass 28743 (K, L); Misima Island, Mt. Sisa, 21 Jul 1956, L.J. Brass 27443 (L). **Morobe**: Herzog Mts., above Gabensis, 680 m, 12 Jun 1991, W. Takeuchi 7040 (L); *Ibid.*, W. Takeuchi 7040A (L); Lae, Markham Point, 240 m, 9 Jan 1963, E.E. Henty NGF 14886 (BO, K, L); Along Tymne-Wago track, 600 m, 14 Mar 1963, T.G. Hartley 11395 (K); Labu Swamp, 7 May 1990, W. Takeuchi 5668 (BO, K, L). **New Britain**: Kandrian, Piri Longi, 400 m, 13 Mar 1965, C.D. Sayers NGF 21952 (BO, K, L). **Sepik**: Lumi, Mt. Torricelli, 884 m, 22 Aug 1961, P.J. Darbyshire 247 (K, L). **Southern Highlands**: Lake Kutubu, near Tage, 823 m, 21 Sep 1961, R. Schodde 2189 (K, L).

#### 13. Dissochaeta celebica

Taxon classificationPlantaeMyrtalesMelastomataceae

Blume, Mus. Bot. 1(3): 36. 1849.

Dissochaeta subviridis Elmer, Leafl. Philipp. Bot. 4: 1193. 1911. Type: Philippines. Mindanao: Davao Del Sur, District of Davao, Todaya (Mt. Apo), 3500 ft., May 1909, A.D.E. Elmer 10577 (lectotype, designated here: GH [GH00072246, image seen]!; isolectotypes: BISH [BISH1003278, image seen]!, BM [BM000944483]!, BO [BO1751865]!, CAS [CAS0033376, image seen]!, E [E00680851]!, HBG [HBG514874, image seen]!, K [K000859610]!, L [L0537286]!, MO [MO-313697, image seen]!, NY [NY00228566, image seen]!, P [P02274813, image seen]!, U [U0004006]!, US [US00120533, image seen]!).
Neodissochaeta celebica (Blume) Bakh.*f.*, Contr. Melastom.: 141. 1943.

##### Type.

Indonesia. North Sulawesi: Res. Menado, Tomohon, G. Mahawoe, Feb 1841, E.A. Forsten 305 (lectotype, designated here: L [L0537287]!; isolectotype: L [L0625953]!).

##### Description.

Climbing up to 8 m in height. Branchlets terete, ca. 3 mm in diameter, glabrescent to sparsely covered with brown stellate hairs, more densely so in young parts; nodes swollen, with an interpetiolar line; internodes 5.7–6 cm long. Leaves: petioles terete, 1–1.2 cm long, densely stellate-furfuraceous; blades ovate-elliptic, 8.4–10 × 3.3–4.5 cm, membranous, base rounded to nearly emarginate, margin entire, apex acuminate, tip ca. 0.5 cm long; nervation with 1 pair of lateral nerves and 1 pair of intramarginal nerves; adaxially glabrous, abaxially densely covered with brown stellate-furfuraceous hairs. Inflorescences terminal or in the upper leaf axils, up to 30 cm long, many-flowered; main axis densely covered with brown stellate-furfuraceous hairs; primary axes up to 28 cm long with 5 or 6 nodes, secondary axes up to 6 cm long with 2 or 3 nodes, tertiary axes 1.2–2 cm long with 1 or 2 nodes; bracts lanceolate, 8–11 mm long, densely stellate-furfuraceous, caducous; bracteoles linear, 3–5 mm long, densely stellate-furfuraceous, caducous; pedicels covered with brown stellate hairs, 1–2 mm long in central flowers, ca. 0.5 mm long in lateral flowers or latter subsessile. Hypanthium campanulate funneliform, 3–4 × 1–1.5 mm, densely covered with brown stellate-furfuraceous hairs; calyx lobes truncate with small triangular tips, 1–3 mm long, glabrous, purple inside; petal bud conical, 2–3.5 mm long; mature petals obovate, 3–4 × ca. 2 mm long, reflexed, base clawed, apex rounded, dark purple, glabrous. Stamens 4, equal, alternipetalous; filaments glabrous, curved sideways, 4–5 mm long, bent at the end, white pinkish; anthers oblong, curved, thecae 3–4 mm long, yellowish-white, pedoconnective ca. 1 mm long, basal crest triangular or rounded, ca. 0.5 mm long, lateral appendages filiform, 2–3 mm long, paired, with irregular margin, brownish. Ovary ¾ of hypanthium in length, apex pubescent; style glabrous, 5–6 mm long, curved at the end, purple; stigma minute; extra-ovarial chambers 4, shallow. Fruits subglobose or ovoid, 5–8 × 3–5 mm long, densely covered with stellate hairs and becoming glabrous when mature, calyx lobes persistent, 1–3 mm long. Seeds numerous, ca. 0.5 mm long.

##### Distribution.

Sulawesi and Philippines (Mindanao).

##### Note.

See under *D.biligulata*.

#### Key to varieties of *D.celebica*

**Table:** 

1	Calyx lobes truncate with small triangular tips, ca. 1 mm long, glabrous, purple inside; petal bud 2–2.5 mm long; fruits ovoid, 5–8 × 3–5 mm long, without longitudinal lines	**var. celebica**
–	Calyx lobes slightly triangular, 2–3 mm long, densely brown stellate-furfuraceous; petal bud 3–3.5 mm long; fruits subglobose, 6–8 × 4–5 mm long, slightly 8-lined	**var. longilobata**

#### 13.1. Dissochaeta celebica Blume var. celebica

Taxon classificationPlantaeMyrtalesMelastomataceae

Fig. 9 , Map 8

##### Description.

Hypanthium campanulate-funneliform, 3–4 × ca. 1 mm long, densely covered with brown stellate-furfuraceous hairs; calyx lobes truncate with small triangular tips, ca. 1 mm long, glabrous, purple inside; petal bud conical, 2–2.5 mm long. Fruits ovoid, 5–8 × 3–5 mm long, densely covered with stellate hairs and becoming glabrous when mature, calyx lobes persistent, erect, ca. 1 mm long.

##### Distribution.

Sulawesi and Philippines (Mindanao).

##### Ecology.

Open montane forest at 600–1900 m elevation.

##### Vernacular names.

Philippines: *getungu ulangan* (Zamboanga); *lebong* (Cebuano); *tolasola* (Bagobo).

**Figure 9.**
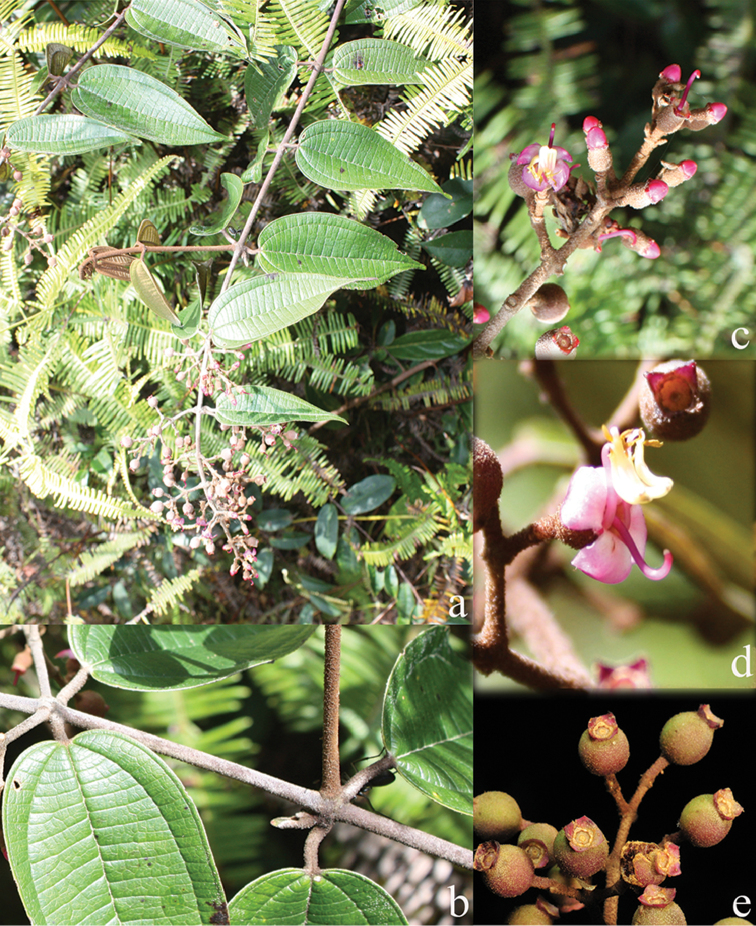
Dissochaetacelebicavar.celebica**a** habit **b** branchlet **c** hypanthium **d** flowers **e** fruits. Photographs by Supriatna; voucher: Widjaja et al. 9846a (BO).

##### Specimens examined.

**INDONESIA. Central Sulawesi**: Poso, Between Boro and Sungai Malei, 1700 m, 11 Aug 1937, P.J. Eyma 1656 (BO); Lore Lindu Area, Sopu Valley, 1000 m, 22 May 1979, E.F. de Vogel 5517 (BO, K, L). **North Sulawesi**: Tomohon, Mt. Mahawu, Feb 1841, E.A. Forsten 305 (L); *Ibid.*, 800 m, 13 Feb 2009, D. Girmansyah 1187 (BO); Bolaang Mongondow, Mt. Ambang, Lake Moat Area, 1000 m, 14 Apr 1985, E.F. de Vogel & J.J. Vermeulen 7177 (BO, L). **South Sulawesi**: Malili, between Takolekaju and Tawi Baru, 30 Oct 1938, P.J. Eyma 4168 (BO); Masamba, between Mabusa and Sae, 1700 m, 21 Jul 1937, P.J. Eyma 1167 (BO, L); Rantepao, on the way to Palopo, 14 Feb 1993, J.J. Afriastini 2125A (BO, K, L); Tojambu, 1000 m, 28 Jun 1929, G.K. Kjellberg 1820a (BO). **South East Sulawesi**: Mt. Mekongga, Tinukari Village, 1900 m, 11 Jul 2011, E.A. Widjaja et al. 9846a (BO); *Ibid.*, Hura-Hura, 1426 m, 28 Nov 2010, E.A. Widjaja & A. Sujadi 9400 (BO); Rawa Aopa, 26 Dec 1978, S. Prawiroatmodjo & Soewoko 1984 (L). **West Sulawesi**: Mt. Papandangan, 1913, Rachmat 397 (BO, L). **PHILIPPINES. Mindanao**: Bukidnon Subprovince, Mt. Candoon, Jun-Jul 1920, M. Ramos & G.E. Edano BS 38870 (BO); Cotabato, Kidapawan, Mt. Apo, Mar-Apr 1991, Gaerlan, Alvarez & Garcia PPI 2635 (L); *Ibid.*, Koronadakal, Mt. Magulo, 1455 m, 10 Apr 1992, Gaerlan, Fuentes & Romero PPI 5245 (L); Davao del Sur, Todaya, Mt. Apo, May 1909, A.D.E. Elmer 10577 (BISH, BM, BO, CAS, E, GH, K, L, MO, NY, P, U, US); *Ibid.*, 914 m, 4 Apr 1905, R.S. Williams 2571 (K); *Ibid.*, Gumate District, 1000 m, Mar 1964, Anon. ANU 1541 (L); *Ibid.*, Mt. McKinley, 1066 m, 29 Aug 1946, G.E. Edano PNH 1042 (L, PNH); Davao Oriental, Mount Galintan, Jun 1927, M. Ramos & G.E. Edano BS 48858 (P); Zamboanga del Norte, 600 m, 1 Feb 1958, C.O. Frake PNH 38277 (L).

#### 13.2. Dissochaeta celebica Blume var. longilobata

Taxon classificationPlantaeMyrtalesMelastomataceae

Karton. var. nov.

urn:lsid:ipni.org:names:60476832-2

Fig. 10 , Map 9

##### Type.

Indonesia. Central Sulawesi: Mount Roreka Timbu, 2000 m elev., 8 May 1979, M.M.J. van Balgooy 3205 (holotype: BO!; isotypes: K!, L [L0652533]!).

##### Description.

Hypanthium campanulate, 3–4 × 1–1.5 mm long, densely covered with brown stellate-furfuraceous hairs; calyx lobes slightly triangular, 2–3 mm long, densely brown stellate-furfuraceous; petal bud conical, 3–3.5 mm long. Fruits subglobose, 6–8 × 4–5 mm, densely covered with stellate hairs and becoming glabrous when mature, slightly 8-lined; calyx lobes persistent, 2–3 mm long.

**Map 9.**
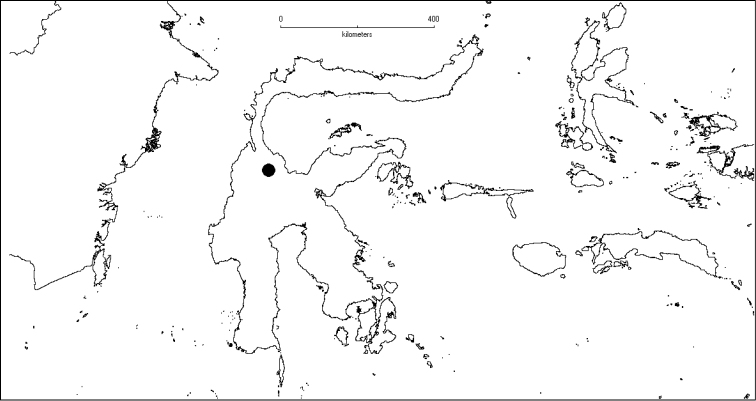
Distribution of D.celebicavar.longilobata (●).

##### Distribution.

Sulawesi (Central Sulawesi).

##### Ecology and habitat.

Montane forest dominated by *Agathis* at ca. 2000 m elevation.

##### Note.

This variety is known only from the type and differs from the var. celebicca by its long triangular calyx lobes (2–3 mm long), persistent in fruit. The length of the calyx lobes is often similar to that of *D.schumannii* from New Guinea, though they are usually caducous in that species.

**Figure 10.**
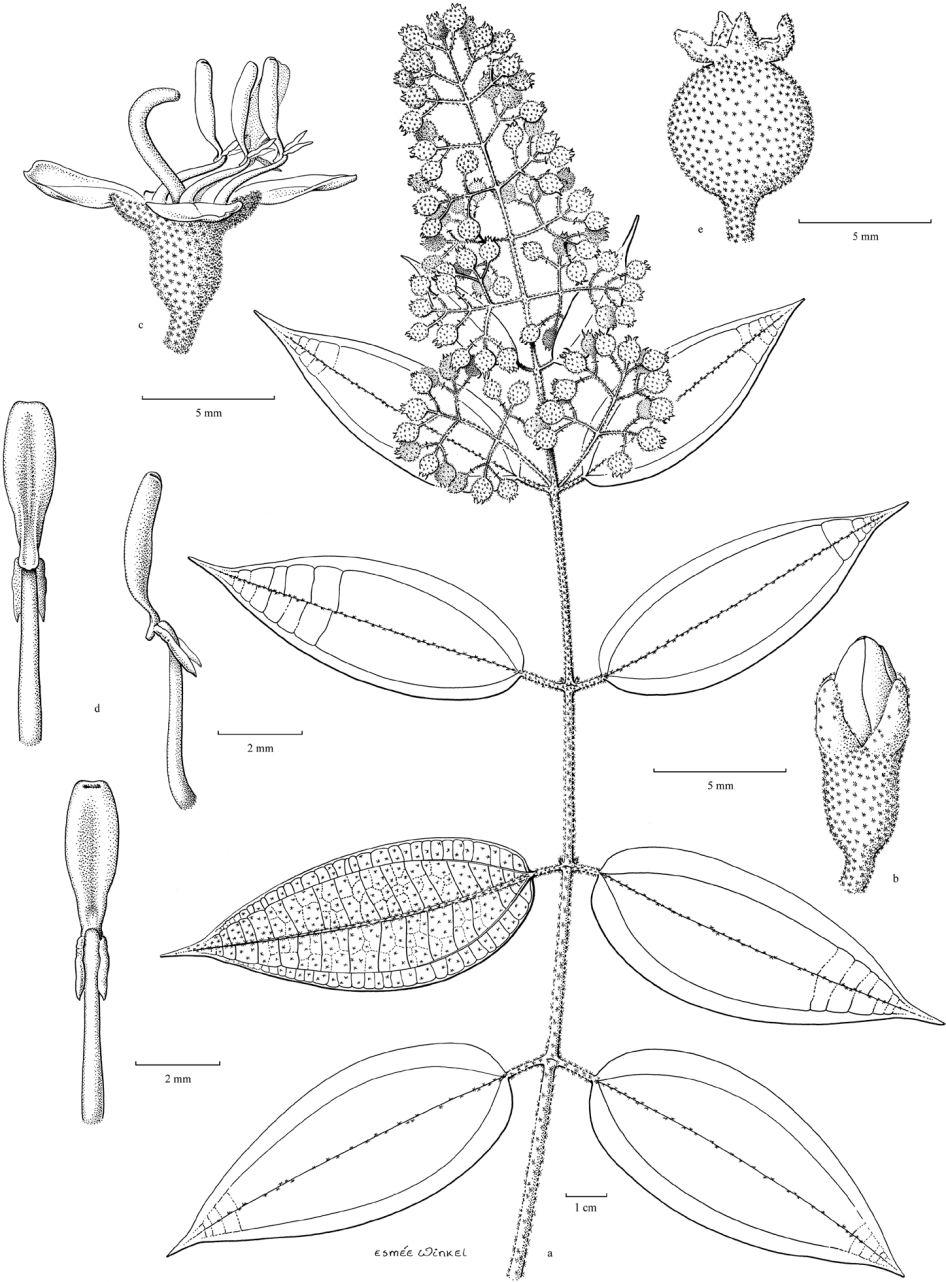
Dissochaetacelebicavar.longilobata**a** habit **b** hypanthium **c** open flower **d** stamens **e** fruit. [drawn from Van Balgooy 3205 (L).

#### 14. Dissochaeta conica

Taxon classificationPlantaeMyrtalesMelastomataceae

(Bakh .f.) Clausing in S.S.Renner et al., Fl. Thailand 7(3): 423. 2001.

Fig. 11 , Map 10

Diplectria conica Bakh.*f.*, Contr. Melastom.: 202. 1943.
Anplectrum crassinodum Merr., Pap. Michigan Acad. Sci. 24: 85. 1939, *nom. illeg.*, non Merr. 1929. Type: Indonesia. North Sumatra: Asahan, Near Loemban Ria, 5–12 Apr 1934, Rahmat si Boeea 7781 (lectotype, designated by Veldkamp et al. 1979, pg. 414: MICH [MICH-1111809, image seen]!; isolectotypes: L [L0008856]!), S [SG-439, image seen]!).

##### Type.

Indonesia. West Sumatra: Agam, Brani, 950 m elev., 19 Jun 1918, H.A.B. Bünnemeijer 3094 (holotype: L [L0537295]!; isotypes: BO [BO1865987, BO1865988]!, L [L0537294]!).

**Figure 11.**
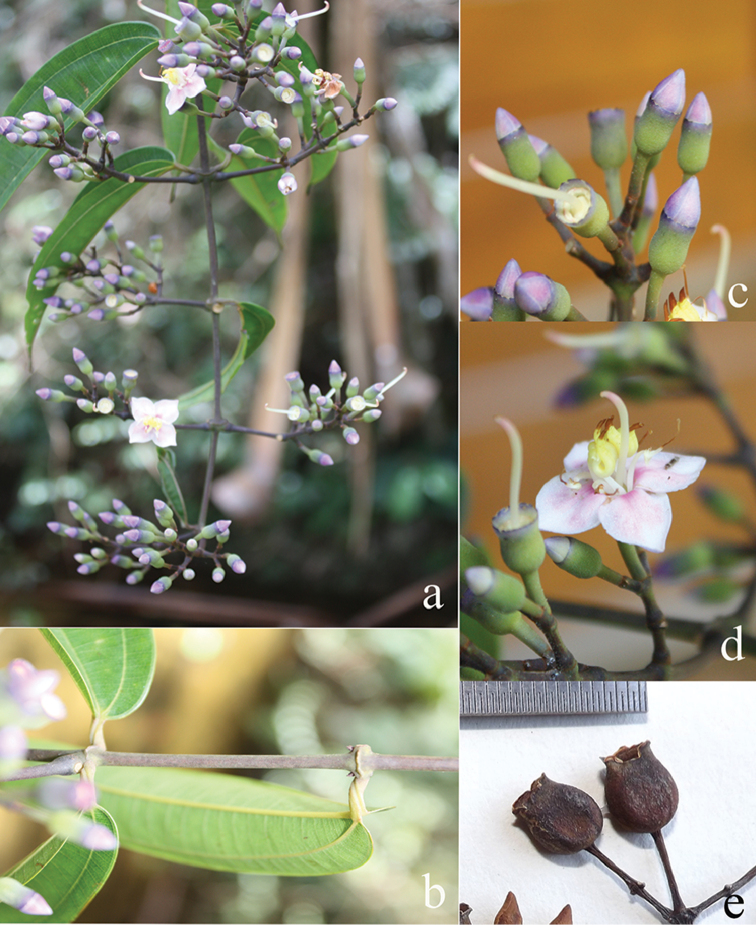
*Dissochaetaconica***a** habit **b** branchlet **c** hypanthium **d** flower **e** fruits. Photographs by A. Kartonegoro; vouchers: Kartonegoro 1078 (BO, L) & Kartonegoro 1101 (BO, L).

**Map 10.**
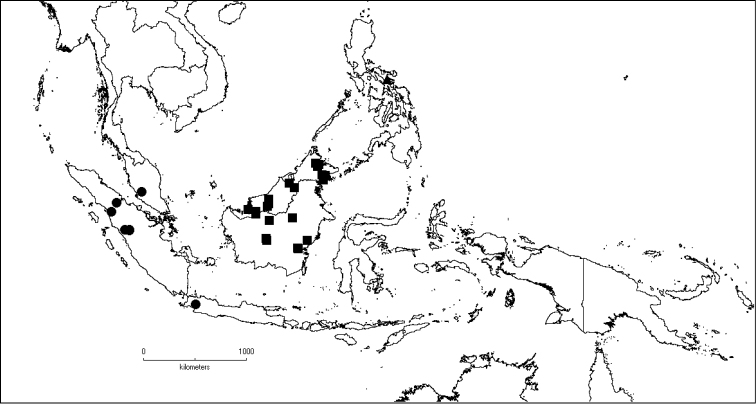
Distribution of *D.conica* (●), *D.cumingii* (▲) and *D.densiflora* (■).

##### Description.

Climbing up to 4 m in height. Branchlets terete, 4–5 mm in diameter, glabrous; nodes swollen, with raised interpetiolar ridge; internodes 4–6.5 cm long. Leaves: petioles terete, 5–10 mm long, glabrous; blades ovate to ovate-elliptic, 10–16 × 4.5–6.5 cm, subcoriaceous, base rounded, margin entire, apex acuminate, tip 0.5–1 cm long; nervation with 1 pair of lateral nerves and 1 pair of intramarginal nerves; surfaces glabrous. Inflorescences terminal and axillary, when terminal, up to 20 cm long and many-flowered, when axillary, up to 10 cm long and with 3–10 flowers; main axis angular, glabrous; primary axes up to 16 cm long with 4 or 5 nodes, secondary axes 1.5–4 cm long with 1–3 nodes, tertiary axes up to 1.5 cm long with 1 node; bracts linear, 2–4 mm long, glabrous, caducous; bracteoles linear, 1–2 mm long, glabrous; pedicels glabrous, 5–8 mm long in central flowers, 4–5 mm long in lateral flowers. Hypanthium cyathiform-tubular, cup-shaped, 7–8 × ca. 5 mm, glabrous; calyx lobes truncate, 1–2 mm long, without distinct tips or with irregularly cracked or rounded tips; petal bud conical, 5–6 mm long; mature petals ovate, 7–8 × ca. 6 mm, reflexed, white or pale purple, base clawed, apex acute. Stamens 8, unequal, filaments straight; alternipetalous stamens staminodial, with ca. 4 mm long filaments, thecae rudimentary, slender, terete, sinuate, ca. 6 mm long, basal crest oblong to triangular, ca. 1 mm long, acute at tip, thin, lateral appendages paired, ligular, ca. 1 mm long; oppositipetalous stamens with 6–7 mm long filaments, anthers thick, curved, S-shaped, thecae 9–10 mm long, connective crest bifid, erose, ca. 0.75 mm long, basal appendages paired, spur-like, ca. 1 mm long, erect. Ovary half as long as hypanthium, apex glabrous; style curved at the end, slender, ca. 15 mm long, glabrous; stigma minute, papillose; extra-ovarial chambers 4, oppositipetalous, extending almost to the base of the ovary. Fruits subglobose to urceolate, 7–8 × 5–6 mm, glabrous; calyx lobes remnant persistent, erect. Seeds ca. 0.5 mm long.

##### Distribution.

Peninsular Malaysia, Sumatra and Java (West).

##### Ecology and habitat.

Low montane forest in open places at 600–950 m elevation.

##### Specimens examined.

**MALAYSIA. Selangor**: Ulu Gombak, 15 Jun 1967, J.C. Carrick 1569 (K, L). **INDONESIA. North Sumatra**: Asahan, Lumban Ria, Rahmat Si Boeea 7781 (L, MICH, S); Tapanuli Selatan, Batang Toru, Telek Nauli, 885 m, 25 Mar 2004, W. Takeuchi et al. 18794 (L). **West Sumatra**: Agam, Brani, 950 m, 19 Jun 1918, H.A.B. Bünnemeijer 3094 (BO, L); Lima Puluh Kota, Harau Valley, Sarasah Bonta, 500 m, 11 Sep 2017, A. Kartonegoro 1078 (BO, L); *Ibid.*, Kelok Sembilan, 800 m, 13 Sep 2017, A. Kartonegoro 1101 (BO, L). **West Java**: Bogor, Nanggung, Mt. Menapa, 600 m, 18 Dec 1940, C.G.G.J. van Steenis 17412 (BO, K, L).

#### 15. Dissochaeta cumingii

Taxon classificationPlantaeMyrtalesMelastomataceae

Naudin, Ann. Sci. Nat., Bot. sér. 3, 15: 75. 1851.

Map 10

##### Type.

Philippines. Luzon: Province of Albay, H. Cuming 1344 (lectotype, designated here: P [P02274812, image seen]!; isolectotypes: BM!, K [K000859608, K000859609]!, L [L0537227]!).

##### Description.

Branchlets terete, 3–4 mm in diameter, densely brown stellate-furfuraceous; nodes swollen, with interpetiolar line; internodes 7–8 cm long. Leaves: petioles flattened, 8–10 mm long, densely brown stellate-furfuraceous; blades ovate-oblong, 7.8–12 × 2.4–4.3 cm, membranous, base emarginate, margin entire, apex acuminate, tip 0.5–1 cm long; nervation with 1 pair of lateral nerves and 1 pair of intramarginal nerves; adaxially glabrous, abaxially densely covered with brown stellate-furfuraceous hairs. Inflorescences terminal or in the upper leaf axils, up to 28 cm long, many-flowered; main axis angular, densely covered with brown stellate-furfuraceous hairs; primary axes up to 25 cm long with 5 nodes, secondary axes up to 6 cm long with 2 or 3 nodes, tertiary axes 0.7–1 cm long with 1 node; bracts linear, 5–6 mm long, densely stellate-furfuraceous, caducous; bracteoles linear, 3–4 mm long, densely stellate-furfuraceous, caducous; pedicels covered with brown stellate hairs, 2–3 mm long in central flowers, 1–2 mm long in lateral flowers. Hypanthium campanulate, angular, 3–5 × ca. 2 mm, densely covered with brown stellate-furfuraceous hairs; calyx lobes truncate with triangular tip, ca. 1 mm long, densely stellate-furfuraceous; petal bud conical, 2–3 mm long, blades elliptic, 5–5.5 × ca. 2 mm; mature petals not seen. Stamens 8, subequal, filaments glabrous, curved sideways; alternipetalous stamens with 6–7 mm long filaments, anthers lanceolate, curved, sickle-shaped, thecae 6–7 mm long, pedoconnective 1–2 mm long, basal crest triangular, ca. 1 mm long, lateral appendages paired, filiform, with irregular margin, 3–4 mm long; oppositipetalous stamens with 5–6 mm long filaments, anthers lanceolate, hook-shaped, thecae 4–5 mm long, basal crest minute, bifid or spuriform, ca. 0.5 mm long, lateral appendages paired, filiform, 3–4 mm long. Ovary ¾ of hypanthium in length, apex pubescent; style slightly curved at apex, 6–8 mm long, glabrous; stigma minute; extra-ovarial chambers 8, extending to the middle of the ovary. Fruits ovoid, 6–7 × 4–5 mm, densely covered with brown stellate-furfuraceous, slightly 8-lined; calyx remnants persistent, erect. Seeds ca. 0.5 mm long.

##### Distribution.

Philippines (Luzon).

##### Note.

This species is known only from the type and lacks any collection notes. The description of the flowers is based on immature flowers which, in some characters, such indumentum and the number and shape of the stamens, resemble *D.sagittata* Blume from Java.

#### 16. Dissochaeta densiflora

Taxon classificationPlantaeMyrtalesMelastomataceae

Ridl., Kew Bull. 1: 32. 1946.

Map 10

Dissochaeta rostrata Korth. var. densiflora (Ridl.) J.F.Maxwell, Gard. Bull. Singapore 33: 319. 1980.
Dissochaeta rostrata Korth. var. esetosa J.F.Maxwell, Gard. Bull. Singapore 33: 319, fig. 4. 1980. Type: Indonesia. East Kalimantan: W. Koetai, Hikam Batoe Beng, 25 m elev., 29 Jul 1925, F.H. Endert 2304 (holotype: L [L0537223]!; isotypes: BO [BO1850828]!, K [K000859632]!).

##### Type.

Malaysia. Sarawak: Saribas, Sungai Plandok, 19 Jul 1892, G.D. Haviland 1550 (lectotype, designated here: K [K000859631]!; isolectotype: SAR *n.v.*).

##### Description.

Climbing up to 10 m in height. Branchlets terete but angular at top part, 2–3 mm in diameter, densely covered with brown stellate-tomentose hairs and scattered short bristle hairs; nodes swollen, with interpetiolar line; internodes 7.3–14 cm long. Leaves: petioles terete, 4–10 mm long, densely stellate-tomentose and with scattered short bristle hairs; blades ovate-elliptic to elliptic, 8.3–11.5 × 3.8–6.5 cm, membranous, base emarginate, margin entire, apex acuminate, tip ca. 0.5 cm long; nervation with 1 pair of lateral nerves and 1 pair of intramarginal nerves; adaxially glabrous except stellate hairs at midrib, abaxially densely covered with brown stellate-tomentose hairs. Inflorescences terminal, up to 25 cm long, many-flowered; main axis densely covered with brown stellate-tomentose and bristle hairs; primary axes up to 20 cm long with 6 or 7 nodes, secondary axes 2.5–5 cm long with 2 or 3 nodes, tertiary axes 6–8 mm long with 1 node; bracts lanceolate or linear, ca. 7 × 1 mm, densely stellate-tomentose and with bristle hairs, caducous; bracteoles lanceolate, ca. 3.5 × 1 mm, densely stellate-tomentose outside, glabrous inside, margin with bristle hairs, caducous; pedicels brown stellate-tomentose, 2–3 mm long in central flowers, 1–1.5 mm long in lateral flowers. Hypanthium tubular, ca. 4 × 2 mm, densely brown stellate-tomentose and with scattered short bristle hairs; calyx lobes triangular, ca. 1 mm long, margin with bristle hairs, apex acute, tomentose; petal bud conical, ca. 2.5 mm long, apex bristly; mature petals ovate, 5–6 × 3–4 mm long, reflexed, base clawed, apex obtuse and bristly, rest glabrous, pale purple or purple. Stamens 8, unequal, filaments glabrous, white, curved sideways; alternipetalous stamens with 5–7 mm long filaments, anthers slender with narrow tip, curved, sickle-shaped, thecae 7–10 mm long, yellow, pedoconnective 1–1.5 mm long, basal crest triangular or ligular, 1–1.5 mm long, lateral appendages paired, filiform, 2–3 mm long; oppositipetalous stamens with 5-6 mm long filaments, anthers S-shaped, thecae 6–8 mm long, basal crest ligular, ca. 1 mm long, lateral appendages paired, filiform, ca. 2 mm long. Ovary ⅔ of hypanthium in length, apex pubescent; style curved at the end, 10–12 mm long, glabrous, white; stigma minute, light purple; extra-ovarial chambers 8, shallow or nearly to the middle of the ovary. Fruits ovoid, 5–6 × 3–3.5 mm, densely covered with stellate-tomentose hairs, sometimes becoming caducous when mature, calyx lobes persistent, reflexed. Seeds ca. 0.5 mm long.

##### Distribution.

Borneo.

##### Ecology and habitat.

Secondary forest along logging road and river banks or submontane forest at 400–1320 m elevation.

##### Vernacular names.

*akar kemunting* (Iban); *lamoy puruk bawi* (Dayak).

##### Specimens examined.

**MALAYSIA. Sabah**: Beluran, Bidu-Bidu Forest Reserve, 21 Jul 1970, L. Madani SAN 128874 (K, L); Lahad Datu, Danum Valley, Ulu Segama, 170 m, 25 Feb 1986, P.J. Edwards 2110 (K, L, P); Lamag, Ulu Sungai Lokan, 12 Nov 1979, Aban & Petrus SAN 90697 (K, L); Sandakan, Telupid Road, 15 Aug 1979, Aban Gibot SAN 91256 (K); Mostyn, Kalumpang Forest Reserve, 17 Feb 1966, Nordin & Ali SAN 54413 (K); *Ibid.*, Tingkayu Camp, 180 m, 14 Sep 1966, J.Sinanggul SAN 57228 (K, L). **Sarawak**: Kapit, Bukit Raya, 14 Jan 1965, Jugah ak Kudi S.23863 (K); *Ibid.*, Pelagus, 7 Jul 1979, B. Lee S.40214 (L); *Ibid.*, Upper Rejang River, 1929, J. Clemens & M.S. Clemens 21139 (BO, K); *Ibid.*, J. Clemens & M.S. Clemens *21569* (K); Kakus, Ulu Mayeng, 150 m, 13 Jul 1964. Sibat ak Luang S.21720 (K, L); Ibid., Tau Range, 152 m, 7 Jun 1956, J.W. Purseglove 5401 (K); Lubok Antu, Lanjak Entimau, Bukit Sengkajang, 600 m, 18 Mar 1974, P.K. Chai S.33998 (K, L); *Ibid.*, Ulu Sg. Bengkari, 21 Mar 1974, P.K. Chai S.34082 (K, L); Miri, Gunung Mulu, 1320 m, 8 Mar 1990, P.C. Yii & Abu Talib S.58220 (L); Saribas, Sungai Plandok, 19 Jul 1892, G.D. Haviland 1550 (K). **INDONESIA. Central Kalimantan**: Bukit Raya, Upper Katingan River, Tumbang Samba, 150 m, 22 Dec 1982, J.P. Mogea & W.J.J.O. de Wilde 4339 (BO, K, L); *Ibid.*, Tumbang Tubus, 150 m, 6 Jan 1983, J.F. Veldkamp 8077 (BO, L). **East Kalimantan**: West Kutai, Hikam Batu Beng, 25 m, 29 Jul 1925, F.H. Endert 2304 (BO, K, L); Balikpapan, Gunung Meratus, 25 Jun 2003, Arbainsyah et al. AA 3115 (BO, K, L); *Ibid.*, Road Kenangan to Mount Meratus, 400 m, 27 Mar 1995, P.J.A. Kessler et al. 913 (K, L, P).

#### 17. Dissochaeta divaricata

Taxon classificationPlantaeMyrtalesMelastomataceae

(Willd.) G.Don, Gen. Hist. 2: 783. 1832.

Fig. 12 , Map 11

Melastoma divaricatum Willd., Sp. Pl., ed. 4, 2(1): 596. 1799 (“divaricata”).
Melastoma glaucum Jack, Trans. Linn. Soc. London 14: 15. 1823 (“glauca”). Type: Malaysia. Peninsular Malaysia, Penang, 1819, W. Jack 49 (lectotype, designated by Veldkamp et al. 1979, pg. 417: E [E00288100]!; isolectotypes: BM [BM000944473]!, G [G00353714, image seen]!, L [L0008857]!).
Melastoma cyanocarpum Blume, Bijdr. Fl. Ned. Ind. 17: 1073. 1826. Type: Indonesia. West Java: G. Salak, C.L. Blume 616 (lectotype, designated by Veldkmap et al. 1979, pg. 417: L [L0008862]!; isolectotypes: K [K000859552]!, L [L0008861, L0008863, L0008864, L0008865]!).
Dissochaeta glauca (Jack) Blume, Flora 14: 501. 1831.
Dissochaeta cyanocarpa (Blume) Blume, Flora 14: 501. 1831.
Osbeckia tetrandra Roxb., Fl. Ind. 2: 224. 1832. Type: Malaysia. Pulo Pinang, W. Roxburgh s.n. (lectotype, designated here: G [G00353906, image seen]!).
Dissochaeta anceps Naudin, Ann. Sci. Nat., Bot. sér. 3, 15: 69. 1851. Type: Indonesia. Lampung: Gunung Batin, 20 Sep 1845, H. Zollinger 3044 (lectotype, designated by Veldkamp et al. 1979, pg. 417: P [P05259330, image seen]!; isolectotypes: BM [BM000944474]!, BO [BO1752509]!, BR [BR5188239, image seen]!, G [G00353563, G00353564, images seen]!, MPU [MPU-013527, image seen]!, P [P05283594, image seen]!).
Dissochaeta spoliata Naudin, Ann. Sci. Nat., Bot. sér. 3, 15: 69. 1851. Type: Malaysia. Peninsular Malaysia, Pulo Pinang, Mar 1837, C. Gaudichaud-Beaupré 95 (lectotype, designated by Veldkamp et al. 1979, pg. 417: P [P05259341, image seen]!; isolectotype: G [G00353567, image seen]!, P [P05259342, image seen]!).
Dissochaeta pepericarpa Naudin, Ann. Sci. Nat., Bot. sér. 3, 15: 71. 1851. Type: Malaysia. Peninsular Malaysia, Malacca, 1821, H. Cuming 2259 (lectotype, designated by Veldkamp et al. 1979, pg. 417]: P [P05259335, image seen]!; isolectotypes: BR [BR5187911, BR5188567, images seen]!, G [G00353565, G00353566, images seen]!, K [K000859566]!, L [L.2537846, L0008858, L0008859, L0008860]!).
Dissochaeta palembanica Miq., Fl. Ned. Ind., Eerste Bijv. 2: 317. 1861. Type: Indonesia. South Sumatra: Res. Palembang, Enim, Pandan Oeloe, J.E. Teijsmann HB 3634 (lectotype, designated by Bakhuizen van den Brink *f.* 1943, pg. 202: U [U0004003]!; isolectotype: BO [BO1752507, BO1426869]!).
Anplectrum divaricatum (Willd.) Triana, Trans. Linn. Soc. London 28: 84. 1872.
Anplectrum glaucum (Jack) Triana, Trans. Linn. Soc. London 28: 84. 1872.
Anplectrum cyanocarpum (Blume) Triana, Trans. Linn. Soc. London 28: 84. 1872.
Anplectrum divaricatum (Willd.) Triana var. anceps (Naudin) Cogn. in Boerl., Handl. Fl. Ned. Ind. 2: 534. 1890.
Diplectria divaricata (Willd.) Kuntze, Revis. Gen. Pl. 1: 246. 1891.
Diplectria cyanocarpa (Blume) Kuntze, Revis. Gen. Pl. 1: 246. 1891.
Diplectria tetrandra (Roxb.) Kuntze, Revis. Gen. Pl. 1: 246. 1891.
Dissochaeta furfurascens Elmer, Leafl. Philipp. Bot. 8: 2754. 1915. Type: Philippines. Mindanao: Agusan Province, Cabadbaran (Mt. Urdaneta), Jul 1912, A.D.E. Elmer 13352 (lectotype, designated here: BO [BO1752508]!; isolectotypes: BISH [BISH-1003259, image seen], BM [BM000944475]!, E [E00288099]!, GH [GH00072244, GH00072245, images seen]!, HBG [HBG514872, image seen]!, K [K000859549]!, L [L0008868]!, MO [MO313698, image seen]!, NY [NY00228563, image seen]!, P [P05259310, image seen]!, PNH [PNH198551, image seen]!, U [U0004004]!).
Anplectrum suluense Merr., Philipp. J. Sci., C 30: 417. 1926. Type: Philippines. Sulu Archipelago: Jolo, Sep 1924, M. Ramos & G.E. Edano BS 44461 (lectotype, designated here: K [K000859548]!; isolectotypes: L [L0008866]!, NY [NY00221310, image seen]!).
Anplectrum patens Geddes, Bull. Misc. Inform. Kew: 72. 1928. Type: Thailand. Pattani: Bachaw, 50 m, 16 Jul 1923, A.F.G. Kerr 7215 (lectotype, designated by Veldkamp et al. 1979, pg. 417: K [K000859554]!; isolectotypes: BK [BK216091, image seen]!, BM!, TCD [TCD-0016998, image seen]!).
Backeria divaricata (Willd.) Raizada, Indian Forester 94: 435. 1968.
Backeria glauca (Jack) Raizada, Indian Forester 94: 435. 1968.
Diplectria furfurascens (Elmer) M.P.Nayar in Veldkamp et al., Blumea 24: 413, fig. 2A. 1979.

##### Type.

India Orientali, Klein 2 ”8218” in Herb. Rottler (lectotype, designated by Veldkamp et al. 1979, pg. 417: B-W [81218-010, image seen]!; isolectotype: C [C10014562, C10014563, images seen]!, K [K000859557]!, L [L0008867, fragm.]!).

**Figure 12.**
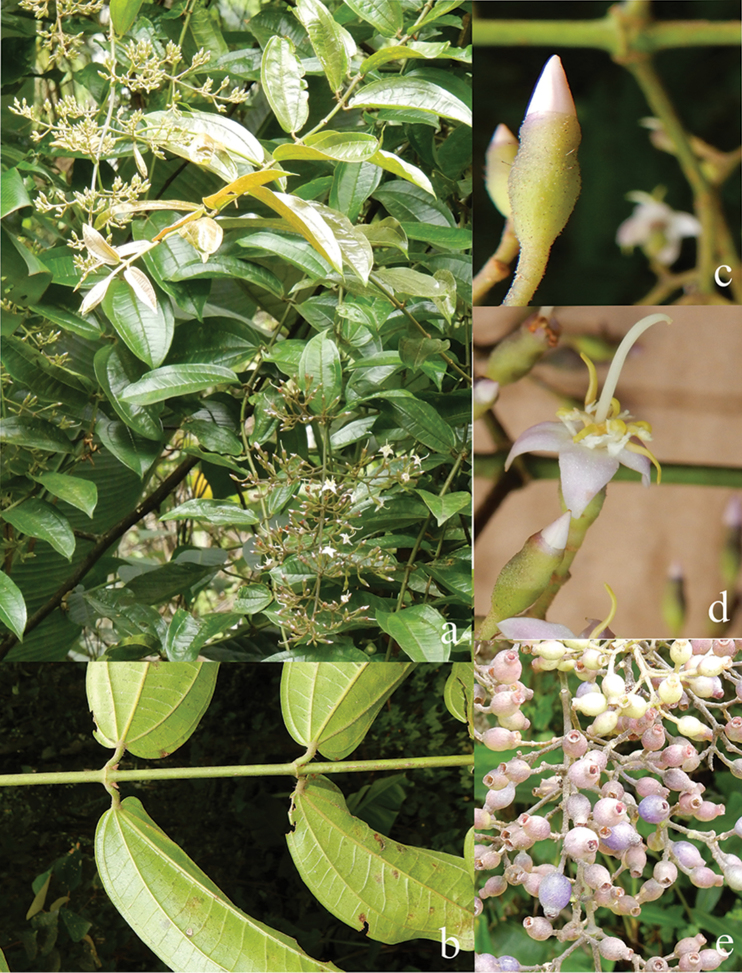
*Dissochaetadivaricata***a** habit **b** branchlet **c** hypanthium **d** flower **e** fruits. Photographs by D. Penneys; voucher: Penneys 2472 (WNC).

**Map 11.**
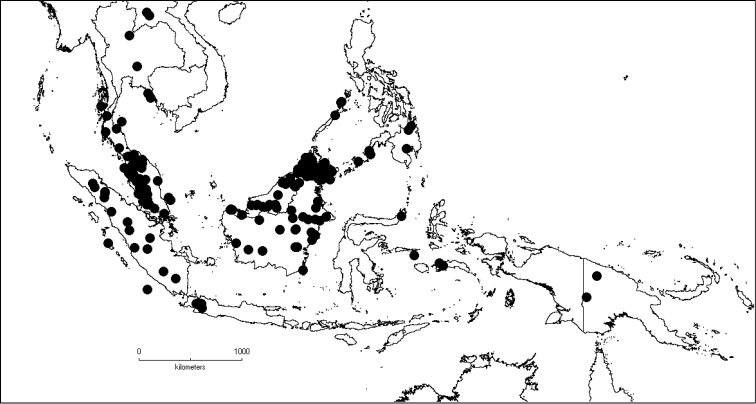
Distribution of *D.divaricata* (●).

##### Description.

Climbing up to 20 m in height. Branchlets terete, 3–5 mm in diameter, glabrous or sparsely to densely covered with brown minute stellate hairs, rarely with dense 2–4 mm long bristle hairs; nodes swollen, with interpetiolar ridge; internodes 3–6 cm long. Leaves: petioles terete, 5–8 mm long, sparsely to densely covered with stellate hairs and often with dense bristle hairs; blades ovate-elliptic, elliptic-oblong to oblong, 6–11 × 2.5–4 cm, chartaceous, base rounded to shallowly cordate, margin entire, apex acuminate, tip 0.5–1 cm long; nervation with 1 pair of lateral nerves and 1 pair of intramarginal nerves; adaxially glabrous, abaxially glabrous or with sparse, stellate hairs, more dense at the midrib. Inflorescences terminal, 9–20 cm long, many-flowered; main axis angular, glabrous or sparsely to densely covered with stellate hairs, often with bristle hairs; primary axes up to 18 cm long with 3–5 nodes, secondary axes up to 4 cm long with 2 or 3 nodes, tertiary axes up to 0.5 cm long with 1 node; bracts ovate or lanceolate, leaf-like, 4–10 × 1–4 mm, covered with stellate hairs, margin ciliate; bracteoles lanceolate or subulate, 2–4 mm long, densely covered with stellate hairs, apex bristly; pedicels glabrous or dorsally covered with stellate hairs, 4–5 mm long in central flowers, 2–3 mm long in lateral flowers. Hypanthium campanulate-angular to suburceolate, 6–7 × 2–3 mm, slightly 8-ridged, sparsely to densely covered with stellate hairs, often with scattered capitate bristles; calyx lobes truncate, ca. 0.5 mm long, apex with 4 minute points, purplish; petal bud conical, 5–9 mm long, acuminate at tip; mature petals ovate, 5–9 × 3–4 mm, reflexed, base clawed, apex acuminate, white or white purplish to purple. stamens 8, unequal, filaments straight; alternipetalous stamens staminodial, with 3–4 mm long filaments, thecae rudimentary, slender, curved, flat, 3–4 mm long, basal crest triangular or hastate, ca. 1 mm long, thin, lateral appendages absent or prolonged from the basal crest, up 2 mm long; oppositipetalous stamens with 4–5 mm long filaments, anthers thick, curved, hook-shaped or S-shaped, thecae 6–10 mm long, yellow, basal appendages bifid, 0.5–1 mm long, connective crest spur-like, triangular, erose, ca. 0.5 mm long. Ovary half as long as hypanthium, apex glabrous; style glabrous, 13–15 mm long, curved at the end, slender, purplish; stigma minute, pappilose; extra-ovarial chambers 4, oppositipetalous, extending almost to the base of the ovary. Fruits urceolate, 4–8 × 2–4 mm, glabrous to puberulous, slightly 8-lined; calyx lobes remnant persistent. Seeds ca. 0.75 mm long.

##### Distribution.

Myanmar, Indochina, Thailand, throughout Malesia (Peninsular Malaysia, Sumatra, Java, Borneo, Philippines, Sulawesi, Moluccas and New Guinea, absent in Lesser Sunda Islands).

##### Ecology and habitat.

Primary and secondary forests, along rivers, roads, on waste lands; usually in the lowlands, rarely up to 1460 m elevation (Veldkamp et al. 1979).

##### Vernacular names.

Peninsular Malaysia: *tuniong utan* (Penang); *sesendok* (Perak). Sumatra: *kedudu akar* (Riau); *kadudu* (Jambi); *sidodo akar* (Palembang). Java: *caluncung areuy* (Sunda). Borneo: *buah apetaah* (Kutai, Kenyah); *akar kemunting* (Iban); *uduk-uduk hutan* (Brunei); *kauelan* (Bagobo).

##### Notes.

1. *Dissochaetadivaricata* is one of the species with the most widespread distribution in the region and it has a wide variation in the indumentum. The specimens vary from glabrous to densely pubescent on the branchlets, abaxially surfaces of the leaf blades, inflorescences axes and the hypanthium. Sometimes they also have scattered bristle hairs on these parts. The variation in bracts and bracteoles ranges from linear to lanceolate, leaf-like. The acuminate tip of the petal bud is a good character for recognising the species and for distinguishing it from other species, e.g. *D.barbata* and *D.conica*.

2. *Melastomadivaricatum* Willd. was reported by Willdenow (1799) from “India Orientali”, although the type is labelled only “Ind.”. At that time, “India Orientali” did not refer to what is now India, but to the entire region now known as South and South-East Asia, the former British East Indies. Veldkamp et al. (1979) and Maxwell (1980a) presumed that the actual source of the type specimen might have been from southern Thailand or further south on the Malay Peninsula.

##### Selected specimens examined.

**MYANMAR.** Lamby Kyum, Jun 1909, C. dÁlleizette 2445 (L). **LAOS. Vientiane**: Vang Vieng, Pu Yang, 250 m, 20 May 2011, J.F. Maxwell 11-25 (L). **THAILAND. Nakhon Nayok**: Khao Yai, 800 m, 24 May 1970, T. Smitinand BKF 46194 (L). **Nakhon Si Thammarat**: Wat Kiri Wang, 100 m, 2 May 1918, A.F.G. Kerr 15580 (BM, K). **Narathiwat**: Khao Tae Saton, 20 Nov 1961, B. Sangkhachand BKF 36947 (K, L, P). **Phang Ngha**: Nai Chong, 100 m, 11 May 1973, R. Geesink & T. Santisuk 5336 (K, L, P). **Pattani**: Bachaw, 50 m, 16 Jul 1923, A.F.G. Kerr 7215 (BK, BM, K, TCD); Betong, 500 m, 11 Mar 1925, A.F.G. Kerr 10075 (BM, K). **Ranong**: 4 Jan 1929, A.F.G. Kerr 16529 (BM, K). **Surat Thani**: Kao Samui, 50 m, 1 Jan 1930, A.F.G. Kerr 17904 (BM, K). **Trang**: Khao Libong, 300 m, 23 Apr 1930, A.F.G. Kerr 19087 (BM, K). **Trat**: Koh Kut Island, 5 Apr 1959, S. Sorensen, K. Larsen & B. Hansen 7175 (L); Koh Chang Island, 3 Aug 1973, R. Geesink & C. Phengkhlai 6262 (L). **Uttaradit**: Ban Phra, 400 m, 18 Apr 1970, T. Smitinand & M. Cheke BKF 46605 (K, L). **Yala**: Ban Rang, 100 m, 24 Apr 1974, R. Geesink & T. Hattink 6394 (K, L); Bannang Sata, Bahng Lahng, 150 m, 12 Nov 1986, J.F. Maxwell 86-905 (L). **MALAYSIA. Johor**: Bukit Bonang, 1900, H.N. Ridley 11103 (K); Pulau Pemanggil, Bukit Durian, 8 Jul 1966, M. Noor & Samsuri 66 (K, L). **Kedah**: Baling, G. Inas, Bukit Iboi, 2 Nov 2007, K. Imin, L.H. Kueh & S.N. Phoon FRI 58596 (K, L). **Malacca**: W. Griffith KD 2288 (BM, K, L, P); 1821, H. Cuming 2259 (BR, G, K, L, P); Tampin, 21 Nov 1916, I.H. Burkill SFN 2297 (K). **Negri Sembilan**: Bukit Tangga, 19 Dec 1920, H.N. Ridley s.n. (BM, K); Jinderam Estate, 90 m, 22 Sep 1957, M. Shah 133 (K, L). **Pahang**: Cameron Highlands, 1210 m, 10 Apr 1937, M. Nur SFN 32600 (L, P); Fraser’s Hill, 19 Jun 1967, J.C. Carrick 1575 (K, L), Richmond, 1280 m, 16 Apr 1955, J.W. Purseglove 4114 (K, L). **Penang**: 1819, Jack 49 (BM, L); Mar 1837, C. Gaudichaud-Beaupré 95 (G, P); Government Hill, A.C. Maingay KD 793 (K, L). **Perak**: Goping, Aug 1880, King’s collector 369 (BM, L, P); Maxwell’s Hill, 42 m, 19 Sep 1949, J. Sinclair & Kiah SFN 38817 (BM, K, L); Sungai Siput, 180 m, 5 Oct 1967, F.S.P. Ng FRI 5739 (K). **Selangor**: Genting-Simpah road, 5 Feb 1965, F.S.P. Ng KEP 99079 (K, L); Kepong, 31 Jan 1968, K. Ogata 10040a (L); Ulu Gombak, 2 Dec 1965, J.C. Carrick 1428 (K, L). **Terengganu**: Kemaman, Bukit Kajang, 152 m, 4 Nov 1935, E.J.H. Corner SFN 30207 (K, L). **Sabah**: Beaufort, Halogilat, Saliwangan, 8 May 1973, Dewol & Karim SAN 77566 (K, L); Keningau, Sepulut, Labang, 18 Oct 1988, Fidilis SAN 125669 (K, L); *Ibid.*, Witti Range, 600 m, 22 Sep 1983, J.H. Beaman 7030 (K, L); Kalabakan, 15 Apr 1982, Fidilis SAN 94758 (K, L); Ranau, Kota Belud, Kelawat, 10 Apr 1950, Kiah SFN 38984 (K, L, P); Lahad Datu, Danum Valley, 7 May 1989, C.E. Ridsdale 1981 (K); Sosopodon, 1066 m, 25 Jun 1963, J. Sinanggul SAN 38278 (K, L); Sandakan, Dec 1921, A.D.E. Elmer 20333 (BM, BO, K, L, P, U); *Ibid.*, M. Ramos BS 1292 (P); *Ibid.*, Kalatuan, 6 Jul 1948, Abdul Rahim A 418 (K, L); Tawau, A.D.E. Elmer 21187 (BM, BO, K, L, P, U). **Sarawak**: O. Beccari PB 2186 (K); Bakelalan, W.M.A. Brooke 10391 (BM, L); Balleh, Ulu Mujong, 250 m, 20 Mar 1964, P.S. Ashton S.13984 (K, L); Baram, C. Hose 181 (BM, K, L, P); Kapit, Bukit Raya, 457 m, 16 Oct 1965, Jugah ak Kudi S.23880 (K, L); Miri, Kelabit Highlands, 950 m, 1 Mar 1995, Christensen & Apu 717 (K); Gat, Upper Rejang River, 2 Jul 1929, J. Clemens & M.S. Clemens 21568 (BM, K, L, P); Sibu, Rejang, Aug 1893, G.D. Haviland 3143 (BM, K); Sarikei, 6 Jul 1954, W.M.A. Brooke 8757 (BM, L). **SINGAPORE.** W. Jack s.n. (L). **INDONESIA. Aceh**: Gayoland, Gajah-Blangkajeren, 1400 m, 27 Feb 1937, C.G.G.J. van Steenis 9411 (BO, K, L); Leuser Mts., Lau Ketambe, 400 m, 4 Jun 1972, W.J.J.O. de Wilde & B.E.E. de Wilde-Duyfjes 12568 (BO, K, L). **Bengkulu**: Enggano Island, Malakoni, Kuala Besar, 50 m, 22 Apr 2015, M. Ardiyani et al. E167 (BO). **Jambi**: Batang Sungai, 200 m, Sep 1925, O. Posthumus 937 (BO, L). **Lampung**: Gunung Basin, 20 Sep 1845, H. Zollinger 3044 (BM, BO, BR, G, MPU, P). **Mentawai Islands**: Siberut, 11 Sep 1924, C. Boden-Kloss SFN 13082 (BO, K). **North Sumatra**: Brastagi, 1250 m, 21 Feb 1932, W.N. Bangham & C.N. Bangham 1140 (K); Sibolangit, 500 m, 18 Dec 1927, J.A. Lörzing 12317 (BO, K, L); Tapanuli, Between Sidikalang and Pongkolan, 1200 m, 27 Mar 1954, A.H.G. Alston 14816 (BM, L). **Riau**: Tigapuluh Mts., Talanglakat, 100 m, 4 Nov 1988, J.S. Burley & Tukirin 1084 (BO, K, L). **South Sumatra**: Enim, Pandan Ulu, J.E. Teijsmann HB 3634 (BO). **West Sumatra**: Lima Puluh Kota, Harau Valley, Sarasah Bonta, 500 m, 11 Sep 2017, A. Kartonegoro 1069 (BO, L). **West Java**: Bogor, Mount Paniisan, 700 m, 9 Dec 1923, R.C. Bakhuizen van den Brink 6165 (BM, BO, K, L); Mount Salak, C.L. Blume s.n. (K, L). **Central Kalimantan**: Sampit River, Kuala Kwayan, Permantang, 50 m, 25 Jan 1954, A.H.G. Alston 13234 (BM, BO); Barito Ulu, 8 Jun 1990, C.E. Ridsdale PBU 456 (BO, L); Nanga Buli, Sungai Buluh, 250 m, 26 Feb 1984, C. Hansen 1212 (L). **East Kalimantan**: East Kutai, Sg. Menubar, 5 Jun 1951, A.J.G.H. Kostermans 4961 (BO, K, L); *Ibid.*, Muara Ancalong, Long Lees, 100 m, 6 Mar 1978, H. Wiriadinata 1151 (BO, K, L); West Kutai, Hikam Batu Beng, 80 m, 28 Jul 1925, F.H. Endert 2270 (BO, K, L); Tanjung Redeb, Birang River, 23 Oct 1963, A.J.G.H. Kostermans 21644 (BO, K, L); Berau, Mt. Menyapa, Kelai River, 19 Oct 1963, A.J.G.H. Kostermans 21364 (BO, K, L); Gunung Gadut, 31 Mar 1908, H.J.P. Winkler 1752 (BM, K); Samarinda, Loa Haur, 40 m, 16 May 1952, A.J.G.H. Kostermans 6965 (BO, K, L). **North Kalimantan**: Krayan, Long Bawan, 1000 m, 16 Jul 1981, K. Ueda & D. Darnaedy B-8515 (BO, L). **South Kalimantan**: Salimohi, Simpokok, 15 Jul 1908, H.J.P. Winkler 2970 (BM); Pulau Laut, 100 m, 6 Nov 1928, D.F. van Slooten 2282 (BO, K, L). **West Kalimantan**: Danau Sentarum, Semujan Hill, 4 Jul 1986, W. Giesen 69 (K, L). **North Sulawesi**: Gurupahi, 600 m, 19 Mar 1917, J. Kaudern 6 (L). **Moluccas**: Ceram, Piru, 400 m, 16 Nov 1918, L.M.R. Rutten 1904 (BO, L, U); Sula, Mount Berberi, Atje 318 (BO, L). **PHILIPPINES. Mindanao**: Agusan, Cabadbaran (Mt. Urdaneta), Jul 1912, A.D.E. Elmer 13352 (BISH, BM, BO, E, GH, K, L, MO, NY, P, PNH, U); Davao, Mt. McKinley, 640 m, 1 Oct 1946, G.E. Edano PNH 1008 (PNH). **Palawan**: Pagdanan, Ibangley, 40 m, 21 Apr 1984, A. Podzorski SMHI 906 (K, L). **Sulu**: Jolo, Sep 1924, M. Ramos & G.E. Edano BS 44461 (K, L). **PAPUA NEW GUINEA. Sepik**: C.L. Ledermann 6654 (L). **Western District**: Kiunga, 30 m, 6 Aug 1971, H. Streimann LAE 51727 (L).

#### 18. Dissochaeta fallax

Taxon classificationPlantaeMyrtalesMelastomataceae

(Jack) Blume, Flora 14: 493. 1831.

Fig. 13 , Map 12

Melastoma fallax Jack, Trans. Linn. Soc. London 14: 13. 1823.
Melastoma reinwardtianum Blume, Bijdr. Fl. Ned. Ind. 17: 1069. 1826. Type: Indonesia. West Java, H. Kuhl & J.C. van Hasselt s.n. (lectotype, designated by Bakhuizen van den Brink *f.* 1943, pg. 119: L [L0537272]!; isolectotypes L [L0537269, L0537270]!).
Melastoma leprosum Blume, Bijdr. Fl. Ned. Ind 17: 1068. 1826. *p.p.*, excl. type
Dissochaeta reticulata Blume, Flora 14: 499. 1831. Type: Indonesia. Java, C.L. Blume s.n. (lectotype, designated by Bakhuizen van den Brink *f.* 1943, pg. 144: L [L0008896]!; isolectotypes: L [L0008897]!, P [P05283565]!).
Dissochaeta ligulata Blume, Mus. Bot. 1(3): 35. 1849. Type: Indonesia. Java, F.W. Junghuhn s.n. (lectotype, designated by Bakhuizen van den Brink *f.* 1943, pg. 144: L [L0008898]!).
Omphalopus fallax (Jack) Naudin, Ann. Sci. Nat., Bot. sér. 3, 15: 277, pl. 4, fig. 5. 1851.
Omphalopus reticulatus (Blume) Naudin, Ann. Sci. Nat., Bot. sér. 3, 15: 278. 1851.
Dissochaeta diepenhorstii Miq., Fl. Ned. Ind., Eerste Bijv. 2: 317. 1861. Type: Indonesia. West Sumatra: Priaman, H. Diepenhorst HB 1323 (lectotype, designated by Bakhuizen van den Brink *f.* 1943, pg. 119: U [0004007]!; isolectotype: BO [BO1865969]!).
Anplectrum ligulatum (Blume) Triana, Trans. Linn. Soc. London 28: 85. 1872.
Dissochaeta inappendiculata auct. non Blume: Triana, Trans. Linn. Soc. London 28: 84. 1872. *p.p.*, excl. type.
Diplectria ligulata (Blume) Kuntze, Revis. Gen. Pl. 1: 246. 1891.
Dissochaeta celebica auct. non. Blume: Baker *f.*, J. Bot. 62 (Suppl.): 40. 1924. *p.p.*, excl. type.
Dissochaeta leprosa auct. non. Blume: Baker *f.*, J. Bot. 62 (Suppl.): 40. 1924. *p.p.*, excl. type.
Dissochaeta reinwardtiana (Blume) Hochr., Candollea 2: 472. 1925.
Omphalopus fallax (Jack) Naudin var. novoguineensis Mansf., Bot. Jahrb. Syst. 60: 113. 1925. Type: Papua New Guinea. Kaiser Wilhelmsland, Kani-Gebirges, 1000 m, 7 Jan 1908, R.F.F. Schlechter 15159 (lectotype, designated here: NY [NY00229576, image seen]!).
Neodissochaeta reticulata (Blume) Bakh.*f.*, Contr. Melastom.: 143. 1943.
Dissochaeta velutina Blume var. reticulata (Blume) J.F.Maxwell, Gard. Bull. Singapore 33: 321. 1980.

##### Type.

Indonesia. Sumatra, *Jack s.n.* (lost); Indonesia. Bengkulu: Ajer Angat, G. Kaba, H.O. Forbes 2882a (neotype, designated by Kartonegoro and Veldkamp 2010, pg. 132: L [L0822678]!; isoneotype: BM!).

**Figure 13.**
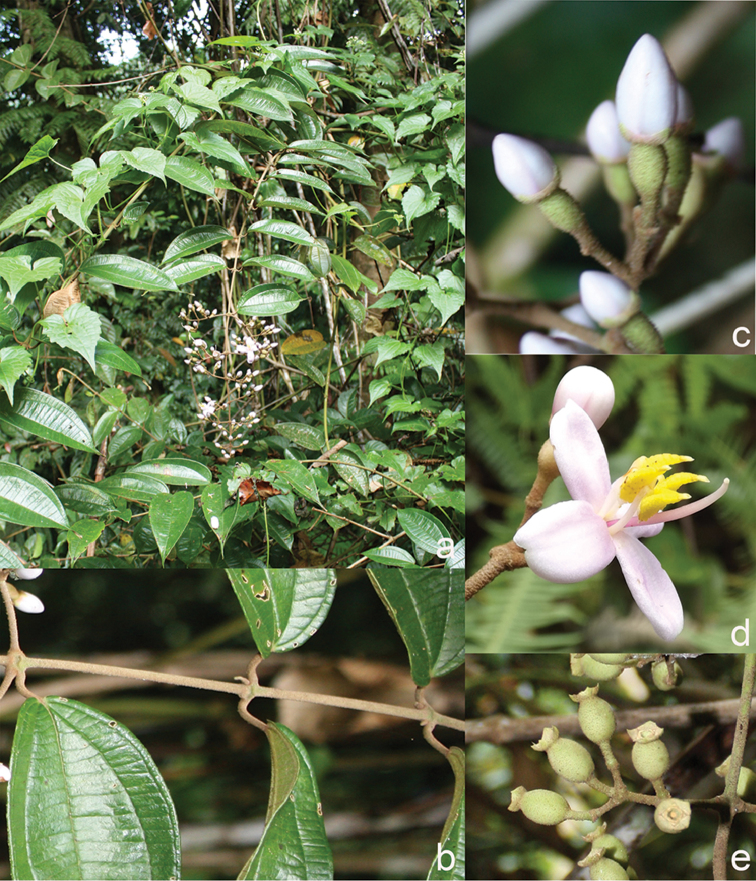
*Dissochaetafallax*. **a** habit **b** branchlet **c** hypanthium **d** Flower **e** immature fruits. Photographs by A. Kartonegoro; vouchers: Kartonegoro 1106 (BO, L).

**Map 12.**
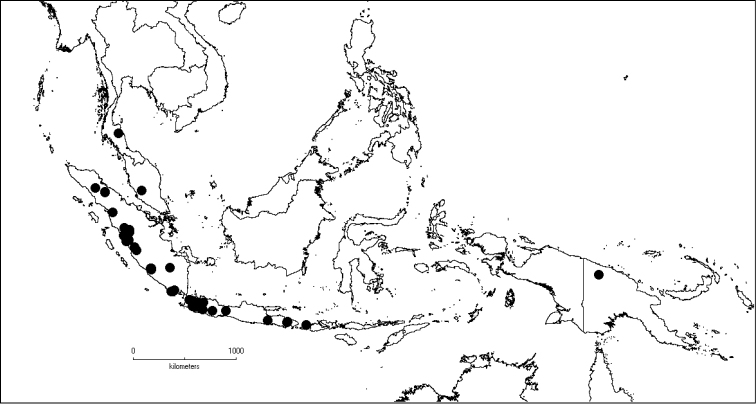
Distribution of *D.fallax* (●).

##### Description.

Climbing up to 25 m in height. Branchlets terete or subquadrangular, 3–6 mm in diameter, puberulous to brown stellate-furfuraceous; nodes swollen, with raised interpetiolar ridge; internodes 3.5–7 cm long. Leaves: petioles terete, 8–20 mm long, puberulous to densely stellate-furfuraceous; blades ovate to ovate-oblong, 6–15.5 × 3–7 cm, membranous, base cordate or subcordate, rarely rounded, margin entire, apex acuminate, tip 0.5–1 cm long; nervation with 1 pair of lateral nerves and 1 pair of intramarginal nerves; adaxially glabrous, light green, glossy, abaxially densely brown stellate-furfuraceous. Inflorescences terminal and in the upper leaf axils, up to 35 cm long, many-flowered; main axis densely stellate-furfuraceous; peduncle up to 8 cm long; primary axes up to 15 cm long with 4 or 5 nodes, secondary axes 3–3.5 cm long with 1–3 nodes, tertiary axes up to 5 mm long with 1 node or undeveloped; bracts linear, 2–3 mm long, densely brown furfuraceous, caducous; bracteoles linear, minute, ca. 1.5 mm long, brown furfuraceous, caducous; pedicels densely stellate-furfuraceous, 3–5 mm long in central flowers, 1–2 mm long in lateral flowers. Hypanthium urceolate, 3–6 × 2–3 mm, green, densely stellate-furfuraceous; calyx lobes truncate with triangular tips, 0.5–1(–2.5) mm long, widened, glabrous or with stellate hairs; petals bud rounded or rarely subconical, 4–10 mm long, with a rounded tip; mature petals obovate, 7–8 × 2–4 mm, not reflexed, base clawed, apex rounded, glabrous or inside with appressed hairs at the base, white pinkish or pink. Stamens 4 or 8, equal or subequal when 8, all fertile, filaments bright white, straight, curved at the end; alternipetalous stamens with 4–5 mm long filaments, anthers ovate or lanceolate, when mature falcate, straight, medifixed, thecae 3–6.5 mm long, bright yellow, beaked, pedoconnective not developed, basal crests triangular, orbicular or ligular, 1–3 mm long, attached to the attachment of the filament, white, lateral appendages absent; the oppositipetalous stamens smaller, with 2–3 mm long filaments, anthers oblong, oblong-lanceolate, medifixed, thecae 2–4 mm long, tessellate-reticulate, yellow, basal crests ligular, erect or sometimes tapering horizontally inward to the anther, 1–1.5 mm long, lateral appendages absent. Ovary half or ⅔ of hypanthium in length, apex pubescent; style 7–15 mm long, curved at the end, glabrous, white with pinkish base; stigma minute; extra-ovarial chambers absent or shallow. Fruits urceolate or subglobose, 4–8(–12) × 4–5(–7) mm, stellate-puberulous, calyx lobe remnants persistent, 1–2 mm long. Seeds ca. 0.5 mm long.

##### Distribution.

Thailand (Southern Peninsula), Peninsular Malaysia, Sumatra, Java, Lesser Sunda Islands (Bali, Lombok) and New Guinea (Papua New Guinea).

##### Ecology and habitat.

Primary or secondary submontane forest, rarely near a crater or at the edge of a forest, 400–1600 m elevation.

##### Vernacular names.

Sumatra: *air wangian* (Minang); *akar gameh* (Pariaman); *gedang serian* (Lampung). Java: *harendong areuy, harendong beureum, harendong oyot* (Sunda). Lesser Sunda: *priyato* (Bali).

##### Notes.

1. *Dissochaetafallax* is easily distinguished by its 4 or 8 stamens with tessellate-reticulate thecae and medifixed anthers. The hypanthium is suburceolate, slender and smaller than the petals in bud, which are usually rounded. This species is common in West Malesia (Sumatra and Java), but so far not found in Borneo and further east except for one collection from New Guinea. The stamens make *Dissochaetafallax* so different from the other species that it has long been regarded as a distinct genus, *Omphalopus* (Naudin 1851, Miquel 1855, Triana 1872, Cogniaux 1891, Bakhuizen van den Brink *f.* 1943). We regard these differences only as infrageneric variation and we follow previous authors (Blume 1831, Maxwell 1980b, Clausing and Renner 2001, Kartonegoro and Veldkamp 2010) to include it in *Dissochaeta*. Future phylogenetic analyses may point out the true relationship of this species.

2. The correct identification of *D.reticulata* is problematic. Naudin (1851) and Miquel (1855) correctly considered this species to be part of former *Omphalopus*, together with *O.fallax* based on its stamen characters. Blume (1831), Veldkamp (1979), Kartonegoro and Veldkamp (2010) regarded it as a distinct species in *Dissochaeta* based on 8 subequal fertile stamens, in which it is similar to *D.inappendiculata*. Bakhuizen van den Brink *f.* (1943, 1964) placed this species under *Neodissochaeta*. On the other hand, Maxwell (1980b) erroneously considered the species to be a variety of *D.velutina* (a synonym of *D.vacillans*). Like *D.fallax*, the type of *D.reticulata* also shows the stamens to have tessellate-reticulate thecae, medifixed stamens and an inappendiculate crest. Therefore, the name is here synonymised with *D.fallax*, whereby the variation in the number of stamens became 4 or 8.

##### Selected specimens examined.

**THAILAND. Nakhon Si Thammarat**: Khao Luang, 750 m, 19 May 1968, C.F. van Beusekom & C. Phengkhlai 880 (K, L, P). **MALAYSIA. Selangor**: Genting Highlands, Gunong Ulu Kali, 1500 m, 9 Apr 1978, J.F. Maxwell 78-81 (L). **INDONESIA. Aceh**: Gayoland, between Kampong Kapi and Kampong Aunan, 1100 m, 21 Mar 1937, C.G.G.J. van Steenis 9972 (BO). **Bengkulu**: Air Hangat, Bukit Kaba, H.O. Forbes 2882a (BM, L); Suban Ayam, 1200 m, 12 Jul 1916, Ajoeb 350 (BO); Balai, 500 m, 13 Jan 1931, C.N.A. de Voogd 581 (BO, L). **Jambi**: Kerinci, Siolak Deras, 915 m, 18 Mar 1914, H.C. Robinson & C. Boden-Kloss s.n. (BM, K). **Lampung**: Sukaraja, 28 Aug 1915, P.J.S. Cramer 107 (BO); Mt. Tanggamus, 1600 m, 2 May 1968, M. Jacobs 8213 (L). **North Sumatra**: Bandar Baru, Mt. Sibayak, 900 m, 9 Oct 1928, J.A. Lörzing 14075 (BO, L); Sibolangit, 12 Sep 1920, J.A. Lörzing 7351a (BO); Padang Sidempuan, Mt. Lubuk Raya, 1000 m, 13 Apr 1978, Maskuri 282 (BO, L). **South Sumatra**: Muara Dua, Tenang, 700 m, 10 Jan 1930, C.N.A. de Voogd 556 (BO). **West Sumatra**: Pariaman, H. Diepenhorst HB 1323 (BO, U); Mt. Malintang, 1 Aug 1918, H.A.B. Bünnemeijer 4225 (BO, K, L, U); Mt. Merapi, 1250 m, 13 Sep 1918, H.A.B. Bünnemeijer 4514 (BO, K, L, U); Lubuk Sikaping, Mt. Gadang, 700 m, 15 Jun 1953, J. van Borssum-Waalkes 1893 (BO, L); Lima Puluh Kota, Kelok Sembilan, 700 m, 20 Dec 1987, M. Hotta & H. Okada 1637 (BO); *Ibid.*, Mt. Sago, Ladang Laweh, 900 m, 28 Jul 1957, W. Meijer 7245 (L); Solok, Mount Talang, 1250 m, 2 Oct 1988, M. Hotta & H. Nagamasu 12 (BO, L). **Banten**: Between Bayah & Sangkop, 600 m, 20 Jun 1911, C.A. Backer 1722 (BO); Pasir Orai, H.O. Forbes 460 (BM, BO); Mt. Karang, Galusur, 700 m, 31 Jun 1912, S.H. Koorders 40738β (BO). **Central Java**: Purbalingga, Mt. Slamet, 1300 m, 13 Mar 2004, W.S. Hoover et al. Deden-36 (BO). **East Java**: Lumajang, Sumber Mujur, Mar 1928, Adm. Ondern. Soember Moedjoer s.n. (BO, L, U). **West Java**: Mt. Paniisan, 600 m, 28 Oct 1928, C.G.G.J. van Steenis 2300 (BO, L); Leuwiliang, Cianten, 900 m, 30 Aug 1918, C.A. Backer 25698 (BO); Mt. Menapa, 600 m, 18 Dec 1940, C.G.G.J. van Steenis 17373 (BO, K, L); Mt. Salak, Kampong Babojong, 700 m, 18 Sep 1896, S.H. Koorders 24270β (BO, K, L); Mt. Halimun, Malasari, 1055 m, 10 Oct 2017, A. Kartonegoro 1106 (BO, L); Cianjur, Cibeber, Cidadap, 1000 m, R.C. Bakhuizen van den Brink 2769 (BO); *Ibid.*, Takokak, S.H. Koorders 33358β (BO); Tasikmalaya, Singaparna, Mt. Galunggung, 900 m, 13 Aug 1913, C.A. Backer 8619 (BO). **Bali**: Jembrana, Mt. Mesehe, 500 m, 18 May 2013, A. Kartonegoro et al. 737 (BO); Mt. Pala 495 m, 5 Sep 1918, Sarip 219 (BO, L). **West Nusa Tenggara**: Lombok, Mt. Rinjani, Jeruk Manis waterfall, 904 m, 16 Feb 2005, H. Azuma et al. A259 (BO). **PAPUA NEW GUINEA. Sepik**: Kaiser Wilhelmsland, Kani Mountains, 1000 m, 7 Jan 1908, R.F.F. Schlechter 15159 (NY).

#### 19. Dissochaeta floccosa

Taxon classificationPlantaeMyrtalesMelastomataceae

(J.F.Maxwell) Karton. comb. nov.

urn:lsid:ipni.org:names:60476835-2

Map 13

##### Basionym.

DissochaetarostrataKorth.var.floccosa J.F.Maxwell, Gard. Bull. Singapore 33: 319, fig. 5. 1980.

##### Type.

Indonesia. West Sumatra: Pajakumbuh, Pakan Raba, Aer nan Dingin, 600 m elev., 1 Sep 1957, S. Maradjo 350 (holotype: L [L0537273]!; isotypes: L [L0537271]!, PNH [PNH59964, image seen]!, SING *n.v.*).

**Map 13.**
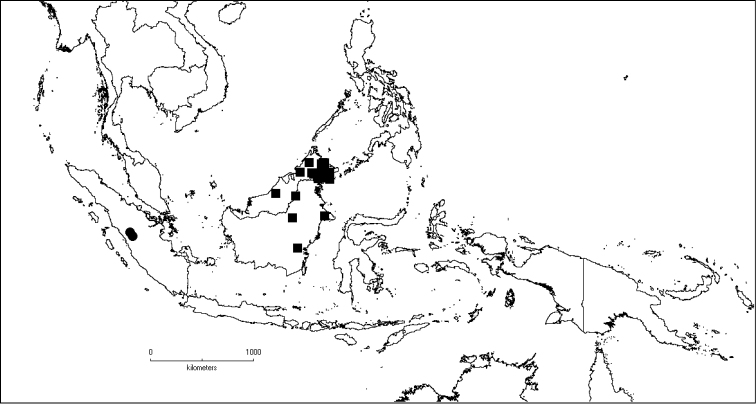
Distribution of *D.floccosa* (●) and D.glabravar.glabra (■).

##### Description.

Branchlets terete, 5–6 mm in diameter, floccose, covered with dense stellate pubescent hairs and with 1–2 mm long capitate bristle hairs; nodes swollen, with interpetiolar ridge; internodes 12–14 cm long. Leaves: petioles terete, ca. 10 mm long, floccose, densely covered with pubescent hairs and with 1–2 mm long brown bristle hairs; blades ovate or ovate-elliptic, 17–20 × 7–9.5 cm, subcoriaceous, base emarginate to slightly cordate, margin entire, apex acuminate, tip 2–3 cm long; nervation with 2 pairs of lateral nerves and 1 pair of intramarginal nerves; adaxially glabrous, abaxially densely floccose, covered with pubescent hairs. Inflorescences terminal, up to 40 cm long, many-flowered; main axis angular, floccose, densely covered with pubescent hairs and 1–2 mm long capitate bristle hairs; primary axes up to 36 cm long with 5 or 6 nodes, secondary axes up to 6 cm long with 2 or 3 nodes, tertiary axes up to 2 cm long with 1 or 2 nodes, quarternary axes when developed up to 1 cm long with 1 node; bracts linear, 10–13 mm long, floccose, densely covered with pubescent hairs and with 1–2 mm long capitate bristle hairs; bracteoles subulate, 5–10 mm long, floccose, densely covered with pubescent hairs and with 1–2 mm long capitate bristle hairs; pedicels floccose, densely covered with pubescent hairs and with capitate bristle hairs, 6–7 mm long in central flowers, 2–3 mm long in lateral flowers. Hypanthium tubular, 7–9 × 3–4 mm, floccose, densely covered with pubescent hairs and with capitate bristle hairs; calyx lobes linear-lanceolate or triangular, 7–11 mm long, floccose, densely covered with pubescent hairs and capitate bristle hairs, base truncate, apex acute; petal bud conical, 3–5 mm long; mature petals not seen. Stamens 8, subequal, filaments curved sideways; alternipetalous stamens with 8–9 mm long filaments, anthers slender, curved, sickle-shaped, thecae 14–15 mm long, pedoconnective 2–3 mm long, basal crest minute, triangular, ca. 1 mm long, lateral appendages paired, filiform, 2–4 mm long; oppositipetalous stamens with ca. 8 mm long filaments, anthers S-shaped, thecae 12–14 mm long, basal crest with minute auricles, ca. 0.5 mm long, lateral appendages paired auricles, 1–1.5 mm long. Ovary ¾ of hypanthium in length, apex floccose; style glabrous, in bud ca. 15 mm long; stigma minute; extra-ovarial chambers 8, extending to the base of the ovary. Fruits urceolate, ca. 10 × 5–6 mm, floccose, densely covered with pubescent hairs and capitate bristle hairs; calyx lobes remnant persistent, 8–11 mm long, reflexed. Seeds ca. 0.5 mm long.

##### Distribution.

Sumatra (West).

##### Ecology and habitat.

Lowland forest at 300–600 m elevation.

##### Note.

*Dissochaetafloccosa* is known only from 2 collections. This species closely resembles *D.horrida* in having long, linear-lanceolate calyx lobes of up to 11 mm long and similar number and shape of the stamens, but differs in having a floccose indumentum all over with shorter bristle hairs.

##### Specimen examined.

**INDONESIA. West Sumatra**: Payakumbuh, Pakan Raba, 600 m elev., 1 Sep 1957, S. Maradjo 350 (L, PNH); Sawahlunto Sijunjung, Kulampi, 300 m, 21 Apr 2000, Erlo 32 (ANDA).

#### 20. Dissochaeta glabra

Taxon classificationPlantaeMyrtalesMelastomataceae

Merr., J. Straits Branch Roy. Asiat. Soc. 76: 101. 1917.

Diplectria glabra (Merr.) M.P.Nayar in Veldkamp *et al*., Blumea 24: 421, fig. 4B. 1979.
Diplectria glabra (Merr.) M.P.Nayar ssp. glabra : Veldkamp *et al*., Blumea 24: 421, fig. 4B. 1979.
Diplectria glabra (Merr.) M.P.Nayar var. glabra : J.F.Maxwell, Gard. Bull. Singapore 33: 313. 1980.

##### Type.

Malaysia. Sabah: Kalabakan, Pinajas River, 20 m elev., 8 Oct 1916, A. Villamil 242 (lectotype, designated here: PNH [PNH32349, image seen]!; isolectotypes: K!, US [US00120530, image seen]!).

##### Description.

Climbing up to 15 m in height. Branchlets terete, 4–5 mm in diameter, glabrous; nodes swollen, with prominent annular crest-like interpetiolar ridge, often with bristle hairs; internodes 6–8.5 cm long. Leaves: petioles terete, 7–10 mm long, furfuraceous and dorsally with bristle hairs; blades ovate to ovate-oblong, 9–16 × 4.5–8 cm, subcoriaceous, base rounded to shallowly subcordate, margin entire, apex acuminate, tip 0.5–0.8 cm long; nervation with 1 or 2 pairs of lateral nerves and 1 pair of intramarginal nerves; adaxially glabrous, abaxially glabrous, basally with a pair of glandular patches. Inflorescences terminal, up to 90 cm long, many-flowered; main axis angular, glabrous or sparsely covered with stellate hairs; primary axes up to 80 cm long with 7–10 nodes, secondary axes 8–20 cm long with 5–7 nodes, tertiary axes up to 6 cm long with 1–3 nodes, quarternary axes if developed 1–3 cm long with 1 or 2 nodes, quinternary axes when developed up to 0.5 cm with 1 node; bracts linear, 4–5 mm long, caducous, stellate puberulous; bracteoles subulate, ca. 1 mm long, densely covered with stellate hairs; pedicels stellate puberulous, 3–5 mm long in central flowers, 1–2 mm long in lateral flowers. Hypanthium cyathiform-tubular, 4–6 × 2–3.5 mm, glabrous or sparsely covered with stellate hairs; calyx lobes truncate, ca. 0.5 mm long, apex with 4 minute points, purplish; petal bud conical, 1–5 mm long; mature petals suborbicular, 3–6 × 3–4.5 mm, reflexed, base clawed, apex acute, white purplish or purple. Stamens 8, unequal, filaments flattened, straight; alternipetalous stamens staminodial, with 3–5 mm long filaments, anthers rudimentary, slender, terete, thecae 0.5–2 mm long, basal crest triangular, 0.75–2 mm long, thin, apex acute, base emarginate or hastate, lateral appendages prolonged from the theca vestige, paired, filiform, 3–6 mm long; oppositipetalous stamens with 4–6 mm long filaments, anthers thick, curved, hook-shaped or S-shaped, thecae 5–6 mm long, yellow, basal crest triangular, 0.3–2 mm long, erose to bifid, lateral appendages ligular with bifid apex, 0.5–1 mm long. Ovary half to ¾ of hypanthium in length, apex glabrous; style 8–12 mm long, curved at the end, slender, glabrous; stigma minute, capitate; extra-ovarial chambers 4, oppositipetalous, extending from ⅓ to the middle of the ovary. Fruits subglobose to urceolate, 4–7 × 4–5 mm, glabrous, slightly 8-lined; calyx lobe remnants persistent. Seeds ca. 0.5 mm long.

##### Distribution.

Borneo.

##### Note.

Within the genus, *D.glabra* has the longest and most robust inflorescences (up to 90 cm long) with up to 5 degrees of ramifications, but it has smaller flowers. Due to the flowers and stamens, this species closely resembles *D.conica* and *D.papuana*. The glabrous appearance and the glandular patches at the leaf base below are similar to *D.beccariana*, which is also endemic to Borneo, but differs in the shape of the alternipetalous stamens.

#### Key to varieties of *D.glabra*

**Table:** 

1	Hypanthium ca. 4 × 2 mm, glabrous or sparsely covered with stellate hairs; petal bud 1–1.5 mm long; mature petals 3–4 × 3–3.5 mm, white; fruits subglobose to urceolate, 4–5 × ca. 4 mm	**var. glabra**
–	Hypanthium 5–6 × ca. 3.5 mm, glabrous; petal bud 4–5 mm long; mature petals 5–6 × ca. 4.5 mm, violet or pink; fruits urceolate, 6–7 × ca. 5 mm	**var. kinabaluensis**

#### 20.1. Dissochaeta glabra Merr. var. glabra

Taxon classificationPlantaeMyrtalesMelastomataceae

Fig. 14 , Map 13

##### Description.

Hypanthium ca. 4 × 2 mm, glabrous or sparsely covered with stellate hairs, dark green; petal bud conical, 1–1.5 mm long; mature petals suborbicular, 3–4 × 3–3.5 mm, reflexed, white purplish or purple, base clawed, apex acute. Stamens 8, unequal, filaments straight; alternipetalous stamens staminodial, with ca. 3 mm long filaments, thecae ca. 0.5 mm long, basal crest triangular, ca. 0.75 mm long, thin, base emarginate or hastate, apex acute, lateral appendages prolonged from the locule vestige, paired, filiform, 3–4 mm long; oppositipetalous stamens with 4–5 mm long filaments, thecae 5–6 mm long, yellow or creamy, basal crest triangular, ca. 0.3 mm long, erose to bifid, lateral appendages ligular with bifid apex, ca. 0.5 mm long. Ovary half to ¾ of hypanthium in length, apex glabrous; style glabrous, 8–10 mm long, curved at the end, slender; stigma minute, capitate; extra-ovarial chambers extending to half of the ovary. Fruits subglobose to urceolate, 4–5 × ca. 4 mm, glabrous.

**Figure 14.**
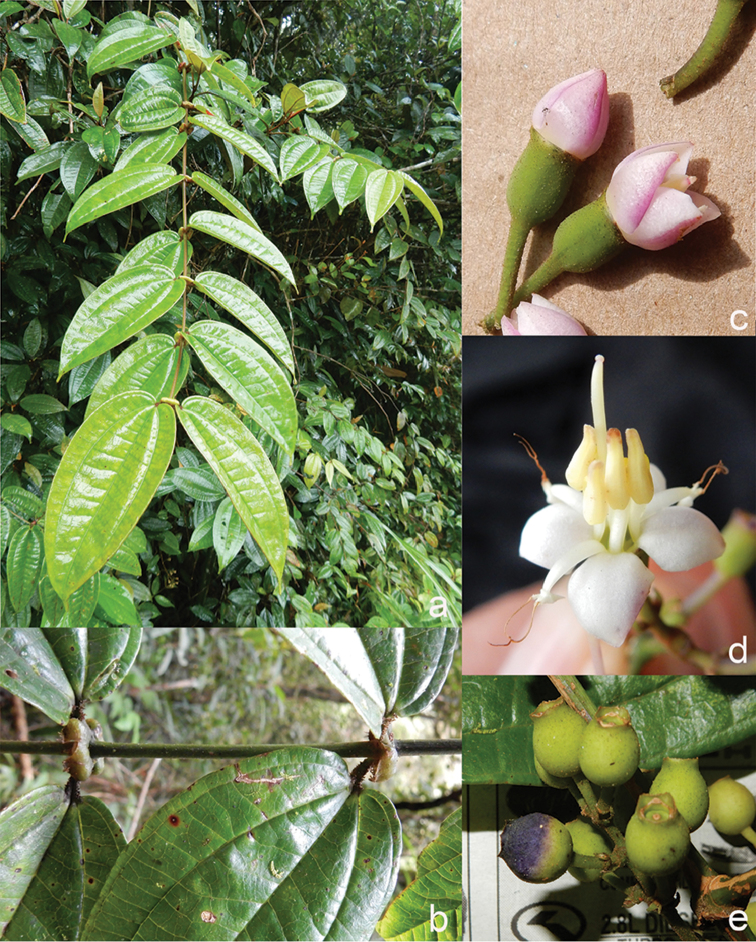
Dissochaetaglabravar.glabra**a** habit **b** branchlet **c** hypanthium **d** flower **e** fruits. Photographs by D. Penneys; vouchers: Penneys 2446 (WNC), Penneys 2474 (WNC) & Penneys 2487 (WNC).

##### Distribution.

Borneo.

##### Ecology and habitat.

Lowland mixed dipterocarp forest to lower montane forest, in open places at 20–1200 m elevation.

##### Specimens examined.

**MALAYSIA. Sabah**: Kalabakan, Pinajas River, 20 m, 8 Oct 1916, A. Villamil 242 (K, PNH, US); *Ibid.*, Ulu Sungai Kalabakan, 19 May 1984, Fidilis & Martin SAN 103669 (K, L); Ibid., Gunong Rara, 21 Apr 1972, G. Shea SAN 75633 (K, L); Lahad Datu, Danum Valley, 12 Jun 1986, E.J.F. Campbell et al. SAN 112059 (L); *Ibid.*, 7 May 1989, C.E. Ridsdale 1961 (K, L); *Ibid.*, 238 m, 6 Jul 2006, S. Suzana et al. SAN 147687 (K, L); *Ibid.*, Silabukan, 183 m, 21 Apr 1967, J. Sinanggul SAN 58046 (K, L); *Ibid.*, Ulu Segama, 170 m, 25 Feb 1986, P.J. Edwards 2111 (K); *Ibid.*, 200 m, 28 Feb 1985, G. Argent et al. 108277 (K, L); Lamag, Inarat, Gunong Lotong, 1000 m, 16 Aug 1976, Saikeh SAN 83210 (K); Nabawan, Gunung Lotong, Meliah Basin, 19 Apr 1988, L. Madani SAN 124418 (K); Ranau, Sungai Nabutan, 23 Mar 1982, Joseph SAN 94559 (K, L); Sandakan, Segaliud Lokan, 106 m, 30 May 1963, Banang SAN 36928 (K, L); *Ibid.*, Gadong Camp, 58 m, 4 Apr 1963, James SAN 35395 (K, L); *Ibid.*, Gomantong, 17 Apr 1970, Rusonkhan SAN 66574 (K); *Ibid.*, Kabili-Sepilok, 4 Jun 1937, Enggoh 7246 (K); *Ibid.*, Sepilok, 7 Apr 1954, D.D. Wood A 2987 (K, L); Tenom, Mandalom, 27 Aug 1987, Asik Mantor SAN 120365 (K, L); Tawau, A.D.E. Elmer 20794 (BM, L, P, PNH, U), St. Lucia, Pinayas, 60 m, 8 May 1940, P. Orolfo 8 (K, L). **Sarawak**: Bintulu, Tubau, Ulu Jejalong, Bukit Sekiwa, 300 m, 2 Sep 1986, Abang Mochtar S.53947 (AAU, L). **INDONESIA. East Kalimantan**: West Kutai, Hikam Batu Beng, 80 m, 28 Jul 1925, F.H. Endert 2275 (BO, L); Sangkulirang, Babi Jolong, 40 m, 3 Jun 1937, Aet 599 (BO). **North Kalimantan**: Malinau, 20 m, 2 Jul 1981, R. Geesink 8926 (L). **South Kalimantan**: Muara Uya, Jaro Dam, 80 m, 17 Nov 1971, E.F. de Vogel 881 (K, L).

#### 20.2. Dissochaeta glabra Merr. var. kinabaluensis

Taxon classificationPlantaeMyrtalesMelastomataceae

(Veldkamp) Karton. comb. nov.

urn:lsid:ipni.org:names:60476836-2

Fig. 15 , Map 14

Diplectria glabra (Merr.) M.P.Nayar var. kinabaluensis (Veldkamp) J.F.Maxwell, Gard. Bull. Singapore 33: 313. 1980.

##### Basionym.

Diplectriaglabra(Merr.)M.P.Nayarssp.kinabaluensis Veldkamp, Blumea 24: 422, fig. 1A, 4C. 1978.

##### Type.

Malaysia. Sabah: Sosopodon, near Kundasang, 4500 ft. elev., 15 Jul 1964, G. Mikil SAN 46742 (holotype: L [L0008869]!; isotypes: K [K000859551]!, L [L0008870]!, SAN *n.v.*).

**Figure 15.**
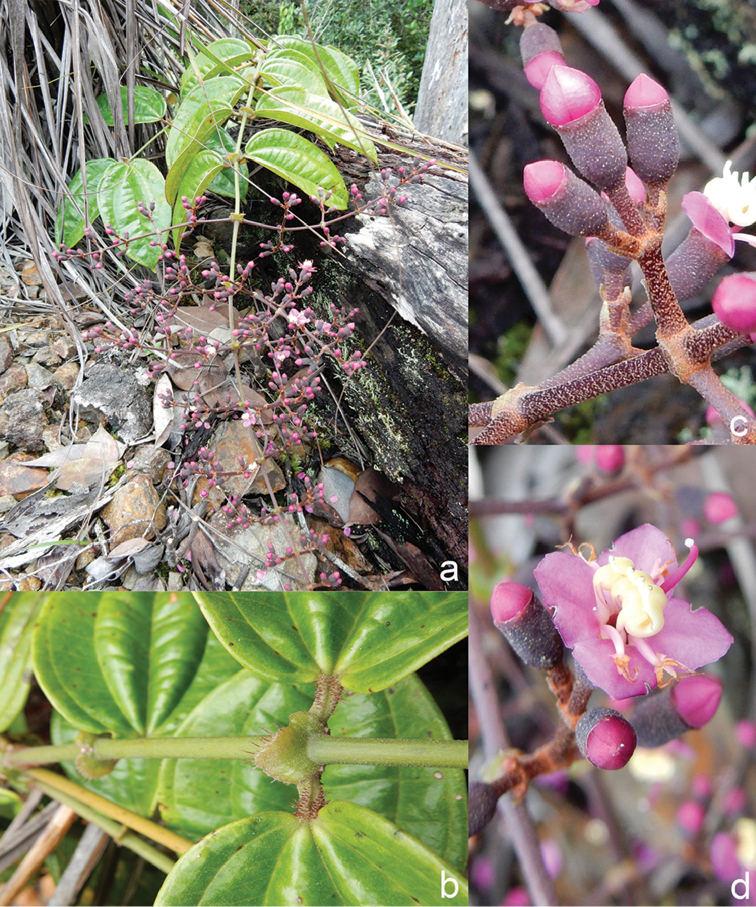
Dissochaetaglabravar.kinabaluensis. **a** habit **b** branchlet **c** hypanthium **d** flower. Photographs by D. Penneys; voucher: Penneys 2542 (WNC).

**Map 14.**
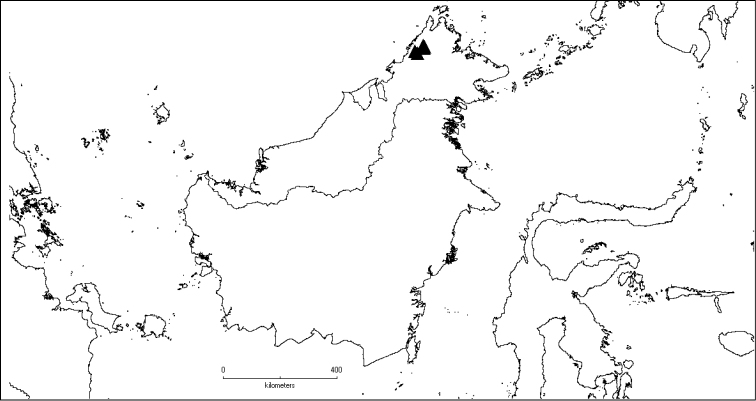
Distribution of D.glabravar.kinabaluensis (▲).

##### Description.

Hypanthium 5–6 × ca. 3.5 mm, glabrous, reddish; petal bud conical, 4–5 mm long; mature petals suborbicular, 5–6 × ca. 4.5 mm, not reflexed, violet or pink, base clawed, apex acute. Stamens 8, unequal, filaments straight; alternipetalous stamens staminodial, with ca. 5 mm long filaments, thecae 1–2 mm long, basal crest triangular, ca. 2 mm long, thin, apex acute, base emarginate or hastate, lateral appendages prolonged from the locule vestige, paired, filiform, 5–6 mm long; oppositipetalous stamens with ca. 6 mm long filaments, thecae 5–6 mm long, yellow, basal crest bifid, ca. 2 mm long, lateral appendages ligular with bifid apex, ca. 1 mm long. Ovary half as long as hypanthium, apex glabrous; style glabrous, 11–12 mm long, curved at the end, slender; stigma minute, capitate; extra-ovarial chambers 8, extending to ⅓ of the ovary. Fruits urceolate, 6–7 × ca. 5 mm, glabrous.

##### Distribution.

Borneo (Sabah).

##### Ecology and habitat.

Montane forest at 1300–1500 m elevation.

##### Note.

This variety differs from var. glabra in having larger inflorescences, flowers and fruits. The fruits of var. kinabaluensis are rather urceolate instead of subglobose. The distribution of the variety is restricted to the montane forest of the Mount Kinabalu Complex and Crocker Range in Sabah, Borneo.

##### Specimens examined.

**MALAYSIA. Sabah**: Ranau, Mt. Kinabalu, Ulu Liwagu-Ulu Mesilau, 1220 m, 2 Sep 1961, W.L. Chew, A.J.G.H. Corner & A. Stainton RSNB 2647 (K, L); *Ibid.*, Tenompok, 1500 m, 29 Apr 1932, J. Clemens & M.S. Clemens 29442 (BM, K, L, NY); *Ibid.*, Penibukan, 1500 m, 10 Jan 1933, J. Clemens & M.S. Clemens 30865 (BM, K, L); *Ibid.*, 7 Feb 1933, J. Clemens & M.S. Clemens 31520 (BM); *Ibid.*, 2000 m, 26 Oct 1933, J. Clemens & M.S. Clemens 40940 (BM, K, L); *Ibid.*, 1670 m, 13 Nov 1933, J. Clemens & M.S. Clemens 50337 (BM, K); *Ibid.*, Gurulau, 1500 m, 25 Nov 1933, J. Clemens & M.S. Clemens 50476 (BM, K, L); *Ibid.*, 29 Nov 1933, J. Clemens & M.S. Clemens 50555A (BM); *Ibid.*, Sosopodon, Kundasang, 1370 m, 15 .Jul 1964, G. Mikil SAN 46742 (K, L); Tambunan, Mount Alab, 1500 m, 2 Mar 1995, J.T. Pereira et al. 111 (L).

#### 21. Dissochaeta glandiformis

Taxon classificationPlantaeMyrtalesMelastomataceae

J.F.Maxwell, Gard. Bull. Singapore 33: 313, fig. 1. 1980.

Map 15

##### Type.

Indonesia. Jambi: Kerintji Region, Gunung Tudjuh, 1800 m elev., Jul 1956, W. Meijer 7282 (holotype: L [L0537274]!).

**Map 15.**
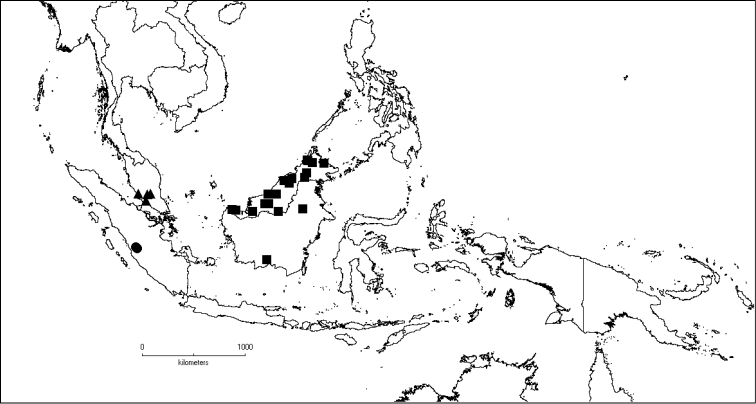
Distribution of *D.glandiformis* (●), *D.glandulosa* (■) and *D.griffithii* (▲).

##### Description.

Climbing up to 10 m in height. Branchlets terete, 4–5 mm diameter, densely stellate-furfuraceous; nodes swollen, with annular ridge crest-like, angular or ligular interpetiolar ridges up to 5 mm wide; internodes 6–8 cm long. Leaves: petioles flattened, 10–16 mm long, densely stellate-furfuraceous; blades ovate to ovate-elliptic, 7–16 × 5–8.5 cm, membranous, base rounded, margin entire, apex acuminate, tip 0.5–2 cm long; nervation with 1 pair of lateral nerves and 1 pair of intramarginal nerves; adaxially glabrous, abaxially densely brown stellate-tomentose. Inflorescences terminal and in the upper leaf axils, up to 22 cm long, many-flowered; main axis angular, densely stellate-furfuraceous; primary axes up to 18–20 cm long with 4–5 nodes, secondary axes 2–5 cm long with 2 or 3 nodes, tertiary axes 1–2.5 cm long with 1 or 2 nodes, quartenary axis up to 0.5 cm long when developed with 1 node; bracts minute, inconspicous; bracteoles linear, 1–2 mm long, densely stellate-furfuraceous; pedicels densely stellate-furfuraceous, 4–5 mm long in central flowers, 2–3 mm long in lateral flowers. Hypanthium campanulate, slightly angular, 3–5 × 1–3 mm, with four distinct ridges, densely stellate-tomentose, somewhat pointing sideways from pedicels; calyx lobes triangular with rounded tips, 2–2.5 mm long, widened, stellate-furfuraceous; lobes in bud distinctly united in thin acorn-like shape with sutures and opening when mature; petal bud conical, 3–6 mm long; mature petals ovate or oblong, 6–9 × 3–5 mm, base clawed, apex obtuse, glabrous or inside at base with appressed hairs, pink. Stamens 4, equal, alternipetalous, filaments flat, 4–6 mm long, straight, apex bent, anthers linear-lanceolate, sickle-shaped or falcate, thecae 5–6 mm long, yellow, pedoconnective ca. 1 mm long, basal crests minute, triangular, ca. 1 mm long, narrow with acute apex, lateral appendages paired, filiform, 3–5 mm long, tan. Ovary half or ⅔ of hypanthium in length, apex pubescent; style erect, 8–13 mm long, curved at apex, glabrous, purple; stigma minute; extra-ovarial chambers 4, alternipetalous, extending to the base of the ovary. Fruits ovoid, 5–6 × 3–5 mm, glabrous to sparsely stellate-furfuraceous, rarely with distinct vertical ridges, green, often pointing sideways from the pedicels; calyx lobe remnants persistent or sometimes caducous. Seeds ca. 0.5 mm long.

##### Distribution.

Sumatra (West).

##### Ecology and habitat.

Montane forest at 900–2500 m elevation.

##### Note.

*Dissochaetaglandiformis* resembles to *D.intermedia* with brown stellate- tomentose hairs in leaf blades abaxiallyly and having only 4 alternipetalous stamens with two filiform lateral appendages. This species differs from the latter by having a conspicous interpetiolar ridge (annular and crest-like or ligular) at the nodes and longer calyx lobes (2–2.5 mm long). The calyx lobes are distinctly united and have an acorn-like appearance when in bud, splitting into four lobes at maturity (Maxwell 1980b). This species is restricted to the montane forest of the Mount Kerinci complex, Sumatra.

##### Specimens examined.

**INDONESIA. Jambi**: Mt. Tujuh, 1800 m, Jul 1956, W. Meijer 7282 (L); *Ibid.*, 6 Aug 1956, M. Jacobs 4517 (BO). **West Sumatra**: Mt. Kerinci, 900 m, 8 Feb 1920, H.A.B. Bünnemeijer 8074 (BO, L); *Ibid.*, 2500 m, 7 Apr 1920, H.A.B. Bünnemeijer 9230 (BO, K, L); *Ibid.*, 2000 m, 13 Apr 1920, H.A.B. Bünnemeijer 9379 (BO, L, U); *Ibid.*, 1800 m, 12 May 1920, H.A.B. Bünnemeijer 10477 (BO, L).

#### 22. Dissochaeta glandulosa

Taxon classificationPlantaeMyrtalesMelastomataceae

Merr., Univ. Calif. Publ. Bot. 15: 224. 1929.

Map 15

##### Type.

Malaysia. Sabah: Myburgh Province, Sandakan, Oct–Dec 1921, A.D.E. Elmer 20259 (lectotype, designated here: BO [BO1421691]!; isolectotypes: BISH [BISH-1003260, image seen]!, BM [BM001190924, BM001190925]!, BR [BR00000522241, image seen]!, BRI [BRI-AQ0023052, image seen]!, C [C10014564, image seen]!, CAS [CAS0033425, image seen]!, CM [CM-1527, image seen]!, F [F65407, image seen]!, GH [GH00072204, GH00072205, images seen]!, HBG [HBG514873, image seen]!, K [K000859503]!, L [L0537261]!, MICH [MICH-1111782, image seen]!, NY [NY00228564, image seen]!, PH [PH00009602, PH00009603, images seen]!, S [SG-2104, image seen]!, U [U0124130]!).

##### Description.

Climbing up to 15 m in height. Branchlets terete, 3–4 mm in diameter, glabrous; nodes swollen, with crest-like interpetiolar ridge; internodes 7–11 cm long. Leaves: petioles terete, 1.3–1.8 cm long, glabrous; blades broadly ovate, 8.5–11.5 × 5.6–6.7 cm, subcoriaceous, base rounded to emarginate, margin entire, apex acuminate, tip up to 1 cm long; nervation with 1 pair of lateral nerves and 1 pair of intramarginal nerves; surfaces glabrous, basally with a pair of glandular patches on abaxially. Inflorescences terminal, up to 25 cm long, many-flowered; main axis glabrous; primary axes up to 11 cm long with 3 nodes, secondary axes 4–4.5 cm long with 1 or 2 nodes, tertiary axes ca. 2 cm long with 1 node; bracts elliptic-oblong, 20–27 × 8–10 mm, glabrous, whitish; bracteoles oblong to lanceolate, ca. 14 × 3–4 mm, glabrous, whitish; pedicels glabrous, purplish, 4–5 mm long in central flowers, 3–4 mm long in lateral flowers. Hypanthium campanulate, 5–6 × 4–5 mm, glabrous, at early stages subglobose to urceolate and enclosing petal bud; calyx lobes truncate, level, 1–2 mm long; petal bud conical, 6–7 mm long; mature petals broadly ovate, 5–6 × ca. 5 mm, base clawed, apex acute, purple above, whitish below. Stamens 8, subequal, filaments curved sideways, light yellow; alternipetalous stamens with ca. 6 mm long filaments, anthers slender, curved, sickle-shaped, thecae ca. 4 mm long, pedoconnective ca. 1 mm long, apex acute, basal crest thin, 1–1.5 mm long, margin erose to fimbriate, lateral appendages paired, filiform, 2–4 mm long, fimbriate; oppositipetalous stamens with 5–6 mm long filaments, bent at the attachment to anthers, anthers thick, slightly curved, hook-shaped, thecae 5–6 mm long, apex obtuse, yellow, basal crest thin, ligular, 2–2.5 mm long, bifid, lateral appendages paired, filiform, up to 2 mm long. Ovary half as long as hypanthium, apex villous; style ca. 14 mm long, glabrous, curved at the end, slender, light green-yellow; stigma minute, purplish; extra-ovarial chambers shallow to nearly undeveloped. Fruits urceolate, ca. 10 × 6 mm, glabrous; calyx lobe remnants persistent. Seeds ca. 0.75 mm long.

##### Distribution.

Borneo.

##### Ecology and habitat.

Lowland Mixed Dipterocarp forests on ridge at 50–400 m elevation.

##### Note.

*Dissochaetaglandulosa* resembles *D.beccariana* because of the similar glabrous indumentum and conspicuous white bracts. However, this species has a much larger campanulate hypanthium (5–6 × 4–5 mm in *D.glandulosa*, 4–7 × 2–3 mm in *D.beccariana*) and urceolate fruits (subglobose in *D.beccariana*).

##### Specimens examined.

**BRUNEI. Belait**: Labi, Bukit Teraja, 350 m, 18 Oct 1991, D.A. Simpson & M. Marsh 2115 (K, L). **Temburong**: Amo, Ulu Belalong, 480 m, 20 Jan 1994, M.J.E. Coode et al. 7864 (L). **MALAYSIA. Sabah**: Nabawan, Ulu Sungai Nabawan, 22 Feb 1990, Fidilis SAN 128385 (L); Penampang, Crocker Range, Kota Kinabalu–Tambunan Road, 900 m, 1 Oct 1983, J.H. Beaman & R.S. Beaman 7110 (K, L); Pensiangan, Pensiangan Kayu FR, 23 Jul 1992, Fidilis SAN 136035 (K, L); Sandakan, Myburgh, Oct-Dec 1921, A.D.E. Elmer 20259 (BISH, BM, BO, BR, BRI, C, CAS, CM, F, GH, K, L, MICH, NY, PH, S, U); Ranau, Ulu Tungud, 343 m, 27 Jul 2005, L.G. Saw et al. SAN 146062 (L); *Ibid.*, Mount Kinabalu, Penibukan, 1200 m, 14 Mar 1931, J.Clemens & M.S. Clemens 32150 (BM). **Sarawak**: Betong, Batang Layar, 1 Jul 1980, B. Lee S.41990 (L); Bintulu, Bukit Pesu, Ulu Kuala Semut, 160 m, 20 Aug 1963, H.P. Fuchs 21352 (K, L); *Ibid.*, Ulu Segan, 274 m, 25 Aug 1968, Ilias Paie S.27219 (K, L); Kapit, Belaga, Rejang River, 500 m, 31 Aug 1958, M. Jacobs 5361 (K, L), Pelagus Protected Forest, 100 m, 16 Sep 1973, P.K. Chai et al. S.33173 (BO, K, L); *Ibid.*, Ulu Baleh, 400 m, 6 May 1991, Runi et al. S.63213 (K, L); Kuching, Selang FR., 91 m, 25 Jul 1957, Ilias Paie S.8462 (K, L); Lundu, G.D. Haviland 1508 (BM, K); Miri, Mulu, Gunung Mulu National Park, 400 m, 14 Oct 1977, P.K. Chai S.39492 (K, L). **INDONESIA. Central Kalimantan**: Kotawaringin Timur, Mentaya River, 50 m, 11 Feb 1994, G. Argent & P. Wilkie 9441 (L). **East Kalimantan**: West Kutai, Long Petah, 600 m, 10 Sep 1925, F.H. Endert 3126 (BO, K).

#### 23. Dissochaeta gracilis

Taxon classificationPlantaeMyrtalesMelastomataceae

(Jack) Blume, Flora 14: 498. 1831.

Fig. 16 , Map 16

Melastoma gracile Jack, Trans. Linn. Soc. London 14: 14. 1823 (“gracilis”).
Melastoma vacillans Blume var. pallens Blume, Bijdr. Fl. Ned. Ind. 17: 1074. 1826. Type: Indonesia. Java, C.L. Blume s.n. (lectotype, designated by Kartonegoro and Veldkamp 2010, pg. 134: L [L0008889]!; isolectotypes: BO!, L [L0008885, L0008886, L0008887, L0008888]!).
Dissochaeta brachyanthera Naudin, Ann. Sci. Nat., Bot. sér. 3, 15: 74. 1851. Type: Indonesia. West Java: Mount Perbakti, 2 Jun 1848, H. Zollinger 3511 (lectotype, designated here: P [P05283573, image seen]!; isolectotypes: A [A00072203, image seen]!, BM!, L [L0537238]!, P [P05283585, P05283587, images seen]!).
Neodissochaeta gracilis (Jack) Bakh.*f.*, Contr. Melastom.: 137. 1943.
Neodissochaeta puberula Bakh.*f.*, Contr. Melastom.: 139. 1943. Type: Indonesia. East Kalimantan: Samarinda, Sungai Wain, L.M.R. Rutten 86 (holotype: U [U0004008]!).
Neodissochaeta compressa Bakh.*f.*, Contr. Melastom.: 146. 1943. Type: Indonesia. South Kalimantan: Limowia, Batoe Babi, 10 Jul 1908, H.J.P. Winkler 2809 (holotype: L [L0537275]!; isotypes: BM [BM001190927]!, BO [BO1765008]!, K [K000859495]!, WRSL *n.v.*).

##### Type.

Indonesia. Sumatra, *Jack s.n.* (lost); Indonesia. Bengkulu: Boekit Daoen, Balai, 1000 m elev., 13 Jul 1931, C.N.A. de Voogd 591 (neotype, designated by Kartonegoro and Veldkamp 2010, pg. 134: L [L0822677]!; isoneotype: BO!).

**Figure 16.**
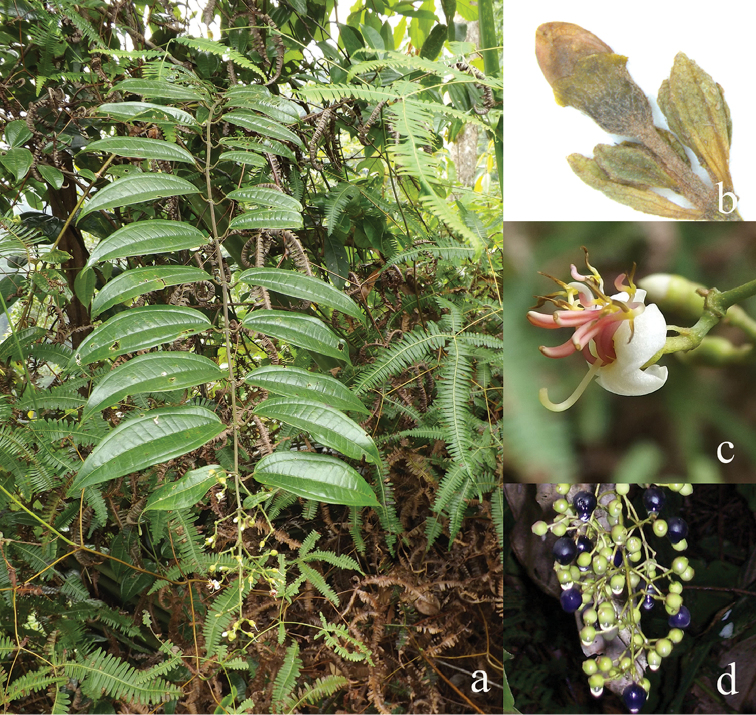
*Dissochaetagracilis*. **a** habit **b** hypanthium **c** flower **d** fruits. Photographs by A. Kartonegoro; vouchers: Kartonegoro 1113 (BO, L).

**Map 16.**
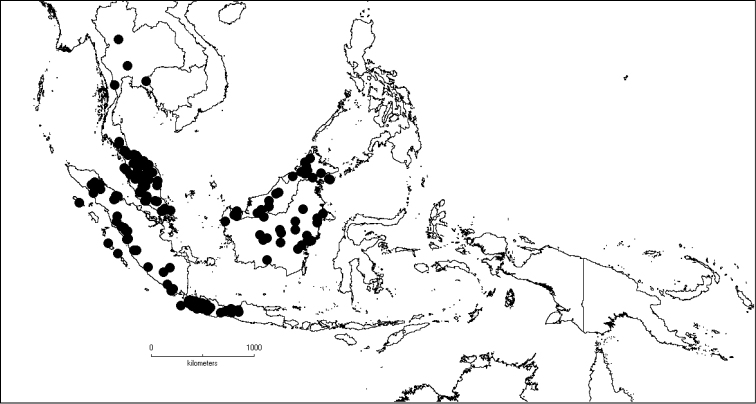
Distribution of *D.gracilis* (●).

##### Description.

Climbing up to 20 m in height. Branchlets terete or nearly quadrangular, 3–4 mm in diameter, glabrous or sparsely puberulous; nodes swollen, with interpetiolar line; internodes 4–6.5 cm long. Leaves: petiole terete, 7–15 mm long, glabrous or sparsely puberulous; blade ovate to elliptic, 8–17 × 3–8 cm, membranous, base rounded, margin entire, apex acuminate, tip 0.5–1 cm long; nervation with 1 pair of lateral nerves and 1 pair of intramarginal nerves; surfaces glabrous. Inflorescences terminal and in the upper leaf axils, 8–20 cm long, cymous, with many flowers; main axis angular, glabrous to sparsely furfuraceous; primary axes 5–17 cm long with 4–6 nodes, secondary axes 1–2 cm long with 2 or 3 nodes, tertiary axes 0.5–0.6 cm long with 1 or 2 nodes; bracts linear or elliptic, 3–6 mm long, thin, sparsely or densely stellate-pubescent; bracteoles linear or rarely oblong, 6–8 mm long, glabrous to stellate-pubescent; pedicels glabrescent, 3–4 mm long in central flowers, 1–2 mm long in lateral flowers. Hypanthium campanulate or suburceolate, 2–3 × ca. 2 mm, glabrous or sparsely puberulous; calyx lobes truncate with undulate tips, 0.5–0.7 mm long, rarely with minutely triangular tip, glabrous; petal bud conical, 2–3 mm long; mature petals ovate, oblong or suborbicular, 2–3 × 2–3 mm, reflexed, base clawed, apex obtuse, glabrous, veined, white or pale pink. Stamens 8, unequal, filaments curved sideways; alternipetalous stamens with 3–5 mm long filaments, curved, bent at end point, anthers clavate, sickle-shaped, thecae 4–6 mm long, white or pink, pedoconnective white, 1–2 mm long, basal crests membranous, 0.25–0.5 mm long, thin, lateral appendages paired, flat, wavy, filiform, ca. 2 mm long, yellow; oppositipetalous stamens with ca. 2 mm long filaments, anthers smaller, slender, somehow staminodial, curved, thecae 1–1.5 mm long, white or pinkish, basal crests minute or spuriform, erect or ligular, ca. 0.3 mm long, lateral appendages paired, filiform, 1.5–2 mm long, yellow. Ovary half as long as hypanthium, apex villous or glabrous; style 4–6 mm long, curved at end. glabrous, white; stigma minute; extra-ovarial chambers 4, alternipetalous, extending to the middle of the ovary. Fruits globose, 5–6 × 2–4 mm, glabrous, bright green with distinct, 8-lined, purple when ripe; calyx lobe remnants persistent. Seeds ca. 0.5 mm long.

##### Distribution.

Thailand, Peninsular Malaysia, Sumatra, Java and Borneo.

##### Ecology and habitat.

Primary or secondary disturbed forest, regrowth forest or riparian, lowland to mountain forest at 10–1500 m elevation.

##### Vernacular names.

Peninsular Malaysia: *sedudu akar* (Malay). Sumatra: *alor sitabong detay* (Simeuleu). Java: *kecambang areuy*, *harendong areuy*, *kingkilaban* (Sunda), *Kalikadep*, *walak* (Java).

##### Note.

This species can easily be distinguished by its thin leaf blades, glabrous on both surfaces and with four fertile alternipetalous stamens and four oppositipetalous staminodes. It has the smallest hypanthium of all species. The extra-ovarial chambers are shallow when compared to the size of the hypanthium and fertile stamens. Amongst all the species, *D.gracilis* is the most common species found in a wide elevation range, between 10–1500 m elevations. This species is also widespread west, but never east of Wallace’s line.

##### Selected specimens examined.

**THAILAND. Ayutthaya**: Sukirin Poo Kao Tong, 200 m, 28 Mar 1987, J.F. Maxwell 87-254 (L, P). **Chanthaburi**: Khao Soi Dao, 100 m, 29 Apr 1930, A.F.G. Kerr 19233 (BM, K). **Narathiwat**: Waeng, 15 Nov 1962, B. Sangkhachand BKF 46913 (K); *Ibid.*, Hala Bala 380 m, 20 Apr 2004, Chongko 315 (L); Sungei Kolok, 28 Feb 1974, K. Larsen & S.S. Larsen 32731 (L). **Pattani**: Kao Kalakiri, 600 m, 11 Sep 1923, A.F.G. Kerr 7812 (BM, K). **Petchaburi**: Khao Daen, 200 m, 19 Apr 1928, A.F.G. Kerr 15310 (BM, K). **Songkhla**: Khao Nam Kang, Na Thawee, 100-150 m, 13 Jun 1992, K. Larsen et al. 42845 (P); Ton Nga Chang, 250 m, 17 Aug 1995, K. Larsen et al. 45732 (L). **Sukhothai**: Klaung Tan, 400 m, 11 Mar 1928, A.F.G. Kerr 14470 (BM, K). **Trang**: Trang Ridge, 800 m, 28 Aug 1915, Vanpruk 697 (K); Khao Pap Pa, 13 Mar 1974, K. Larsen & S.S. Larsen 33279 (L). **Yala**: 8 Feb 1973, B. Sangkhachand & S. Phusomsaeng 1539 (P); Betong, 300 m, 31 Jul 1923, A.F.G. Kerr 7433 (BM, K); Bannang Sata, 21 Dec 1966, B. Sangkhachand BKF 40970 (K, L, P). **MALAYSIA. Johor**: Panti, 19 Nov 1966, T. Suppiah KEP 98980 (K, L); Labis, 150 m, 14 Apr 1967, T. Suppiah KEP 104968 (K, L); Kota Tinggi, 50 m, 22 Apr 1978, J.F. Maxwell 78-211 (L). **Kelantan**: Channing, 2 Feb 1917, H.N. Ridley s.n. (K); Ulu Sungai Lebir Kecil, 17 Sep 1967, P.F. Cockburn FRI 7132 (K, L). **Malacca**: W. Griffith s.n. (BM). **Negeri Sembilan**: Jelebu, Serting Forest Reserve, 200 m, 1 Oct 1996, E.C. Gardette 2280 (L). **Pahang**: Kuala Lipis, Ulu Chimeras, 21 Nov 1924, I.H. Burkill & M. Haniff SFN 15726 (BO); Kuala Tahan, 60 m, 16 Feb 1968, M. Shah 1308 (K, L); Ulu Sungai Kuantan, 183 m, 11 Jun 1934, C.F. Symington & Kiah SFN 28776 (BO, K). **Penang**: N. Wallich 4050 (P); Batu Kawau, Aug 1885, C. Curtis 398 (K). **Perak**: Goping, King’s collector 657 (BM, K, P); Gunong Bubu, 610 m, 18 Aug 1966, W.L. Chew 1236 (K, L); Larut & Matang, Bukit Larut, 645 m, 21 Mar 2007, S.N. Phoon & H.L. Kueh FRI 53289 (K, L). **Selangor**: Rawang, May 1896, H.N. Ridley 7332 (K); Genting Highlands, Ulu Gombak 325 m, 2 Jun 1978, J.F. Maxwell 78-297 (L). **Terengganu**: Kemaman, Bukit Kajang, 183 m, 4 Nov 1935, E.J.H. Corner SFN 30223 (K); Ulu Terengganu, Sekayu, Bt. Lanjut, 20 Sep 1969, H.L. Loh FRI 13499 (K, L). **Sabah**: Keningau, Tulid Area, Ulu Sg. Sembuan, 20 Sep 1988, Asik Mantor SAN 125639 (K, L); Lahad Datu, Danum Valley, 14 May 1989, C.E. Ridsdale 2017 (L); Ranau, Kampong Poring, 30 Mar 1995, S. Sambuling 596 (K); Tawau, Mar 1923, A.D.E. Elmer 21427 (BM, BO, K, L, U); Semporna, Sg. Montoritip, 13 m, 24 Feb 1964, Aban Gibot SAN 40929 (L). **Sarawak**: Bukit Mersing, Anap, 200 m, 24 Aug 1964, Sibat ak Luang S.21910 (BO, K, L); Kuching, 17 Oct 1894, G.D. Haviland & C. Hose 3387 (BM, K, L); *Ibid.*, Mount Penrissen, 1100 m, 4 Dec 1994, J.H. Beaman & Gregory-Smith 11090 (K); Limbang, Bukit Pagon, Sg. Sipayan, 540 m, 3 Aug 1984, D. Awa & B. Lee S.47645 (K); Dataran Tinggi Merurong, Sungei Jelalong, Sungei Ebau, 350 m, 11 Oct 1984, Othman et al. S.48867 (K, L). **SINGAPORE.** Bukit Timah, 1894, E. Langlasse 83 (P). **BRUNEI. Temburong**: Batu Apoi, 350 m, 29 Oct 1991, D.A. Simpson & M. Marsh 2503 (K, L). **INDONESIA. Aceh**: Gayoland, Palo to Kongke, 1000 m, 4 Mar 1937, C.G.G.J. van Steenis 9478 (BO, K, L); Kutacane, Gunung Gurah, 22 Mar 1954, A.H.G. Alston 14627 (BM, BO); Mt. Leuser, Bengkong River, 200 m, 16 Jul 1979, W.J.J.O. de Wilde & B.E.E. de Wilde-Duyfjes 18739 (BO, K, L); Simeuleu Island, Tapah, 26 Mar 1920, Achmad 1757 (BO, K, L). **Bengkulu**: Rimbo Pengadang, 1000 m, 9 Jun 1916, Ajoeb 115 (BO); Bukit Daun, Balai, 1000 m, 13 Jul 1931, C.N.A. de Voogd 591 (BO, L). **Jambi**: Kerinci, Siolak Deras, 915 m, 18 Mar 1914, H.C. Robinson & C. Boden-Kloss s.n. (BM, K). **Lampung**: Semaka, Sukaraja, 530 m, 28 Aug 2008, D. Arifiani et al. 922 (BO); Kota Agung, Ulu Belu, 22 Aug 1915, P.J.S. Cramer 90 (BO, L). **Mentawai Islands**: Siberut, 8 Jul 1953, J. van Borssum-Waalkes 2659 (BO, K, L); Sipora, 9 Oct 1924, C. Boden-Kloss SFN 14661 (BO, K). **North Sumatra**: Bukit Lawang, 100–150 m, 30 Jan 1980, H. Wiriadinata & Maskuri 538 (BO, K, L); Asahan, Bandar Puluh, H.S. Yates 1599 (BO, P); *Ibid.*, H.S. Yates 1271 (BM, BO, L). **South Sumatra**: Muara Dua, Tenang, 700 m, 10 Jan 1930, C.N.A. de Voogd 555 (BO); Lake Ranau, G. Raya, 1300 m, 2 Nov 1929, C.G.G.J. van Steenis 3538 (BO, L). **West Sumatra**: Bukit Tinggi, Mangani, 1100 m, 15 Jun 1918, H.A.B. Bünnemeijer 3015 (BO, L); Agam, Brani, 900 m, 22 Jun 1918, H.A.B. Bünnemeijer 3199 (BO, L, U). **Banten**: Pandeglang, Menes, 100 m, Mar 1913, C.A. Backer 7032 (BO); Mt. Pulosari, 900 m, Mar 1913, C.A. Backer 7054 (BO); Ujung Kulon, Mt. Payung, 300–400 m, 8 Jan 1964, N. Wirawan 265 (BO, K, L). **Central Java**: Banyumas, Mt. Slamet, upper Baturaden, 800 m, 12 Apr 1911, C.A. Backer 186 (BO); Pekalongan, Between Doro and Petung Kriono, 600 m, 8 Sep 1914, C.A. Backer 15750 (BO); Semarang, Mt. Ungaran 1200 m, 22 Mar 1913, W.M. Docters van Leeuwen 1264 (BO); *Ibid.*, Mt. Telomoyo, S.H. Koorders 35839β (BO). **West Java**: Bogor, Mt. Salak, Gunung Bunder, 1000 m, 8 Aug 1909, C.A. Backer 31628 (BO); Leuwiliang, Cianten, 1000 m, 1 Sep 1918, C.A. Backer 25894 (BO); Puraseda, 450 m, 20 Dec 1930, R.C. Bakhuizen van den Brink 7641 (BO, K, L, U); Mt.Pangrango, Bodogol, 600 m, 4 Apr 2009, A. Kartonegoro 314 (BO); Mt. Halimun, Nirmala, 1100 m, 27 Dec 1913, C.A. Backer 11160 (BO); *Ibid.*, 1300 m, 10 Jun 1980, M.M.J. van Balgooy & H. Wiriadinata 2921 (BO, K, L); *Ibid.*, 12 Oct 2017, A. Kartonegoro 1113 (BO, L); Sukabumi, Jampang Tengah, 650 m, 27 Sep 1970, A.J.G.H. Kostermans 23846 (BO, K, L); Cianjur, Cibeber, Cidadap, 1000 m, 12 Jun 1917, C.A. Backer 22493 (BO); *Ibid.*, 12 Jun 1916, R.C. Bakhuizen van den Brink 3839 (BO, K, L). **Central Kalimantan**: Bukit Raya, Tumbang Riang, 150 m, 25 Nov 1982, J.P. Mogea & W.J.J.O. de Wilde 3669 (BO, L); *Ibid.*, Tumbang Tapi, 100 m, 20 Jan 1983, J.F. Veldkamp 8326 (BO, L, PNH); Barito Ulu, 25 May 1990, C.E. Ridsdale PBU 187 (BO, K, L). **East Kalimantan**: West Kutai, Long Liang Beng, 250 m, 31 Aug 1925, F.H. Endert 3015 (BO, K, L); *Ibid*., Kombeng, 30 m, 23 Nov 1925, F.H. Endert 5174 (BO, K, L, PNH). **South Kalimantan**: Limowia, Batu Babi, 10 Jul 1908, H.J.P. Winkler 2809 (BM, BO, K, L). **West Kalimantan**: Long Blu’u, 1896, Jaheri 1297 (BO); Sintang, HPH Km. 87, 100 m, 24 Apr 1994, A.C. Church et al. 1078 (BO, K, L).

#### 24. Dissochaeta griffithii

Taxon classificationPlantaeMyrtalesMelastomataceae

(M.P.Nayar) Karton comb. nov.

urn:lsid:ipni.org:names:60476837-2

Map 15

Dissochaeta annulata Hook.*f.* ex Triana var. griffithii (M.P.Nayar) J.F.Maxwell, Gard. Bull. Singapore 33: 313. 1980.

##### Basionym.

*Macrolenesgriffithii* M.P.Nayar, J. Jap. Bot. 55: 47. 1980.

##### Type.

Malaysia. Malacca, W. Griffith KD 2269 (holotype: K [K001096571]!).

##### Description.

Branchlets terete, 3–4 mm in diameter, densely covered with brown stellate-furfuraceous hairs and simple dark, 2–3 mm long bristle hairs; nodes swollen, with distinct interpetiolar ridge; internodes 4–5.5 cm long. Leaves: petioles terete, 3–6 mm long, densely covered with brown stellate-furfuraceous hairs and simple dark, 2–3 mm long bristle hairs; blades ovate or ovate-oblong, 6–10 × 2.5–3.5 cm, subcoriaceous, base cordate, margin entire, apex acuminate, tip ca. 0.5 cm long; nervation with 1 pair of lateral nerves and 1 pair of intramarginal nerves; adaxially glabrous, dark green, abaxially densely covered with brown stellate-furfuraceous hairs. Inflorescences terminal or in upper leaf axils, up to 12 cm long when terminal, many-flowered, 3–4 cm long when axillary, with 1–3 flowers; main axis angular, densely covered with brown stellate-furfuraceous hairs and simple dark bristle hairs; primary axes 8–9 cm long with 3 or 4 nodes, secondary axes 1–2 cm long with 1 or 2 nodes, tertiary axes when developed ca. 0.5 cm long with 1 node; bracts oblong to lanceolate, 15–20 × 6–8 mm, densely brown stellate-furfuraceous; bracteoles lanceolate, 10–15 × ca. 2 mm, densely brown stellate-furfuraceous; pedicels densely covered with bristle hairs, ca. 1 mm long in central flowers, ca. 0.5 mm long or nearly sessile in lateral flowers. Hypanthium campanulate, 8–10 × 5–7 mm, densely covered with stellate-tomentose hairs and simple 3–5 mm long eglandular bristle hairs; calyx lobes triangular with acute tips, 2.5–3 × 2–3 mm; petal buds conical, 6-8 mm long, apex acute; mature petals ovate to oblong, 11–14 × 6–7.5 mm, reflexed, base clawed, apex obtuse, glabrous with ciliate margin, pink. Stamens 8, unequal, filaments curved sideways, light yellow; alternipetalous stamens with 12–14 mm long filaments, anthers lanceolate, slender, sickle-shaped, thecae 16–18 mm long, apex rostrate, pedoconnective 4–5 mm long, basal crest entire, erose or bifid, 1–2 mm long, lateral appendages paired, filiform, 6–7 mm long; oppositipetalous stamens with 11–12 mm long filaments, anthers hook- or S-shaped, thecae 10–12 mm long, basal crest ligular, obtuse or erose, ca. 1 mm long, lateral appendages paired, filiform, 4–5 mm long, yellow. Ovary ¾ of hypanthium in length, apex villous; style 22–24 mm long, curved sideways in direction opposite to the filaments, curved at the tip, glabrous; stigma minute; extra-ovarial chambers 8, extending to near the base of the ovary. Fruits ovoid to nearly subglobose, 13–15 × 5–8 mm, densely covered with stellate-tomentose hairs and simple 3–5 mm long eglandular bristle hairs; calyx lobe remnants persistent. Seeds ca. 0.5 mm long.

##### Distribution.

Peninsular Malaysia.

##### Ecology and habitat.

Lowland primary forest in open places at 27–120 m elevation.

##### Note.

1. The appearance of the hypanthium with distinct calyx lobes was recognised by Nayar (1980) as typical for *Macrolenes*, but the character of the stamens and its appendages closely resemble *Dissochaeta*, with a pair of filiform appendages on the alternipetalous stamens and not fimbriate appendages as is common in *Macrolenes*. This species resembles *Macrolenesechinulata* (Naudin) Bakh.*f.* which is also distributed in Peninsular Malaysia but differs in lacking paired hair cushions at the base of the leaves abaxiallyly (present in *M.echinulata*) and only has one pair of lateral appendages at alternipetalous stamens (which are fimbriate in *M.echinulata*).

2. Maxwell (1980b) reduces this species to a variety of *D.annulata* based on the similarity in the number and shape of the stamens. *Dissochaetagriffithii* differs from *D.annulata* by its distinct triangular calyx lobes (truncate in *D.annulata*) and hypanthium with dense simple eglandular bristle hairs (lacking or with scattered glandular bristle hairs in *D.annulata*).

##### Specimens examined.

**MALAYSIA. Malacca**: W. Griffith KD 2269 (K); A.C. Maingay KD 784 (K). **Negeri Sembilan**: Jelebu, Pasoh Forest Reserve, 80–120 m, 9 Jun 1996, E.C. Gardette 1989 (K, L). **Pahang**: Tasek Bera, 76 m, 28 Oct 1961, W.L. Chew & M. Noor 270 (K, L). **Selangor**: Rantau Panjang, 27 m. 29 Sep 1927, E.J. Strugnell 13965 (K).

#### 25. Dissochaeta hirsutoidea

Taxon classificationPlantaeMyrtalesMelastomataceae

Furtado, Gard. Bull. Singapore 20: 109, fig. 2C. 1963.

Fig. 17 , Map 17

Dissochaeta stellulata Furtado, Gard. Bull. Singapore 20: 113, fig. 2F. 1963. Type: Malaysia. Sarawak: Lodong, G.D. Haviland 862 (holotype: SAR *n.v.*; isotype: K [K000859626]!).

##### Type.

Malaysia. Sabah: Sandakan, Bettotan, 19 Aug 1927, C. Boden-Kloss SFN 19156 (holotype: SING *n.v.*; isotypes: BO!, K [K000859628]!).

**Figure 17.**
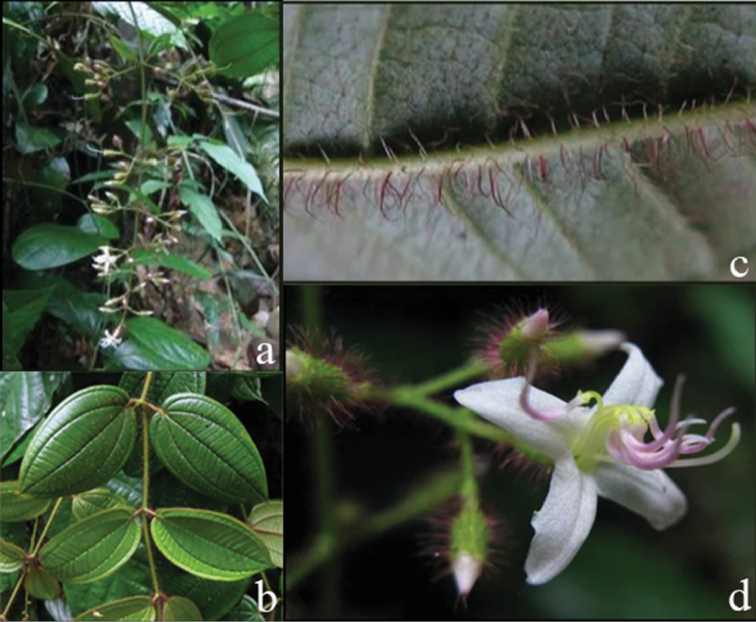
*Dissochaetahirsutoidea***a** habit **b** branchlet **c** abaxial midrib **d** hypanthium and flower. Photographs by J. Henrot.

**Map 17.**
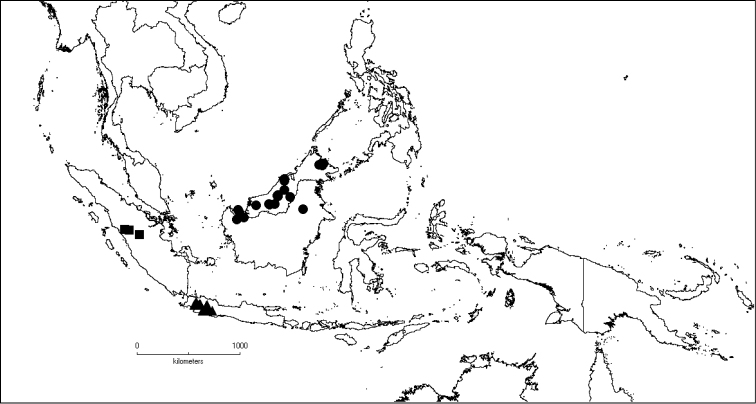
Distribution of *D.hirsutoidea* (●), *D.horrida* (■) and *D.intermedia* (▲).

##### Description.

Climbing up to 9 m in height. Branchlets terete, 3–5 mm in diameter, densely covered with dark thin bristle hairs; nodes swollen, with interpetiolar ridge; internodes 6–9 cm long. Leaves: petioles terete, 5–15 mm long, densely covered with bristle hairs; blades ovate or ovate-elliptic, 6–15.5 × 2–8.7 cm, membranous, base cordate, margin entire, ciliate, apex acuminate, tip 0.5–1 cm long; nervation with 1 or 2 pairs of lateral nerves and 1 pair of intramarginal nerves; adaxially hirsute, scabrid, glabrous or with scattered stellate and bristle hairs, abaxially densely covered with bristle hairs in most part, more dense at midrib and margin. Inflorescences terminal, up to 30 cm long, many-flowered; main axis angular, covered with sparsely minute stellate hairs and dense bristle hairs; primary axes up to 26 cm long with 4–6 nodes, secondary axes 1.5–9 cm long with 1–4 nodes, tertiary axes 0.8–2 cm long with 1 or 2 nodes, quarternary axes when developed up to 1 cm long with 1 node; bracts linear, 6–7 mm, covered with dense bristle hairs; bracteoles linear, 2–3 mm long, covered with dense bristle hairs; pedicels densely stellate-furfuraceous and with glandular bristle hairs, 3–4 mm long in central flowers, ca. 1 mm long in lateral flowers. Hypanthium campanulate, 4–5 × ca. 2 mm, densely covered with glandular capitate bristle hairs; calyx lobes truncate, ca. 1 mm long, apex triangular; petal bud conical, 2–3 mm long, apex bristly; mature petals oblong, 6–7 × 2–3 mm, reflexed, base clawed, apex obtuse, bristly, rest glabrous, white or purplish. Stamens 8, subequal, filaments curved sideways; alternipetalous stamens with ca. 5 mm long filaments, anthers lanceolate, sickle-shaped, thecae 8–9 mm long, purple, apex rostrate, pedoconnective 0.5–1 mm long, basal crest minute, triangular, 0.3–0.5 mm long, lateral appendages paired, auricles or filiform, 0.5–2 mm long; oppositipetalous stamens with 3–4 mm long filaments, anther thick, S-shaped, thecae 7–8 mm long, purple, basal crest minute, spur-like, erect, ca. 0.2 mm long, lateral appendages absent or a minute pair of erect auricles. Ovary half as long as the hypanthium, apex villous; style 6–7 mm long, curved at end, glabrous; stigma minute, capitate; extra-ovarial chambers 8, extending to the middle of the ovary. Fruits subglobose, 4–6 × 3–4 mm, densely covered with glandular bristle hairs; calyx lobe remnants persistent, reflexed. Seeds ca. 0.5 mm long.

##### Distribution.

Borneo.

##### Ecology and habitat.

Lowland mixed dipterocarp forest, open places or along road sides at 20–250 m elevation.

##### Specimens examined.

**MALAYSIA. Sabah**: Sandakan, Lamag Road, 13 Sep 1971, Imbungan & Patrick SAN 74187 (K); *Ibid.*, Segaliud Lokan, 17 Mar 1975, L. Madani SAN 81490 (K, L); *Ibid.*, Bettotan, 19 Aug 1927, C. Boden-Kloss SFN 19156 (BO, K); *Ibid.*, Sepilok Forest Reserve, 21 Sep 1963, W. Meijer SAN 34332 (K, L); *Ibid.*, Telupid Road, 20 Aug 1978, Aban Gibot SAN 91283 (K, L); *Ibid.*, Sungai Lantoh, 15 m, 23 Aug 1977, Saikeh SAN 87884 (K, L). **Sarawak**: Belaga, Ulu Belaga, Sungai Semawat, 250 m, 15 Oct 1981, C. Hansen 645 (L); *Ibid.*, Batang Belaga, 250 m, 29 Oct 1981, C. Hansen 878 (L); *Ibid.*, Sepakau, 250 m, 5 Nov 1981, C. Hansen 954 (L); Kapit, Bukit Raya, 14 Jan 1965, Jugah ak Kudi S.23863 (L); Lodong, G.D. Haviland 862 (K); Baleh, Mujong, Amau, 14 Apr 1964, Ilias Paie S.19868 (K, L); Kapit, Pelagus, Bukit Wong, 150 m, 20 Apr 1963, P.S. Ashton S.17786 (BO, K, L); Marudi, Sungai Silat Basin, Sungai Palutan, 24 Mar 2003, N. Normaya et al. S.91083 (L); Serian, Sabal Tapang, 237 m, 12 May 1974, S. Tong S.34270 (K, L); Santubong, 60 m, 20 Mar 1967, W.L. Chew 1428 (K, L); Samarahan, 1 Feb 1955, W.M.A. Brooke 9669 (BM, L); Long Bah, 14 Aug 1954, W.M.A. Brooke 9010 (L). **BRUNEI. Belait**: Labi, Between Mendaram and Teraja, 1 May 1988, K.M. Wong s.n. (K); *Ibid*., Bukit Teraja, 22 May 1993, N. Nangkat et al. BRUN 15203 (K); *Ibid.*, Bukit Telingan, 170 m, 7 Jun 1995, A. Kalat, et al. BRUN 16512 (L). **INDONESIA. East Kalimantan**: West Kutai, Long Petah, 400 m, 16 Sep 1925, F.H. Endert 3360 (BO, L). **West Kalimantan**: Pontianak, Gunung Bentuang, 200 m, 19 Jun 1989, J.S. Burley & Tukirin 2692 (BO, L); *Ibid.*, 250 m, 23 Jun 1989, J.S. Burley & Tukirin 2827 (BO, L).

#### 26. Dissochaeta horrida

Taxon classificationPlantaeMyrtalesMelastomataceae

(Bakh .f.) Karton. comb. nov.

urn:lsid:ipni.org:names:60476838-2

Fig. 18 , Map 17

Dissochaeta rostrata Korth. var. horrida (Bakh.*f.*) J.F.Maxwell, Gard. Bull. Sing. 33: 320. 1980.

##### Basionym.

*Macroleneshorrida* Bakh.*f.*, Contr. Melastom.: 208. 1943.

##### Type.

Indonesia. West Sumatra: Agam, Brani, 850 m elev., 22 Jun 1918, H.A.B. Bünnemeijer 3200 (holotype: L [L0537276]!; isotypes: BO [BO1751324, BO1751325]!).

**Figure 18.**
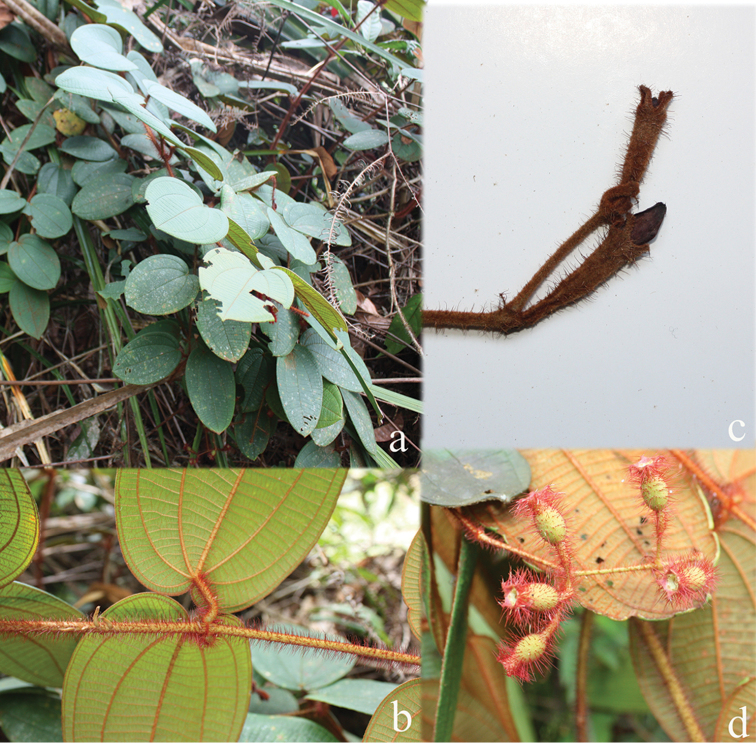
*Dissochaetahorrida***a** habit **b** branchlet **c** hypanthium **d** fruits. Photographs by A. Kartonegoro; vouchers: Kartonegoro 1073 (BO, L).

##### Description.

Climbing up to 2 m in height. Branchlets terete, 4–5 mm in diameter, densely covered with brown-reddish pubescent hairs and with 4–5 mm long brown-reddish bristle hairs, young parts densely covered with red pubescent hairs and bristle hairs; nodes swollen, with interpetiolar ridge; internodes 7–8 cm long. Leaves: petioles terete, 10–15 mm long, densely covered with brown-reddish pubescent hairs and with 4–5 mm long brown-reddish bristle hairs; blades broadly ovate to suborbicular, 13–18 × 6–10 cm, subcoriaceous, base emarginate, margin entire, apex acuminate, tip 0.5–1 cm long; nervation with 2 pairs of lateral nerves and 1 pair of intramarginal nerves; adaxially glabrous, dark green, abaxially densely brown-reddish pubescent. Inflorescences terminal, up to 60 cm long, many-flowered; main axis angular, densely covered with brown-reddish pubescent hairs and with 4–5 mm long brown bristle hairs; primary axes up to 34 cm long with 6 or 7 nodes, secondary axes up to 6 cm long with 2 or 3 nodes, tertiary axes up to 2 cm long with 1 or 2 nodes, quarternary axes when developed up to 1 cm long with 1 node; bracts linear, 7–9 mm long, densely covered with brown-reddish pubescent hairs and with 4–5 mm long brown-reddish bristle hairs; bracteoles subulate, ca. 2 mm long, densely covered with brown-reddish pubescent hairs and with 4–5 mm long brown-reddish bristle hairs; pedicels densely covered with brown pubescent hairs and with glandular tip bristle hairs, 6–7 mm long in central flowers, 2–3 mm long in lateral flowers. Hypanthium tubular, 7–8 × ca. 3 mm, densely covered with brown-reddish pubescent hairs and with brown-reddish glandular-tipped bristle hairs; calyx lobes linear-lanceolate, 6–10 mm long, base truncate, apex acute, densely covered with brown-reddish pubescent hairs and with glandular-tipped bristle hairs; petal bud conical, 3–4 mm long, glabrous; mature petals oblong to suborbicular, 6–8 × 5–6 mm, purple. Stamens 8, subequal, filaments curved sideways; alternipetalous stamens with 6–8 mm long filaments, anthers slender, curved, sickle-shaped, thecae 12–13 mm long, pedoconnective with 1–3 mm long, basal crest minute, triangular, ca. 0.5 mm long, lateral appendages paired, filiform, 1.5–2 mm long; oppositipetalous stamens with 5 mm long filaments, bent at the end, anthers curved, S-shaped, thecae 9–10 mm long, basal crest consisting of minute auricles, ca. 0.3 mm long, lateral appendages paired, auricles, 0.5–1 mm long. Ovary ¾ of hypanthium in length, apex villous; style 15–20 mm long, glabrous; stigma minute; extra-ovarial chambers 8, extending to the base of the ovary. Fruits urceolate, ca. 10 × 5–6 mm, densely covered with brown-reddish pubescent hairs and with glandular-tipped bristle hairs; calyx lobe remnants persistent, lanceolate, 6–8 mm long, reflexed. Seeds ca. 0.7 mm long.

##### Distribution.

Sumatra (West).

##### Ecology and habitat.

Lowland dipterocarp forest or open cliff area at 500–850 m elevation.

##### Note.

*Dissochaetahorrida* is easy to recognise because most parts have reddish brown pubescent hairs and dense bristle hairs except the green, glabrous adaxially surface of the leaf blades. The shape of the calyx lobes (linear-lanceolate, 6–10 mm long) resembles *D.floccosa*, also from Sumatra, but the indumentum of the latter is floccose rather than pubescent.

##### Specimens examined.

**INDONESIA. Riau**: Indragiri, Taluk, 11 Jan 1956, W. Meijer 4271 (L). **West Sumatra**: Agam, Brani, 850 m, 22 Jun 1918, H.A.B. Bünnemeijer 3200 (BO, L); Lima Puluh Kota, Harau Valley, Sarasah Bonta, 500 m, 2 May 2001, Uce et al. 106 (ANDA); *Ibid.*, 11 Sep 2017, A. Kartonegoro 1073 (BO, L).

#### 27. Dissochaeta inappendiculata

Taxon classificationPlantaeMyrtalesMelastomataceae

Blume, Flora 14: 499. 1831.

Fig. 19 , Map 18

Dissochaeta inappendiculata Blume var. purpurascens Blume, Flora 14: 499. 1831. Type: Indonesia. West Java: Preanger Regentsch., Mount Megamendoeng, H. Kuhl & J.C. van Hasselt s.n. (holotype: L [L0537245]!).
Dissochaeta inappendiculata Blume var. tomentosa Blume, Flora 14: 499. 1831. Type: Indonesia. West Java: Rendang, F.W. Junghuhn s.n. (holotype: L [L0537237]!).
Dissochaeta cinnamomea Blume, Mus. Bot. 1(3): 36. 1849. Type: Indonesia. Java, C.L. Blume s.n. (lectotype, designated here: L [L0537241]!; isolectotype: L [L0537240]!).
Dissochaeta vacillans auct. non Blume: Veldkamp, Blumea 24: 440. 1979. *p.p.*, excl. type.

##### Type.

Indonesia. Java, C.L. Blume s.n. (lectotype, designated here: L [L0537236]!; isolectotypes: K [K000859623]!, L [L0537235]!, P [P05283569, image seen]!).

**Figure 19.**
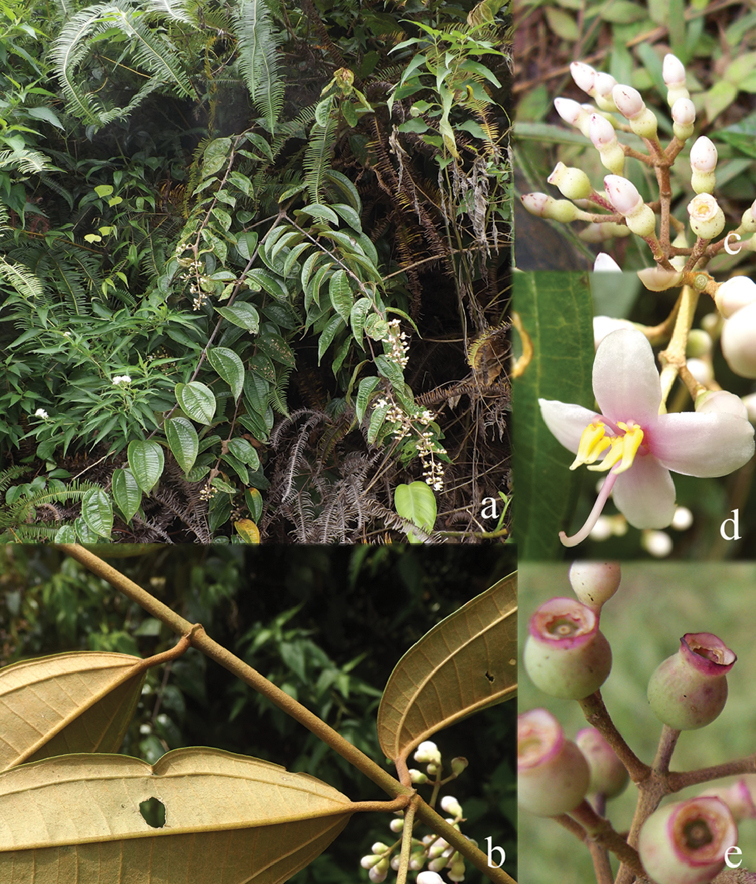
*Dissochaetainappendiculata***a** habit **b** branchlet **c** hypanthium **d** flower **e** fruits. Photographs by A. Kartonegoro; vouchers: Kartonegoro 1104 (BO, L).

**Map 18.**
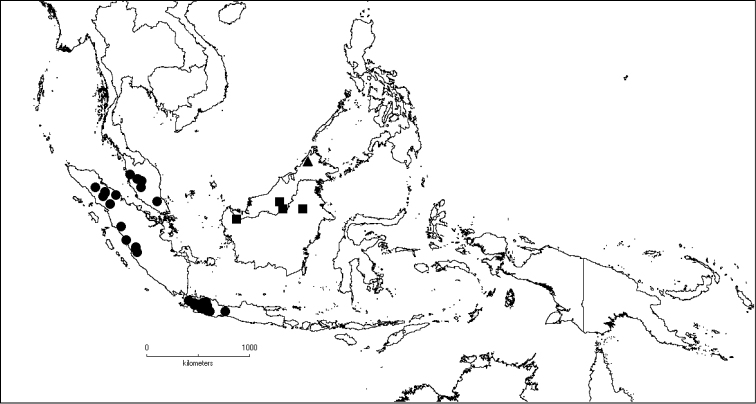
Distribution of *D.inappendiculata* (●), *D.laevis* (■) and *D.macrosepala* (▲).

##### Description.

Climbing up to 20 m in height. Branchlets terete, 3–5 mm diameter, glabrous or glabrescent or sometimes with stellate-hairs; nodes swollen, with interpetiolar ridge; internodes 6–9 cm long. Leaves: petioles terete or flattened, 5–18 mm long, glabrous, rarely furfuraceous; blades elliptic to oblong, (5–)7–14 × (1–)3–6 cm, membranous, base rounded, margin entire, apex acute or acuminate, tip 0.5–2 cm long; nervation with 1 pair of lateral nerves and 1 pair of intramarginal nerves; adaxially glabrous, abaxially densely brown stellate-furfuraceous. Inflorescences terminal, up to 34 cm long, many-flowered; main axis angular, stellate-puberulous or stellate-furfuraceous; primary axes 22.5–32 cm long with 8–10 nodes, secondary axes 2–8 cm long with 2–5 nodes, tertiary axes 1–2.5 cm long with 1 or 2 nodes, quarternary axes when developed 0.4–0.8 cm long with 1 node; bracts linear to lanceolate, (0.7–)2–3.5 × 1–1.5 mm, caducous, glabrescent; bracteoles linear, 1–2 mm long, glabrescent or stellate-furfuraceous, sometimes with bristly hairs along the margin; pedicels brown stellate-puberulous, 4–6 mm long in central flowers, 2–4 mm long in lateral flowers. Hypanthium campanulate to suburceolate, 1–4 × 1–3 mm, glabrescent to densely furfuraceous or stellate-furfuraceous; calyx lobes truncate with 4 undulate, 0.5–1 mm long, rounded, or subtriangular apices, glabrous, purplish; petal bud conical to subrounded, 1–5 mm long; mature petals obovate, 4–6 × 2–3 mm, not reflexed, apex acute, base clawed, glabrous or inside with appressed hairs at base, purple to purplish-white. Stamens 8, unequal, filaments straight, pink; alternipetalous stamens with 2–3.5 mm long filaments, anthers oblong or lanceolate, thecae 3–4 mm long, straight, yellow, pedoconnective not formed or absent, basal crests triangular or ovate, ca. 1 mm long, with acute narrow apex, bright white, lateral appendages absent; oppositipetalous stamens staminodal, with 1.5–2 mm long filaments, thecae 1–1.5 mm long, clavate, straight, yellow, basal crests spuriform, ca. 0.5–0.8 mm long, erect, lateral appendages absent. Ovary ⅔ of hypanthium in length, apex puberulous; style 5–8 mm long, curved at apex, glabrous, purple; stigma minute; extra-ovarial chambers 4, reaching to the middle of the ovary. Fruits subglobose to urceolate, 2–5(–7) × 2–5 mm, glabrous to sparsely stellate-furfuraceous; calyx lobes persistent, erect. Seeds ca. 0.5 mm long.

##### Distribution.

Peninsular Malaysia, Sumatra and Java.

##### Ecology and habitat.

Primary montane forest or secondary forest, edge of forest with open shaded area at 400–1800 m elevation.

##### Vernacular names.

Java: *harendong*, *harendong cai* (Sunda).

##### Note.

*Dissochaetainappendiculata* is easily recognised by its purple corolla, eight straight stamens of which the alternipetalous ones are fertile and the oppositipetalous ones are staminodal. The alternipetalous stamens are always inappendiculate and usually without lateral appendages and with a triangular or orbicular crest. The oppositipetalous (less than ⅓ of the alternipetalous ones) have a distinct spuriform crest (Kartonegoro and Veldkamp 2010).

##### Selected specimens examined.

**MALAYSIA. Pahang**: Cameron Highlands, 1600 m, 14 Apr 1978, J.F. Maxwell 78-139 (L); *Ibid.*, Tanah Rata, Robinson’s Fall, 11 Sep 1985, A. Latiff, A. Zainudin & S. Miran 859 (L); Fraser’s Hill, 19 Jun 1967, J.C. Carrick 1574 (K, L); *Ibid.*, 1000 m, 1 Apr 2000, J.J. Vermeulen & H. Duistermaat 2023 (L); Telom, Nov 1890, H.N. Ridley 13686 (BM). **Perak**: Maxwell’s Hill, 4 Mar 1965, Hardial & Samsuri 278 (K, L). **INDONESIA. Aceh**: Kutacane, Gunung Gurah, 22 Mar 1954, A.H.G. Alston 14628 (BM). **Jambi**: Mt. Kerinci, Kayu Aro, 1400 m, 9 Aug 1956, M. Jacobs 4550 (BO, K); *Ibid.*, Sungai Penuh, 3 Jun 1914, H.C. Robinson & C. Boden-Kloss s.n. (BM, K). **North Sumatra**: H.S. Yates 1424 (BM); Toba, Siburong-Burong, 1200 m, 23 Jun 1896, C.D. Ouwehand s.n. (BO); Berastagi, 1300 m, 29 Aug 1971, K. Iwatsuki et al. S-405 (BO); Dairi, 1400 m, 1939, T.W.G. Dames 72 (BO); Karo, Sikulikap Waterfall, 1200 m, 6 Mar 1973, Soedarsono 432 (BO); Sibolangit, 600 m, 12 Sep 1920, J.A. Lörzing 7351 (BO). **West Sumatra**: Mt. Kerinci, 1700 m, 16 Mar 1920, H.A.B. Bünnemeijer 8937 (BO); Ophir, Talu, 950 m, 10 Apr 1917, H.A.B. Bünnemeijer 124 (BO); Padang, Air Sirah,, 1000 m, 2 May 1985, E.F. de Vogel & J.J. Vermeulen 7312 (BO). **Banten**: Pandeglang, Mt. Pulosari, 1050 m, 11 Feb 1954, A.G.L. Adelbert 476 (BO, K, L); *Ibid.*, 762 m, 11 May 1843, H. Zollinger 1288 (P). **Central Java**: Banyumas, Mt. Slamet, Baturaden, 1200 m, 14 Apr 1911, C.A. Backer 296 (BO). **West Java**: Bogor, Pasir Madang, 13 Aug 1843, H. Zollinger 1489 (BM, P); Nanggung, 600 m, 23 Dec 1913, C.A. Backer 10522 (BO); Mt.Salak, Gunung Bunder, 8 Aug 1909, C.A. Backer s.n. (BO); Mt.Halimun, Nirmala, 1400 m, 19 Dec 1913, C.A. Backer 10794 (BO); *Ibid.*, Malasari, 1055 m, 10 Oct 2017, A. Kartonegoro 1104 (BO, L); Mount Gede, Situ Gunung, 1000 m, 19 Nov 1933, C.G.G.J. van Steenis 5685 (BO); Cianjur, Mt. Gede, 1200 m, Oct 1877, L. Pierre 3032 (P); Cibeber, Cidadap, 1100 m, 11 Sep 1917, C.A. Backer 23703 (BO); Bandung, Mt. Jayagiri, 1460 m, 26 Mar 1920, H.J. Lam 141 (BO, L); Mt. Malabar, 2130 m, 19 Oct 1861, T. Anderson 92 (K); *Ibid.*, 26 Mar 1880, H.O. Forbes 1047 (P); Mt. Sembung, 1300 m, 18 Mar 1914, C.A. Backer 12256 (BO); Garut, Mt. Cikuray, Pasir Klotok, 1000 m, 15 Aug 1913, C.A. Backer 8687 (BO).

#### 28. Dissochaeta intermedia

Taxon classificationPlantaeMyrtalesMelastomataceae

Blume, Flora 14: 493. 1831.

Map 17

Dissochaeta monticola Blume, Flora 14: 494. 1831. Type: Indonesia. West Java: Kuripan, C.L. Blume s.n. (lectotype, designated here: L [L0537300]!; isolectotypes: L [L0537301, L0537302]!).

##### Type.

Indonesia. West Java: Preanger Regentsch., Mount Pangrango, Gegerbentang, C.L. Blume 539 (lectotype, designated here: L [L0537299]!; isolectotypes: K [K000859493, K000859494]!, L [L0537296, L0537297, L0537298]!, P [P05283548, image seen]!).

##### Description.

Climbing up to 20 m in height. Branchlets terete, 4–7 mm diameter, glabrescent to densely stellate-furfuraceous; nodes swollen, with interpetiolar ridge or line; internodes 8–9 cm long. Leaves: petioles flattened, 10–20 mm long, glabrescent to densely stellate-furfuraceous; blades ovate to ovate-elliptic, 7–16 × 5–8.5 cm, membranous, base rounded, margin entire, apex acuminate, tip 0.5–2 cm long; nervation with 1 pair of lateral nerves and 1 pair of intramarginal nerves; adaxially glabrous, abaxially densely brown stellate-tomentose. Inflorescences terminal and in the upper leaf axils, up to 32 cm long, many-flowered; main axis angular, densely stellate-furfuraceous; primary axes up to 25–30 cm long with 4–6 nodes, secondary axes 2–7 cm long with 2 or 3 nodes, tertiary axes 1–3 cm long with 1 or 2 nodes, quartenary axis up to 0.8 cm long when developed with 1 node; bracts minute, inconspicuous; bracteoles linear to ovate-oblong, 1–2 mm long, densely stellate-furfuraceous; pedicels densely stellate-furfuraceous, 4–7 mm long in central flowers, 2–4 mm long in lateral flowers. Hypanthium campanulate, slightly angular, 2–5 × 1–3 mm, with four distinct ridges, stellate-puberulous to densely stellate-tomentose, somewhat pointing sideways from pedicels; calyx lobes truncate with undulate tips, 0.5–1 mm long, widened, stellate-furfuraceous; petal bud conical 3–7 mm long; mature petals ovate or oblong, 6–10 × 3–5 mm, base clawed, apex obtuse, glabrous or inside at base with appressed hairs, pink or violet. Stamens 4, equal, alternipetalous, filaments flat, 4–6 mm long, straight, apex bent, anthers linear-lanceolate, sickle- or S-shaped or falcate, thecae 5–6 mm long, yellow, pedoconnective ca. 1 mm long, basal crests minute, triangular, ca. 1 mm long, narrow with acute apex, lateral appendages paired, filiform, 3–4 mm long, tan. Ovary half or ⅔ of hypanthium in length, apex pubescent; style erect, 8–12 mm long, curved at apex, glabrous, purple; stigma minute; extra-ovarial chambers 4, alternipetalous, extending to the base of the ovary. Fruits ovoid, 5–6 × 3–5 mm, glabrous to sparsely stellate-furfuraceous, rarely with distinct vertical ridges, green, often pointing sideways from the pedicels; calyx lobe remnants persistent. Seeds ca. 0.5 mm long.

##### Distribution.

Java (West).

##### Ecology and habitat.

Primary montane forest or in edge of forest in open, 1200–2000 m elevation.

##### Vernacular names.

*harendong cai*, *harendong areuy*, *harendong kowe* (Sunda).

##### Note.

*Dissochaetaintermedia* resembles *D.leprosa* in the number and shape of the stamens, but is different in having a smaller hypanthium and truncate calyx lobes with undulate tips. Together with *D.leprosa*, this species is common at high elevations up to 2000 m in Java.

##### Specimens examined.

**INDONESIA. West Java**: Bogor, Puncak Pass, 1300 m, 3 Mar 1927, J.G.B. Beumée A390 (BO); *Ibid.*, Cisarua, 1200 m, 19 Feb 1950, C.G.G.J. van Steenis 12735 (L); Mt. Salak, 1200 m, H. Raap 158 (L); Kuripan, C.L. Blume s.n. (L); Megamendung, H. Kuhl & J.C. van Hasselt s.n. (L); *Ibid.*, F.W. Junghuhn s.n. (U); Mt.Halimun, Nirmala, 1250 m, 9 Jun 1980, M.M.J. van Balgooy & H. Wiriadinata 2889 (BO, L); *Ibid.*, Mt. Bintang Gading, 19 May 2002, W.S. Hoover et al. 5502 (BO); Cianjur, Mt.Gede, Cibodas, J.G. Boerlage s.n. (L); *Ibid.*, 1400 m, 22 Aug 1879, Arsin 19556 (BO); *Ibid.*, 9 Jun 1906, A.A. Pulle 4073 (U); *Ibid.*, 19 Jul 1913, S.H. Koorders 42153β (BO); *Ibid.*, 6 May 1914, J.A. Lörzing 1493 (BO); *Ibid.*, 14 Feb 1915, H.N. Ridley s.n. (K); *Ibid.*, Gunung Putri, 6 Nov 1896, S.H. Koorders 25927β (BO); *Ibid.*, Sindanglaya, 1400 m, 6 Jun 1917, C.A. Backer 22286 (BO); *Ibid.*, J.G. Hallier 626 (BO); *Ibid.*, 25 Aug 1893, J.G. Hallier 432 (BO); *Ibid.*, 1915, Sapiin 57 (BO); *Ibid.*, 15 Jul 1908, T. Valeton s.n. (BO); *Ibid.*, Mt. Pangrango, P. de Monchy s.n. (BO); *Ibid.*, Geger Bentang, C.L. Blume 539 (K, L, P); Bandung, Mt. Tangkuban Perahu, 1700 m, 4 Apr 1912, C.A. Backer 2387 (BO); *Ibid.*, 1600 m, 5 Mar 1912, C.A. Backer 2415 (BO); *Ibid.*, Jul 1915, W.M. Docters van Leeuwen 2301a (BO); *Ibid.*, 24 Jul 1927, W.M. Docters van Leeuwen 11423 (BO); *Ibid.*, 2000 m, 21 Nov 1952, W. Meijer 1363 (BO); *Ibid.*, 28 May 1908, H.H. Zeijlstra 19 (BO); *Ibid.*, 6 Feb 1927, C.A. Wisse 1188 (BO); Mt. Patuha, Telaga Patengan, 1700 m, 28 Mar 1914, C.A. Backer 12787 (BO); Mt. Burangrang, Situ Lembang, 1600 m, 24 Jul 1920, R.C. Bakhuizen van den Brink 4557 (BO, K, L); *Ibid.*, 30 Dec 1956, S. Reksodihardjo 6 (BO); Mt. Malabar, 27 Jun 1871, R.H.C.C. Scheffer s.n. (BO); Mt. Mandalagiri, 1570 m, 29 Mar 1920, H.J. Lam 230 (BO).

#### 29. Dissochaeta johorensis

Taxon classificationPlantaeMyrtalesMelastomataceae

Furtado, Gard. Bull. Singapore 20: 110. 1963.

Map 19

Dissochaeta hirsuta auct. non Hook.*f.* ex Triana; King, J. Asiat. Soc. Bengal, Pt. 2, Nat. Hist. 69(2): 51. 1900; Ridl., Fl. Malay Penins. 1: 797. 1922. *p.p.*, excl. type.

##### Type.

Malaysia. Johor: Gunong Panti, 1892, H.N. Ridley 4185 (holotype: SING *n.v.*; isotypes: BM [BM000944478]!, K [K000859526]!).

**Map 19.**
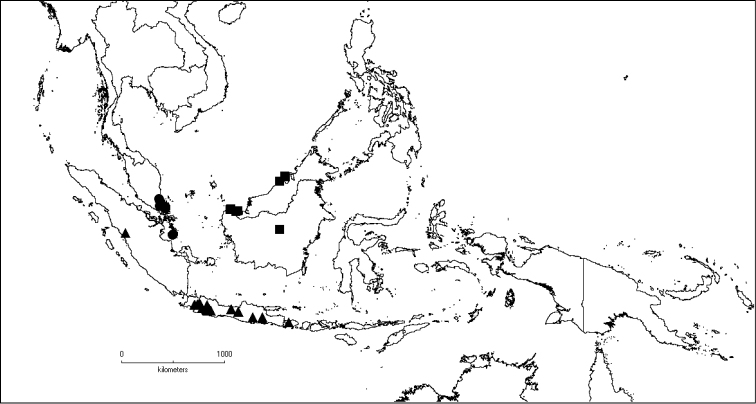
Distribution of *D.johorensis* (●), *D.latifolia* (■) and *D.leprosa* (▲).

##### Description.

Climbing up to 4.5 m in height. Branchlets terete, 2–4 mm in diameter, densely covered with brown stellate-furfuraceous hairs and 1–2 mm long bristle hairs; nodes swollen, with interpetiolar ridge; internodes 3.5–7.5 cm long. Leaves: petioles terete, 6–10 mm long, densely covered with stellate-furfuraceous and bristle hairs; blades ovate or elliptic, 7–10 × 2.8–6 cm, membranous, base emarginate, margin entire, ciliate, apex acuminate, tip 1–1.5 cm long; nervation with 1 pair of lateral nerves and 1 pair of intramarginal nerves; adaxially hirsute to scabrid with scattered bristle hairs, abaxially densely covered with bristle hairs in most part, more densely so at midrib and margin. Inflorescences terminal, up to 25 cm long, many-flowered; main axis angular, densely covered with brown stellate-furfuraceous and bristle hairs; primary axes up to 21 cm long with 5 or 6 nodes, secondary axes 2–6 cm long with 1–3 nodes, tertiary axes up to 1 cm long with 1 node; bracts linear, ca. 4 mm long, densely covered with stellate-furfuraceous and bristle hairs; bracteoles linear, ca. 2 mm long, densely covered with stellate-furfuraceous and bristle hairs; pedicels densely covered with stellate-furfuraceous and bristle hairs, ca. 4 mm long in central flowers, 1–2 mm long in lateral flowers. Hypanthium suburceolate, 5–6 × 2–2.5 mm, densely covered with stellate-furfuraceous hairs and glandular bristle hairs, dark green; calyx lobes slightly triangular, 1–2 mm long, tips acute, densely bristly, pinkish; petal bud conical, 3–4.5 mm long, apex glabrous; mature petals obovate to oblong, 7–8 × ca. 3 mm, not-reflexed, base clawed, apex acute, glabrous, pink or violet. Stamens 8, subequal, filaments curved sideways, pale yellow; alternipetalous stamens with 6–7 mm long filaments, anthers lanceolate, sickle-shaped, thecae 10–11 mm long, apex rostrate, white-cream, pedoconnective 1–1.5 mm long, basal crests minute, triangular, ca. 0.5 mm long, lateral appendages paired, filiform, up to 3 mm long, cream; oppositipetalous stamens with 6–7 mm filaments, anther thick, S-shaped, thecae 9–10 mm long, white-cream, basal crests minute, laminar or spur-like, ca. 0.5 mm long, lateral appendages small, bifid, ca. 0.3 mm long or paired and filiform, 2–2.5 mm long. Ovary ⅔ of hypanthium in length, apex villous; style curved at end, 9–12 mm long, glabrous, white; stigma minute, capitate; extra-ovarial chambers 8, extending to the middle of the ovary. Fruits urceolate, 8–10 × 4.5–5 mm, sparsely stellate-furfuraceous and densely covered with glandular bristle hairs; calyx lobe remnants persistent, reflexed. Seeds ca. 0.5 mm long.

##### Distribution.

Peninsular Malaysia (Johor) and Sumatra (Riau Archipelago).

##### Ecology and habitat.

Edge of evergreen forest in open thickets and lowland forest at 10–200 m elevation.

##### Note.

*Dissochaetajohorensis* resembles *D.rostrata* in its indumentum of dense bristle hairs on most parts, but differs by its acute, triangular calyx lobes (vs. obtuse, ovate lobes) and urceolate fruits (vs. subglobose to ovoid fruits). The shape of the stamens and the appendages in both species are also different.

##### Specimens examined.

**MALAYSIA. Johor**: Kota Tinggi, Gunong Panti, 1892, H.N. Ridley 4185 (BM, K); *Ibid.*, 1880, H. Kunstler 197 (K, P); *Ibid.*, 200 m, 8 Apr 1977, J.F. Maxwell 77-181 (L); *Ibid.*, 7 Feb 1978, J.F. Maxwell 78-31 (L); *Ibid.*, 50 m, 8 Dec 1979, J.F. Maxwell 79-48 (L); *Ibid.*, 4 Jan 1980, K. Bremer 1840 (K); Kluang FR., 60 m, 31 Jan 1966, F.S.P. Ng KEP 97965 (K); *Ibid.*, 24 Nov 1967, Alphonso, Sanusi & Sidek S.197 (K, L); Endau Rompin, Kuala Jasin, 100 m, 1 Mar 1996, M.M.J. van Balgooy 7114 (L); Lombong FR, 30 m, 22 May 1959, H.M. Burkill HMB 1822 (K, L); Sungai Kayu, 9 Mar 1937, Kiah SFN 32357 (BM, K, PNH); Gunong Muntahak, 183 m, 3 Mar 1928, M. Nur SFN 19975 (BM, BO, K); Labis, Sungei Kinchin, 30 m, 25 Aug 1988, L.G. Saw FRI 36361 (L). **INDONESIA. Riau Archipelago**: Lingga Islands, Singkep Island, Kampung Raya, 10 m, 1 Aug 1919, H.A.B. Bünnemeijer 7096 (BO); *Ibid.*, Manggu, 40 m, 2 Aug 1919, H.A.B. Bünnemeijer 7175 (BO); *Ibid.*, Dabo, 40 m, 4 Aug 1919, H.A.B. Bünnemeijer 7276 (BO).

#### 30. Dissochaeta laevis

Taxon classificationPlantaeMyrtalesMelastomataceae

Ohwi ex J.F.Maxwell, Gard. Bull. Singapore 33: 315, fig. 2. 1980 (“ laeve”).

Map 18

##### Type.

Indonesia. East Kalimantan: W. Koetai, Long Petah, 600 m elev., 10 Sep 1925, F.H. Endert 3127 (holotype: L [L0537281]!; isotypes: BO [BO1760872]!, K [K000859490]!).

##### Description.

Climbing up to 6 m in height. Branchlets terete, 3–4 mm in diameter, glabrescent; nodes swollen, with short flat crest-like interpetiolar ridge, margin often with minute bristles; internodes 5–6 cm long. Leaves: petioles terete, 8–10 mm long, glabrous, tessellate; blades ovate to ovate-elliptic, 6–12 × 2.3–5.5 cm, membranous, base rounded, margin entire, apex acuminate, tip 0.5–1 cm long; nervation with 1 pair of lateral nerves and 1 pair of intramarginal nerves; surfaces glabrous, basally with a pair of glandular patches abaxially. Inflorescences terminal and axillary, up to 11 cm long when terminal, up to 9 cm long when axillary, many-flowered; main axis glabrescent; primary axes up to 8 cm long with 3 or 4 nodes, secondary axes 2–4.5 cm long with 2 or 3 nodes, tertiary axes 0.6–1 cm long with 1 node; bracts linear, 1–2 mm long, stellate-furfuraceous; bracteoles subulate, 0.5–1 mm long, stellate-furfuraceous; pedicels stellate-furfuraceous, ca. 4 mm long in central flowers, 2–3 mm long in lateral flowers. Hypanthium cyathiform, cup-shaped, 3–4 × ca. 2 mm, glabrous; calyx lobes truncate, ca. 0.3 mm long, with 4 distinct undulations; petal bud conical, 2–3 mm long, apex narrowly acuminate, glabrous; mature petals ovate, ca. 5 × 2.5 mm, reflexed, base clawed, apex acuminate, glabrous. Stamens 8, subequal, filaments straight; alternipetalous stamens with 2.5–3 mm long filaments, anthers oblong or ovate, curved, sickle-shaped, thecae ca. 3 mm long, pedoconnective short, ca. 0.3 mm long, basal crest triangular or ligular with hastate base, ca. 1.5 mm long, lateral appendages absent or paired, filiform, up to 1.5 mm long; oppositipetalous stamens with 2–2.5 mm long filaments, anthers sickle- or S-shaped, thecae ca. 2.5 mm long, basal crest ligular, ca. 2 mm long, lateral appendages absent. Ovary half as long as hypanthium, apex glabrous; style straight, 7–8 mm long, glabrous; stigma minute; extra-ovarial chambers shallow. Fruits globose, 3–5 × 3–4 mm, glabrous; calyx lobe remnants persistent. Seeds ca. 0.5 mm long.

##### Distribution.

Borneo.

##### Ecology and habitat.

Mixed dipterocarp forest or heath forest at 600–800 m elevation.

##### Note.

A glabrous species with a pair of glandular patches near the base of the leaf blade on the abaxially surface, resembling *D.beccariana*, *D.glabra*, *D.glandulosa* and *D.papuana*. It differs from these taxa by the axillary inflorescences.

##### Specimens examined.

**MALAYSIA. Sarawak**: Belaga, Linau-Balui, Sungei Jelini, 800 m, 1 Sep 1978, B. Lee S. 39326 (K, L); Kapit, Batang Balui, Bukit Kumbong, 700 m, 27 Feb 1992, Runi et al. S.62014 (K, L). **INDONEISA. East Kalimantan**: West Kutai, Long Petah, 600 m, 10 Sep 1925, F.H. Endert 3127 (BO, K, L). **West Kalimantan**: Pontianak, Bentiang, Gunung Dawuh, 800 m, 29 Oct 1980, G. Shea 26846 (BO, L).

#### 31. Dissochaeta latifolia

Taxon classificationPlantaeMyrtalesMelastomataceae

(Triana) Karton. comb. nov.

urn:lsid:ipni.org:names:60476839-2

Map 19

Diplectria latifolia (Triana) Kuntze, Revis. Gen. Plant 1: 246. 1891.

##### Basionym.

*Anplectrumlatifolium* Triana, Trans. Linn. Soc. London 28: 85. 1872.

##### Type.

Malaysia. Borneo, 1853, T. Lobb s.n. (lectotype, designated by Veldkamp et al. 1979, pg. 412: K [K000859553]!).

##### Description.

Climbing up to 9 m in height. Branchlets terete, 5–6 mm in diameter, glabrous to covered with brown stellate-furfuraceous hairs; nodes swollen, with pectinate or subulate interpetiolar appendages, apex acute, up to 10 mm long and 1 mm wide, pointing upwards and downwards; internodes 10–16 cm long. Leaves: petioles terete, 15–25 mm long, densely covered with brown stellate-furfuraceous hairs; blades suborbicular to ovate, 17–19 × 8.5–10 cm, subcoriaceous, base slightly subcordate, margin entire, apex acute; nervation with 1 or 2 pairs of lateral nerves and 1 pair of intramarginal nerves; adaxially glabrous, glossy green, abaxially sparsely brown stellate-furfuraceous to glabrescent. Inflorescences terminal, up to 20 cm long, many-flowered; main axis angular, densely stellate-furfuraceous; primary axes up to 18 cm long with 4 or 5 nodes, secondary axes up to 4 cm long with 2 or 3 nodes, tertiary axes up to 0.8 cm long with 1 or 2 nodes; bracts linear, 4–6 mm long, densely stellate-furfuraceous; bracteoles subulate, 1–3 mm long, densely stellate-furfuraceous; pedicels densely stellate-furfuraceous, often with a few scattered capitate bristles, 5–6 mm long in central flowers, 2–3 mm long in lateral flowers. Hypanthium tubular, ca. 6 × 2 mm, glabrous to glabrescent; calyx lobes truncate, level, ca. 0.5 mm long; petal bud conical, 3–4 mm long, apex acuminate and bristly; mature petals suborbicular, 6–7 × ca. 6 mm, base clawed, apex acuminate, pink. Stamens 8, unequal, filaments straight; alternipetalous stamens staminodial with 2–3 mm long filaments, anthers rudimentary, terete, thecae 2–3 mm long, basal crest triangular or ovate, 1–2 mm long, apex acuminate, lateral appendages paired, ligular, ca. 2 mm long; oppositipetalous stamens with 4–5 mm long filaments, anthers thick, curved, hook- or S-shaped, thecae 10–11 mm long, apex rostrate, yellow, basal crest ligular, ca. 1 mm long, lateral appendages absent or minute, bifid, ca. 0.5 mm long. Ovary ⅔ of hypanthium in length, apex glabrous; style curved at the end, 8–10 mm long, glabrous, slender; stigma minute; extra-ovarial chambers 4, oppositipetalous, extending to the base of the ovary. Fruits urceolate, 8–9 × ca. 6 mm, glabrous; calyx lobe remnants persistent, erect. Seeds ca. 0.75 mm long.

##### Distribution.

Borneo.

##### Ecology and habitat.

Lowland mixed dipterocarp forest or heath forest at 45–150 m elevation.

##### Note.

*Dissochaetalatifolia* resembles *D.pubescens* in the tomentose indumentum of the branchlets, abaxially leaf surfaces and inflorescences. The size and shape of the leaves of both species are also similar, sometimes resulting in a misidentification. *Dissochaetalatifolia* is different in not having a membranous calyptra that encloses the petal bud before anthesis. The pectinate and subulate appendages in the interpetiolar nodal ring are also distinct in *D.latifolia*, making it easy to recognise amongst all species of *Dissochaeta*.

##### Specimens examined.

**MALAYSIA. Sarawak**: O. Beccari PB 800 (K, P); O. Beccari PB 1089 (K); T. Lobb s.n. (K); Miri, Lambir Hills, 152 m, 10 Jun 1961, D. Bakar S.4367 (K, L); *Ibid.*, 5 Dec 1962, Joseph Au S.17256 (L); Kuching, Semariang Batu, 25 Jun 1976, Bagong et al. S.37642 (K, L); Lundu, Sematan, Pueh, Sungai Kopak, 200 m, 20 Aug 1996, R. Jawa & S.H. Lai S.74524 (L). **BRUNEI. Belait**: Sungai Liang, 1 Feb 1989, N. Nangkat 89 (K, L); Andulau, 45 m, 24 Apr 1957, P.S. Ashton S.5922 (K, L). **INDONESIA. Central Kalimantan**: Barito Ulu, 24 Jun 1990, C.E. Ridsdale PBU 647 (BO, K, L).

#### 32. Dissochaeta leprosa

Taxon classificationPlantaeMyrtalesMelastomataceae

(Blume) Blume, Flora 14: 494. 1831.

Fig. 20 , Map 19

Melastoma leprosum Blume, Bijdr. Fl. Ned. Ind 17: 1068. 1826.
Omphalopus leprosus (Blume) Naudin, Ann. Sci. Nat., Bot. sér. 3, 15: 278. 1851.
Dissochaeta calothyrsa Miq., Fl. Ned. Ind. 1(1): 523. 1855. Type: Indonesia. West Java: Pengalengan, F.W. Junghuhn 13 (lectotype, designated here: U [U0004009]!; isotype: L [L0822682, L0822683, L0822684]!).
Dissochaeta intermedia Blume var. leprosa (Blume) J.F.Maxwell, Gard. Bull. Singapore 33: 315. 1980.

##### Type.

Indonesia. West Java: Mount Gede, H. Kuhl & J.C. van Hasselt s.n. (lectotype, designated here: L [L0008890]!; isolectotype: K [K000859492]!, L [L0822675, L0822676]!).

**Figure 20.**
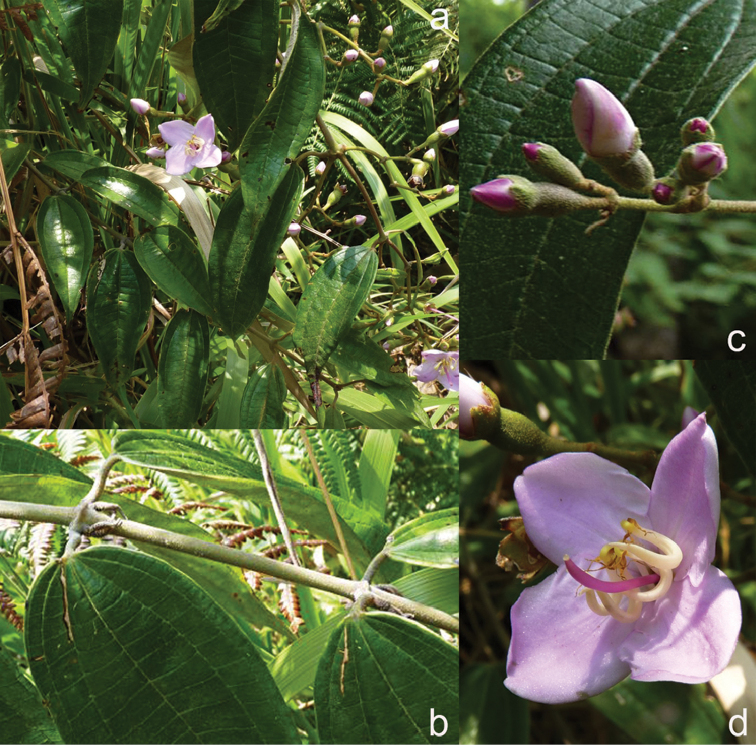
*Dissochaetaleprosa***a** habit **b** branchlet **c** hypanthium **d** flower. Photographs by C. Bravard.

##### Description.

Climbing up to 25 m in height. Branchlets terete, 4–6 mm in diameter, densely stellate-furfuraceous to stellate-tomentose; nodes swollen, with interpetiolar ridge; internodes 11–13 cm long. Leaves: petioles flattened, 12–18 mm long, densely stellate-furfuraceous; blades ovate-elliptic or elliptic, 8–17 × 4.8–8 cm, membranous, base rounded or subcordate, margin entire, apex acuminate, tip 0.5–1.5 cm long; nervation with 1 or 2 pairs of lateral nerves and 1 pair of intramarginal nerves; adaxially glabrous, abaxially densely stellate-tomentose. Inflorescences terminal, up to 27 cm long, many-flowered; main axis angular, densely stellate-tomentose; primary axes up to 22 cm long with 3 or 4 nodes, secondary axes 4–5 cm long with 2 nodes, tertiary axes 1.2–1.5 cm long with 1 node; bracts lanceolate, ca. 10 × 2 mm, densely stellate-tomentose, caducous; bracteoles linear or lanceolate, 1–6 mm long, densely stellate-pubescent; pedicels densely stellate-tomentose, 7–8 mm in central flowers, 3–5 mm long in lateral flowers. Hypanthium campanulate or suburceolate, 5–8 × 3–4 mm, slightly angular, covered with densely stellate-tomentose hairs; calyx lobes truncate with distinctly triangular tips, 3–4 mm long, erect, densely stellate-tomentose; petal buds conical, 7–10 mm long, apex acute; mature petals obovate or ovate, 6–10 × 3–8 mm, base clawed, apex obtuse, glabrous or hairy at base inside, pink or white purplish. Stamens 4, equal, alternipetalous, filaments flat, 4–8 mm long, straight, apex bent, anthers glabrous, linear or lanceolate, falcate or S-shaped, thecae 8–12 mm long, pedoconnective 1–2 mm long, basal crests distinctly triangular, 1–1.5 mm long, narrow with acute apex, lateral appendages paired, flat, filiform, 4–7 mm long, sometimes divided at the apex. Ovary half or ⅔ of hypanthium in length, apex pubescent; style 8–15 mm long, apex curved, glabrous; stigma capitate; extra-ovarial chambers 4, alternipetalous, extending to the base of the ovary. Fruits ovoid or urceolate, 7–12 × 5–6 mm, stellate-puberulous or nearly stellate-tomentose, greenish when unripe, with 4 distinct vertical ridges; calyx lobe remnants persistent, 2–3 mm long; stalks densely stellate-furfuraceous, 4–11 mm long. Seeds ca. 0.75 mm long.

##### Distribution.

Sumatra, Java and Lesser Sunda Islands (Bali).

##### Ecology and habitat.

Primary or secondary montane forest, rarely near crater, at 1000–1700 m elevation.

##### Vernacular names.

Java: *harendong cai* (Sunda); *kramas madu* (Java).

##### Note.

*Dissochaetaleprosa* resembles *D.intermedia*, but differs in the larger hypanthium and distinctly triangular calyx lobe tips. Otherwise, *D.leprosa* has a more tomentose indumentum than *D.intermedia*. The similarity in shape of the alternipetalous stamens between both species made Maxwell (1980b) regard *D.leprosa* as a variety of *D.intermedia*, but because of the differences in size and shape of the hypanthium, the calyx lobe tips and the alternipetalous stamens, it is considered to be a distinct species (Kartonegoro and Veldkamp 2010).

##### Specimens examined.

**INDONESIA. West Sumatra**: Mt. Singgalang, O. Beccari PS 369 (BM, L). **Central Java**: Pekalongan, Mt. Praboto, 1350 m, 12 Sep 1914, C.A. Backer 15983 (BO); *Ibid.*, Petung Kriono, 1400 m, 9 Sep 1914, C.A. Backer 15787 (BO); Semarang, Mt.Telomoyo, 1500 m, 14 Jun 1892, S.H. Koorders 27844β (BO); *Ibid.*, 12 May 1899, S.H. Koorders 35840β (BO); *Ibid.*, S.H. Koorders 35841β (BO); Magelang, Mt. Andong, 19 Jun 1897, S.H. Koorders 27846β (BO). **East Java**: Ponorogo, Mt.Wilis, Sikandang, 15 Aug 1897, S.H. Koorders 29308β (BO); Malang, Punten, 1200 m, 25 Dec 1928, C.G.G.J. van Steenis 2486 (BO); Mt.Kawi, 1160 m, 13 May 1982, Anon. FS 48 (L). **West Java**: Bogor, Puncak Pass, 1 Sep 1896, Sapiin 1115 (BO); *Ibid.*, Mt. Pancar, 20 Dec 1893, V.F. Schiffner 2291 (BO, L); *Ibid.*, Gunung Melati, F. Went s.n. (L); Mt.Halimun, H. Uchida 20 (BO); *Ibid.*, Mt. Botol, 4 Mar 2000, W.S. Hoover et al. 32663 (BO); *Ibid.*, Nirmala Estate, 1300 m, 10 Jun 1980, M.M.J. van Balgooy & H. Wiriadinata 2922 (BO, L); Cianjur, Mt. Gede, H. Kuhl & J.C. van Hasselt s.n. (L); *Ibid.*, Cibodas, 1400 m, 10 Dec 1925, B.H. Danser 5955 (L); *Ibid.*, 24 Oct 1898, S.H. Koorders 31506β (BO); *Ibid.*, 16 Oct 1898, S.H. Koorders 31523β (BO); *Ibid.*, 18 Oct 1896, S.H. Koorders 25949β (BO); *Ibid.*, Geger Bentang, 1600 m, 2 Jun 1948, Kakah 92 (BO, L); *Ibid.*, 20 Jul 1914, C.A. Backer 14714 (BO); *Ibid.*, 27 Mar 1924, M.L.A. Bruggeman 48 (BO); *Ibid.*, 4 Aug 1924, M.L.A. Bruggeman 211 (BO); *Ibid.*, 11 Sep 1927, M.L.A. Bruggeman 839 (BO); *Ibid.*, R.H.C.C. Scheffer s.n. (BO); *Ibid.*, 1 May 1950, S.J. van Ooststroom 13840 (L, PNH); *Ibid.*, 3 Jul 1896, H. Raap 667 (L); *Ibid.*, J.G. Boerlage s.n. (L); *Ibid.*, 10 Feb 1895, J.G. Hallier 626a (BO); Cibeber, Mt. Beser, 1000 m, 27 Jun 1917, J.J. Smith 719 (BO, L, U); Bandung, Mt. Tangkuban Perahu, 1600 m, 26 Dec 1919, W.A. Horst 2 (BO); *Ibid.*, 26 Jul 1927, W.M. Docters van Leeuwen 11487 (BO); *Ibid.*, 18 Jul 1916, W.M. Docters van Leeuwen 2301 (BO); *Ibid.*, J.G. Boerlage s.n. (L); *Ibid.*, W.H. de Vriese 149 (L); *Ibid.*, Oct 1903, C.A. Backer s.n. (BO); *Ibid.*, 4 Mar 1912, C.A. Backer 2386 (BO); *Ibid.*, 28 May 1908, H.H. Zeijlstra 19 (L); Cibeureum, 1550 m, 3 Apr 1911, J.J. Smith & A. Rant 125 (BO, U); Mt. Malabar, R.H.C.C. Scheffer s.n.(BO); Pengalengan, F.W. Junghuhn 13 (L, U); Mt.Rendang, F.W. Junghuhn s.n. (L); Garut, Mt.Guntur, 1500 m, 1937, B.J. Karsten 66 (L); Pagencongan, Jan 1909, C.A. Backer s.n. (BO); Cianjur, Cireungas, Gunung Malang, 14 Mar 1909, C.A. Backer s.n. (BO). **Bali**: Mt. Patas, 1015 m, 20 Nov 1918, Sarip 465 (BO).

#### 33. Dissochaeta macrosepala

Taxon classificationPlantaeMyrtalesMelastomataceae

Stapf, J. Linn. Soc., Bot. 42: 80. 1914.

Fig. 21 , Map 18

Dissochaeta rostrata Korth. var. macrosepala (Stapf) J.F.Maxwell, Gard. Bull. Singapore 33: 320. 1980.

##### Type.

Malaysia. Sabah: Ranau, Mt. Kinabalu, Ridge above Bundu Tuhan, 3000 ft. elev., Feb 1910, L.S. Gibbs 3951 (holotype: K [K000859636]!).

**Figure 21.**
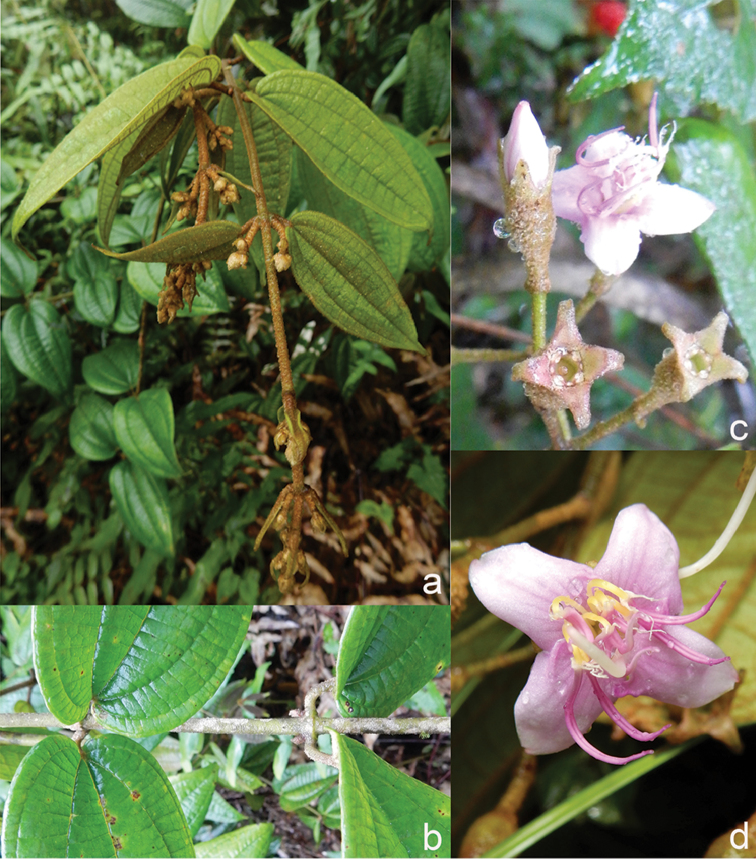
*Dissochaetamacrosepala***a** habit **b** branchlet **c** hypanthium **d** flower Photographs by D. Penneys; voucher: Penneys 2512 (WNC)].

##### Description.

Climbing up to 3 m in height. Branchlets terete, 3–4 mm in diameter, densely covered with brown stellate-tomentose hairs, glabrescent; nodes swollen, with interpetiolar ridge; internodes 4.4–5.5 cm long. Leaves: petioles terete, 5–8 mm long, densely brown stellate-tomentose; blades ovate, 6–6.2 × 4–4.3 cm, subcoriaceous, base cordate, margin entire, apex acuminate, tip ca. 0.5 cm long; nervation with 1 or 2 pairs of lateral nerves and 1 pair of intramarginal nerves; adaxially glabrous, with prominent reticulate sunken venation, abaxially densely covered with brown stellate-tomentose hairs. Inflorescences terminal, up to 15 cm long, many-flowered; main axis densely covered with brown stellate-tomentose hairs; primary axes up to 12 cm long with 3 or 4 nodes, secondary axes 2–2.5 cm long with 1–3 nodes, tertiary axes up to 1 cm long with 1 node; bracts linear or leaf-like, 4–15 mm long, stellate-tomentose, caducous; bracteoles subulate, 2–6 mm long, stellate-tomentose, caducous; pedicels brown stellate-tomentose, 3–4 mm long in central flowers, 1–2 mm long in lateral flowers. Hypanthium tubular to suburceolate, 5–6 × 2–3 mm, densely brown stellate-tomentose; calyx lobes lanceolate, 4–4.5 mm long, apex acute, tomentose; petal buds conical, 4–5 mm long, glabrous; mature petals ovate, 6–8 × 4–5 mm, base clawed, apex acute, bright pink. Stamens 8, subequal, filaments curved sideways, yellowish; alternipetalous stamens with 6–7 mm long filaments, anthers slender, lanceolate, sickle-shaped, thecae 8–9 mm long, purplish, pedoconnective 2–3 mm long, basal crest triangular 1–2 mm long, whitish, lateral appendages prolonged from base of crest, paired, filiform, 4–5 mm long, whitish; oppositipetalous stamens with 5–6 mm long filaments, anthers thicker, S-shaped, thecae 6–7 mm long, purplish, basal crest minute or spur-like, whitish, lateral appendages from base with a paired, ligulate appendages, ca. 1 mm long, whitish. Ovary ⅔ of hypanthium in length, apex pubescent; style 10–12 mm long, curved at the apex, glabrous, white with purplish apex; stigma minute, capitate, yellowish; extra-ovarial chambers 8, extending nearly to the middle of the ovary. Fruits ovoid or urceolate, 8–9 × 5–6 mm long, densely covered with stellate-tomentose hairs; calyx lobes persistent, reflexed. Seeds ca. 0.5 mm long.

##### Distribution.

Borneo (Mount Kinabalu).

##### Ecology and habitat.

Lower montane forest, in open places, at ca. 914 m elevation.

##### Note.

The indumentum of *D.macrosepala* resembles *D.densiflora*, but the former species is different in the long, lanceolate calyx lobes. Maxwell (1980b) considered both species as varieties of *D.rostrata*. Stapf and Green (1914) incorrectly noted that the species has four stamens, but it has 8 stamens like the other similar species.

##### Specimens examined.

**MALAYSIA. Sabah**: Ranau, Mt. Kinabalu, Bundu Tuhan, 914 m, Feb 1910, L.S. Gibbs 3951 (K); *Ibid.*, Dallas, 914 m, 1 Dec 1931, J. Clemens & M.S. Clemens 30340 (L).

#### 34. Dissochaeta malayana

Taxon classificationPlantaeMyrtalesMelastomataceae

Furtado, Gard. Bull. Singapore 20: 110. 1963.

Map 20

Dissochaeta rostrata Korth. var. malayana (Furtado) J.F.Maxwell, Gard. Bull. Singapore 33: 320. 1980.

##### Type.

Malaysia. Terengganu: Kemaman, Bukit Kajang 350 m, E.J.H. Corner SFN 30381 (holotype: SING!).

**Map 20.**
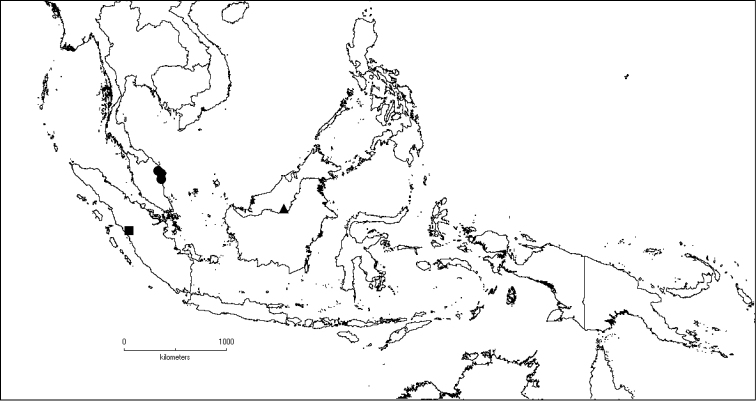
Distribution of *D.malayana* (●), *D.marumioides* (■) and *D.maxwellii* (▲).

##### Description.

Branchlets terete, 2–3 mm in diameter, covered with scattered, ca. 1 mm long, dark purple bristle hairs; nodes swollen, with interpetiolar ridge, densely covered with brown stellate-furfuraceous hairs and scattered bristles; internodes 4.5–7 cm long. Leaves: petioles terete, 5–8 mm long, densely covered with stellate-furfuraceous and bristle hairs; blades ovate, 7.5–10 × 3.5–4.8 cm, membranous, base emarginate, margin entire, apex acuminate, tip ca. 0.5 cm long; nervation with 1 pair of lateral nerves and 1 pair of intramarginal nerves; surfaces hirsute, with sparse stellate hairs and scattered dark bristle hairs, more densely so at midrib and margin. Inflorescences terminal, up to 15 cm long, many-flowered; main axis angular, covered with scattered, dark purple bristle hairs; primary axes up to 12 cm long with 3–5 nodes, secondary axes 2–5 cm long with 1 or 2 nodes, tertiary axes up to 1.5 cm long with 1 node; bracts linear, 3–4 mm long, with stellate-furfuraceous and dark purple bristle hairs; bracteoles linear or subulate, 1–2 mm long, with stellate-furfuraceous and dark bristle hairs; pedicels with densely stellate-furfuraceous and dark purple capitate bristle hairs, 5–6 mm long in central flowers, 2–3 mm long in lateral flowers. Hypanthium suburceolate, 8–10 × 3–4 mm, densely covered with stellate-furfuraceous and dark capitate purple bristle hairs; calyx lobes truncate with undulate tip, 1–1.5 mm long; petal buds conical or rounded, 4–5 mm long, apex bristly; mature petals obovate, ca. 7 × 4 mm, not-reflexed, base clawed, apex rouded, bristly, otherwise glabrous, pink-purple. Stamens 8, subequal, filaments curved sideways, yellow; alternipetalous stamens with 7–8 mm long filaments, anthers lanceolate, sickle-shaped, thecae ca. 10 mm long, apex rostrate, yellow, pedoconnective 1–1.5 mm long, basal crests minute, triangular, ca. 0.5 mm long, lateral appendages paired, filiform, 3–4 mm long; oppositipetalous stamens with 7–8 mm filaments, anthers thick, slightly S-shaped, thecae 9–10 mm long, pink, basal crests minute, ligular or spur-like, ca. 0.5 mm long, lateral appendages paired, filiform, 1–1.5 mm. Ovary ⅔ of hypanthium in length, apex villous and bristly; style 13–15 mm long, curved at end, glabrous, pink; stigma minute, capitate; extra-ovarial chambers 8, extending to the middle of the ovary. Fruits urceolate, 8–10 × 4.5–5 mm, sparsely stellate-furfuraceous and densely covered with dark capitate bristle hairs; calyx lobe remnants persistent, reflexed. Seeds ca. 0.5 mm long.

##### Distribution.

Peninsular Malaysia (Terengganu).

##### Ecology and habitat.

Lowland forest at 150–400 m elevation.

##### Note.

This species resembles *D.johorensis*; both species have most parts covered by bristle hairs. *Dissochaetamalayana* is different in having more distinct dark purple bristle hairs and truncate calyx lobes and geographically it is disjunct, only present in the northern part of the peninsula.

##### Specimens examined.

**MALAYSIA. Terengganu**: Ulu Brang, 365 m, Jul 1937, L. Moysey & Kiah SFN 33858 (BO, K); Kemaman, Bukit Kajang, E.J.H. Corner SFN 30381 (SING); Dungun, Jerangau Road, 16 Nov 1954, J. Sinclair & Kiah SFN 40492 (BM, BO, K, L).

#### 35. Dissochaeta marumioides

Taxon classificationPlantaeMyrtalesMelastomataceae

Cogn. in A.DC. & C.DC., Monogr. Phan. 7: 556. 1891. non. D. marumioides Furtado, 1963 (see under D. spectabilis).

Map 20

##### Type.

Indonesia. West Sumatra: Mt. Singgalang, 1600 m elev., O. Beccari s.n. (holotype: FI [FI007931, image seen]!).

##### Description.

Branchlets terete, 3–5 mm in diameter, brown stellate-furfuraceous; nodes swollen, with annular interpetiolar ridge; internodes 3–5 cm long. Leaves: petioles flattened, 10–15 mm long, densely stellate-furfuraceous; blades ovate-elliptic, 8.3–14 × 3.5–5 cm, membranous, base cordate, margin entire, apex acuminate, tip 1–2 cm long; nervation with 1 pair of lateral nerves and 1 pair of intramarginal nerves; adaxially glabrous, dark green, abaxially brown stellate-furfuraceous. Inflorescences terminal, up to 20 cm long, up to 20 flowers; main axis quadrangular, densely stellate-furfuraceous; primary axes up to 17 cm long with 5 nodes, secondary axes up to 3 cm long with 1 or 2 nodes, tertiary axes when developed 0.6–0.7 cm long with 1 node; bracts leaf-like, oblong-lanceolate, 15–22 × 4–6 mm, densely brown stellate-furfuraceous; bracteoles linear, 2–3 mm long, densely brown stellate-furfuraceous; pedicels densely brown stellate-furfuraceous, ca. 3 mm long in central flowers, 1–2 mm long in lateral flowers. Hypanthium campanulate, 7–9 × 3–5 mm, densely with stellate-furfuraceous and scattered, thickened, 1–2 mm long bristles; calyx lobes rounded, ca. 2 mm long, margin ciliate, apex obtuse, stellate-furfuraceous; petal buds conical, 3–5 mm long, acute; mature petals elliptic to suborbicular, ca. 12 × 9 mm, base clawed, apex acute, glabrous. Stamens 8, unequal, filaments curved sideways, glabrous; alternipetalous stamens with 6–7 mm long filaments, anthers slightly curved, lanceolate, thecae ca. 8 mm long, pedoconnective ca. 2 mm long, basal crest triangular, ca. 1.5 mm long, thin, entire or bifid, lateral appendages 4, filiform, 2–3 mm long; oppositipetalous stamens with ca. 6 mm long filaments, anthers sinuate, curved, thecae ca. 8 mm long, basal crest minute, ca. 0.3 mm long, lateral appendages paired, filiform appendages 2–3 mm long. Ovary ⅔ of hypanthium in length, apex villous; style ca. 15 mm long, curved at top, glabrous; stigma minute; extra-ovarial chambers 8, extending nearly to the base of the ovary. Fruits not seen.

##### Distribution.

Sumatra (West).

##### Ecology and habitat.

Montane forest at ca. 1600 m elevation.

##### Note.

*Dissochaetamarumioides* is only known from the type from Mount Singgalang, West Sumatra. This species has a non-bristly indumentum on branchlets and leaves; though sparse bristles are present on the hypanthium and calyx lobes. The 4 lateral appendages of the alternipetalous stamens are exceptional, all other species in the genus having 2. The calyx lobes with rounded apex are similar to those of *D.rostrata* from Borneo, but the species differs in the shape of the bracts.

#### 36. Dissochaeta maxwellii

Taxon classificationPlantaeMyrtalesMelastomataceae

(Karton.) Karton. comb. nov.

urn:lsid:ipni.org:names:60476840-2

Map 20

##### Basionym.

*Diplectriamaxwellii* Karton., Kew Bull. 73-23: 1, fig. 1. 2018.

##### Type.

Malaysia. Sarawak: Kapit Division, Batang Baleh, Nanga Serani, 500 m elev., 4 May 1991, Runi et al. S.63137 (holotype: K [K000566618]!; isotypes: KEP [KEP43526, image seen]!, L [L3908632]!, SAN! [image seen], SAR *n.v.*).

##### Description.

Climbing up to 6 m in height. Branchlets terete, angular in the upper branches, ca. 3 mm in diameter, glabrous to sparsely stellate-puberulous; nodes swollen, with distinct interpetiolar ridge, annulum, densely covered with stellate-pubescent hairs and 4–6 mm long, brown, bristle hairs, prominent, ca. 0.1 mm thick at base, narrowing towards the acute tip; annulus 1–2 mm high, brownish; internodes ca. 5–6 cm long. Leaves: petioles terete, 10–12 mm long, densely stellate-pubescent and covered with dense, 4–7 mm long, brown bristle hairs; blades elliptic-oblong, 10.3–12.9 × 3.8–5 cm, membranous, base rounded, margin entire, apex acuminate, tip 1.2–1.5 cm long; nervation with 1 pair of lateral nerves and 1 pair of intramarginal nerves; adaxially glabrous, dark green, abaxially glabrous or stellate-puberulous on midrib and sparsely hairy on lateral veins, light brown. Inflorescences terminal, up to 14.8 cm long, many-flowered; main axis quadrangular, densely stellate-pubescent and sparsely covered with bristle hairs, purplish-green, nodes densely stellate-pubescent and densely covered with 3–5 mm long brown bristle hairs; primary axes ca. 12 cm long with 3 or 4 nodes, secondary axes 1.5–6 cm long with 1 or 2 nodes, tertiary axes 6–8 mm long with 1 node; bracts oblong-lanceolate, 5–7 mm long, densely stellate-pubescent, covered with 3–4 mm brown bristle hairs at the margin; bracteoles oblong, 2–3 mm long, densely stellate-pubescent; pedicels densely stellate-pubescent, purplish, 4–7 mm long in central flowers, 3–3.5 mm long in lateral flowers. Hypanthium tubular, campanulate, 3.5–4 × ca. 2.5 mm, densely pubescent and sparsely covered with glandular bristle hairs in middle and base; calyx lobes truncate, ca. 0.5 mm long, minutely apiculate; petal buds conical, 1–2 mm long, acute; mature petals obovate, 3–4.5 × 2.5–3 mm, apex acute, base clawed, glabrous. Stamens 8, unequal, filaments straight, glabrous; alternipetalous stamens staminodial with 3–4 mm long filaments, anthers reduced, thecae rudimentary, lanceolate, 2–4 mm long, terete, thin, basal crest triangular with erose tip, ca. 1 mm long, lateral appendages absent; oppositipetalous stamens with ca. 3 mm long filaments, anthers clavate, sickle-shaped, curved, thecae 4–5 mm long, glabrous, smooth, basal crest minute, ca. 0.3 mm long, lateral appendages paired, small, ligular, ca. 0.5 mm long. Ovary ⅔ of hypanthium in length, densely pubescent at top; style curved at top, 6–10 mm long, glabrous; stigma minute, inconspicuous; extra-ovarial chambers 4, extending to the middle of the ovary. Fruits globose, 5–8 × 3–6 mm, glabrous, red when ripe; calyx lobe remnants persistent, erect. Seeds ca. 0.5 mm long.

##### Distribution.

Borneo (Sarawak).

##### Ecology and habitat.

Sub-montane forest at ca. 500 m elevation.

##### Vernacular name.

*akar kemunting* (Kapit).

##### Note.

A distinct species that resembles *D.viminalis* in the number and shape of the stamens, but differs in having dense prominent bristle hairs on the nodes, petioles, bracts and bracteoles. Known only from the type from Kapit Division, Sarawak.

#### 37. Dissochaeta micrantha

Taxon classificationPlantaeMyrtalesMelastomataceae

(Veldkamp) Karton. comb. nov.

urn:lsid:ipni.org:names:60476841-2

Map 21

Diplectria glabra (Merr.) M.P.Nayar var. micrantha (Veldkamp) J.F.Maxwell, Gard. Bull. Singapore 33: 313. 1980.

##### Basionym.

*Diplectriamicrantha* Veldkamp, Blumea 24: 422, fig. 5B. 1978.

##### Type.

Malaysia. Sabah: Ranau District, Mount Kinabalu, Sosopodon, 4720 ft. elev., 30 Jan 1962, I.H. Sario SAN 28959 (holotype: L [L0008871]!; isotypes: K [K000859550]!, SAN *n.v.*).

**Map 21.**
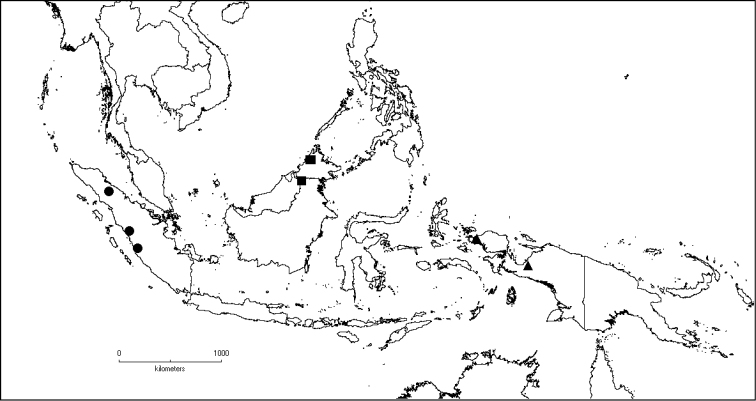
Distribution of *D.micrantha* (■), *D.nodosa* (●) and *D.papuana* (▲).

##### Description.

Branchlets terete, 3–4 mm in diameter, glabrous; nodes swollen, with prominent annular, 2–3 mm wide, crest-like interpetiolar ridge, margin with 3–5 mm long simple bristles; internodes 6–12 cm long. Leaves: petioles terete, 5–10 mm long, glabrous to nearly furfuraceous and dorsally with bristle hairs; blades ovate, 6.5–9 × 3.5–5 cm, subcoriaceous, base cordate, margin entire, apex acuminate, tip 0.5–1 cm long; nervation with 1 pair of lateral nerves and 1 pair of intramarginal nerves; adaxially glabrous, abaxially glabrous except scattered stellate hairs on the nerves. Inflorescences terminal, up to 25 cm long, many-flowered; main axis angular, glabrous; primary axes up to 23 cm long with 7–9 nodes, secondary axes 8–10 cm long with 5–7 nodes, tertiary axes up to 1.5 cm long with 1 or 2 nodes, quarternary axes when developed up to 0.5 cm long with 1 node; bracts linear or ligular, 3–4 mm long, glabrous, caducous; bracteoles subulate, 1–2 mm long, glabrous; pedicels stellate-furfuraceous, 2–3 mm long in central flowers, ca. 1 mm long in lateral flowers. Hypanthium urceolate, ca. 3 × 1–1.5 mm, glabrous; calyx lobes truncate, ca. 0.3 mm long, apex with 4 minute points; petal buds conical, 1–1.5 mm long; mature petals suborbicular, 2–2.5 × ca. 2 mm, reflexed, base clawed, apex acute, pink. Stamens 8, unequal, filaments straight; alternipetalous stamens staminodial, with ca. 1 mm long filaments, thecae rudimentary, ca. 0.5 mm long, slender, terete, basal crest triangular, thin, ca. 1 mm long, apex erose, base with a small auricle, lateral appendages absent; oppositipetalous stamens with 1–2 mm long filaments, anthers thick, oblong, thecae 2–2.5 mm long, white, basal crest keels bifid, ca. 0.2 mm long, lateral appendages bifid, ca. 0.3 mm long. Ovary ⅔ of hypanthium in length, apex glabrous; style 3–4 mm long, glabrous; stigma minute, capitate; extra-ovarial chambers 4, oppositipetalous, extending to ⅓ of the ovary. Fruits subglobose, 4–5 × ca. 4 mm, glabrous; calyx lobe remnants persistent. Seeds ca. 0.5 mm long.

##### Distribution.

Borneo (Sabah and North Kalimantan).

##### Ecology and habitat.

Montane forest on river banks or in open places at 1100–1400 m elevation.

##### Note.

The desciption of the flowers is based on a bud only since mature flowers are still unknown. This species is restricted to the montane forests of Mount Kinabalu, Sabah and Kayan Mentarang, North Kalimantan.

##### Specimens examined.

**MALAYSIA. Sabah**: Ranau, Mt. Kinabalu, Sosopodon, 1440 m, 30 Jan 1962, I.H. Sario SAN 28959 (K, L); *Ibid.*, Tenompok, 1500 m, 29 Feb 1932, J. Clemens & M.S. Clemens 28588 (BM, K); *Ibid.*, May 1932, J. Clemens & M.S. Clemens 30341 (K); *Ibid.*, between Tenompok and Kundasan, 1400 m, 15 Jul 1957, J. Sinclair et al. 9235 (L); *Ibid.*, Sungai Mamut, 1200–1400 m, 15 Feb 1969, S. Kokawa & M. Hotta 5798 (L). **INDONESIA. North Kalimantan**: Krayan, Kayan Mentarang, Long Bawan, 1100 m, 29 Jul 1981, M. Kato et al. B-10105 (BO, L).

#### 38. Dissochaeta nodosa

Taxon classificationPlantaeMyrtalesMelastomataceae

Korth. in Temminck, Verh. Nat. Gesch. Ned. Bezitt., Bot.: 239. 1844.

Map 21

Aplectrum nodosum (Korth.) Blume, Mus. Bot. 1(3): 37. 1849.
Anplectrum nodosum (Korth.) Triana, Trans. Linn. Soc. London 28: 84. 1872.
Dissochaeta montana Cogn. in A.DC. & C.DC., Monogr. Phan. 7: 558. 1891. Type: Indonesia. West Sumatra: Mt. Singgalang, 1700 m elev., O. Beccari PS 4124 (holotype: FI [FI007930, image seen]!).
Anplectrum yatesii Merr., Pap. Michigan Acad. Sci. 19: 175. 1934. Type: Indonesia. North Sumatra: Karoland, Berastagi, 12 Mar 1926, H.S. Yates 2012 (lectotype, designated here: BO [BO1429407]!; isolectotypes: L [L0537225]!, MICH [MICH-1111808, image seen]!, NY [NY00221311, image seen]!).
Dissochaeta yatesii (Merr.) Veldkamp, Blumea 24: 435, 443. 1979.

##### Type.

Indonesia. West Sumatra: Indrapoera, P.W. Korthals s.n. (lectotype, designated by Kartonegoro and Veldkamp 2010, pg. 143: L [L0537233]!; isolectotype: L [L0537232]!).

##### Description.

Branchlets terete, 4–5 mm in diameter, stellate-furfuraceous; nodes swollen, interpetiolar ridge present; internodes 5.5–7.3 cm long. Leaves: petioles flattened, 8–10 mm long, stellate-furfuraceous; blades ovate to elliptic, 8.3–12.5 × 3.5–6 cm, membranous, base subcordate, margin entire, apex acuminate, tip ca. 8–15 mm long; nervation with 1 pair of lateral nerves and 1 pair of intramarginal nerves; adaxially glabrous, abaxially glabrous to sparsely stellate-furfuraceous. Inflorescences terminal, up to 17 cm long, many-flowered; main axis angular, glabrescent to sparsely stellate-puberulous; primary axes up to 15 cm long with 5 or 6 nodes, secondary axes up to 4 cm long with 1 or 2 nodes, tertiary axes up to 1.5 cm long with 1 node; bracts and bracteoles inconspicuous, caducous; pedicels sparsely stellate-furfuraceous, 3–4 mm long in central flowers, 1–2 mm long in lateral flowers. Hypanthium broadly campanulate, ca. 4 × 3 mm, stellate-furfuraceous; calyx lobes truncate with 4 triangular tips, ca. 1 mm long, glabrous; petal buds conical, 2–3 mm long; mature petals obovate, 3–4 × ca. 3 mm, base clawed, apex rounded, glabrous. Stamens 8, equal or subequal, straight, filaments straight; alternipetalous stamens with filaments 4–6 mm long, anthers oblong or lanceolate, thecae ca. 5 mm long, pedoconnective ca. 0.5 mm long, basal crest triangular, ca. 1.5 mm long, acute, lateral appendages absent; oppositipetalous stamens with filaments ca. 3 mm long, anthers lanceolate, thecae 3–4 mm long, thick, basal crest ligular, 1–1.5 mm long, lateral appendages absent. Ovary ¾ of hypanthium in length, apex puberulous; style 8–10 mm long, curved at top, glabrous; stigma minute; extra-ovarial chambers 8, extending to the middle of the ovary. Fruits subglobose, 6–7 × 3–4 mm, glabrescent, apex mammiform; calyx lobes persistent, erect. Seeds ca. 0.5 mm long.

##### Distribution.

Sumatra.

##### Ecology and habitat.

Montane forest, in open places at 1300–1700 m elevation.

##### Specimens examined.

**INDONESIA. North Sumatra**: Karo, Brastagi, H.S. Yates 2012 (BO, L, MICH, NY). **West Sumatra**: Mount Singgalang, O. Beccari PS 4124 (FI); Indrapura, P.W. Korthals s.n. (L).

#### 39. Dissochaeta pallida

Taxon classificationPlantaeMyrtalesMelastomataceae

(Jack) Blume, Flora 14: 500. 1831.

Fig. 22 , Map 22

Melastoma pallidum Jack, Trans. Linn. Soc. London 14: 12. 1823 (“pallida”).
Dissochaeta ovalifolia Naudin, Ann. Sci. Nat., Bot. sér. 3, 15: 76. 1851. Type: Malaysia. Peninsular Malaysia, Pulo Pinang, Mar 1837, C. Gaudichaud-Beaupré 94 (lectotype, designated here: P [P02274807, image seen]!; isolectotype: P [P02274808, image seen]!).
Dissochaeta superba Naudin, Ann. Sci. Nat., Bot. sér. 3, 15: 77. 1851. Type: Malaysia. Peninsular Malaysia, Pulo Pinang, Mar 1837, C. Gaudichaud-Beaupré 93 (lectotype, designated here: P [P02274809, image seen]!; isolectotypes: P [P02274810, P02274811, images seen]!).
Dissochaeta astrosticta Miq., Fl. Ned. Ind., Eerste Bijv. 2: 318. 1861. Type: Indonesia. Sumatra, Bangka bij Djeboes, J.E. Teijsmann HB 3424 (lectotype, designated here: BO [BO1865965]!; isolectotypes: BM [BM000944481]!, BO [BO1865966]!, K [K000859484]!, U [U0004010, U0124122]!).
Dissochaeta sumatrana Boerl. & Koord. in Koord.-Schum., Syst. Verz. 2: 46. 1911. Type: Indonesia. Riau: Sangkamiang, 40 m elev., 29 Mar 1891, S.H. Koorders 22330β (holotype: BO [BO1294108]!).
Dissochaeta borneensis Bakh.*f.*, Contr. Melastom.: 231. 1943. Type: Indonesia. West Kalimantan: Pontianak, Kp. Andjongan, 5 Apr 1931, Mondi 252 (holotype: L [L0126153]!; isotypes: BO [BO1779331, BO1779332, BO1779333]!, K [K000859487, K000859488]!).

##### Type.

Malaysia. Peninsular Malaysia, Penang, W. Jack 55 (lectotype, designated here: BM [BM000944482]!).

**Figure 22.**
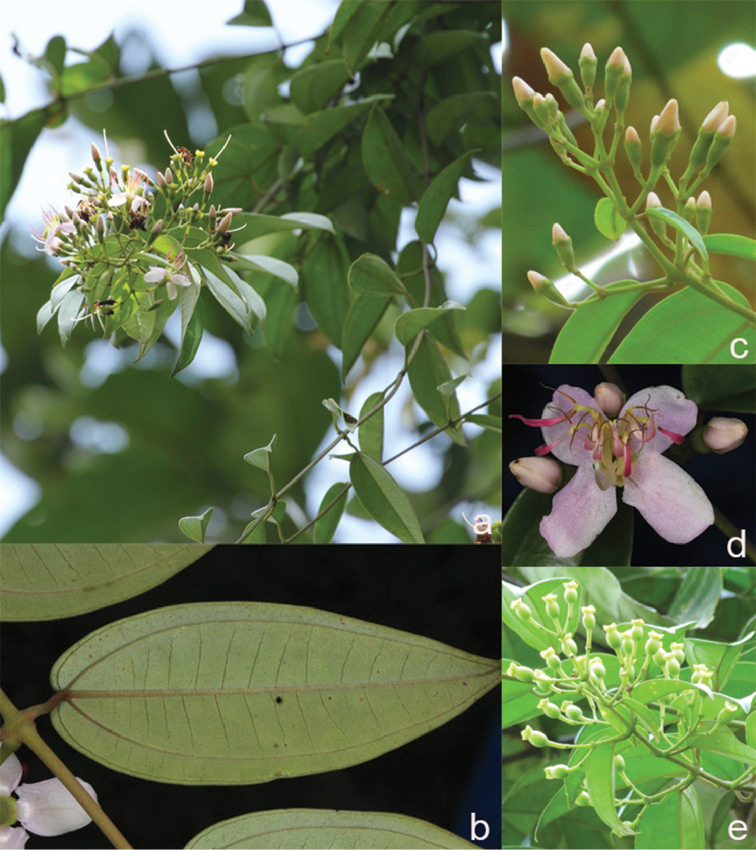
*Dissochaetapallida***a** habit **b** branchlet **c** hypanthium **d** flower **e** fruits. Photographs by W.F. Ang.

**Map 22.**
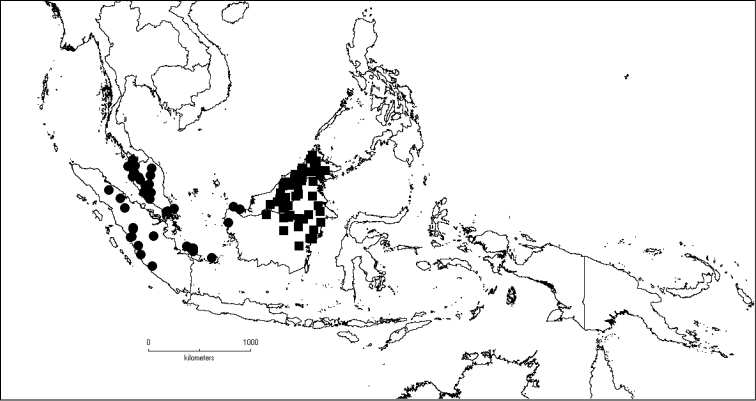
Distribution of *D.pallida* (●) and *D.pulchra* (■).

##### Description.

Climbing up to 25 m in height. Branchlets terete, 2–4 mm in diameter, smooth, sparsely covered with minute stellate hairs, glabrescent; nodes swollen, with interpetiolar ridge; internodes 7–11.5 cm long. Leaves: petioles flattened, 10–20 mm long, sparsely furfuraceous; blades ovate to elliptic, 6–14 × 4–8 cm, subcoriaceous, base broadly rounded to cordate, margin entire, apex acuminate, tip ca. 0.5 cm long; nervation with 1 pair of lateral nerves and 1 pair of intramarginal nerves; adaxially glabrous, abaxially glabrous or punctate. Inflorescences terminal and axillary; up to 25 cm long when terminal, up to 11 cm long when axillary; many-flowered; main axis sparsely furfuraceous or glabrous; primary axes 8–15 cm long with 5 or 6 nodes, secondary axes 4.5–5 cm long with 1 or 2 nodes, tertiary axes 0.8–1.2 cm long with 1 node; bracts linear or lanceolate, 5–7 mm long, glabrous or furfuraceous; bracteoles linear or lanceolate, 2–4 mm long; pedicels glabrous, 4–5 mm long in central flowers, ca. 2 mm long in lateral flowers. Hypanthium campanulate-tubular, 6–8 × 4–5 mm, obscurely 8-lined or 8-ridged, glabrous or punctate; calyx lobes truncate, 1–2 mm long, with 4 undulate or acute tips; petal buds slightly conical, 9–15 mm long; mature petals obovate to oblong, 7–8 × ca. 5 mm, reflexed, base clawed, apex acute to subrounded, glabrous, bright white or white pinkish. Stamens 8, unequal, filaments curved sideways, yellow; alternipetalous stamens with 10–12 mm long filaments, anthers narrow, curved, sickle-shaped, thecae 12–15 mm long, pedoconnective 3–4 mm, basal crest triangular, ca. 1 mm long, lateral appendages paired, filiform, 5–6 mm long; oppositipetalous stamens with 9–10 mm long filaments, anthers S-shaped, thecae 4–7 mm long, thick, basal crest ligular, ca. 1 mm long, lateral appendages paired, filiform, 5–6 mm long. Ovary half as long as hypanthium, apex pubescent; style slender, 20–24 mm long, glabrous; stigma minute; extra-ovarial chambers 8, extending nearly to the base of the ovary. Fuits ovoid-urceolate, 8–10 × 6–7 mm, glabrous, punctate; calyx lobe, remnants persistent, widened. Seeds ca. 0.5 mm long.

##### Distribution.

Malay Peninsula, Sumatra and Borneo (Western part).

##### Ecology and habitat.

Primary lowland dipterocarp forest, in open places from 60–700 m elevation.

##### Vernacular names.

Peninsular Malaysia: *akar sunudo, akar duman bukit, akar sial munahon* (Malacca). Sumatra: *kedudu akar* (Riau). Borneo: *lingkodo kliko* (Pontianak).

##### Note.

*Dissochaetapallida* is distinguished by its campanulate-tubular 8-lined or 8-ridged hypanthium and truncate calyx lobes with undulate acute tip. The minute punctation on the abaxially leaf surface is a reliable field character to recognise this species. The indumentum on the leaves is similar to that of *D.punctulata* Hook.*f.* ex Triana, from which it differs in the indumentum of the hypanthium and the number of appendages of the stamens.

##### Specimens examined.

**MALAYSIA. Kedah**: Gunong Bintang, 300 m, 8 Apr 1968, Sidek bin Kiah 276 (K, L); Bukit Perak, 26 Nov 1969, Chan FRI 13138 (K). **Kelantan**: Bukit Baka, 2 Jun 1982, B.C. Stone & Chin 15244 (L). **Malacca**: W. Griffith KD 2292 (K). **Negri Sembilan**: Jelebu, Pasoh Forest, 80-120 m, 19 Jun 1996, E.C. Gardette 2046 (K, L); Gunong Angsi, 457 m, 20 Nov 1923, M. Nur SFN 11529 (BO). **Pahang**: Gunong Tahan, 21 Jun 1922, M. Haniff & M. Nur SFN 8077 (K); Ulu Krau, Gunung Benom, 580 m, 20 Apr 1967, Yusoff KEP 99109 (K, L). **Penang**: W. Jack 55 (BM); N. Wallich 4049 (BM, K, L, NY, P); G.W. Walker 28 (BM, K); R.W. Hullet 179 (BM, P); Mar 1837, C. Gaudichaud-Beaupré 93 (P); C. Gaudichaud-Beaupré 94 (P); C. Gaudichaud-Beaupré 96 (P); Government Hill, 152 m, Oct 1884, C. Curtis 80 (K); *Ibid.*, Apr 1890, C. Curtis 2297 (K, P); *Ibid.*, A.C. Maingay KD 792 (BM, K, L); *Ibid.*, A.C. Maingay KD 793 (2227) (K, L); Western Hill, 700 m, 1 Mar 1965, Hardial & Samsuri 181 (K, L); *Ibid.*, 720 m, 7 Feb 1991, L.G. Saw FRI 37342 (L); Penang Hill, 22 Aug 1879, King’s collector s.n. (P). **Perak**: B. Scortechini 22 (P); B. Scortechini 371 (L); B. Scortechini 1650 (L, P); B. Scortechini s.n. (P); Larut, Nov 1881, King’s collector 2570 (K); *Ibid.*, Mar 1883, King’s collector 3965 (BO); Maxwell’s Hill, 4 Mar 1965, Hardial & Samsuri 295 (K, L); Dending, 13 Mar 1896, Anon s.n. (BM); Tapah, L. Wray 1370 (U); *Ibid.*, Nov 1888, H.N. Ridley s.n. (BM); Thaiping Hill, 300–450 m, Feb 1886, King’s collector 8499 (P). **Selangor**: Klang Watercatchment, 12 Mar 1922, I.H. Burkill SFN 6841 (BO, K); Kuala Lumpur, H.N. Ridley 2015 (BM); Semangkok, 700 m, 6 May 1970, Chan FRI 13278 (K). **Sarawak**: Kuching, 22 Mar 1893, G.D. Haviland 3144 (K); Lundu, Gunung Pueh, 60 m, 19 Mar 1996, Julaihi & Runi S.73359 (L). **SINGAPORE.** A.C. Maingay KD 793 (2685) (BM, K); Bukit Timah, Mar 1890, H.N. Ridley 2017a (BM); Choa Chu Kang, 9 Dec 1890, H.N. Ridley 2017 (BM); Jurong River, 13 Mar 1919, I.H. Burkill SFN 4081 (BO); Seletar, 29 Mar 1889, H.N. Ridley s.n. (BM). **INDONESIA. Bangka-Belitung**: Bangka, J.D. Kobus s.n. (BO); *Ibid.*, T. Horsfield 15 (K); *Ibid.*, Jebus, J.E. Teijsmann HB 3424 (BM, BO, K); *Ibid.*, J.E. Teijsmann s.n. (BO); *Ibid.*, Plangas, J.E. Teijsmann HB 3197 (BO); *Ibid.*, Sungai Liat, Bukit Tampang, 70 m, 23 Oct 1917, H.A.B. Bünnemeijer 1675 (BO); Belitung, Tanjung Pandan, J.E. Teijsmann s.n. (BO). **Bengkulu**: C.J. Brooks s.n. (K). **North Sumatra**: Labuhan Batu, Aek Kanopan, Lundut Concession, Kualu, 14 Mar 1927, E. Bartlett 6900 (K, L); *Ibid.*, 1 Apr 1927, E. Bartlett 7315 (L); *Ibid.*, Kota Pinang, Langga Payung, 7-30 Mar 1933, Rahmat Si Toroes 3294 (L); *Ibid.*, 7–14 Apr 1933, Rahmat Si Toroes 3837 (L). **Riau**: Indragiri Hulu, Kuala Belilas, 22 Apr 1939, P. Buwalda 6666 (BO, K, L, PNH); Sangkamiang, 40 m, 29 Mar 1891, S.H.Koorders 22330β (BO). **West Sumatra**: Padang, Limau Manis, 400 m, 5 Sep 2017, A. Kartonegoro 1058 (BO, L); Lima Puluh Kota, Taram, River Campo, 500-1000 m, 26 Aug 1957, W. Meijer 7024 (L); *Ibid.*, W. Meijer 7026a (L); *Ibid.*, Kelok Sembilan, 800 m, 20 May 2001, Putri et al. 63 (ANDA). **West Kalimantan**: Pontianak, Kp. Anjongan, 5 Apr 1931, Mondi 252 (BO, K, L).

#### 40. Dissochaeta papuana

Taxon classificationPlantaeMyrtalesMelastomataceae

(Mansf.) Karton. comb. nov.

urn:lsid:ipni.org:names:60476842-2

Map 21

Diplectria papuana (Mansf.) Bakh.*f.*, Contr. Melastom.: 202. 1943.
Diplectria glabra (Merr.) M.P.Nayar var. papuana (Mansf.) J.F.Maxwell, Gard. Bull. Singapore 33: 313. 1980.

##### Basionym.

*Anplectrumpapuanum* Mansf., Nova Guinea 14: 202. 1924.

##### Type.

Indonesia. Papua: Siriworivier, Jul 1912, R.F. Janowsky 132 (lectotype, designated here: L [L0008872]!; isolectotype: BO [BO1865947]!).

##### Description.

Branchlets terete, 3–4 mm in diameter, glabrous; nodes swollen, with raised annular crest-like interpetiolar ridge, often with bristle hairs; internodes 6–8.7 cm long. Leaves: petioles terete, ca. 10 mm long, furfuraceous and dorsally with bristle hairs; blades ovate, 8–16 × 4.5–9 cm, membranous to subcoriaceous, base cordate, margin entire, apex acuminate, tip 1–1.5 cm long; nervation with 1 pair of lateral nerves and 1 pair of intramarginal nerves; surfaces glabrous, abaxially with a pair of glandular patches at the base. Inflorescences terminal, up to 30 cm long, many-flowered; main axis angular, covered with stellate hairs; primary axes up to 28 cm long with 6 or 7 nodes, secondary axes up to 13 cm long with 4 or 5 nodes, tertiary axes up to 5 cm long with 3 or 4 nodes, quarternary axes when developed 1–1.5 cm long with 1 or 2 nodes; bracts linear, ca. 3 mm long, stellate-furfuraceous, caducous; bracteoles subulate, ca. 1 mm long, densely stellate-furfuraceous; pedicels stellate-furfuraceous, ca. 3 mm long in central flowers, 1–2 mm long in lateral flowers. Hypanthium suburceolate, 3–4 × ca. 2 mm, glabrous; calyx lobes truncate, ca. 0.5 mm long, apex triangular; petal buds conical, 1.5–2 mm long, apex acuminate; mature petals ovate, 3–3.5 × ca. 2 mm, reflexed, base clawed, apex acute, glabrous. Stamens 8, unequal, filaments straight; alternipetalous stamens staminodial, with ca. 2 mm long filaments, anthers rudimentary, thecae ca. 2 mm long, slender, terete, basal crest triangular, ca. 1 mm long, thin, base emarginate or hastate, apex acute, lateral appendages absent; oppositipetalous stamens with ca. 3 mm long filaments, anthers thick, curved, S-shaped, thecae 3–3.5 mm long, yellow, basal crest bilobed, ca. 0.3 mm long, lateral appendages absent. Ovary half as long as hypanthium, apex glabrous; style 7–8 mm long, curved at the end, slender, glabrous; stigma minute, capitate; extra-ovarial chambers 4, oppositipetalous, extending to the middle of the ovary. Fruits subglobose, 3–4 × ca. 3 mm, glabrous; calyx lobe remnants persistent. Seeds ca. 0.5 mm long.

##### Distribution.

New Guinea (Indonesian Papua).

##### Ecology and habitat.

Lowland forest at river banks at ca. 50 m elevation.

##### Note.

*Dissochaetapapuana* resembles *D.glabra* in its glabrous appearance and pair of glandular patches at the abaxially base of the leaves, but differs in the ovate shape of the leaf blade and the more urceolate hypanthium.

##### Specimens examined.

**INDONESIA. Papua**: Geelvink Bay, Siriwo River, Jul 1912, R.F. Janowsky 132 (BO, L). **West Papua**: Sorong, 50 m, 28 Aug 1948, D.R. Pleyte 705 (BO, K, L).

#### 41. Dissochaeta porphyrocarpa

Taxon classificationPlantaeMyrtalesMelastomataceae

Ridl., Kew Bull. 1: 32. 1946.

Fig. 23 , Map 23

Dissochaeta tawaensis Furtado, Gard. Bull. Singapore 20: 114. 1963. Type: Malaysia. Sabah: Elphinstone Province, Tawao, A.D.E. Elmer 21426 (holotype: SING!; isotypes: BM [BM000944477, BM001190928]!, BO [BO1429430]!, C [C10014565, image seen]!, K [K000859635]!, L [L0537282]!, P [P05283550, image seen]!, U [U0004011]!).
Dissochaeta rostrata Korth. var. porphyrocarpa (Ridl.) J.F.Maxwell, Gard. Bull. Singapore 33: 321. 1980.
Dissochaeta hirsuta auct. non Hook.*f.* ex Triana: Merr., Univ. Calif. Publ. Bot. 15: 224. 1929. *p.p.*, excl. type.

##### Type.

Malaysia. Sarawak: Ulu Tawaran, 2000 ft., G.D. Haviland 1287 (lectotype, designated here: K [K000859633]!).

**Figure 23.**
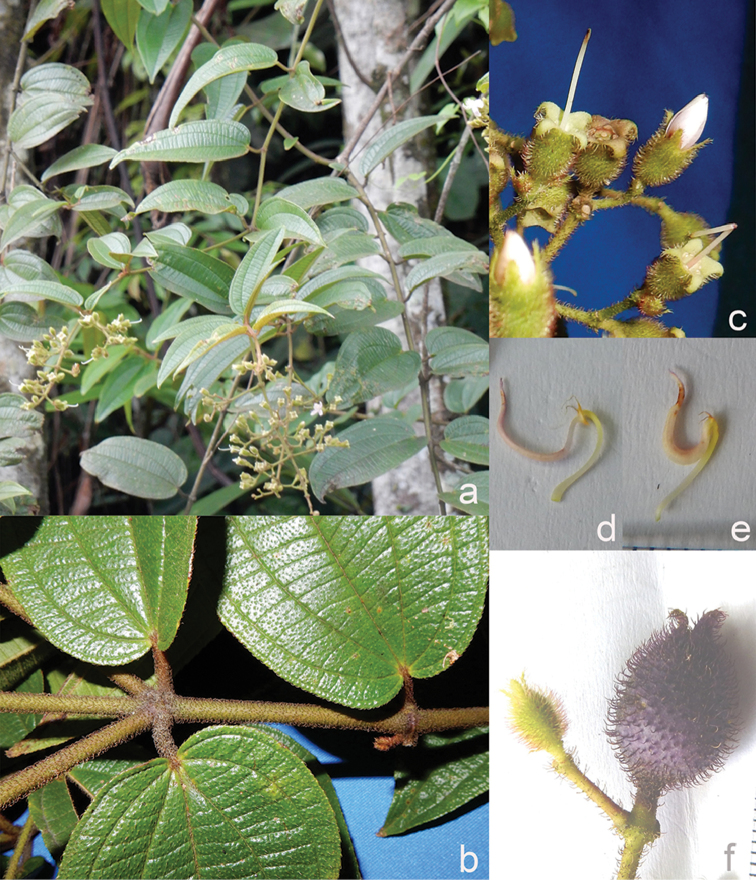
*Dissochaetaporphyrocarpa***a** habit **b** branchlet **c** hypanthium **d** alternipetalous stamen **e** oppositipetalous stamen **f** fruit. Photographs by D. Penneys; voucher: Penneys 2486 (WNC).

**Map 23.**
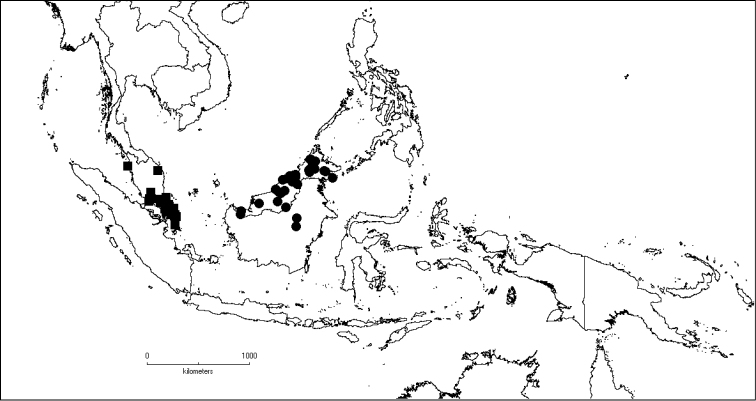
Distribution of *D.porphyrocarpa* (●) and *D.punctulata* (■).

##### Description.

Climbing up to 6 m in height. Branchlets terete, ca. 3 mm in diameter, sparsely brown stellate-puberulous and densely covered with 1–2 mm long curved purplish-tip bristle hairs; nodes swollen, with an interpetiolar ridge; internodes 6.5–8 cm long. Leaves: petioles terete, 8–15 mm long, densely covered with stellate hairs and curved bristle hairs; blades ovate or elliptic, 9.7–12 × 4.7–7.8 cm, membranous, base subcordate, margin entire, apex acute; nervation with 1 pair of lateral nerves and 1 pair of intramarginal nerves; adaxially scabrid, with hairs and scattered curved bristle hairs, abaxially densely covered with curved bristle hairs in most parts, more densely so on midrib and margin. Inflorescences terminal, up to 24 cm long, many-flowered; main axis angular, densely covered with minute stellate hairs and curved purplish-tip bristle hairs; primary axes up to 12 cm long with 3 or 4 nodes, secondary axes 1.5–5 cm long with 1 or 2 nodes nodes, tertiary axes up to 1 cm long when developed with 1 node; bracts linear, 7–8 mm, covered with dense bristle hairs; bracteoles linear, ca. 4 mm long, covered with dense bristle hairs; pedicels densely stellate-furfuraceous and with curved purplish-tipped bristle hairs, 3–5 mm long in central flowers, 1–2 mm long in lateral flowers. Hypanthium campanulate, 5–6 × 3–4 mm, densely covered with brown stellate and curved purplish-tipped bristle hairs; calyx lobes truncate at base, slightly lanceolate, 3–4 mm long, densely bristly, glabrous inside; petal buds conical, 4–8 mm long, apex bristly; mature petals ovate-oblong, 5–6 × 3–5 mm, reflexed, apex obtuse, base clawed, glabrous, purple. Stamens 8, unequal, filaments curved sideways, light green; alternipetalous stamens with ca. 7 mm long filaments, anthers lanceolate, sickle-shaped, thecae 9–10 mm long, apex rostrate, purple, pedoconnective ca. 2 mm long, basal crests minute, triangular or ligular, 1–2 mm long, lateral appendages paired, filiform, 4–5 mm long, white; oppositipetalous stamens with ca. 6 mm long filament, anthers thick, S-shaped, thecae 8–9 mm long, purple, basal crests minute, spur-like, erect, ca. 0.5 mm long, lateral appendages paired, filiform, 1–2 mm long, white. Ovary ⅔ of hypanthium in length, apex villous; style 8–10 mm long, curved at end, glabrous, light green; stigma minute, capitate; extra-ovarial chambers 8, extending to the middle of the ovary. Fruits urceolate, 8–10 × 6–8 mm, densely covered with curved purplish-tip bristle hairs; calyx lobe remnants persistent, reflexed. Seeds ca. 0.5 mm long.

##### Distribution.

Borneo.

##### Ecology and habitat.

Primary mixed Dipterocarp forest or low montane forest on river banks or in open places at 180–900 m elevation.

##### Vernacular name.

*Bang derd* (Kenyah).

##### Note.

The bristles in *D.porphyrocarpa* are distinct as they are eglandular, short and curved; they resemble those of *D.rostrata*. However, bracteoles in *D.porphyrocarpa* are caducous while persistent and conspicuous in *D.rostrata*.

##### Specimens examined.

**MALAYSIA. Sabah**: Keningau, Crocker Range, 15 Aug 1978, Nordin Abas SAN 85957 (K, L); *Ibid.*, Witti Range Area, Ulu Sg. Mantuluk, 17 Jan 1986, Asik Mantor SAN 113279 (K); Nabawan, 12 Nov 1973, Dewol & Abdul Karim SAN 77955 (K, L); Kalabakan, Ulu Segama, 16 Dec 1982, Fidilis SAN 95585 (L); Lahad Datu, Danum Valley, 238 m, 6 Jul 2006, E. Rosalia et al. SAN 145988 (K, L); *Ibid.*, Ulu Segama, 16 Jul 1970, P.F. Cockburn SAN 70925 (K, L); Nabawan, Ulu Sungai Nabawan, 21 Feb 1990, Asik Mantor SAN 128382 (L); Pandewan, Mesopo River, 10 Mar 1986, Sumbing & Asik SAN 114076 (K, L); Pinangah, Imbak River, 200 m, 1 Jul 2000, W.J.J.O. de Wilde, M. Tajudin & M. Postar SAN 143959 (K); Ranau, Ulu Sg. Bidon, 19 Jul 1985, Amin & Ismail SAN 110449 (K, L); *Ibid.*, Mt. Kinabalu, Gurulau Spur, 8 Nov 1915, M.S. Clemens 10789 (PNH); *Ibid.*, Mt. Kinabalu, Gurulau Spur, 27 Nov 1915, M.S. Clemens 10824 (PNH); *Ibid.*, Mt. Kinabalu, Dallas, 914 m, 7 Aug 1931, J. Clemens & M.S. Clemens 30359 (K, L); *Ibid.*, 11 Aug 1931, J. Clemens & M.S. Clemens 26058A (BO); *Ibid.*, 31 Oct 1931, J. Clemens & M.S. Clemens 26028 (BM, BO, K, L); *Ibid.*, Nov 1931, J. Clemens & M.S. Clemens 30357 (BO, K, L); *Ibid.*, Ulu Tungud, 343 m, 27 Jul 2005, L.G. Saw et al. SAN 146068 (L); Tawau, Oct 1922–Mar 1923, A.D.E. Elmer 21426 (BM, BO, C, K, L, P, SING, U). **Sarawak**: Ulu Tawaran, 600 m, G.D. Haviland 1287 (K); Tampassuk, near Kiau, 1066 m, G.D. Haviland 1288 (K); Baram, Ulu Tinjar, Dulit Range, 365 m, 11 Aug 1974, P.K. Chai S.34771 (K, L); Balleh, Mujong, N Semperaja, 200 m, 17 Apr 1964, Othman bin Haron S.19873 (K, L); Belaga, Sungai Semawat, 250 m, 19 Oct 1981, C. Hansen 705 (L); Bintulu, Lumut Range, Ulu Sg. Gelang Bata, 250 m, 19 Sep 1992, Abang Mochtar & P.C. Yii S.65799 (K, L); *Ibid.*, Tubau, Bukit Sekiwa, 180 m, 4 Sep 1986, Abang Mochtar S.53996 (L); Kapit, Batang Balui, Sungai Jitang, 300 m, 28 Feb 1992, Othman et al. S.62032 (L); Marudi, Pulong Tau National Park, Ulu Sungai Baong, 9 May 2007, J. Sang et al. S.98016 (K); Miri, Lambir National Park, 27 Sep 1978, R. George S.40438 (K); *Ibid.*, Gunung Mulu, Melinau Crossing, 200 m, 5 May 1978, G. Argent & B. Coppins 1160 (BM); Padawan, Kampung Braang Wah, 300 m, 8 May 1975, James et al. S.37410 (K, L); *Ibid.*, Bukit Woen, 350 m, 2 Oct 1987, P.C. Yii S.61445 (K, L). **BRUNEI. Belait**: Labi, Mendaram Valley, 20 m, 23 Oct 1989, L.L. Forman & J.B.J. Blewett 1034 (K, L); *Ibid.*, 18 Mar 1991, M.J.S. Sands & R.J. Johns 5445 (K, L); *Ibid.*, Wong Kadir, 150 m, 19 Mar 1993, M.J.E. Coode et al. 7227 (K, L). **Temburong**: Kuala Belalong, Batu Apoi, 17 Nov 1991, C. Hansen 1579 (K, L); Temburong River Valley, 50 m, 27 Apr 1992, R.J. Johns et al. 7389 (K, L). **INDONESIA. East Kalimantan**: West Kutai, River Kiauw, 700 m, 27 Oct 1925, F.H. Endert 4660 (BO, L); *Ibid.*, Long Ibut, 150 m, 10 Nov 1925, F.H. Endert 4754 (BO).

#### 42. Dissochaeta pubescens

Taxon classificationPlantaeMyrtalesMelastomataceae

(Merr.) Karton. comb. nov.

urn:lsid:ipni.org:names:60476843-2

Map 24

Anplectrum beccarianum Cogn. in A.DC. & C.DC., Monogr. Phan. 7: 568. 1891. Type: Malaysia. Sarawak, O. Beccari PB 809 (lectotype, designated here: FI [FI008755, image seen]!; isolectotypes: BR [BR5188895, image seen]!, K [K000859574, K000859575]!).
Diplectria beccariana (Cogn.) Kuntze, Revis. Gen. Pl. 1: 246. 1891.
Dalenia furfuracea Ridl., Kew Bull. 1: 33. 1946. Type: Malaysia. Sarawak: Pengkulu Ampat, G.D. Haviland & C. Hose 144 (lectotype, designated by Nayar 1966, pg. 159: K [K000859506]!; isolectotype: BM [BM001190921, BM001190922]!, SING *n.v.*).
Dalenia beccariana (Cogn.) M.P.Nayar, Kew Bull. 20: 157. 1966.
Dalenia beccariana (Cogn.) M.P.Nayar var. matangensis M.P.Nayar, Kew Bull. 20: 158. 1966. Type: Malaysia. Sarawak: Matang, Aug 1905, H.N. Ridley 12259 (holotype: K [K000859571]!; isotype: SING *n.v.*).

##### Basionym.

*Daleniapubescens* Merr., J. Straits Branch Roy. Asiat. Soc. 86: 338. 1922.

##### Type.

Malaysia. Sabah: Kiau, Mount Kinabalu, 4 Des 1915, M.S. Clemens 10301 (lectotype, designated here: A [A00072194, image seen]!).

**Map 24.**
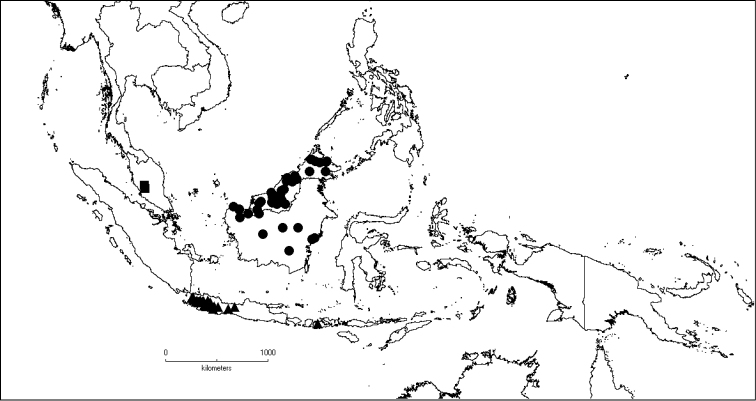
Distribution of *D.pubescens* (●), *D.rectandra* (■) and *D.vacillans* (▲).

##### Description.

Climbing up to 20 m in height; branchlets terete, 4–5 mm in diameter, covered with brown stellate-furfuraceous hairs; nodes swollen, with raised annular crest-like interpetiolar ridge, apex rounded, up to 6 mm high, densely covered with stellate hairs; internodes 8–21 cm long. Leaves: petioles terete, 15–35 mm long, densely brown stellate-furfuraceous; blades suborbicular, ovate to elliptic, (8–)15–30 × (4–)10–20 cm, subcoriaceous, base rounded to cordate, margin entire, apex acute or acuminate, tip 0.5–1 cm long; nervation with 1 or 2 pairs of lateral nerves and 1 pair of intramarginal nerves; adaxially glabrous, glossy green, abaxially densely covered with brown pubescent hairs. Inflorescences terminal, up to 80 cm long, many-flowered; main axis angular, densely covered with brown stellate hairs; primary axes 35–76 cm long with 6–10 nodes, secondary axes up to 18 cm long with 2–4 nodes, tertiary axes up to 4 cm long with 1 or 2 nodes, quarternary axes when developed up to 8 mm long with 1 node; bracts either crest-like, erect, as wide as the rachis, 2–3 mm high or somewhat ligulate, 1.5–2 mm long, both types caducous, oftenly seen only on the terminal cymules; bracteoles linear or ligulate, 1–2 mm long, caducous; pedicels densely stellate-furfuraceous, brown, 9–10 mm long in central flowers, 5–8 mm long in lateral flowers. Hypanthium campanulate to suburceolate, 6–10 × 4–6 mm, covered with stellate pubescent hairs; calyx lobes truncate, without 4 distinct tips, ca. 0.5 mm long, forming calyptra enveloping petal buds, calyptra conical, up to 9 mm long, with acute tip, densely brown pubescent; petal buds conical, 7–8 mm long, apex acute; mature petals ovate, 7–8 × ca. 4 mm, base clawed, apex acute to obtuse, white to pink or purple. Stamens 8, unequal, filaments straight, white; alternipetalous stamens staminodial with 4–5 mm long filaments, anthers rudimentary, thecae ca. 2 mm long, slender, basal crest triangular 1–1.5 mm long, lateral appendages paired, thin, flat, filiform, 4–5 mm long; oppositipetalous stamens with 4–5 mm long filaments, anthers thick, curved, hooked- or S-shaped, thecae 8–9 mm long, apex rostrate, yellow, basal crest shortly triangular, ca. 0.5 mm long, lateral appendages absent or minute points. Ovary ⅔ of hypanthium in length, apex glabrous; style 13–15 mm long, curved at the end, slender, glabrous, white; stigma minute; extra-ovarial chambers 4, oppositipetalous, reaching to the base of the ovary. Fruits ovoid-urceolate, rarely subglobose, 10–12 × 6–8 mm, brown stellate-pubescent; calyx lobes remnant persistent, erect. Seeds ca. 0.5 mm long.

##### Distribution.

Borneo.

##### Ecology and habitat.

Lowland dipterocarp forest or low montane forest, roadside along the forest edges, river banks or logged forests at 25–900 m elevation.

##### Vernacular name.

*Akar* (Sintang).

##### Note.

*Dissochaetapubescens* is recognised by its robust leaf blades (up to 30 × 20 cm) and inflorescences (up to 80 cm long). The typical indumentum of most parts resembles that of *D.axillaris* and *D.latifolia*. In bud, the petals of *D.pubescens* are enclosed by a thin calyptra, which will fall off during anthesis. The calyptra is similar to that of *D.pulchra*. The epithet *pubescens*, the first available heterotypic synonym, is used here for the new combination name because an older *Dissochaetabeccariana* already exists.

##### Specimens examined.

**MALAYSIA. Sabah**: Labuk Sugut, Bukit Timimbang, 21 Sep 1984, Sigin et al. SAN 67567 (K); Lahad Datu, Ulu Segama, 14 Aug 1986, Joseph SAN 116983 (K); Nabawan, Nabawan–Pandewan Road, 12 Mar 1990, Sumbing SAN 128066 (K); Sandakan, Batu Lima, Sep-Dec 1920, M. Ramos BS 1585 (BM, BO, K, L, P); Labuk Road, 137 m, 8 Dec 1971, Dewol, Leopold & Shea SAN 74565 (K, L); Ranau, Mount Kinabalu, Kiau, 4 Dec 1915, M.S. Clemens 10301 (A); *Ibid.*, Dallas, 914 m, 1 Oct 1931, J. Clemens & M.S. Clemens 26667 (BM, K, L); *Ibid.*, J. Clemens & M.S. Clemens 30389 (K, L); *Ibid.*, Ulu Tungud, Gunung Monkobo, 296 m, L.G. Saw et al. SAN 146684 (L). **Sarawak**: 26 Oct 1894, G.D. Haviland & C. Hose 144E (L); 1871, O. Beccari PB 809 (K); Kuching, Matang, Aug 1905, H.N. Ridley 12259 (K); *Ibid.*, 17 Mar 1955, W.M.A. Brooke 9714 (L); Pengkulu Ampat, G.D. Haviland 144 (BM, K); Kapit, Balleh, Ulu Mujong, 26 Mar 1964, Asah ak Unyong S.21200 (K); *Ibid.*, 17 Apr 1964, Othman bin Haron S.19946 (BO, K, L); Baram, Batang Tinjar, Ulu Sg. Sekiwa, 152 m, 1 Sep 1974, S. Tong S.35025 (K, L); *Ibid.*, Mount Dulit, 300 m, 16 Aug 1932, P.W. Richards 1301 (K, L); *Ibid.*, Ulu Sungai Melinau, 122 m, 24 Jun 1961, J.A.R. Anderson S.4071 (K, L); Bintulu, Ulu Segan, 244 m, 24 Aug 1968, Ilias Paie S.27216 (BO, K, L); Kapit, Bukit Raya, 518 m, 4 May 1969, E. Soepadmo & P.K. Chai S.28175 (K, L); *Ibid.*, Pelagus, 7 Jul 1979, B. Lee S.40230 (K, L); Limbang, Ulu Mendamit, Sg. Ensungei, 14 Sep 1980, R. George et al. S.42897 (K, L); Lundu, Mount Poi, 1929, J. Clemens & M.S. Clemens 20270 (K); Miri, Gunong Mulu, Melinau Gorge, 375 m, 2 Feb 1978, C. Hansen 244 (K); Marudi, Pulong Tau, 15 May 2007, R.P. de Kok et al. S.97859 (K); Tatau, Batang Anap, 240 m, 12 Jun 1982, Abang Mochtar S.41775 (K, L); Betong, Bukit Sadok, 15 Oct 1982, Banyeng & Ilias Paie S.45092 (K, L); Sibu, Ulu Sungai Pasai, Bukit Tanggi, 50 m, 29 Mar 1992, P.C. Yii & Jegong S.64404 (K, L); Sri Aman, Batang Ai, 350 m, 12 Dec 1994, P.C. Yii et al. S.69527 (L); Batang Balui, Ulu Sungai Elyak, 950 m, 12 Mar 1989, P.C. Yii S.56745 (K); *Ibid.*, P.C. Yii S.56746 (K); Tubau, Merurong, 16 Oct 1984, Othman et al. S.48949 (K); *Ibid.*, Batu Laga, 960 m, 19 Mar 1989, P.C. Yii S.56899 (AAU, L); Ulu Simunjan, G. Angkong, 23 Sep 1975, Martin & Othman S.36955 (K, L). **BRUNEI. Belait**: Labi, Mendaram, 30 m, 20 Jun 1995, A. Kalat et al. BRUN 16786 (K, L). **Temburong**: Pagon, 150 m, 22 Jul 1990, K.M. Wong 1869 (K); Kuala Belalong, 25 m, 22 Jun 1989, P.C. Boyce et al. 392 (K, L). **Tutong**: Ulu Tutong, Bukit Bahak, 210 m, 17 Dec 1991, D.W. Kirkup et al. 578 (K). **INDONESIA. Central Kalimantan**: Barito Ulu, 25 May 1990, C.E. Ridsdale PBU 185 (BO, L). **East Kalimantan**: West Kutai, Long Liang Beng, 250 m, 1 Sep 1925, F.H. Endert 3060 (BO, K, L); Samarinda, ITCI concession area, 250 m, 7 Jun 1989, M.M.J. van Balgooy 5840 (L); Wanariset, Samboja-Semoi road, 50 m, 7 May 1991, Ambriansyah & Z. Arifin W728 (K, L). **West Kalimantan**: Pontianak, Bentiang, Gunung Bayuh, 750 m, 31 Oct 1980, G. Shea 27146 (BO, K, L); Sintang, Bukit Baka, Sungai Ella, 320 m, 21 Oct 1993, A.C. Church et al. 273 (BO, L).

#### 43. Dissochaeta pulchra

Taxon classificationPlantaeMyrtalesMelastomataceae

(Korth.) J.F.Maxwell, Gard. Bull. Singapore 33: 318. 1980.

Fig. 24 , Map 22

Dalenia pulchra Korth. in Temminck, Verh. Nat. Gesch. Ned. Bezitt., Bot.: tab. 58, 1842.
Dalenia speciosa Korth. in Temminck, Verh. Nat. Gesch. Ned. Bezitt., Bot.: 244. 1844, *nom. superfl*. Type: based on Daleniapulchra Korth.
Dalenia korthalsii Blume, Mus. Bot. 1(3): 39. 1849, *nom. superfl*. Type: based on Daleniapulchra Korth.
Anplectrum macrophyllum Ridl., Kew Bull. 1: 31. 1946. Type: Malaysia. Sarawak: Baram, Pata River, Nov 1894, C. Hose 478 (lectotype, designated here: K [K000859569]!; isolectotypes: BM!, E [E00288103, E00288104]!, K [K000859570]!, L [L0537213]!, P [P02274828, image seen]!, PNH [PNH24835, image seen], SING *n.v.*).

##### Type.

Indonesia. Central Kalimantan: Kapoeas-Barito, Tewe Rivier, P.W. Korthals s.n. (lectotype, designated here: L [L0537210]!; isolectotypes: L [L0537211, L0729471, L0729472, L0729473]!, P [P02274827, image seen]!).

**Figure 24.**
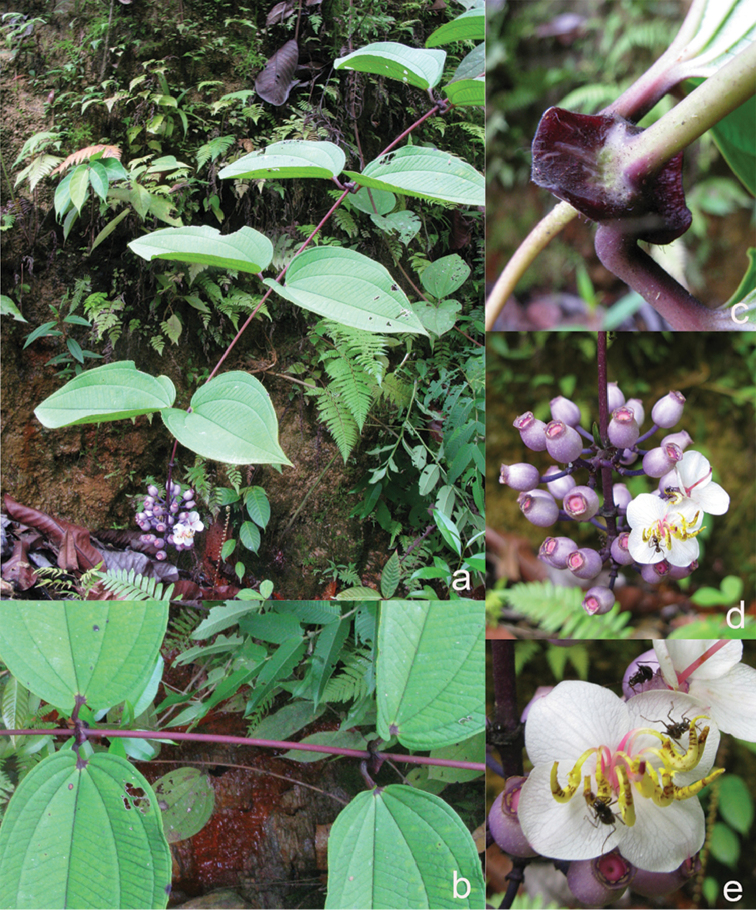
*Dissochaetapulchra***a** habit **b** branchlet **c** leaf node **d** infrutescence/inflorescence **e** flower. Photographs by J. Henrot.

##### Description.

Climbing up to 13 m in height; branchlets terete, 2–3 mm in diameter, glabrous, smooth; nodes swollen, with pulvinate interpetiolar ridge, crest-like or laminar up to 5 mm wide, dark purple; internodes 11.5–15 cm long. Leaves: petioles flattened, 15–25 mm long, glabrous; blades ovate to suborbicular, 14–20 × 8.5–13 cm, membranous, base broadly cordate, margin slightly serrulate, apex acuminate, tip 1.5–2 cm long; nervation with 2 pairs of lateral nerves and 1 pair of intramarginal nerves; adaxially glabrous, abaxially glabrous and partially scabrid, with a pair of glandular patches at base. Inflorescences terminal, up to 25 cm long, many-flowered; main axis angular, glabrous, purplish-blue; primary axes up to 13 cm long with 4 or 5 nodes, secondary axes 1–3 cm long with 1 or 2 nodes, tertiary axes 0.5–1 cm long with 1 node; bracts lanceolate, 6–10 mm long, furfuraceous, caducous; bracteoles subulate, 1–2 mm long; pedicels glabrous, 5–7 mm long in central flowers, 2–5 mm long in lateral flowers. Hypanthium campanulate-tubular, 8–9 × 3–4 mm, glabrous, purplish; calyx lobes truncate with undulate tips, 1–2 mm long, forming thin calyptra enveloping petal buds, calyptra conical, up to 8 mm long, with acute tip, glabrous, purple; petal buds conical, 4–6 mm long; mature petals ovate, 8–12 × 7–8 mm, reflexed, apex acute, base clawed, glabrous, white or white pinkish. Stamens 8, subequal, filaments curved sideways, white; alternipetalous stamens with 6–7 mm long filaments, anthers narrow, curved, sickle-shaped, thecae 8–10 mm long, bright yellow, pedoconnective ca. 0.5 mm long or undeveloped, basal crest thin, triangular or ligular, 0.8–1 mm long, lateral appendages paired, filiform, 1–2 mm long; oppositipetalous stamens with 6–7 mm long filaments, anthers S-shaped, thecae 7–8 mm long, thick, yellow, basal crest ligular or spur-like, ca. 0.5 mm long, lateral appendages paired, small auricles or bifid, ca. 1 mm long. Ovary half as long as hypanthium, apex glabrous; style slender, 12–14 mm long, reddish, turning white apically, glabrous; stigma minute, purple; extra-ovarial chambers 8, the 4 alternipetalous ones extending nearly to the base of the ovary, the 4 oppositipetalous ones extending to the middle of the ovary. Fruits ovoid-urceolate, 9–10 × ca. 7 mm, glabrous, blue-violet; calyx lobe remnants persistent, widened. Seeds ca. 0.75 mm long.

##### Distribution.

Borneo.

##### Ecology and habitat.

Primary or secondary forest, low montane forest, river banks in riparian forest at 80–1000 m elevation.

##### Vernacular names.

*dangkong* (kadazan putatan); *buak penan* (Kenyah); *kunceng badak gaka* (Tunjung Benua).

##### Notes.

1. *Dissochaetapulchra* is easily recognised by its thin, glabrous, ovate-suborbicular leaf blade with minutely serrulate margin; all other species have entire margins. The crest-like interpetiolar ridge is similar to *D.stipularis* and *D.pubescens*, but there are differences in shape and number of the fertile stamens. This species also has abaxially glandular patches at the base of the leaves similar to *D.beccariana*, *D.glabra*, *D.glandulosa* and *D.laevis*.

2. The epithet *pulchra* is considered as a valid name for the species as it is based on Korthals’ plate 58, published in 1842, two years earlier than the description in which the epithet *speciosa* was used (Korthals 1844, Nayar 1966). Blume (1849) tried to settle the matter by giving a new name, *D.korthalsii* to the species. It follows that *D.speciosa* and *D.korthalsii* are both superfluous names under the Code and are therefore illegitimate. Both *D.pulchra* and *D.speciosa* have been used by various authors, but *D.speciosa* appears to have been the most widely used (Nayar 1966).

##### Specimens examined.

**MALAYSIA. Sabah**: Beaufort, Gunung Lumaku, 300 m. 5 Mar 1969, H.P. Nooteboom 1138 (L); *Ibid.*, 6 Mar 1969, H.P. Nooteboom 1198 (L); *Ibid.*, Halogilat, 10 May 1973, Dewol & Karim SAN 77594 (L); *Ibid.*, Rayoh, 15 Aug 1972, Saikeh SAN 72234 (L); Beluran, Bukit Luminitong, 11 Mar 1982, Aban Gibot SAN 94467 (L); Kalabakan, Gunong Rara, 488 m, 15 Apr 1972, Chow SAN 75687 (L); Keningau, Pinangah FR, 24 Sep 1985, Asik Mantor SAN 110269 (K); *Ibid.*, Imbak River, 200 m, 6 Jul 2000, W.J.J.O. de Wilde, M. Tajudin & Good SAN 143924 (K); Lahad Datu, Ulu Sungai Bole, 1 Mar 1988, Dewol Sundaling et al. SAN 123756 (L); Nabawan, Sungai Pingas-Pingas, 27 May 1986, Fidilis & Asik SAN 115864 (L); Pensiangan, Ponontomon, 19 Aug 1994, Asik Mantor SAN 139507 (L); Ranau, Kampung Monggis, 23 Feb 1998, M. Rumutom 473 (K); *Ibid.*, Telupid, Tampias, 14 Dec 1974, Aban & Kodoh SAN 81101 (L); Sandakan, Telupid-Ranau Road, 91 m, 15 Aug 1978, L. Madani SAN 89206 (L); Sipitang, Muruk Miau, 175 m, 27 Apr 1997, J. Kulip et al. SAN 133876 (L); Tenom, near Pangi, 150 m, 23 Dec 1968, S. Kokawa & M. Hotta 2603 (L); *Ibid.*, Gunong Lumaku, 12 Sep 1991, Maikin et al. SAN 132681 (L); *Ibid.*, Crocker Range, Rayoh, 26 Sep 1974, Dewol & Karim SAN 78319 (L); Tongod, Maliau Valley, 250 m, 16 Jul 2001, M. Postar, et al. SAN 144145 (L). **Sarawak**: Balleh, Ulu Mujong, Sungai Sebatang, 18 Apr 1964, Othman bin Haron S.21105 (BO, L); Baram, Pata River, Nov 1894, C. Hose 478 (BM, L, P, PNH); *Ibid.*, Selungo, 25 Nov 1914, Native Collector 2819 (PNH); *Ibid.*, Tinjar River, Sg. Bok, Long Teru, 50–100 m, 10 Mar 1969, M. Hotta 6113 (L); Belaga, Linau, Sg. Bunut, 3 Nov 1982, B. Lee S.45415 (L); *Ibid.*, Ulu Belaga, Sungai Semawat, 250 m, 17 Oct 1981, C. Hansen 670 (L); *Ibid.*, Bukit Kuang, 900 m, 8 Mar 1989, P.C. Yii S.56571 (L); *Ibid.*, Sungai Berangan, 600 m, 18 Aug 1995, J.T. Pereira et al 246 (L); *Ibid.*, Batu Laga, 600 m, 24 Jun 1995, Lai et al. S.72466 (K); *Ibid.*, Sungai Murum, 14 Aug 2001, Yahud S.84494 (L); Kapit, Upper Rejang River, 1929, J. Clemens & M.S. Clemens 21140 (BO); *Ibid.*, J. Clemens & M.S. Clemens 21579 (BO); *Ibid.*, Batang Baleh, Sungai Serani, 200 m, 5 May 1991, Runi, et al. S.63183 (K, L); *Ibid.*, Ulu Balui, 100-500 m, 17 Aug 1995, J.B. Sugau 165B (L); Maputi, 21 Jun 1955, W.M.A. Brooke 10091 (BM, L); Marudi, Bok-Tisam, Bukit Mentagai, 122 m, 10 May 1965, Sibat ak Luang S.23280 (L); *Ibid.*, Pulong Tau National Park, Long Lobang River, R.P. de Kok et al. S.97859 (K). **BRUNEI. Temburong**: Kuala Belalong, Batu Apoi Forest Reserve, 20 Nov 1991, C. Hansen 1593 (L); *Ibid.*, Bukit Gelagas, 350 m, 20 Oct 1991, D.A. Simpson & M. Marsh 2510 (L). **INDONESIA. Central Kalimantan**: Tewe River, P.W. Korthals s.n. (L, P). **East Kalimantan**: Sungai Bocleng, 1898, Amdjah 115 (BO); Pamaluan, L.M.R. Rutten 103 (U); West Kutai, Long Gemelei, 200 m, 28 Aug 1925, F.H. Endert 2933 (BO, L); *Ibid.*, Long Puhus, 80 m, 9 Aug 1925, F.H. Endert 2425 (BO, L); *Ibid.*, Mahakam Ulu, Hulu Riam Halo, 100 m, 28 Jun 1975, H. Wiriadinata 691 (BO); *Ibid.*, Tabang, 160 m, 20 Dec 1980, M. Kato & H. Wiriadinata B-7131 (BO, L); *Ibid.*, Gunung Kongkat–Gunung Kongbotak, 29 Jan 1981, M. Kato & H. Wiriadinata B-5202 (L); Kutai National Park, Kayu Mas, 130 m, 25 Jul 1986, S. Tagawa, E. Suzuki & S. Miyagi 077 (BO); *Ibid.*, 200 m, 19 Aug 1986, S. Tagawa, E. Suzuki & S. Miyagi 587 (BO); Balikpapan, Kenangan, 700 m, 13 Aug 1974, J. Dransfield 4414 (BO, L); Sangkulirang, Karangan River, Batu Pondong, 100 m, 3 Sep 1957, A.J.G.H. Kostermans 13690 (BO, L); Sebulu, 28 Dec 1978, G. Murata et al. B-587 (BO); *Ibid.*, G. Murata et al. B-600 (BO, L); Kenangan, PT ITCI Area, 100 m, 1 Mar 1991, K. Sidiyasa 719 (K, L); Berau, Inhutani I area, 175 m, 6 Oct 1996, P.J.A. Kessler et al. Berau 128A (L); Sungai Nakan, 13 Jun 1986, Arbainsyah AA1921 (BO). **North Kalimantan**: Long Bawan, Krayan, 1100 m, 28 Jul 1981, M. Kato, M. Okamoto & E.B. Walujo B-10052 (BO, L); Bulungan, Seturan River, 200 m, 5 Oct 1999, Ismail & Z. Arifin BRF 1714 (BO); Malinau, Pujungan, Kayan Mentarang National Park, 1000 m, 24 Jul 1992, J.A. McDonald & Ismail 3600 (BO, L); *Ibid.*, 200-500 m, 5 Apr 2002, M. Koizumi & Lalo 317 (BO, L); Ulu Sebuku, Aug 1912, Amdjah 404 (BO, L). **South Kalimantan**: Tabalong, 750 m, 6 Jul 2000, K. Sidiyasa & Z. Arifin 2010 (L). **West Kalimantan**: Sungai Blu’u, 1896, Jaheri 1104 (BO); Penigin, 1896, Jaheri 330 (BO).

#### 44. Dissochaeta punctulata

Taxon classificationPlantaeMyrtalesMelastomataceae

Hook .f. ex Triana, Trans. Linn. Soc. London 28: 83. 1872.

Map 23

##### Type.

Malaysia. Peninsular Malaysia, Malacca, W. Griffith KD 2291 (lectotype, designated here: K [K000859531]!; isolectotype: BM!).

##### Description.

Climbing up to 27 m in height; branchlets terete, 3–5 mm in diameter, sparsely furfuraceous with minute red-brown hairs; nodes swollen, with interpetiolar line; internodes 2–7 cm long. Leaves: petioles terete, 5–15 mm long, striate, glabrescent; blades ovate or ovate-elliptic, 4.2–11 × 2.5–5.5 cm, subcoriaceous, base rounded to cuneate, margin entire, apex acuminate, tip ca. 0.5 cm long; nervation with 1 pair of lateral nerves and 1 pair of intramarginal nerves; adaxially glabrous, abaxially sparsely stellate punctate. Inflorescences terminal and in the upper leaf axils, up to 15 cm long, many-flowered; main axis densely stellate-furfuraceous; primary axes 15–20 cm long with 4–6 nodes, secondary axes 3–10 cm long with 2–4 nodes, tertiary axes 1–2 cm long with 1 or 2 nodes; bracts linear, ca. 4 mm long, densely furfuraceous, caducous; bracteoles linear, 2–3 mm long, densely furfuraceous, caducous; pedicels densely furfuraceous, 2–3 mm long in central flowers, 1–2 mm long or subsessile in lateral flowers. Hypanthium campanulate, but urceolate to subglobose at first stage, enclosing the petal bud, 6–10 × 5–6 mm, densely red-brown tomentose; calyx lobes triangular with acute tips, 1–2 mm long, densely stellate-furfuraceous, densely pubescent inside; petal buds conical, 2–4 mm long; mature petals obovate, ca. 10 × 5–7 mm, reflexed, base clawed, apex rounded, glabrous with ciliate margin, white. Stamens 8, unequal to subequal, filaments curved sideways, white yellowish; alternipetalous stamens with (5–)9–10 mm long filaments, anthers curved, sickle-shaped, slender, thecae 9–12 mm long, flexed, maroon, pedoconnective 3–4 mm long, basal crest fimbriate, somewhat branched, 4–5 mm long, lateral appendages paired, filiform or fimbriate, 7–8 mm long; oppositipetalous stamens with 5–8 mm long filaments, anthers S-shaped, thick, thecae 7–8 mm long, yellow, basally erose or bifid or with a fimbriate crest, somewhat branched, 3–5 mm long, lateral appendages paired, irregularly filiform, 6–8 mm long. Ovary ¾ of hypanthium in length, apex villous; style slender, 5–8 mm long, curved at top, white; stigma minute, villous; extra-ovarial chambers absent or not developed. Fruits urceolate or subglobose, 8–10 × 4–7 mm, sparsely to densely furfuraceous; calyx lobe remnants persistent, reflexed. Seeds ca. 0.75 mm long.

##### Distribution.

Malay Peninsula and Sumatra (Riau Archipelago).

##### Ecology and habitat.

Disturbed lowland primary forest or secondary forest at 300–600 m elevation.

##### Vernacular name.

Peninsular Malaysia: *akar muroyan busuh* (Malacca).

##### Specimens examined.

**MALAYSIA. Johor**: Feb 1890, H.N. Ridley 2016 (BM); Puiron, Nov 1891, N. Cantley 62 (K); Gunung Pulai, 24 Apr 1922, M. Nur & Kiah SFN 7799 (BO, K); *Ibid.*, 18 Dec 1922, G.A. Best SFN 7849 (BO); *Ibid.*, 4 Sep 1971, Chan FRI 17660 (K, L); *Ibid.*, 550 m, 29 Jan 1978, J.F. Maxwell 78-23 (L); *Ibid.*, 500 m, 18 Jan 1981, J.F. Maxwell 81-15 (L); Bukit Paloh, 30-60 m, 9 Apr 1958, M. Shah & Kadim 431 (K, L); Endau, Kampong Hubong, 18 Jul 1959, Kadim & M. Noor 357 (BO, K); Mawai-Jemaluang Road, 5 May 1935, E.J.H. Corner SFN 29377 (BO, K); Labis, 27 Apr 1986, L.G. Saw FRI 34336 (K, L). **Malacca**: W. Griffith s.n. (BO, K, L, P); W. Griffith KD 2291 (BM, K); D.F.A. Hervey s.n. (BM); 16 May 1886, A.C. Maingay KD 789 (1220A) (K, L); Sungai Hudang, 1892, H.N. Ridley 548 (BM, K). **Negri Sembilan**: Tebong, 19 Jan 1917, H.N. Ridley s.n. (K); Jelebu, Pasoh Forest, 80-120 m, 10 May 1996, E.C. Gardette 1850 (K, L); *Ibid.*, E.C. Gardette 1850B (K, L). **Penang**: G.W. Walker 30 (BM, K). **Terengganu**: Gunong Padang, Ulu Brang, 600 m, 23 Sep 1969, T.C. Whitmore FRI 12786 (K, L); *Ibid.*, T.C. Whitmore FRI 12794 (K, L). **SINGAPORE.** N. Cantley s.n. (BM); Bukit Timah, 21 Sep 1890, H.N. Ridley 3858 (BM, L); *Ibid.*, 24 Feb 1977, J.F. Maxwell 77-88 (L); Changi, 26 May 1891, H.N. Ridley s.n. (BM); Punggol, Mar 1878, R.W. Hullett 505 (K); Pierce Reservoir, 25 Jun 1955, J. Sinclair SFN 40649 (BO, K, L, P); *Ibid.*, 13 May 1981, J.F. Maxwell 81-91 (L); Tanjong Gul, 5 Feb 1950, J. Sinclair s.n. (L, P); Bukit Mandai, 1893, Anon 4803 (BM); Seletar, 22 Jul 1892, H.N. Ridley 3918 (BM). **INDONESIA. Riau Archipelago**: Lingga Islands, Rejai Island, 20 m, 19 Aug 1919, H.A.B. Bünnemeijer 7633 (BO, K, PNH); Senggarang Island, J.E. Teijsmann s.n. (BO, L); Bintan Island, Tanjung Pinang, J.E. Teijsmann s.n. (BO); *Ibid.*, Gunung Bintan, 300 m, 19 Jun 1919, H.A.B. Bünnemeijer 6162 (BO); *Ibid.*, Mount Kijang, 25 m, 30 Jun 2013, D. Girmansyah 1847 (BO).

#### 45. Dissochaeta rectandra

Taxon classificationPlantaeMyrtalesMelastomataceae

Karton. sp. nov.

urn:lsid:ipni.org:names:60476831-2

Fig. 25 , Map 24

##### Type.

Malaysia. Pahang: Fraser’s Hill, 3 Aug 1967, J.C. Carrick 1606 (holotype L [L.2533494]!; isotypes: K!, KLU *n.v.*, L [L.2533495]!, SING *n.v.*).

**Figure 25.**
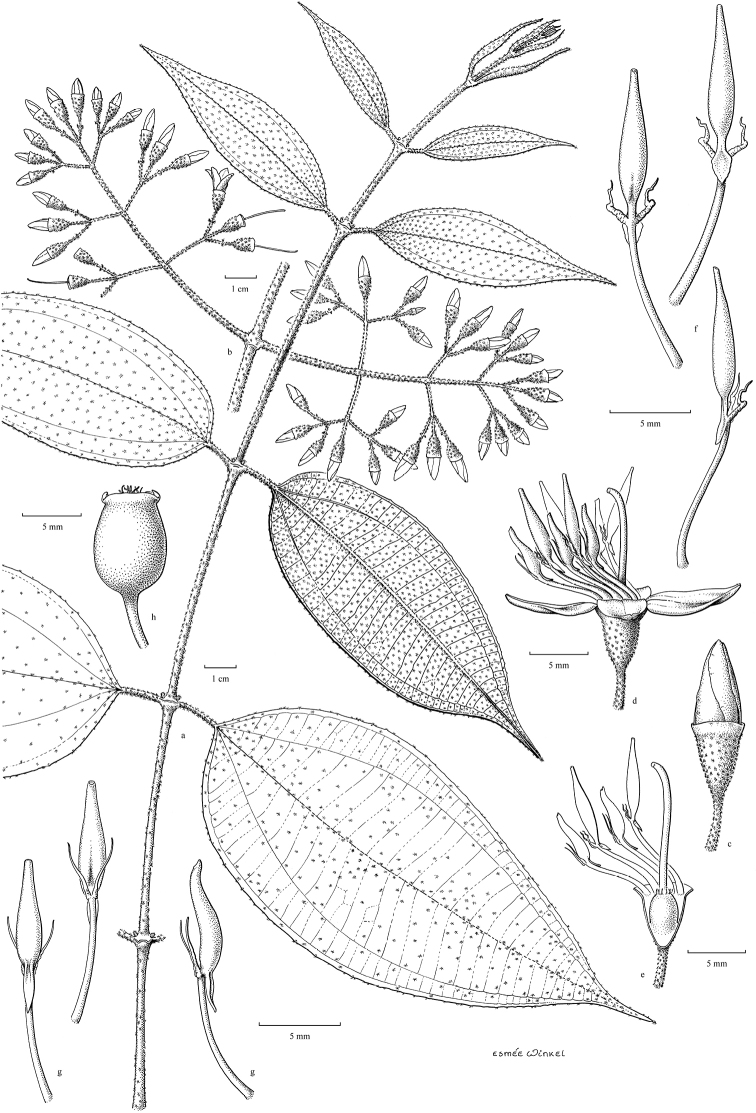
*Dissochaetarectandra***a** habit **b** inflorescence **c** hypanthium **d** open flower **e** ovary **f** alternipetalous stamens **g** oppositipetalous stamens **h** fruit. [drawn from Carrick 1606 (L).

##### Diagnosis.

Resembles *D.bakhuizenii* Veldkamp. Leaf blade margin entire or thinly serrate with glabrous to sparsely stellate punctation below. Hypanthium campanulate-angular, 6–8 × 3–3.5 mm, glabrescent to sparsely stellate-puberulous, calyx lobes truncate with 4 small undulate points, 1–1.5 mm long, glabrous outside, stellate-furfuraceous inside. Stamens 8, subequal, filaments and anthers straight upwards, alternipetalous stamens with triangular basal crest and paired, filiform, lateral appendages, oppositipetalous stamens with ligular basal crest and paired filiform appendages. Fruits with mammiform apex.

##### Description.

Climbing up to 7.5 m. Branchlets terete, 3–5 mm in diameter, glabrescent; nodes swollen, interpetiolar ridge distinct with annular crest-like ridge; internodes 5–12 cm long. Leaves: petioles flattened, 10–23 mm long, stellate-punctate; blades elliptic or ovate-elliptic, 9.5–18 × 5–9.2 cm, membranous, base rounded to slightly cuneate, margin entire or thinly serrate, apex acuminate, tip 1–2 cm long; nervation with 1–2 pairs of lateral nerves and 1 pair of intramarginal nerves; adaxially glabrous, dark green, abaxially glabrous to brownish stellate-punctate, maroonish and glabrous when young. Inflorescences terminal, up to 40 cm long, many-flowered; main axis terete, glabrescent to sparsely stellate-puberulous; primary axes up to 33 cm long with 5 or 6 nodes, secondary axes up to 10 cm long with 3 or 4 nodes, tertiary axes 1–2.5 cm long with 1 or 2 nodes, quarternary axes when developed up to 0.8 cm long with 1 node; bracts linear, 2–2.5 mm long, stellate-furfuraceous, caducous; bracteoles subulate, 1–1.5 mm long, stellate-furfuraceous; pedicels stellate-furfuraceous, 4–6 mm long in central flowers, 2–3 mm long in lateral flowers. Hypanthium campanulate-angular, 6–8 × 3–3.5 mm, glabrescent to sparsely stellate-puberulous; calyx lobes truncate with 4 small undulate points, 1–1.5 mm long, glabrous outside, stellate-furfuraceous inside; petal buds conical, 4–9 × 2–3 mm, glabrous; mature petals obovate to suborbicular, 10–11 × 8–9 mm, base clawed, margin ciliate, apex rounded, glabrous, pink to dark purple. Stamens 8, subequal, filaments straight, white pinkish; alternipetalous stamens with 8–9 mm long filaments, anthers lanceolate, thecae 7–8 mm long, straight, yellow, pedoconnective 1.5–2 mm long, basal crests triangular, 1–1.5 mm long, acute, lateral appendages prolonged from basal crest, paired, filiform, 2–2.5 mm long; oppositipetalous stamens with filaments 7–8 mm long, bent at top, anthers oblong-lanceolate, thecae 6–7 mm long, straight, yellow, basal crest ligular, 1–1.5 mm long, apex narrow, lateral appendages paired, filiform, 2–3 mm long. Ovary half as long as hypanthium, apex pubescent, bristly; style 12–13 mm long, straight, curved at top, glabrous; stigma minute, capitate; extra-ovarial chambers 8, extending from the middle to the base of the ovary. Fruits urceolate, 8–10 × 6–9 mm, apex mammiform, bristly, rest glabrous; calyx lobes persistent, erect. Seeds ca. 0.5 mm long.

##### Distribution.

Peninsular Malaysia (Selangor and Pahang).

##### Ecology and habitat.

Montane forest, open areas along road sides at 1280–1800 m elevation.

##### Etymology.

The species is named after the orientation and shape of its stamens, erect, straight upwards when mature.

##### Note.

*Dissochaetarectandra* resembles *D.bakhuizenii* in having 8 fertile stamens, a triangular basal crest (alternipetalous) and ligular basal crest (oppositipetalous). *Dissochaetarectandra* has thinly serrate leaf margins, large flowers and longer lateral appendages on the stamens (vs. entire margins of leaf blades, smaller flowers and short to absent lateral appendages in *D.bakhuizenii*). This species is restricted to the montane forest of Fraser’s Hill (Pahang) and the Genting Highlands (Selangor).

##### Specimens examined.

**MALAYSIA. Pahang**: Fraser’s Hill, 3 Aug 1967, J.C. Carrick 1606 (K, L); *Ibid.*, Richmond, 1280 m, 16 Apr 1955, J.W. Purseglove 4112 (L). **Selangor**: Genting Highlands, Gunong Ulu Kali, 1800 m, 3 Jun 1978, J.F. Maxwell 78-307 (L); *Ibid.*, 1500 m, 3 Jun 1978, J.F. Maxwell 78-312 (L).

#### 46. Dissochaeta rostrata

Taxon classificationPlantaeMyrtalesMelastomataceae

Korth. in Temminck, Verh. Nat. Gesch. Ned. Bezitt., Bot. 239. 1844.

Map 25

Anplectrum korthalsii Triana, Trans. Linn. Soc. London 28: 85. 1872. Type: Based on Dissochaetarostrata Korth.
Dissochaeta hirsuta Hook.*f.* ex Triana, Trans. Linn. Soc. London 28: 83. 1872. Type: Malaysia. Sarawak: Labuan, J. Motley s.n. (holotype: K [K000859629]!).
Diplectria korthalsii (Triana) Kuntze, Revis. Gen. Pl. 1: 246. 1891.
Dissochaeta setosa O.Schwartz, Mitt. Inst. Allg. Bot. Hamburg 7: 250. 1931. Type: Indonesia. West Kalimantan: Lebang Hara 150 m elev., 1 Jan 1925, J. Winkler 1167 (lectotype, designated here: HBG [HBG522818, image seen]!; isolectotypes: BO [BO1747972]!, HBG [HBG522819, HBG522820, images seen]!, L [L0008893]!).
Macrolenes ruttenii Bakh.*f.*, Contr. Melastom.: 210. 1943. Type: Indonesia. East Kalimantan: Samarinda, Soengei Boengaloen, 12 Nov 1911, L.M.R. Rutten 535 (holotype: U [U0004012]!).
Dissochaeta rostrata Korth. var. hirsuta (Hook.*f.* ex Triana) J.F.Maxwell, Gard. Bull. Singapore 33: 319. 1980.
Dissochaeta rostrata Korth. var. setosa (O.Schwartz) J.F.Maxwell, Gard. Bull. Singapore 33: 321. 1980.

##### Type.

Indonesia. South Kalimantan: G. Prarawin, P.W. Korthals s.n. (lectotype, designated here: L [L0729470]!; isolectotype: L [L0729469]!).

**Map 25.**
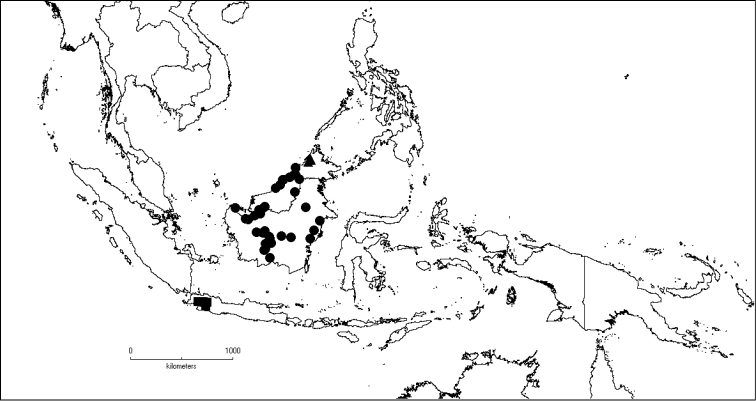
Distribution of *D.rostrata* (●), *D.rubiginosa* (▲) and *D.sagittata* (■).

##### Description.

Climbing up to 8 m in height. Branchlets terete, 2–4 mm in diameter, sparsely brown puberulous and densely covered with 1–2 mm long bristle hairs; nodes swollen, with interpetiolar ridge, thickly covered with bristle hairs; internodes 7–7.5 cm long. Leaves: petioles terete, 8–18 mm long, densely covered with bristle hairs; blades ovate, ovate-elliptic to elliptic, 7.5–14 × 3.8–9 cm, membranous, base emarginate, margin entire, apex acuminate, tip ca. 1 cm long; nervation with 2 pairs of lateral nerves and 1 pair of intramarginal nerves; adaxially hirsute, covered with scattered bristle hairs, abaxially densely covered with bristle hairs in most part, more densely so at midrib and margins. Inflorescences terminal, up to 25 cm long, many-flowered; main axis terete, densely covered with bristle hairs; primary axes up to 15 cm long with 4 nodes, secondary axes 2–4 cm long with 2 or 3 nodes, tertiary axes up to 1 cm long with 1 node; bracts oblong-lanceolate, 15–17 × ca. 5 mm, sparsely stellate puberulous and with dense bristle hairs, thin; bracteoles oblong, 4–7 × 2–3 mm, sparsely stellate puberulous and margin with dense bristle hairs; pedicels densely stellate-furfuraceous and with bristle hairs, 2–3 mm long in central flowers, 1–2 mm long in lateral flowers. Hypanthium campanulate or suburceolate, ca. 4 × 2.5–3 mm, densely covered with brown stellate hairs and bristle hairs; calyx lobes subtriangular or oblong, 2–2.5 mm long, apex obtuse, densely bristly at margin, pinkish to purplish; petal bud conical, 2–3.5 mm long, apex bristly; mature petals obovate or suborbicular, 5–6 × 4–5 mm, not-reflexed, base clawed, apex rounded, glabrous, veined, purple, light purple or pink. Stamens 8, subequal, filaments curved sideways, pale yellow; alternipetalous stamens with 4–5 mm long filaments, anthers lanceolate, sickle-shaped, thecae 5–6 mm long, apex rostrate, purple, pedoconnective ca. 2 mm long, basal crests minute, thin, ca. 0.5 mm long, lateral appendages paired, wavy, filiform, 1–2 mm long, white; oppositipetalous stamens with 4–5 mm long filaments, anthers thick, S-shaped, thecae 4–5 mm long, basal crest minute, ca. 0.5 mm long, lateral appendages paired, filiform, ca.1 mm long, white. Ovary ⅔ of hypanthium in length, apex villous; style curved at end, 10–11 mm long, glabrous, white; stigma minute, capitate; extra-ovarial chambers 8, extending to the middle of the ovary. Fruits subglobose or ovoid, 5–6 × 3–5 mm, densely covered with bristle hairs; calyx lobe remnants persistent, reflexed. Seeds ca. 0.5 mm long.

##### Distribution.

Borneo.

##### Ecology and habitat.

In mixed lowland dipterocarp forest, open areas at margin of forest and in riverine forest at 50–200 m elevation.

##### Vernacular name.

*akar kemunting* (Iban).

##### Notes.

1. *Dissochaetarostrata* can easily be distinguished from other species with a bristly indumentum by its oblong-lanceolate bracts and bracteoles and subtriangular-oblong calyx tube.

2. The type collection (*Korthals s.n.*) from Leiden is only vegetative with mature leaves. The type of *D.hirsuta* (*Motley s.n.*) from Kew consists of a fruiting branch with mature leaves. The appearance of both is similar (shape and indumentum on the leaf blades), therefore we consider them to indicate the same species and we synonymise *D.hirsuta* with *D.rostrata*, an action following Veldkamp et al. (1979).

3. *Dissochaetasetosa* (type *Winkler 1167*), which is also limited to Borneo, has fruits that are similar to *D.hirsuta* with the distinct subtriangular-oblong calyx lobe remnants, hence we also included *D.setosa* in the species concept of *D.rostrata*.

4. Maxwell (1980b) recognised several species with a bristly indumentum as varieties of *D.rostrata* and made the species concept of the latter much wider. Here we consider them to represent distinct species: *D.alstonii*, *D.densiflora*, *D.floccosa*, *D.horrida*, *D.macrosepala*, *D.malayana* and *D.porphyrocarpa*.

##### Specimens examined.

**MALAYSIA. Sarawak**: Parai, 11 Dec 1892, G.D. Haviland 2036 (BM, K); Bintulu, Bukit Urang, 30 m, 7 Dec 1959, F. Brunig S.11981 (K); Kuching, E. Bartlett s.n. (BM); Lubok Antu, River Delok, Nanga Sumpa, 150 m, 27 Feb 1993, Christensen 1244 (K), Lubok Antu, Lanjak Entimau, 14 Mar 1974, P.K. Chai S.33819 (K); Kapit, Ulu Katibas, Sg. Joh, 110 m, 27 Jun 1993, A. Zainudin 4535 (L); *Ibid.*, 150 m, 15 Nov 1997, K.G. Pearce et al. ITTO/BB 0431 (BO); Lawas, 22 May 1955, W.M.A. Brooke 10034 (BM, L); Lundu, Mt. Gading, 100 m, 19 Jul 1963, W.L. Chew 597 (K, L); Marudi, Long Tukan, 13 Mar 1972, Othman, Jugah & Anyie S.31862 (K, L); Miri, Lambir National Park, 8 May 1966, Banyeng ak Nudong S.25084 (BO, K, L), Ulu Sungei Lepoh, 18 Sep 1978, R. George S.40264 (L); Labuan, J. Motley s.n. (K); Niah, Niah River, 4 Apr 1979, P.C. Yii S.40124 (L). **BRUNEI. Belait**: Jalan Merangking-Buau, 10 Aug 1991, N. Nangkat 251 (K, L). **Temburong**: Batu Apoi, Selapon, 30 m, 27 Jan 1994, M.J.E. Coode et al. 7912 (L). **INDONESIA. Central Kalimantan**: Sungai Mentaya, 50 m, 1 Aug 1993, P. Wilkie 93374 (BO, E, K, L); *Ibid.*, Tuke P1 1000 (L); *Ibid.*, Tuke P5 1010 (L); Kapuas, Kayu Mas, 130 m, 24 Apr 1979, P.J.A. Kessler et al. 1461 (BO, L); Sampit River, Kuala Kuayan, 20 m, 1 Aug 1953, A.J.G.H. Kostermans 8045 (BO, L); *Ibid.*, 27 Nov 1982, J.J. Afriastini 427 (BO); *Ibid.*, Permantang, 50 m, 27 Jan 1954, A.H.G. Alston 13375 (BM); *Ibid.*, 4 Apr 1984, C. Hansen 1366 (L); Bukit Raya, Tumbang Samba, 200 m, 27 Nov 1982, J.P. Mogea & W.J.J.O. de Wilde 3716 (BO, L); *Ibid.*, 19 Dec 1982, H.P. Nooteboom 4370 (BO, L); *Ibid.*, Batu Badinding, 23 Dec 1982, J.P. Mogea & W.J.J.O. de Wilde 4376 (BO, L); *Ibid.*, Tumbang Tubus, 150 m, 6 Jan 1983, J.F. Veldkamp 8076 (BO, L). **East Kalimantan**: Sangata, Mentoko River, 300 m, 24 Jan 1979, R. Leighton 433 (L); Sebulu, 10 Aug 1973, K. Kartawinata 1185 (BO, L); *Ibid.*, 27 Dec 1978, G. Murata et al. B-459 (BO, L); Road Kenangan to Gunung Meratus, 400 m, 27 Mar 1995, P.J.A. Kessler et al. 913 (P); Samarinda, Bengalon, 12 Nov 1911, L.M.R. Rutten 535 (U); West Kutai, Long Petah, 450 m, 16 Sep 1925, F.H. Endert 3360 (BO). **South Kalimantan**: Mount Prarawin, P.W. Korthals s.n. (L). **West Kalimantan**: Sintang, 150 m, 11 Apr 1994, U.W. Mahyar et al. 832 (BO, L); *Ibid.*, Sungai Posang, 110 m, 30 Apr 1994, U.W. Mahyar et al. 1229 (BO, L); *Ibid.*, Tegua Tibun, 75 m, 16 Oct 2000, Albertus & K. Sidiyasa 2236 (L); Lebang Hara, 150 m, 1 Jan 1925, J. Winkler 1167 (BO, L); Sanggau, Noyan, Ngira, 20 Feb 1994, De Jong 749 (BO, L); Katingan-Seruyan, 213 m, 26 Jul 2011, R. Susanti et al. 264 (BO).

#### 47. Dissochaeta rubiginosa

Taxon classificationPlantaeMyrtalesMelastomataceae

Stapf, J. Linn. Soc., Bot. 42: 79. 1914.

Map 25

##### Type.

Malaysia. Sabah: Ranau, Mount Kinabalu, Gurulau Spur, 5500 ft elev., Feb 1910, L.S. Gibbs 3977 (holotype: K [K000859491]!).

##### Description.

Climber. Branchlets terete, 3–5 mm in diameter, covered with densely stellate-furfuraceous hairs; nodes swollen, interpetiolar ridge raised; internodes 3.5–10 cm long. Leaves: petiole terete, 10–15 mm long, densely stellate-furfuraceous; blade elliptic-oblong or oblong, 7–11 × 2–4.8 cm, membranous, base rounded or cuneate, margin entire, apex acuminate, tip 0.5–1 cm long; nervation with 1 pair of lateral nerves and 1 pair of intramarginal nerves; adaxially glabrous, dark glossy green, abaxially brown, stellate-furfuraceous. Inflorescences terminal and in the upper leaf axils, cymous, with many flowers, 17–25 cm long; main axis angular densely stellate-furfuraceous; primary axes up to 23 cm long with 5–7 nodes, secondary axes 2–9 cm long with 2–5 nodes, tertiary axes 0.7–1.8 cm long with 1 or 2 nodes; bracts and bracteoles minute, less than 2 mm long, densely stellate-furfuraceous, caducous; pedicel densely stellate-furfuraceous, 2–3 mm long in central flowers, 1–2 mm long in lateral flowers. Hypanthium urceolate, 3–4 × 1–1.5 mm, densely stellate-furfuraceous; calyx lobes truncate with 4 triangular tips or occasionally slightly free triangular lobes, ca. 1 mm long; petal bud conical, 3–5 mm long, glabrous; mature petals oblong, 5–6 × ca. 2 mm, glabrous, red, base clawed, apex acute. Stamens 4, equal, filaments straight; alternipetalous stamens with 4–5 mm long filaments, anthers oblong or lanceolate, thecae ± straight, 4–5 mm long, pedoconnective short or slightly undeveloped, ca. 0.5 mm long, basal crest triangular, up to 1 mm long, lateral appendages paired, filiform, of unequal length, ca. 2 mm long at one side, 1–1.5 mm long on the other side. Ovary ¾ of hypanthium in length, apex villous; style glabrous, 7–10 mm long, straight but slightly curved apically; stigma capitate; extra-ovarial chambers 4, alternipetalous, shallow, reaching to ca. ⅓ of ovary. Fruits ovoid-urceolate, 5–6 × 3–3.5 mm, glabrescent, calyx remnant persistent up to 2 mm long. Seeds ca. 0.5 mm long.

##### Distribution.

Borneo (Sabah).

##### Ecology and habitat.

Montane forest at 900–1670 m elevation.

##### Note.

*Dissochaetarubiginosa* resembles *D.angiensis* in indumentum and number of stamens, but differs by having more distinct triangular calyx lobes. The erect persistent calyx on the fruits is also different from *D.angiensis*.

Stapf & Green (1914) mention the collections Wallich 4052 from Penang and Helfer 2286 from Myanmar and refer them to this species. However, both specimens have a shorter (<3 mm long) campanulate hypanthium rather than the urceolate and long hypanthium (3–4 mm long) of *D.rubiginosa* and both are identified as *D.biligulata* in this revision. The flower petals were recorded as red, a colour uncommon in the genus (Stapf & Green 1914).

##### Specimen examined.

**MALAYSIA. Sabah**: Ranau, Mount Kinabalu,Gurulau Spur, 1670 m, L.S. Gibbs 3977 (K); *Ibid.*, Marai Parai spur, 22 Nov 1915, M.S. Clemens 10941 (PNH); *Ibid.*, Dallas, 900 m, J. Clemens & M.S. Clemens 26058A (K, L); *Ibid.*, Sosopodon, 1500 m, Adam Gintus SAN 56381 (K, L); *Ibid.*, S. Kokawa & M. Hotta 5190 (L); Tambunan, Mt. Alab, S. Kokawa & M. Hotta 2089 (L).

#### 48. Dissochaeta sagittata

Taxon classificationPlantaeMyrtalesMelastomataceae

Blume, Flora 14: 500. 1831.

Map 25

Dissochaeta intermedia Blume var. sagittata (Blume) J.F.Maxwell, Gard. Bull. Singapore 33: 315. 1980.

##### Type.

Indonesia. Java, Bantam, C.L. Blume 11 (lectotype, designated here: L [L0537226]!; isolectotype: L [L0537228]!).

##### Description.

Climbing up to 7 m in height. Branchlets terete, 3–4 mm in diameter, densely stellate-furfuraceous; nodes swollen, with interpetiolar ridge, covered by stellate-furfuraceous hairs; internodes 6–7 cm long. Leaves: petioles flattened, 10–15 mm long, densely stellate-furfuraceous; blades oblong, 8.5–13 × 3–4.5 cm, membranous, base rounded, margin entire, apex acuminate, tip ca. 1 cm long; nervation with 1 pair of lateral nerves and 1 pair of intramarginal nerves; adaxially glabrous, abaxially densely brown stellate-furfuraceous. Inflorescences terminal and in the upper leaf axils, up to 30 cm long, many-flowered; main axis quadrangular, densely stellate-furfuraceous; primary axes up to 26 cm long with 5 or 6 nodes, secondary axes 3–6 cm long with 2 or 3 nodes, tertiary axes up to 0.8–2 cm long with 1 or 2 nodes; bracts oblong to lanceolate, 9–10 × 2–3 mm, stellate-furfuraceous; bracteoles lanceolate, 4–6 mm long, stellate-furfuraceous, distinctly nerved; pedicels densely stellate-furfuraceous, 4–7 mm long in central flowers, 2–5 mm long in lateral flowers. Hypanthium campanulate, 4–7 × 3–5 mm, densely stellate-furfuraceous; calyx lobes truncate with 4 more or less triangular tips, ca. 1 mm long, stellate-furfuraceous; petal buds conical, 2–8 mm long; mature petals oblong, 9–11 × ca. 4 mm, base clawed, apex obtuse, glabrous, red to pinkish-red. Stamens 8, unequal, filaments straight; alternipetalous stamens with ca. 6 mm long filaments, anthers lanceolate, thecae 6–7 mm long, straight, pedoconnective 1–1.5 mm long, basal crest triangular, hastate to sagittate, ca. 1.5 mm long, lateral appendages paired, filiform, 3–4 mm long, sometimes unequal in length; oppositipetalous stamens with 4–6 mm long filaments, anthers ovate, thecae 3–5 mm, straight or falcate, basal crest spuriform or ligulate, 0.5–1 mm long, lateral appendages absent. Ovary ⅔ of hypanthium in length, apex pubescent; style 13–15 mm long, curved at apex, glabrous; stigma minute, capitate; extra-ovarial chambers 8, extending to below the middle of the ovary. Fruits urceolate, 8–10 × 5–6 mm, sparsely hairy to glabrescent; calyx lobes persistent, erect. Seeds ca. 0.5 mm long.

##### Distribution.

Java (West).

##### Ecology and habitat.

Secondary forest at 700–1400 m elevation.

##### Specimens examined.

**INDONESIA. Banten**: C.L. Blume 11 (L). **West Java**: Bogor, Mt. Karang Gantungan, C.A. Backer 6272 (BO); *Ibid.*, Cisangku, C.A. Backer 10549 (BO); Cianjur, Sukanegara, E.R. Hellendoorn 8 (BO); Mt. Gede, H. Raap 695A (L); Bandung, Nanggerang, C.A. Backer 9097 (BO).

#### 49. Dissochaeta sarawakensis

Taxon classificationPlantaeMyrtalesMelastomataceae

(M.P.Nayar) J.F.Maxwell, Gard. Bull. Singapore 33: 321. 1980.

Map 26

Neodissochaeta sarawakensis M.P.Nayar, Bull. Bot. Surv. India 11: 195. 1969.

##### Type.

Malaysia. Sarawak: Pengkulu Ampat, G.D. Haviland 69 (holotype: K [K000859625]!).

**Map 26.**
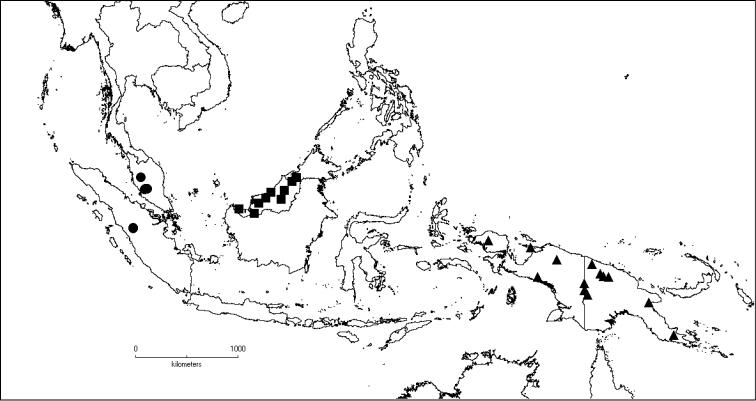
Distribution of *D.sarawakensis* (■), *D.schumannii* (▲) and *D.spectabilis* (●).

##### Description.

Climbing up to 9 m in height; branchlets terete, 3–4 mm in diameter, covered with thin, 1–2 mm long bristle hairs; nodes swollen, with distinct crest-like interpetiolar ridges, margin bristly, up to 4 mm wide; internodes 2–5 cm long. Leaves: petioles terete, 4–7 mm long, sparsely stellate-puberulous and with scattered bristles at dorsal groove; blades ovate to elliptic, 5–6 × 2.3–3.8 cm, subcoriaceous, base rounded to subcordate, margin entire, apex acuminate, tip ca. 0.5 cm long; nervation with 1 pair of lateral nerves and 1 pair of intramarginal nerves; surfaces glabrous, sometimes abaxially with pair of glandular patches at base. Inflorescences terminal, up to 10 cm long, many-flowered; main axis stellate-furfuraceous; primary axes up to 4 cm long with 2 or 3 nodes, secondary axes 1–2 cm long with 1 or 2 nodes, tertiary axes up to 0.8 cm long with 1 node; bracts linear, 2–3 mm long, glabrous; bracteoles subulate, ca. 1 mm long, stellate-furfuraceous; pedicels furfuraceous, 3–4 mm long in central flowers, 1–2 mm long in lateral flowers. Hypanthium campanulate, 2–3 × ca. 2 mm, glabrous; calyx lobes truncate, ca. 0.5 mm long, with 4 small minute points; petal buds conical, 2–3 mm long, apex narrowly acuminate, glabrous; mature petals ovate, ca. 5 × 3.5 mm, glabrous, reflexed, base clawed, apex acute, white or pale pink. Stamens 8, equal, filaments curved sideways; alternipetalous stamens with 3–4 mm long filaments, anthers oblong, curved, sickle-shaped, thecae 3–4 mm long, yellow, pedoconnective short, ca. 0.5 mm long, basal crest ligular, bi- or trifid, ca. 2 mm long, lateral appendages paired, filiform, 4–5 mm long; oppositipetalous stamens with 3–4 mm long filaments, anthers S-shaped, thecae 3–3.5 mm long, yellow, basal crest ligular, ca. 1.5 mm long, lateral appendages paired, filiform, ca. 2 mm long. Ovary ⅔ of hypanthium in length, apex glabrous; style glabrous, ca. 9 mm long, reddish; stigma minute; extra-ovarial chambers absent or not developed. Fruits globose, 2–3 × ca. 2 mm, glabrous; calyx lobe remnants persistent. Seeds ca. 0.5 mm long.

##### Distribution.

Borneo (Brunei and Sarawak).

##### Ecology and habitat.

Heath forest or mixed dipterocarp forest at 240–1000 m elevation.

##### Vernacular names.

*akar kemunting* (Iban); *akar kitum* (Kenyah).

##### Note.

Vegetatively similar to *D.stipularis* in its distinct wide, crest-like interpetiolar ridges and glabrous leaf blades. The difference is that *D.sarawakensis* has fertile alternipetalous stamens, which are equal to the oppositipetalous ones, while in *D.stipularis* the alternipetalous stamens are staminodes, reduced and smaller than the oppositipetalous ones.

##### Specimens examined.

**MALAYSIA. Sarawak**: Pengkulu Ampat, G.D. Haviland 69 (K); Baram, Gunung Mulu, 365 m, 4 Jul 1961, J.A.R. Anderson & H. Keng K7 (BO, K, L); *Ibid.*, Ulu Tinjar, Dulit Range, 300 m, 9 Aug 1974, P.K. Chai S.34713 (K, L); Balingian, Bawan, Begrih, 10 m, 20 Oct 1963, P.K. Chai S.19481 (K); Bintulu, Segan FR., 244 m, 18 Aug 1968, Ilias Paie S.27040 (BO, K, L); Kuching, Mount Matang, 396 m, 27 Mar 1929, J. Clemens & M.S. Clemens 20929 (BO, K); Kapit, Batang Rejang, Batu Laga, 1000 m, 12 Sep 1984, Abang Mochtar S.48265 (K, L); Sri Aman, Gunong Silantek, 530 m, 27 Aug 1980, Ilias Paie S.42599 (K, L). **BRUNEI. Temburong**: Amo, Bukit Tudal, 840-1160 m, 6 Oct 1994, P.C. Bygrave et al. 29 (K, L).

#### 50. Dissochaeta schumannii

Taxon classificationPlantaeMyrtalesMelastomataceae

Cogn. in K.Schum. & Hollrung, Fl. Kais. Wilh. -Land: 88. 1889.

Map 26

Neodissochaeta lamiana Bakh.*f.*, Contr. Melastom.: 142. 1943. Type: Indonesia. Papua: Prauwenbivak, Mamberamo River, 140 m elev., 29 Aug 1920, H.J. Lam 935 (holotype: L [L0537230]!; isotypes: BO [BO1747956]!, K [K000859605, K000859606]!, U [U0004013]!).
Neodissochaeta schumannii (Cogn.) M.P.Nayar, Kew Bull. 20: 160. 1966.

##### Type.

Papua New Guinea. Kaiser Wilhelmsland, Augusta Fluss, 1887, U.M. Hollrung 656 (lectotype, designated here: BO [BO1747958]!; isolectotypes: BR [BR5187904, image seen]!, K [K000859604]!, L [L0537229]!).

##### Description.

Climbing up to 4.5 m in height; branchlets terete, 3–4 mm in diameter, sparsely to densely covered with stellate hairs; nodes swollen, with interpetiolar line; internodes 7.5–8.5 cm long. Leaves: petioles terete, 8–10 mm long, stellate-furfuraceous; blades ovate to elliptic, 8–16 × 4–6.5 cm, membranous, base emarginate, margin entire, apex acuminate, tip up to 1 cm long; nervation with 1 pair of lateral nerves and 1 pair of intramarginal nerves; adaxially glabrous, abaxially with dense, stellate, brown or grey tomentose hairs. Inflorescences terminal, up to 25 cm long, many-flowered; main axis densely stellate-furfuraceous; primary axes 12–20 cm long with 4 or 5 nodes, secondary axes 3–6 cm long with 2 or 3 nodes, tertiary axes 1–2 cm long with 1 node; bracts lanceolate, 3–4 × ca. 2 mm, stellate-furfuraceous; bracteoles linear, 2–4 mm long, stellate-furfuraceous, caducous; pedicels stellate-tomentose, 3–4 mm long in central flowers, 1–2 mm long in lateral flowers. Hypanthium campanulate, 3–4 × ca. 2 mm, densely stellate-tomentose, sometimes with a few scattered, ca. 0.5 mm long glandular bristles; calyx lobes slightly triangular, ca. 4 × 2.5 mm, apex acute, densely stellate-furfuraceous, sometimes with a few glandular bristles, caducous; petal buds conical, ca. 4 mm long, glabrous; mature petals obovate, 4–5 × ca. 3 mm, base clawed, apex obtuse, glabrous, pink. Stamens 4, equal, alternipetalous, filaments curved sideways, 3–4 mm long, anthers oblong, curved, sickle-shaped, thecae 3–4 mm long, yellow, pedoconnective ca. 1 mm long, basal crest triangular with irregular edge, ca. 0.5 mm long, lateral appendages paired, filiform, 1–1.5 mm long. Ovary ¾ of hypanthium in length, apex villous; style slender ca. 5 mm long; stigma minute; extra-ovarial chambers 4, alternipetalous, shallow, ca. ¼ of ovary. Fruits globose or ovoid, 5–6 × 3–4 mm, glabrous or stellate puberulous, slightly 8-lined; calyx lobe remnants, caducous. Seeds ca. 0.5 mm long.

##### Distribution.

New Guinea.

##### Ecology and habitat.

Lowland primary forest, in open places at 90–600 m elevation.

##### Vernacular names.

*nangumush* (Waskuk); *soiya* (Wagu).

##### Note.

Different from other tetrandrous species by its distinct triangular calyx lobes which are caducous in fruit. The leaf blade underneath is also typical by the greyish tomentose indumentum, which differs from other New Guinean species (e.g. *D.angiensis* and *D.brassii*).

##### Specimens examined.

**INDONESIA. Papua**: Camp Prauwen, Mamberamo River, 140 m, 29 Aug 1920, H.J. Lam 935 (BO, K, L); Mimika, Kuala Kencana, 65 m, 25 Jan 1998, R.J. Johns, S. Puradyatmika & A. Sadili 8887 (BO, K, L); *Ibid.*, 10 m, 10 Apr 2000, T.M.A. Utteridge et al. 312 (BO, K, L); *Ibid.*, 20 Nov 2000, E.J. Lucas et al. 24 (BO, K, L); Ingembit to Konomptan, 12 Jun 1967, S. Reksodihardjo 481 (BO, K, L). **West Papua**: Ayawasi, 450 m, 18 Mar 1996, C.E. Ridsdale 2327 (L). **PAPUA NEW GUINEA. East Sepik**: Ambunti, Near Wagu, 90 m, 1 Jun 1966, R.D. Hoogland & L.A. Craven 10180 (BO, K, L); *Ibid.*, 8 Jul 1966, R.D. Hoogland & L.A. Craven 10514 (BO, K, L); *Ibid.*, Mount Garamambu, 21 Aug 1949, J.S. Womersley NGF 3751 (BO, K, L). **Milne Bay**: Biniguni Airstrip, Mt. Suckling, 365 m, 5 Jul 1972, R. Pullen 8424 (BO, K, L). **Morobe**: Lae, Gabensis, 600 m, 25 Apr 1990, S. Simaga 1836 (L). **Sepik**: Kaiser Wilhelmsland, Augusta River, 1887, U.M. Hollrung 656 (BO, K, L). **Western District**: Kiunga, 21 m, 5 Aug 1971, H. Streimann & P. Katik NGF 46798 (BO, K, L); *Ibid.*, Ingembit, 146 m, 13 Jun 1967, E.E. Henty, C.E. Ridsdale & M. Galore NGF 33015 (L); Bigel, 24 May 2002, P. Piskaut UPNG 20172 (K, L). **West Sepik**: Carpentaria, Ekwaii River, 500 m, Dec 1977, W.S. Hoover 473 (L).

#### 51. Dissochaeta spectabilis

Taxon classificationPlantaeMyrtalesMelastomataceae

J.F.Maxwell, Gard. Bull. Singapore 33: 321. 1980.

Fig. 26 , Map 26

Dissochaeta marumioides Furtado, Gard. Bull. Singapore 20: 111, fig. 1. 1963., *nom. illeg*., non Cogn. 1891.

##### Type.

Malaysia. Pahang: Cameron Highlands, Tanah Rata, 1300–1500 m elev., A. Johnston & M. Johnston 86 (holotype: SING!).

**Figure 26.**
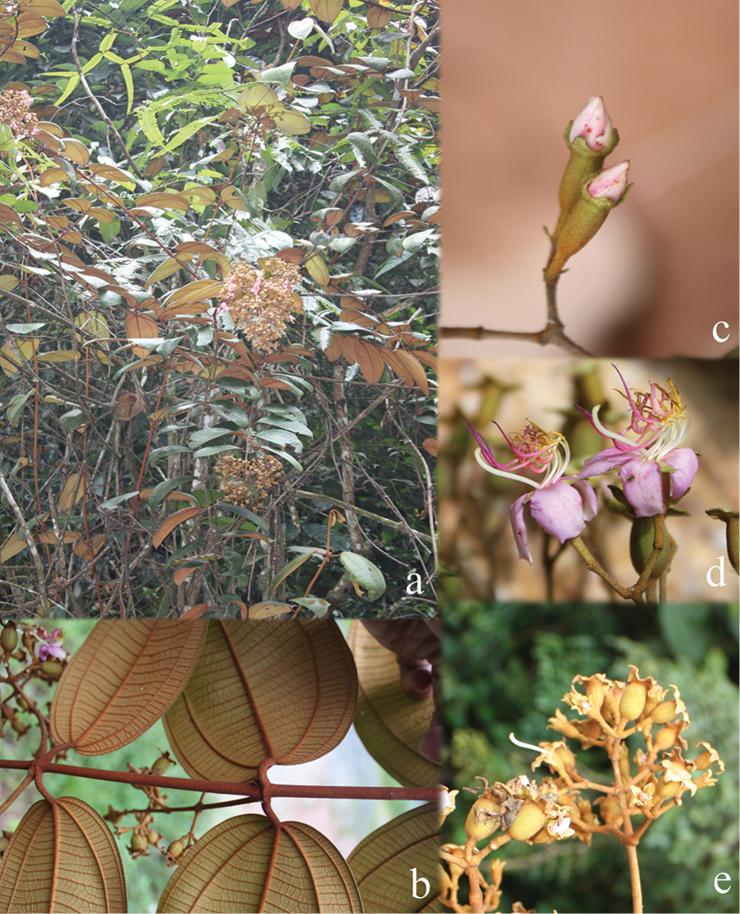
*Dissochaetaspectabilis*. **a** habit **b** branchlet **c** hypanthium **d** flowers **e** fruits. Photographs by A. Kartonegoro; vouchers: Kartonegoro 1100 (BO, L).

##### Description.

Climbing up to 5 m in height; branchlets terete, 3–5 mm in diameter, densely stellate-furfuraceous; nodes swollen, with interpetiolar ridge; internodes 4–5.5 cm long. Leaves: petioles flattened, 10–15 mm long, densely stellate-furfuraceous; blades ovate, ovate-elliptic or elliptic, 9–13.5 × 4.3–6 cm, membranous, base rounded to subcordate, margin entire, apex acuminate, tip 1.5–2 cm long; nervation with 1 pair of lateral nerves and 1 pair of intramarginal nerves; adaxially glabrous, abaxially densely brown stellate-tomentose. Inflorescences terminal, up to 42 cm long, many-flowered; main axis angular, densely stellate-tomentose; primary axes up to 38 cm long with 4–7 nodes, secondary axes 1.2–7.5 cm long with 1–4 nodes, tertiary axes 0.7–2.5 cm long with 1 or 2 nodes, quarternary axes when developed 0.3–0.8 cm long with 1 node; bracts linear, 8–10 mm long, densely stellate-tomentose, caducous; bracteoles linear, 5–6 mm long, densely stellate-tomentose; pedicels densely stellate-tomentose, 2–4 mm in central flowers, 1-2 mm long or subsessile in lateral flowers. Hypanthium campanulate, 7–8 × 3–4 mm, slightly 8-ridged, densely stellate-tomentose; calyx lobes triangular, 3–4 mm long, erect, apex acute, densely stellate-tomentose; petal buds conical, 8–10 × 3–4 mm, glabrous; mature petals obovate or ovate, ca. 10 × 8 mm, glabrous, reflexed, base clawed, apex obtuse, pink. Stamens 8, unequal, filaments flattened, white, base pinkish, apex yellowish, curved sideways; alternipetalous stamens with 8–10 mm long filaments, anthers lanceolate, sickle-shaped, thecae 12–14 mm long, pink, pedoconnective 3–5 mm long, basal crests distinctly triangular, ca. 1 mm long, yellow, narrow with acute apex, lateral appendages paired, filiform, 2–3 mm long, yellow; oppositipetalous stamens with 8–10 mm long filaments, anthers S-shaped, thecae 7–10 mm long, thick, cream to bright pink, basal crest spur-like, ca. 1 mm long, bifid, erect, lateral appendages paired, filiform, 2–3 mm long, brownish. Ovary ¾ of hypanthium in length, apex pubescent; style glabrous, 10–12 mm long, white, apex curved; stigma capitate, minute; extra-ovarial chambers 8, extending almost to the base of the ovary. Fruits ovoid or urceolate, 13–15 × 8–20 mm, stellate-puberulous or nearly stellate-tomentose, with calyx lobes remnant persistent to sometimes caducous. Seeds ca. 0.75 mm long.

##### Distribution.

Peninsular Malaysia (Pahang and Selangor) and Sumatra (West).

##### Ecology and habitat.

Lower to upper montane forest, in open places at 520–1740 m elevation.

##### Note.

*Dissochaetaspectabilis* can be distinguished from other species by its dense tomentose indumentum and erect, triangular, calyx lobes, which are subpersistent in fruit. The calyx lobes are remniscent of the genus *Macrolenes*, therefore Furtado (1963) used this as an epithet (*marumioides*, like *Marumia*, a synonym of *Macrolenes*). Since this epithet was already used by Cogniaux (1891) for a species from Sumatra, *D.marumioides* Furtado became a later illegitimate homonym. Therefore, Maxwell (1980b) proposed the new name of *D.spectabilis*.

##### Specimens examined.

**MALAYSIA. Pahang**: Bentong, 1500 m, 17 May 1987, R.D. Worthington 12804 (L); Gunung Bunga Buah, 1432 m, 4 Dec 2006, M.K. Hisham et al. FRI 52081 (K, L); Cameron Highlands, Robinson Falls, 1430 m, 30 Aug 1956, H.M. Burkill HMB 750 (K, L); *Ibid.*, Tanah Rata, 1300-1500 m, A. Johnston & M. Johnston 86 (SING); *Ibid.*, 1550 m, 14 Apr 1978, J.F. Maxwell 78-133 (L). **Selangor**: Genting Highlands, Gunong Ulu Kali, 1670 m, 17 Dec 1977, B.C. Stone 13535 (K); *Ibid.*, 1700 m, 3 Jun 1978, J.F. Maxwell 78-323 (L, P); *Ibid.*, 5 Aug 1979, B.C. Stone 14131 (L); *Ibid.*, 1700 m, 11 Jun 1977, W.H. Siew 95 (L); *Ibid.*, 1520 m, 2 Jul 1977, W.H. Siew 210 (L). **INDONESIA. West Sumatra**: Lima Puluh Kota, Aer Putih, 520 m, 22 Feb 1954, A.H.G. Alston 13816 (BM, BO); *Ibid.*, Harau Valley, Sarasah Bonta, 500-580 m, 17 Apr 1999, Seren 65 (ANDA); *Ibid.*, Kelok Sembilan, 800 m, 20 May 2001, Zul et al. 43 (ANDA); *Ibid.*, 13 Sep 2017, A. Kartonegoro 1100 (BO, L).

#### 52. Dissochaeta stipularis

Taxon classificationPlantaeMyrtalesMelastomataceae

(Blume) Backer ex Clausing in S.S.Renner et al., Fl. Thailand 7(3): 431. 2001.

Map 27

Melastoma stipulare Blume, Bijdr. Fl. Ned. Ind 17: 1073. 1826.
Aplectrum stipulare (Blume) Blume, Flora 14: 503. 1831.
Anplectrum stipulare (Blume) Triana, Trans. Linn. Soc. London 28: 84. 1872.
Anplectrum annulatum Triana, Trans. Linn. Soc. London 28: 84. 1872. Type: Malaysia. Peninsular Malaysia, Penang, N. Wallich 4056 (holotype: K *n.v.*).
Diplectria stipularis (Blume) Kuntze, Revis. Gen. Pl. 1: 246. 1891.
Diplectria annulata (Triana) Kuntze, Revis. Gen. Pl. 1: 246. 1891.
Anplectrum lepidoto-setosum King, J. Asiat. Soc. Bengal, Pt. 2, Nat. Hist. 69(2): 56. 1900. Type: Malaysia. Peninsular Malaysia, Perak, B. Scortechini 2106 (lectotype, designated here: K [K000859525]!; isolectotypes: CAL *n.v.*, SING *n.v.*).
Anplectrum crassinodum Merr., Univ. Calif. Publ. Bot. 15: 223. 1929, non. Merr. (1939). Type: Malaysia. Sabah: Elphinstone Province, Tawao, A.D.E. Elmer 21291 (lectotype, designated here: BO [BO1865944]!; isolectotypes: K [K000859624]!, L [L0537307]!, SING *n.v.*).
Backeria stipularis (Blume) Bakh.*f.*, Contr. Melastom.: 132. 1943.
Diplectria annulata (Triana) Kuntze var. seticarpa Furtado, Gard. Bull. Singapore 20: 107. 1963. Type: Malaysia. Pahang: Bentong, Sabai Estate, 400 ft. elev., 27 Jan 1958. M. Shah 176 (holotype: SING *n.v.*; isotypes: BO [BO1760865]!, K [K000859524]!, L [L0537303]!).
Backeria annulata (Triana) Raizada, Indian Forester 94: 435. 1968.

##### Type.

Indonesia. West Java, G. Seribu, C.L. Blume 857 (lectotype, designated by Veldkamp et al. 1979, pg. 424: L [L0537306]!; isolectotypes: L [L0537304, L0537305]!, P [P02274923, image seen]!).

**Map 27.**
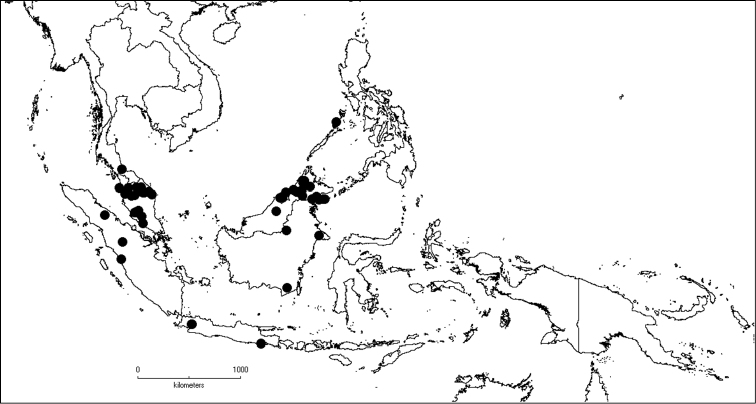
Distribution of *D.stipularis* (●).

##### Description.

Climbing up to 8 m in height; branchlets terete, 3–4 mm in diameter, with minute stellate hairs and scattered, ca. 2 mm long, reddish-brown bristles; nodes swollen, with raised annular crest-like interpetiolar ridge, up to 5 mm wide, densely covered with stellate hairs and scattered bristles; internodes 4.7–10 cm long. Leaves: petioles terete, 5–8 mm long, densely with stellate hairs and bristles; blades elliptic to elliptic oblong, 11–13.5 × 3.7–4.8 cm, membranous or subcoriaceous, base rounded to subcordate, margin entire, apex acute or acuminate, tip 1–2 cm long; nervation with 1 pair of lateral nerves and 1 pair of intramarginal nerves; adaxially glabrous, abaxially glabrous, except sparse stellate hairs and bristles at base of midrib. Inflorescences terminal, up to 12 cm long, many-flowered; main axis densely covered with stellate hairs and bristles, bright red; primary axes 3–5 cm long with 2–4 nodes, secondary axes up to 4 cm long with 1 or 2 nodes, tertiary axes up to 1.5 cm long with 1 node; bracts linear, 5–8 mm long, densely stellate-furfuraceous and bristly; bracteoles linear, 3–4 mm long, densely stellate-furfuraceous and bristly; pedicels densely stellate-furfuraceous and with bristle hairs, 3–5 mm long in central flowers, 1–3 mm long in lateral flowers, bright red. Hypanthium cyathiform-tubular, 3–3.5 × 2–2.5 mm, glabrous or stellately furfuraceous and covered with bristle hairs; calyx lobes truncate with 4 small points, ca. 0.5 mm long, densely bristly; petal buds conical, 1–2 mm long; mature petals ovate, 2–3 × ca. 2 mm, base clawed, apex acute, white or white purplish. Stamens 8, unequal, filaments straight; alternipetalous stamens staminodial with 2–2.5 mm long filaments, anthers rudimentary, thecae 2–3 mm long, slender, terete, white, basal crest triangular or ligular, thin, ca. 1 mm long, lateral appendages paired, linear, flat, filiform, ca. 1 mm long; oppositipetalous stamens with 2–3 mm long filaments, anthers thick, curved, hooked, thecae 2–3 mm long, apex obtuse, yellow, basal crest shortly triangular, ca. 0.3 mm long, lateral appendages absent. Ovary ⅔ of hypanthium in length, apex glabrous; style 4–6 mm long, curved at the end, slender, glabrous, pink; stigma minute; extra-ovarial chambers 4, oppositipetalous, shallow reaching only upper ⅓ of the ovary. Fruits subglobose, 4–5 × 3–4 mm, glabrous or bristly; calyx lobe remnants persistent. Seeds ca. 0.5 mm long.

##### Distribution.

Thailand (Southern Peninsula), Peninsular Malaysia, Sumatra, Java, Borneo and Philippines (Palawan).

##### Ecology and habitat.

Secondary forest or along riverbanks at 75–250 m elevation.

##### Vernacular names.

Peninsular Malaysia: *kayu matahari* (Pahang); *akar lekumbang* (Selangor); *sesendok* (Perak). Borneo: *kudok-kudok* (Brunei).

##### Specimens examined.

**THAILAND. Songkhla**: Hat Yai, Ko Hong, 75 m, 23 Jan 1986, J.F. Maxwell 86-45 (L). **MALAYSIA. Kelantan**: Chaning, 2 Feb 1917, H.N. Ridley s.n. (K); Gunong Stong, 244 m, 15 Aug 1969, T.C. Whitmore FRI 12509 (K, L); Sungai Terang, S. Lebir, 8 Jul 1935, M.R. Henderson SFN 29646 (K); *Ibid.*, Ulu Lebir, Anon. FRI 17707 (K, L); Kuala Mersing, Sungai Brok, 183 m, 13 Jun 1967, F.S.P. Ng FRI 5423 (L). **Malacca**: Ayer Panas, Nov 1894, H.N. Ridley 1574 (BM). **Negeri Sembilan**: Jelebu, Serting Forest Reserve, 200 m, 1 Oct 1996, E.C. Gardette 2279 (L). **Pahang**: Bentong, Sabai Estate, 122 m, 27 Jan 1958, M. Shah 176 (BO, K, L). **Penang**: Government Hill, Oct 1886, C. Curtis 1078 (K). **Perak**: B. Scortechini 2106 (K); Grik, 16 Nov 1966, Ismail KEP 95044 (K, L); Gunong Bubu, 19 Jun 1969, P. Selvaraj FRI 11163 (L). **Selangor**: Kepong, 1 Sep 1927, Pawanche & Awang Lela 13652 (K); Klang Gates, 1 Jan 1921, H.N. Ridley s.n. (K); Ulu Gombak, 180 m, 26 Aug 1928, P.F. Strugnell 13618 (K). **Terengganu**: Dungun, Jerangau Road, 16 Nov 1954, J. Sinclair & Kiah SFN 40491 (BM, K, L); Ulu Brang, 106 m, Jul 1937, L. Moysey & Kiah SFN 33853 (K); Tasik Kenyir, Simpan Tembat, 217 m, 19 Nov 2008, M. K. Hisham FRI 59338 (L). **Sabah**: Beaufort, Beaufort Hill, 29 m, 12 Mar 1962, G. Mikil SAN 28134 (L); Kalabakan, Benaword, 24 Apr 1980, Fidilis & Sumbing SAN 91826 (L); Keningau, Sepulut, 10 Jul 1974, Fidilis & Sumbing SAN 103629 (K, L); Nabawan, Rashna Road, 21 Aug 1976, Dewol SAN 83878 (L); Witti Range, Shang Lian, 23 Aug 1985, Sumbing SAN 110178 (L); Ulu Sungai Tinagalan, 15 Nov 1985, Asik Mantor SAN 113060 (L); Ranau, Trus Madi, Mamut Copper Mine, 1000 m, 9 Jul 1984, J.H. Beaman et al. 10653 (L); *Ibid.*, Mount Kinabalu, 550 m, 8 Jun 1961, W.L. Chew, E.J.H. Corner & A. Stainton RSNB 528 (L); *Ibid.*, Hot Spring, 762 m, 23 Sep 1964, G. Mikil SAN 41905 (L); Sandakan, Telupid, Tangkulap, 28 May 1988, Dewol & Maidil SAN 124683 (L); Semporna, Mount Pock, 91 m, 24 Nov 1965, N. Gansau SAN 54477 (L); Tawau, 1923, A.D.E. Elmer 21291 (BO, K, L); *Ibid.*, Apas Road, 30 m, 24 Jun 1959, W. Meijer SAN 19243 (L, PNH); *Ibid.*, Tawau River, 300 m, 11 Nov 1968, S. Kokawa & M. Hotta 892 (L); *Ibid.*, Brumas, 8 Feb 1977, Fidilis & Awang SAN 85351 (L); Tenom, Tomani, Kambaliangan Hill, 122 m, 10 Oct 1966, A. Talip SAN 50522 (L). **Sarawak**: Baram, Mount Dulit, Ulu Tinjar, 300 m, 23 Aug 1932, P.W. Richards 1401 (L). **BRUNEI. Belait**: Merangking, 17 Nov 1994, N. Nangkat et al. BRUN 15570 (K); *Ibid.*, 40 m, 12 Apr 1995, S. Ismail et al. BRUN 16601 (L); Muara, Bukit Tempayan, 19 May 1994, Joffre et al. BRUN 15465 (K). **Tutong**: Ulu Tutong, Kampong Litad, 29 Jun 1993, N. Nangkat et al. BRUN 15229 (K, L); Tasik Merimbun, 27 Feb 1996, K. Ogata et al. Og-B94 (L); *Ibid.*, 20 m, 15 Sep 2000, E. Suzuki K.13181 (L); Sungei Tutong, 20 m, 5 Nov 1991, D.A. Simpson & M. Marsh 2600 (L). **INDONESIA. North Sumatra**: Pematang Siantar, 450 m, 22 Jul 1937, J.A. Lörzing 17296 (BO, L). **Riau**: Kampar, Tambun, Bukit Suligi, 400-550 m, 29 Apr 1999, D. Arbain & R. Tamin 12 AS (ANDA). **West Sumatra**: Padang, Limau Manis, 400 m, 14 Dec 1991, Nurainas 071 (ANDA). **Banten**: Gunung Seribu, C.L. Blume s.n. (L, P). **East Java**: Malang, Tirtoyudo, Pujiharjo, Tumpak Kembang, Oct 2002, S. Riswan, J.J. Afriastini & Nurdin ML051 (BO, L). **East Kalimantan**: Sangkulirang, Pelawan Besar, Gunung Toda, 25 m, 11 May 1937, Aet 294 (BO, L); Long Sungai Barang, 750 m, 16 Oct 1991, J. van Valkenburg 1041 (L). **South Kalimantan**: Tanah Laut, Kintap, 200 m, 17 Apr 1985, A.J.M. Leeuwenberg & Rudjiman 13432 (L). **PHILIPPINES. Palawan**: Pagdanan Range, San Vicente, 100 m, 23 Apr 1984, A.C. Podzorski SMHI 944 (K, L).

#### 53. Dissochaeta vacillans

Taxon classificationPlantaeMyrtalesMelastomataceae

(Blume) Blume, Flora 14: 495. 1831.

Fig. 27 , Map 24

Melastoma vacillans Blume, Bijdr. Fl. Ned. Ind. 17: 1074. 1826.
Dissochaeta fusca Blume, Flora 14: 497. 1831. Type: Indonesia. Java, C.L. Blume 1791 (lectotype, designated by Kartonegoro and Veldkamp 2010, pg. 143: L [L0729468]!; isolectotypes: K [K000859621]!, L [L0537244]!, P [P05283572, image seen]!,).
Dissochaeta fusca Blume var. ferruginea Blume, Flora 14: 497. 1831. Type: Indonesia. Java, Bantam, J.C. van Hasselt s.n. (lectotype, designated by Kartonegoro and Veldkamp 2010, pg. 143: L [L0537248]!; isolectotypes: L [L0537239, L0537247]!).
Dissochaeta fusca Blume var. obtuso-acuminata Blume, Flora 14: 497. 1831. Type: Indonesia. West Java: Buitenzorg, Tjiampea, C.L. Blume s.n. (lectotype, designated by Kartonegoro and Veldkamp 2010, pg. 143: L [L0537242]!; isolectotypes: L [L0537246]!).
Dissochaeta velutina Blume, Flora 14: 497. 1831. Type: Indonesia. Java, Bantam, Leuwi Boengoer, H. Kuhl & J.C. van Hasselt s.n. (lectotype, designated by Kartonegoro and Veldkamp 2010, pg. 143: L [L0537234]!; isolectotypes: K [K000859622]!, L [L0537249, L0537250]!).
Dissochaeta decipiens Blume, Mus. Bot. 1(3): 36. 1849. Type: Indonesia. Java, H. Kuhl & J.C. van Hasselt s.n. (lectotype, designated by Kartonegoro and Veldkmap 2010, pg. 131: L [L0008892]!; isolectotypes: K [K000859489]!, L [L0008891]!).
Dissochaeta inappendiculata Blume var. fusca (Blume) Miq., Fl. Ned. Ind. 1(1): 525. 1855.
Dissochaeta monticola auct. non Blume: Triana, Trans. Linn. Soc. London 28: 83. 1872. *p.p.*, excl. type.
Neodissochaeta fusca (Blume) Bakh.*f.*, Contr. Melastom.: 136. 1943.
Neodissochaeta vacillans (Blume) Bakh.*f.*, Contr. Melastom.: 144. 1943.

##### Type.

Indonesia. West Java: Buitenzorg, Tjiawi, C.G.C. Reinwardt s.n. (lectotype, designated by Kartonegoro and Veldkamp 2010, pg. 143: L [L0008894]!; isolectotype: L [L0008895]!).

**Figure 27.**
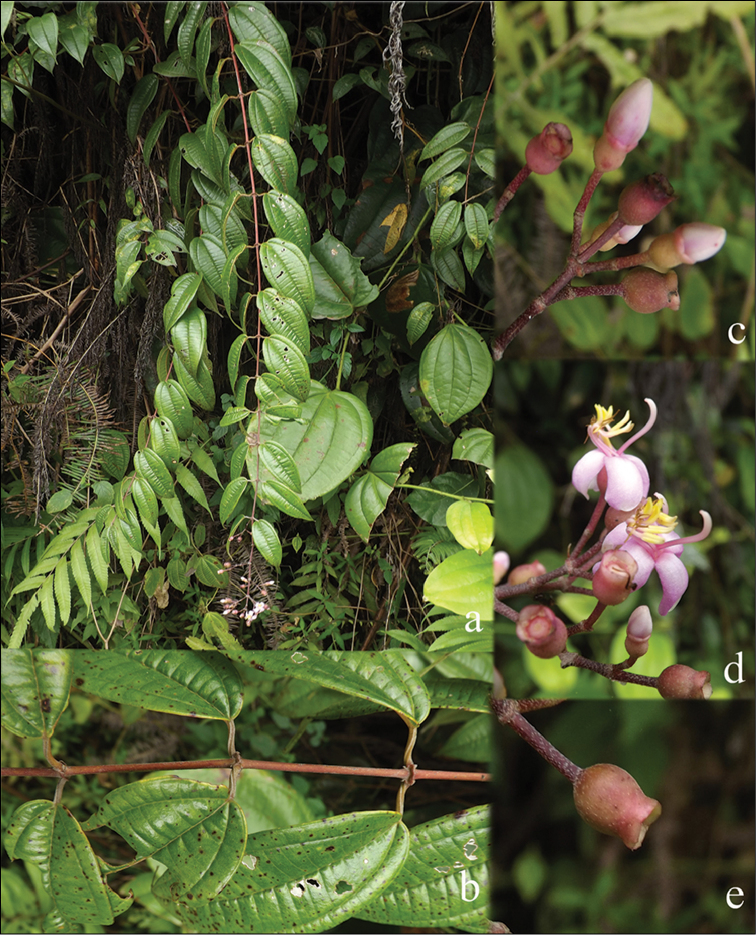
*Dissochaetavacillans*. **a** habit **b** branchlet **c** hypanthium **d** flowers **e** immature fruit. Photographs by A. Kartonegoro; voucher: Kartonegoro 1105 (BO).

##### Description.

Climbing up to 3 m height. Branchlets terete or subquadrangular, 2–6 mm diameter, glabrescent to stellate-furfuraceous; nodes swollen, with interpetiolar ridge; internodes 4.5–10 cm long. Leaves: petioles terete, 5–24 mm long, puberulous to furfuraceous; blades ovate, ovate-oblong or elliptic-oblong, 6.5–15 × 2.5–7 cm, membranous, base rounded, margin entire, apex acuminate, tip 0.5–2 cm long; nervation with 1 pair of lateral nerves and 1 pair of intramarginal nerves; adaxially glabrous, abaxially glabrous to nearly brown sparsely stellate-furfuraceous. Inflorescences terminal and in the upper leaf axils, up to 30 cm long, many-flowered; main axis angular, sparsely stellate-furfuraceous; peduncle up to 7 cm long; primary axes up to 25 cm long with 4–6 nodes, secondary axes up to 4 cm long with 1–3 nodes, tertiary axes up to 1.5 cm long with 1 node; bracts minute or linear, ca. 3 mm long, furfuraceous, caducous; bracteoles minute or linear to oblong, 1.5–4 mm long, stellate-furfuraceous; pedicels sparsely stellate-furfuraceous, 4–6 mm long in central flowers, 1–3 in lateral flowers. Hypanthium campanulate or suburceolate, 2–5 × 1–3 mm, glabrescent or sparsely to densely stellate-furfuraceous; calyx lobes truncate, 0.5–1 mm long, with 4 undulating apices, rounded, or subtriangular, glabrous; petal buds conical, 2–4 mm long; mature petals ovate or oblong, 5–6 × ca. 3 mm, base clawed, apex obtuse, glabrous or with minute hairs at margin, white-pinkish or pink. Stamens 8, sometimes 4 with the oppositipetalous ones undeveloped, unequal when 8, filaments straight; alternipetalous stamens with 2–4 mm long filaments, anthers oblong or lanceolate, thecae 3–5 mm long, yellow, pedoconnective ca. 0.5 mm long, basal crests triangular, 1–2 mm long, margin irregular, lateral appendages paired, filiform, 1.5–3 mm long; oppositipetalous stamens when developed with 2–3 mm long filaments, anthers ovate-oblong or lanceolate, thecae 2.5–4 mm long, basal crests triangular or ligular, 0.5–1 mm long, erect, lateral appendages paired or reduced to a single lateral one, filiform, 1–2 mm long. Ovary half or ⅔ of hypanthium in length, apex puberulous or pubescent; style 5–8 mm long, apex curved, glabrous; stigma minute; extra-ovarial chambers 8, the 4 alternipetalous ones extending between the apex and the middle of the ovary, the 4 oppositipetalous ones shallow. Fruits subglobose to urceolate, 3–6(–10) × 2–6 mm, glabrous; stalks 2–5(–7) mm long, calyx lobe persistent, erect. Seeds ca. 0.5 mm long.

##### Distribution.

Java and Lesser Sunda Islands (Sumbawa).

##### Ecology and habitat.

Forest, secondary or depleted forest or edge of river at 500–1400 m elevation.

##### Vernacular names.

Java: *harendong areuy, harendong bokor areuy, harendong gede* (Sunda).

##### Note.

*Dissochaetadecipiens*, with only four fertile, alternipetalous stamens, is synonymised with *D.vacillans*, because it has a similar appearance due to the indumentum on the branchlets, leaves and inflorescences; moreover, the shape of the stamens and the appendages are also similar.

##### Specimens examined.

**INDONESIA. Banten**: Pandeglang, Mt. Karang, C.A. Backer 7470 (BO); *Ibid.*, Galusur, 500 m, 28 May 1912, S.H. Koorders 40659β (BO); *Ibid.*, 700 m, 1 Jun 1912, S.H. Koorders 40727β (BO); *Ibid.*, Menes, Mt. Pulasari, 1000 m, Mar 1913, C.A. Backer 7055 (BO); Lebak, J.C. van Hasselt s.n. (L); *Ibid.*, Leuwi Bungur, H. Kuhl & J.C. van Hasselt s.n. (L); *Ibid.*, Citorek & Muncang, C.A. Backer 1835 (BO). **Central Java**: Mt. Slamet, Baturaden, 1000 m, 30 Mar 1970, B.J. Bernardius D43051 (BO); Pekalongan, Josorejo, C.A. Backer 16219 (BO). **West Java**: Bogor, Mt. Salak, Gunung Bunder to Kawah Ratu, 1300 m, 8 Jan 1941, C.N.A. de Voogd & S. Bloembergen s.n. (BO); *Ibid.*, C.A. Backer 4198 (BO); *Ibid.*, Upper Lido, 1200 m, 22 Feb 2000, H. Wiriadinata & W.S. Hoover 31188 (BO); *Ibid.*, W.S. Hoover & M. Hendra 32564 (BO); Leuwiliang, Puraseda, 500 m, 2 Feb 1929, C.G.G.J. van Steenis 2712 (BO); *Ibid.*, Mt. Butik Buligir, C.A. Backer 6150 (BO); *Ibid.*, Mt. Sunarari, 1000 m, 1 Jan 1913, C.A. Backer 6380 (BO); Ciampea, C.L. Blume s.n.(L); Ciawi, C.G.C. Reinwardt s.n. (L); Puncak Pass, Tugu, Above Gunung Mas, 1300-1500 m, 18 Mar 1952, W. Meijer 105 (BO, K, L); *Ibid.*, Gunung Luhur, Tugu, 1700 m, 8 Aug 1982, M.M.J. van Balgooy & J.P. Mogea 4284 (BO); Mount Pangrango, Bodogol, A. Kartonegoro 318 (BO); Mt. Halimun, C.A. Backer 10914 (BO); *Ibid.*, R.C. Bakhuizen van den Brink 3336 (BO, L, U); *Ibid.*, Cikaniki, 1000 m, 11 Jan 2001, D. Arifiani et al. 142 (BO); *Ibid.*, 9 Mar 2000, W.S. Hoover, D. Girmansyah & J. Hunter 32172 (BO); *Ibid.*, Nirmala, 1300 m, 10 Jun 1980, M.M.J. van Balgooy & H. Wiriadinata 2922 (BO, L); *Ibid.*, Malasari, 1055 m, 10 Oct 2017, A. Kartonegoro 1105 (BO); Cianjur, Mt. Gede, Cibodas, 1450 m, J.G. Boerlage s.n. (BO); *Ibid.*, R.H.C.C. Scheffer s.n. (BO); *Ibid.*, 3 Nov 1987, E.A. Widjaja 3220 (BO); *Ibid.*, Sindanglaya, 1000 m, Dec 1916, C.A. Backer 21507 (BO); *Ibid.*, Pasir Pangsalatan, 1500 m, 2 Jun 1948, Enoh 181 (BO, L); *Ibid.*, S.H. Koorders 31670β (BO); Sukanegara, E.R. Hellendoorn 5 (BO); Cibeber, Campaka, 1000 m, 16 Jun 1923, J.J. Smith 822 (BO, L); *Ibid.*, Cidadap, Cadas Malang, 1000 m, 19 Apr 1916, R.C. Bakhuizen van den Brink 1469 (BO); *Ibid.*, 20 Mar 1923, W.F. Winckel 1176β (BO,K, L); Takokak, 1200 m, 9 Jun 1900, S.H. Koorders 33314β (BO); *Ibid.*, S.H. Koorders 33315β (BO); Bandung, Cigenteng, 1400 m, 26 Jan 1897, S.H. Koorders 26306β (BO); Mt. Tangkuban Prahu, 1600 m, 26 Jul 1927, W.M. Docters van Leeuwen 11487a (BO); Mt. Sembung, Margalangu, 1200 m, 19 Mar 1914, C.A. Backer 12298 (BO); Garut, Rawa Cangkuang, R.H.C.C. Scheffer s.n. (BO); *Ibid.*, Pasawahan, 400 m, 31 Dec 1911, C.A. Backer 2261 (BO); *Ibid.*, Mt. Ciparay, 1100 m, 27 Jul 1914, C.A. Backer 15041 (BO); Mt.Cikuray, Pasir Kolotok, 1000 m, 15 Aug 1913, C.A. Backer 8685 (BO); Tasikmalaya, Panjalu, 720 m, 4 Aug 1917, S.H. Koorders 47851β (BO). **West Nusa Tenggara.** Sumbawa, Sumbawa Barat, Mt. Batulante, 700 m, 3 Nov 1961, A.J.G.H. Kostermans 19164 (BO, K, P).

#### 54. Dissochaeta viminalis

Taxon classificationPlantaeMyrtalesMelastomataceae

(Jack) Clausing in S.S.Renner et al., Fl. Thailand 7(3): 433. 2001.

Fig. 28 , Map 28

Melastoma viminale Jack, Trans. Linn. Soc. London 14: 16. 1823 (“viminalis”).
Melastoma rostratum Blume, Bijdr. Fl. Ned. Ind 17: 1074. 1826. Type: Indonesia. West Java, G. Seribu, C.L. Blume 856 (lectotype, designated by Veldkamp et al. 1979, pg. 427: L [L0008883]!; isolectotype: L [L0008882]!).
Aplectrum viminale (Jack) Blume, Flora 14: 502. 1831.
Aplectrum rostratum (Blume) Blume, Flora 14: 502. 1831.
Aplectrum pallens Blume, Mus. Bot. 1(3): 38. 1849. Type: Indonesia. West Sumatra, P.W. Korthals s.n. (lectotype, designated by Veldkamp et al. 1979, pg. 428: L [L0008877]!; isolectotypes: K [K000859618]!, L [L0008875, L0008876]!, P [P02274926, P02274927, images seen]!).
Aplectrum confine Blume, Mus. Bot. 1(3): 38. 1849. Type: Indonesia. West Sumatra, P.W. Korthals s.n. (lectotype, designated by Veldkamp et al. 1979, pg. 427: L [L0008878]!; isotypes: K [K000859620]!, L [L0008879, L0008880]!, P [P02274925, image seen]!).
Aplectrum pallens Blume var. latum Miq., Fl. Ned. Ind. 1(1): 554. 1855. Type: Indonesia. West Sumatra: Mount Malintang, P.W. Korthals s.n. (lectotype, designated by Veldkamp et al. 1979, pg. 427: L [L0008873]!; isolectotypes: K [K000859619]!; L [L0008874]!, P [P02274928, P02274929, images seen]!).
Aplectrum pallens Blume var. confinis (Blume) Miq., Fl. Ned. Ind. 1(1): 554. 1855.
Anplectrum viminale (Jack) Triana, Trans. Linn. Soc. London 28: 84. 1872.
Anplectrum rostratum (Blume) Triana, Trans. Linn. Soc. London 28: 84. 1872.
Anplectrum pallens (Blume) Triana, Trans. Linn. Soc. London 28: 84. 1872.
Anplectrum confine (Blume) Triana, Trans. Linn. Soc. London 28: 84. 1872.
Diplectria viminalis (Jack) Kuntze, Revis. Gen. Pl. 1: 246. 1891.
Diplectria rostrata (Blume) Kuntze, Revis. Gen. Pl. 1: 246. 1891.
Diplectria confinis (Blume) Kuntze, Revis. Gen. Pl. 1: 246. 1891.
Dissochaeta anomala King, J. Asiat. Soc. Bengal, Pt. 2, Nat. Hist. 69(2): 55. 1900. Type: Malaysia. Perak: Larut, Aug 1881, King’s collector (Kunstler) 2258 (lectotype, designated here: K [K000859556]!; isolectotypes: BM!, CAL *n.v.*, SING *n.v.*).
Backeria viminalis (Jack) Bakh.*f.*, Contr. Melastom. 133. 1943.
Backeria viminalis (Jack) Bakh.*f.* var. rostrata (Blume) Bakh.*f.*, Contr. Melastom.: 134. 1943.
Backeria viminalis (Jack) Bakh.*f.* var. confinis (Blume) Bakh.*f.*, Blumea 12: 61. 1963.
Backeria pallens (Blume) Raizada, Indian Forester 96: 435. 1968.
Diplectria anomala (King) Veldkamp in Veldkamp et al., Blumea 24: 426, fig. 5C. 1979.

##### Type.

Indonesia. Sumatra, *Jack s.n.* (lost); Indonesia. West Java, G. Seribu, C.L. Blume 856 (neotype, designated by Veldkamp et al. 1979, pg. 427, erroneously ascribed to Kuhl & Van Hasselt: L [L0008883]!; isoneotype: L [L0008882]!).

**Figure 28.**
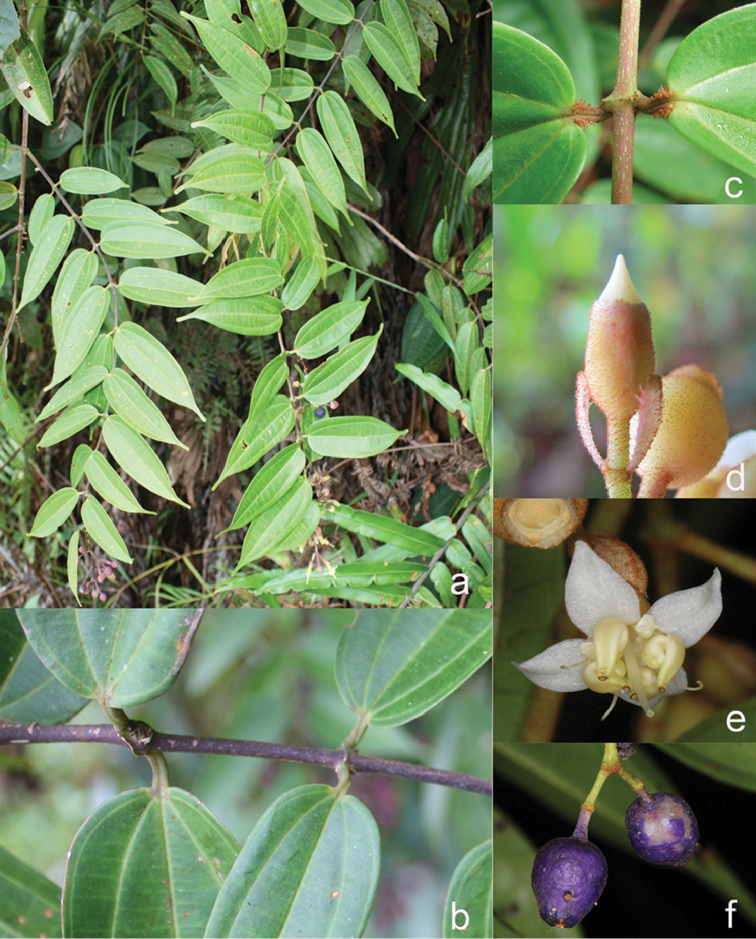
*Dissochaetaviminalis*. **a** habit **b** branchlet **c** leaf node **d** hypanthium **e** flower **f** fruits. Photographs by A. Kartonegoro; vouchers: Kartonegoro 1075 (BO, L)]

**Map 28.**
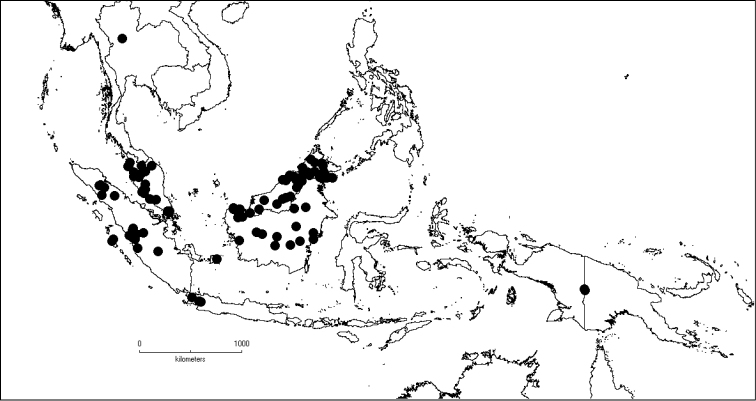
Distribution of *D.viminalis* (●).

##### Description.

Climbing up to 5 m in height; branchlets terete, 3–5 mm in diameter, glabrous; nodes swollen, with raised interpetiolar ridge, covered with stellate hairs; internodes 3–4 cm long. Leaves: petioles terete, 3–8 mm long, glabrous to minutely stellate puberulous, densely covered with red-brown bristles lateral of the groove and near the attachment with the blade; blades ovate, elliptic-oblong to oblong, (2.5–)5–14 × (1–)2–6 cm, membranous, base rounded to cordate when large, margin entire, apex acute or acuminate, tip 8–15 mm long; nervation with 1 pair of lateral nerves and 1 pair of intramarginal nerves; adaxially glabrous, abaxially glabrous with sparsely stellate hairs at the midrib and nerves. Inflorescences terminal and axillary, when terminal up to 12 cm long, many-flowered, when axillary ones up to 5.5 cm long, 3–9-flowered; main axis stellate-puberulous; primary axes up to 5 cm long with 2–4 nodes, secondary axes up to 2 cm long with 1–3 nodes, tertiary axes when developed up to 0.5 cm long with 1 node; bracts linear or elliptic, 3–6 × 3–4 mm, stellate-puberulous, caducous; bracteoles ovate to oblong, 3–4 mm long, stellate-puberulous, margin ciliate; pedicels glabrescent to stellate-puberulous, 3–5 mm long in central flowers, 1–2 mm long in lateral flowers. Hypanthium cyathiform-tubular, 3–4 × 2–2.5 mm, glabrous or stellately-puberulous; calyx lobes truncate, ca. 0.5 mm long, without any distinct tips; petal buds conical, 1–2 mm long, apex acuminate; mature petals ovate, 4–5 × ca. 3 mm, reflexed, base clawed, apex acute, white. Stamens 8, unequal, filaments flattened, straight; alternipetalous stamens staminodial with 1–2 mm long filaments, anthers rudimentary, thecae 2–3 mm long, slender, terete, curved, white, basal crest ovate, thin, ca. 1 mm long, lateral appendages paired, flat, filiform, 1–1.5 mm long; oppositipetalous stamens with 1.5–2 mm long filaments, anthers thick, curved, hook-shaped, thecae 2–2.5 mm long, apex obtuse, white, basal crest consisting of a short pair of keels, ca. 0.2 mm long, lateral appendages absent. Ovary half as long as hypanthium, apex glabrous; style 5–6 mm long, curved at the end, slender, glabrous, pink; stigma minute; extra-ovarial chambers 4, oppositipetalous, shallow, extending only upper ⅓ of the ovary. Fruits subglobose, 4–5 × 3–4 mm, glabrous or stellate-puberulous; calyx lobe remnants persistent. Seeds ca. 0.5 mm long.

##### Distribution.

Thailand, Malay Peninsula, Sumatra, Java, Borneo and New Guinea.

##### Ecology and habitat.

Lowland and mixed dipterocarp forest, open areas, road sides or river banks at 45–1150 m elevation.

##### Vernacular names.

Peninsular Malaysia: *akar sindodo* (Kelantan); *laka tulang* (Penang). Sumatra: *kadudu besar* (Jambi); *karamunting akar* (Belitung). Borneo: *akar kemunting* (Iban); *wa perawi* (Kelabit).

##### Note.

This species is easily recognised by the glabrous branchlets and the dense, parallel, brown bristle hairs at the apex of the petioles. The shape and number of stamens resemble those of *D.stipularis* and *D.maxwellii*. The neotype of *Melastomaviminale* Jack that was chosen by Veldkamp et al. (1979) in Leiden was said to be collected by Kuhl & Van Hasselt from Mount Seribu in West Java. It is seemingly the specimen collected by Blume, who visited the mountain in 1824 while Kuhl & Van Hasselt never visited that locality. He mostly did collecting in Banten (Bantam) Province (Van Steenis-Kruseman 1950).

##### Selected specimens examined.

**THAILAND. Sukhotai**: Kalung Tan, 400 m, 11 Mar 1928, A.F.G. Kerr 14461 (BM, K). **Narathiwat**: Phu Khao Thong, Lamthan Thong Reservoir, 120 m, 21 Jul 2004, R. Pooma et al. 4520 (L). **MALAYSIA. Johor**: Tebing Tinggi, Nov 1900, H.N. Ridley 11105 (K). **Kedah**: Gunong Jerai, Sungai Terol, 762 m, 12 Sep 1979, H. Keng et al. 122 (L). **Kelantan**: Kuala Tapah, Ulu Sungai Aring, 21 Sep 1967, P.F. Cockburn FRI 7154 (K, L). **Malacca**: A.C. Maingay KD 795 (2663) (K, L). **Pahang**: Raub, Sungai Sempan, 16 Apr 1970, E. Soepadmo 653 (K). **Penang**: N. Wallich 4053 (BM, K); West Hill, Mar 1915, H.N. Ridley s.n. (BM, K); Penang Hill, 22 Aug 1879, King’s collector s.n. (P). **Perak**: Larut Hill, King’s collector 2258 (BM, K); Ulu Bubong, King’s collector 10468 (BM, K); Gunong Bubu, 600 m, 18 Aug 1966, W.L. Chew 1230 (K, L); Taiping Hill, Batu Hampar, 8 Dec 1965, M. Shah & Sidek 1163 (K, L); Maxwell Hill, May 1886, L. Wray 641 (K). **Selangor**: Rantau Panjang, Jul 1914, C. Boden-Kloss s.n. (BM, K); Ayer Hitam, Puchong, 17 Jan 1968, Y. Teo & J.W. Purseglove 23 (K, L). **Sabah**: Kalabakan, Hap Seng, 20 May 1982, Fidilis SAN 94808 (K); Keningau, Sepulut, 20 Oct 1983, Sigin & Francis SAN 69084 (K, L); Kinabatangan, Nurod-Urod, 680 m, 20 Nov 2006, J.B. Sugau et al. SAN 149046 (K); Lahad, Datu, Danum Valley, 3 May 1989, C.E. Ridsdale 1935 (L); Lamag, Inarat, Gunong Lotung 381 m, 7 May 1976, P.F. Cockburn SAN 83025 (L); Ranau, Mount Kinabalu, Dallas, 914 m, 27 Oct 1931, J. Clemens & M.S. Clemens 26872 (BM); Sandakan, Telupid Road, 152 m, 23 Sep 1969, A. Talip & Termiji SAN 67969 (L); Tawau, 1923, A.D.E. Elmer 21305 (BM, L, P, U); *Ibid.*, A.D.E. Elmer 21649 (BM, L, P, U). **Sarawak**: O. Beccari PB 372 (P); Native collector 86 (L, P, PNH); Native collector 580 (BM, L, P, PNH); Bario, Kelabit Highlands, 1000 m, 25 Mar 1970, H.P. Nooteboom & P.K. Chai 1664 (L); Kapit, Balleh, Ulu Sungai Melinau, 24 Apr 1976, P.K. Chai et al. S.37213 (L); Kuching, Sep 1892, G.D. Haviland & C. Hose 159 (L); *Ibid.*, 26 Jan 1894, G.D. Haviland & C. Hose 971 (BM, L); Lubok Antu, Ulu Sungai Kaup, Bukit Ubah-Ribu 600 m, 12 Mar 1974, P.K. Chai S.33794 (L); Lundu, Gunung Undan 200 m, 30 Apr 1983, P.C. Yii & Jegong S.45974 (L); Miri, Lambir Hill, 21 May 1966, Sibat ak Luang S.24301 (BO, L); Serian, Gunung Rawan 830 m, 5 Apr 1983, D. Awa & Ilias Paie S.45555 (L). **SINGAPORE.** A.C. Maingay KD 2492 (BM); Mandai Road, 16 Feb 1936, E.J.H. Corner SFN 30666 (K); Pulau Ubin, 1890, H.N. Ridley 2014 (BM); Nee Soon 10 m, 28 Nov 1980, J.F. Maxwell 80-210 (L); MacRitchie Resevoir, Thompson Ridge 5 m, 2 Jul 1981, J.F. Maxwell 81-158 (L); Pierce Reservoir 5 m, 18 Nov 1981, J.F. Maxwell 81-222 (L). **BRUNEI. Tutong**: Tasik Merimbun, 7 Apr 1988, K.M. Wong 347 (L). **INDONESIA. Aceh**: Mount Leuser, Lau Ketambe, Gunung Mamas 800-1000 m, 9 Feb 1975, W.J.J.O. de Wilde & B.E.E. de Wilde-Duyfjes 14660 (L). **Bangka-Belitung**: Belitung Island, Manggar, J.E. Teijsmann s.n. (BO, L). **Jambi**: Sungai Lesing, 30 m, Oct 1925, O. Posthumus 987 (BO, L). **North Sumatra**: Asahan, Dolok Tomuan, Jun 1936, Rahmat Si Boeea 9041 (L); Bohorok, Bukit Lawang, 30 Aug 1983, T.C. Whitmore & T. Kalima 3245 (K, L). **Riau**: Indragiri, Taluk, 13 Jan 1956, W. Meijer 4329 (L). **West Sumatra**: Lima Puluh Kota, Mount Malintang, P.W. Korthals s.n. (L); Harau Ravine, 500 m, 15 Aug 1956, M. Jacobs 4596 (L); *Ibid.*, 11 Sep 2017, A. Kartonegoro 1075 (BO, L); Sungei Bulu, Sep 1878, O. Beccari PS 913 (L); *Ibid.*, O. Beccari PS 934 (BM, L); Indrapura, P.W. Korthals s.n. (L, P); Sijunjung, Muaro Kalumpi, Sungai Kwantan, 170 m, 28 Feb 1974, E.F. de Vogel 2748 (BO, L); Solok, Talang Babungo, 1100 m, 26-27 May 2001, Chan et al. 85 (ANDA); Mentawai Islands, Siberut Island, 25 Sep 1924, Iboet 276 (BO, L). **Banten**: Gunung Seribu, H. Kuhl & J.C. van Hasselt 856 (L). **West Java**: Bogor, Leuwiliang, Pasir Sijahe 600 m, R.C. Bakhuizen van den Brink 6401 (BO, U); *Ibid.*, Gunung Ciputi 550 m, 17 Jun 1921, R.C. Bakhuizen van den Brink 853 (BO, U). **Central Kalimantan**: Buntok, Sarbaballo Lake, 21 Aug 1908, H.J.P. Winkler 3272 (L); Kapuas, Timpah-Buntok, 45 m, 11 Oct 2001, K. Sidiyasa et al. 2536 (K, L). **East Kalimantan**: West Kutai, Long Ibut, 130 m, 10 Aug 1925, F.H. Endert 2559 (BO, L); Long Sei Barang, 750 m, 13 May 1993, Ambriansyah AA 763 (L). **North Kalimantan**: Krayan, Long Bawan, Gunung Leputung, 900 m, 9 Jul 1981, M. Kato et al. B-7957 (BO, L). **West Kalimantan**: Pontianak, Bentiang, Gunung Mikei 750 m, 30 Oct 1980, G. Shea 27039 (BO, L); Sintang, Sungai Posang 110 m, 22 Apr 1994, A.C. Church et al. 1041 (BO, K, L). **PAPUA NEW GUINEA. Western District**: Kiunga, Ingembit, 91 m, 8 Jun 1967, E.E. Henty, C.E. Ridsdale & M. Galore NGF 31814 (L); *Ibid.*, 10 Jun 1967, C.E. Ridsdale, E.E. Henty & M. Galore NGF 31912 (K, L); *Ibid.*, 125 m, 29 May 1969, S. Reksodihardjo 295 (BO, L).

### Excluded taxa

*Anplectrumanomalum* King & Stapf ex King, J. Asiat. Soc. Bengal, Pt. 2, Nat. Hist. 69(2): 58. 1900 = ***Creochitonanomalus* (King & Stapf ex King) Veldkamp**, Blumea 24: 438. 1979.

*Anplectrumassamicum* C.B.Clarke in Hook.*f.*, Fl. Brit. India 2(6): 546. 1879 = ***Pseudodissochaetaassamica* (C.B.Clarke) M.P.Nayar**, J. Bombay Nat. Hist. Soc. 65(3): 559. 1969.

*Anplectrumhomoeandrum* Stapf, Trans. Linn. Soc. London, Bot. 4(2): 161. 1894 = ***Medinillahomoeandra* (Stapf) M.P.Nayar**, Kew Bull. 20: 240. 1966.

*Anplectrummonticola* Ridl., Kew Bull. 1: 31. 1946 = ***Creochitonmonticola* (Ridl.) Veldkamp**, Blumea 24: 438. 1979.

*Aplectrummyrtifolium* Miq., Fl. Ned. Ind. 1(1): 555. 1855 = ***Medinillamyrtiformis* (Naudin) Triana**, Trans. Linn. Soc. London 28: 86. 1872.

*Aplectrummyrtiforme* Naudin, Ann. Sci. Nat., Bot. sér. 3, 15: 305. 1851 = ***Medinillamyrtiformis* (Naudin) Triana**, Trans. Linn. Soc. London 28: 86. 1872.

*Anplectrumovalifolium* A.Gray, U. S. Expl. Exped., Phan. 15: 597. 1854 = ***Medinillaovalifolia* (A.Gray) A.C.Sm.**, Contr. U. S . Nat. Herb. 37: 85. 1967.

*Anplectrumparviflorum* Benth., Fl. Hongk.: 116. 1861 = ***Blastuscochinchinensis* Lour.**, Fl. Cochinch. 2: 527. 1790.

*Anplectrumrubifructus* (Ohwi) Ohwi, Bull. Natl. Sci. Mus., Tokyo 26: 12. 1949 = ***Medinillarubifructus* Ohwi**, Bot. Mag. (Tokyo) 57: 7. 1943.

*Anplectrumyunnanense* Kraenzl., Vierteljahrsschr. Naturf. Ges. Zürich 76: 153. 1931 = ***Pseudodissochaetaseptentrionalis* (W.W.Sm.) M.P.Nayar**, J. Bombay Nat. Hist. Soc. 65(3): 565. 1969.

*Dissochaetabarthei* Hance *ex* Benth., Fl. Hongk.: 115. 1861 = ***Bartheabarthei* (Hance *ex* Benth.) Krasser** in Engl. & Prantl, Nat. Pflanzenfam. 3(7): 175. 1893.

*Dissochaetabibracteata* (Blume) Baill., Hist. Pl. 7: 25. 1877 = ***Creochitonbibracteatus* (Blume) Blume**, Flora 14: 507. 1831.

*Dissochaetaheteromorpha* Naudin, Ann. Sci. Nat. Bot. sér. 3, 15: 78. 1851 = ***Dichaetantheraheteromorpha* (Naudin) Triana**, Trans. Linn. Soc. London 28: 61. 1872.

*Dissochaetanovoguineensis* Baker *f.*, J. Bot. 61 (Suppl.): 21. 1923 = ***Creochitonnovoguineensis* (Baker *f.*) Veldkamp & M.P.Nayar**, Blumea 24: 438. 1979.

*Dissochaetapentamera* Burkill, Bull. Misc. Inform. Kew: 5. 1906 = ***Poikilogynepentamera* (Burkill) Baker *f.*** in Gibbs, Phytogeogr. & Fl. Arfak Mts.: 157. 1917.

*Dissochaetaquintuplinervis* Cogn. in A.DC. & C.DC., Monogr. Phan. 7: 556. 1891 = ***Catantheraquintuplinervis* (Cogn.) M.P.Nayar**, Gard. Bull. Singapore 24: 353. 1969.

*Dissochaetasarcorhiza* Baill., Adansonia 12: 88. 1877 = ***Medinillasarcorhiza* (Baill.) Cogn.** in A.DC. & C.DC., Monogr. Phan. 7: 587. 1891.

## Supplementary Material

XML Treatment for Dissochaeta

XML Treatment for Dissochaeta acmura

XML Treatment for Dissochaeta alstonii

XML Treatment for Dissochaeta angiensis

XML Treatment for Dissochaeta annulata

XML Treatment for Dissochaeta atrobrunnea

XML Treatment for Dissochaeta axillaris

XML Treatment for Dissochaeta bakhuizenii

XML Treatment for Dissochaeta barbata

XML Treatment for Dissochaeta beccariana

XML Treatment for Dissochaeta biligulata

XML Treatment for Dissochaeta bracteata

XML Treatment for Dissochaeta brassii

XML Treatment for Dissochaeta celebica

XML Treatment for Dissochaeta celebica Blume var. celebica

XML Treatment for Dissochaeta celebica Blume var. longilobata

XML Treatment for Dissochaeta conica

XML Treatment for Dissochaeta cumingii

XML Treatment for Dissochaeta densiflora

XML Treatment for Dissochaeta divaricata

XML Treatment for Dissochaeta fallax

XML Treatment for Dissochaeta floccosa

XML Treatment for Dissochaeta glabra

XML Treatment for Dissochaeta glabra Merr. var. glabra

XML Treatment for Dissochaeta glabra Merr. var. kinabaluensis

XML Treatment for Dissochaeta glandiformis

XML Treatment for Dissochaeta glandulosa

XML Treatment for Dissochaeta gracilis

XML Treatment for Dissochaeta griffithii

XML Treatment for Dissochaeta hirsutoidea

XML Treatment for Dissochaeta horrida

XML Treatment for Dissochaeta inappendiculata

XML Treatment for Dissochaeta intermedia

XML Treatment for Dissochaeta johorensis

XML Treatment for Dissochaeta laevis

XML Treatment for Dissochaeta latifolia

XML Treatment for Dissochaeta leprosa

XML Treatment for Dissochaeta macrosepala

XML Treatment for Dissochaeta malayana

XML Treatment for Dissochaeta marumioides

XML Treatment for Dissochaeta maxwellii

XML Treatment for Dissochaeta micrantha

XML Treatment for Dissochaeta nodosa

XML Treatment for Dissochaeta pallida

XML Treatment for Dissochaeta papuana

XML Treatment for Dissochaeta porphyrocarpa

XML Treatment for Dissochaeta pubescens

XML Treatment for Dissochaeta pulchra

XML Treatment for Dissochaeta punctulata

XML Treatment for Dissochaeta rectandra

XML Treatment for Dissochaeta rostrata

XML Treatment for Dissochaeta rubiginosa

XML Treatment for Dissochaeta sagittata

XML Treatment for Dissochaeta sarawakensis

XML Treatment for Dissochaeta schumannii

XML Treatment for Dissochaeta spectabilis

XML Treatment for Dissochaeta stipularis

XML Treatment for Dissochaeta vacillans

XML Treatment for Dissochaeta viminalis

## Index to Numbered Collections

Abbe, E.C. 9061 (inappendiculata).

Abdul Samat bin Abdullah 133 (biligulata).

Achmad 231, 1197 (annulata); 1757 (gracilis); 1794 (annulata).

Adelbert, A.G.L. 459 (gracilis); 476 (inappendiculata); 478 (gracilis).

Aet 294 (stipularis); 599 (glabra var. glabra); 669 (biligulata); 739 (axillaris).

Afriastini, J.J. 427 (rostrata); 2125A (celebica var. celebica).

Ajoeb 115 (gracilis); 350 (fallax).

Albertus 45 (beccariana); 2236 (rostrata).

d’Alleizette, C. 2420 (biligulata); 2445 (divaricata).

Alphonso, Sanusi & Sidek 197 (johorensis); 200 (biligulata).

Alston, A.H.G. 12928 (fallax); 13234 (divaricata); 13375 (rostrata); 13816 (spectabilis); 14627 (gracilis); 14628 (inappendiculata); 14727 (divaricata); 14790, 14791 (bakhuizenii); 14813 (alstonii); 14816 (divaricata); 15233 (biligulata).

Amdjah 115, 404 (pulchra).

Andau 86 (biligulata).

Anderson, J.A.R. & Keng, H. K.7 (sarawakensis).

Anderson, T. 67 (biligulata); 92 (inappendiculata); s.n. (vacillans).

Arbain, D. & Tamin, R. 12 (stipularis); 47 (divaricata); 122 (biligulata).

Ardiyani, M. E.167 (divaricata).

Argent, G.C.G. 108277 (glabra var.glabra).

Argent, G.C.G. & Coppins, B. 1160 (porphyrocarpa).

Argent, G.C.G. & Wilkie, P. 9441 (glandulosa).

Arief 225 (gracilis).

Arifiani, D. 74 (gracilis); 136 (inappendiculata); 142 (vacillans); 922 (gracilis).

Armstrong, K. 627 (barbata).

Arsin 19556 (intermedia).

Atje 318 (divaricata).

Australian National University Number (ANU) 1541 (celebica var. celebica).

Azuma, H. A259 (fallax).

Backer, C.A. 186 (gracilis); 296 (inappendiculata); 774, 1249, 1697 (gracilis); 1722 (fallax); 1835 (vacillans); 1839 (bakhuizenii); 2261 (vacillans); 2386 (leprosa); 2387, 2415 (intermedia); 2479 (fallax); 4031, 4130 (gracilis); 4139 (bracteata); 4198 (vacillans); 4201 (inappendiculata); 4206 (fallax); 6027 (gracilis); 6150 (vacillans); 6272 (sagittata); 6305 (divaricata); 6314 (gracilis); 6379 (fallax); 6380 (vacillans); 7032, 7054 (gracilis); 7055, 7470 (vacillans); 8619 (fallax); 8685 (vacillans); 8687 (inappendiculata); 8726 (fallax); 8761 (inappendiculata); 8790, 8940 (gracilis); 9097 (sagittata); 10060 (gracilis); 10358, 10373, 10449 (fallax); 10522 (inappendiculata); 10549 (sagittata); 10571 (fallax); 10579 (gracilis); 10794 (inappendiculata); 10914 (vacillans); 11160 (gracilis); 12256 (inappendiculata); 12298 (vacillans); 12787 (intermedia); 14108, 14361 (gracilis); 14714 (leprosa); 15019 (gracilis); 15041 (vacillans); 15119, 15750 (gracilis); 15787, 15983 (leprosa); 16056 (gracilis); 16219 (vacillans); 17129, 18668 (gracilis); 21507 (vacillans); 22286 (intermedia); 22493 (gracilis); 22623, 22891, 23703, 23839 (inappendiculata); 25698 (fallax); 25705 (bakhuizenii); 25714 (gracilis); 25893 (fallax); 25894 (gracilis); 26007 (fallax); 31626, 31627, 31628 (gracilis).

Bakhuizen van den Brink, R.C. 195 (fallax); 732 (inappendiculata); 872 (fallax); 1469 (vacillans); 1474, 1495, 1631, 2049 (fallax); 2731 (gracilis); 2769 (fallax); 3156 (gracilis); 3548 (fallax); 3839, 3892, 3922 (gracilis); 4557 (intermedia); 5181 (divaricata); 6048 (gracilis); 6165 (divaricata); 6401 (viminalis); 6854, 7550 (divaricata); 7641 (gracilis).

Bakhuizen van den Brink Jr, R.C. 853 (viminalis); 1305 (gracilis); 2328 (divaricata); 2591 (fallax); 3157 (gracilis); 3287 (inappendiculata); 3301 (gracilis); 3336 (vacillans).

Balakrishnan, N.P. 3899 (biligulata).

Bangham, W.N. & Bangham, C.N. 951 (bakhuizenii); 1140 (divaricata).

Bangkok Forestry Number (BKF) 36947 (divaricata); 40970 (gracilis); 46194 (divaricata); 46409 (gracilis); 46605 (divaricata); 46913 (biligulata); 52202 (gracilis); 54194 (biligulata).

Bannaniers 5003 (barbata).

Bartlett, H.H. 6900, 7315 (pallida).

Bartlett, H.H. & La Rue, C.D. 435 (gracilis).

Beaman, J.H. 7030 (divaricata); 7110 (glandulosa); 10653 (stipularis); 11090 (gracilis); 11232 (divaricata).

Beaman, R.S. et al. 10647 (annulata).

Beaman, T.E. & Repin, R. 164 (biligulata).

Beccari, O. PB372 (viminalis); PB800 (latifolia); PB809 (pubescens); PB834 (viminalis); PB1089 (latifolia); PB2186 (divaricata); PB2190 (beccariana); PB3282 (annulata); PS369 (leprosa); PS913, PS934 (viminalis); PS4124 (nodosa); s.n. (marumioides).

Beddome, R.H. 3066 (divaricata).

Bernard, C. et al. 3 (viminalis); 8 (biligulata).

Bernardius, B.J. 43051 (vacillans).

Beumée, J.G.B. A390 (intermedia).

Bicknell, D. 1490 (acmura).

Bidin 105 (gracilis).

Binnendijk, S. HB359 (fallax); HB360, HB418 (inappendiculata).

Blume, C.L. 11 (sagittata); 539 (intermedia); 856 (viminalis); 857 (stipularis); 1791 (vacillans); 3084 (gracilis).

Boerlage, J.G. 32 (gracilis).

Boonkongchart, A. 210 (divaricata).

Boschwezen Number (BW) 6783 (annulata).

Boyce, P.C. 392 (pubescens).

Brass, L.J. 23485, 27443, 28743 (brassii).

Bremer, K. 1840 (johorensis).

British North Borneo Number (A) 418 (divaricata); 456 (annulata); 2987 (glabra var. glabra).

Brooke, W.M.A. 8757 (divaricata); 9010, 9669 (hirsutoidea); 9714 (pubescens); 9998 (viminalis); 10034 (rostrata); 10091 (pulchra); 10267 (viminalis); 10372, 10391 (divaricata).

Brooks, C.J. 6675 (biligulata); s.n. (pallida).

Bruggeman, M.L.A. 48, 211, 839 (leprosa).

Brunei Forestry Series Number (BRUN) 671 (biligulata); 15203 (hirsutoidea); 15229, 15465, 15570 (stipularis); 16512 (hirsutoidea); 16601 (stipularis); 16786 (pubescens); 17315 (beccariana).

Bünnemeijer, H.A.B. 124 (inappendiculata); 125 (gracilis); 1053 (bakhuizenii); 1169 (inappendiculata); 1675 (pallida); 3015 (gracilis); 3094 (conica); 3095 (gracilis); 3125 (fallax); 3187 (bakhuizenii); 3199 (gracilis); 3200 (horrida); 3467 (gracilis); 3468 (fallax); 3587 (biligulata); 4225, 5263, 4514 (fallax); 6114 (biligulata); 6158 (annulata); 6162 (punctulata); 7096 (johorensis); 7160 (biligulata); 7175, 7276 (johorensis); 7633 (punctulata); 8074 (glandiformis); 8937 (inappendiculata); 9230, 9379, 10477 (glandiformis).

Burck, W. 26, 30 (fallax).

Bureau Science Number (BS) 16612, 22859, 23644, 29114, 30366 (acmura); 38870 (celebica var. celebica); 43902 (axillaris); 44461 (divaricata); 46108 (bracteata); 48858 (celebica var. celebica).

Burkill, H.M. 750 (spectabilis); 1063 (divaricata); 1227 (biligulata); 1822 (johorensis).

Burley, J.S. & Afriastini, J.J. 512 (viminalis).

Burley, J.S. & Tukirin 852 (atrobrunnea); 1084, 1471 (divaricata); 1472 (biligulata); 1479 (divaricata); 2692, 2827 (hirsutoidea); 2857, 2877 (beccariana).

Buwalda, P. 6535 (biligulata); 6666 (pallida).

Bygrave, P. 29 (sarawakensis).

Cantley, N. 62 (punctulata).

Carrick, J.C. 1428 (divaricata); 1474 (annulata); 1569 (conica); 1574 (inappendiculata); 1575 (divaricata); 1606 (rectandra); 1613 (annulata).

Chan 85 (viminalis).

Charoenchai, P. 260 (divaricata).

Cheine 908 (divaricata).

Chew, W.L. 270 (griffithii); 426 (annulata); 597 (rostrata); 1230 (viminalis); 1236 (gracilis); 1428 (hirsutoidea).

Chongko, S. 315 (gracilis).

Christensen, H.M. 717 (divaricata); 1244 (rostrata).

Church, A.C. 273 (pubescens); 636 (viminalis); 637 (annulata); 832 (rostrata); 1000 (beccariana); 1041 (viminalis); 1078 (gracilis); 1373 (annulata).

Clemens, J. & Clemens, M.S. 1395 (angiensis); 20270 (pubescens); 20929 (sarawakensis); 21138 (divaricata); 21139 (densiflora); 21140 (pulchra); 21568 (divaricata); 21569 (densiflora); 21570 (divaricata); 21579 (pulchra); 22285 (divaricata); 26028 (porphyrocarpa); 26058, 26058A (rubiginosa); 26667 (pubescens); 26872 (viminalis); 28588 (micrantha); 29442 (glabra var. kinabaluensis); 30339 (bracteata); 30340 (macrosepala); 30341 (micrantha); 30342 (beccariana); 30357, 30359 (porphyrocarpa); 30389 (pubescens); 30865, 31520 (glabra var. kinabaluensis); 32150 (glandulosa); 40940, 50337, 50476, 50555A (glabra var. kinabaluensis).

Clemens, M.S. 1155 (acmura); 9773 (biligulata); 9816 (bracteata); 10122 (biligulata); 10301 (pubescens); 10789, 10824 (porphyrocarpa); 10941 (rubiginosa).

Conn, B. 1746 (annulata).

Coode, M.J.E. 7227 (porphyrocarpa); 7772 (biligulata); 7864 (glandulosa); 7900 (viminalis); 7912 (rostrata).

Cramer, P.J.S. 90 (gracilis); 107 (fallax).

Cuming, H. 815 (acmura); 1344 (cumingii); 2259 (divaricata); 2838, 2840 (acmura).

Curtis, C. 78 (fallax); 80 (pallida); 398 (gracilis); 471 (viminalis); 740 (annulata); 1078 (stipularis); 1301 (biligulata); 2297 (pallida); 2298 (bracteata); 2433, 2725, 3806 (biligulata).

Dakkus, P.W. 169 (fallax); 193 (gracilis).

Dames, T.W.G. 72 (inappendiculata).

Danimihardja, S. 2248 (gracilis).

Danser, B.H. 5955 (leprosa).

Darbyshire, P.J. 247 (brassii).

Darnton, S. 230, 231 (biligulata); 505 (bracteata).

Davies, S.J. 99128 (beccariana).

De Jong, W. 749 (rostrata).

De Monchy, P. 27 (inappendiculata); s.n. (fallax).

De Vogel, E.F. 881 (glabra var. glabra); 884 (densiflora); 2748 (viminalis); 4181 (annulata); 5517 (celebica var. celebica).

De Vogel, E.F. & Vermeulen, J.J. 7177 (celebica var. celebica); 7312 (inappendiculata).

De Voogd, C.N.A. 555 (gracilis); 556 (fallax); 557 (gracilis); 581 (fallax); 591 (gracilis).

De Vriese, W.H. 41 (inappendiculata); 43 (fallax); 49 (annulata); 50 (fallax); 58 (vacillans); 66 (gracilis); 67 (bracteata); 69 (gracilis); 77 (vacillans); 92, 93 (divaricata); 95 (vacillans); 100 (inappendiculata); 130 (fallax); 132 (vacillans); 149 (leprosa); 154 (inappendiculata); 169 (gracilis); 171 (vacillans); 173, 174 (gracilis).

De Wilde, W.J.J.O. & De Wilde-Duyfjes, B.E.E. 12157, 12568, 12651 (divaricata); 13528 (bakhuizenii); 14660, 15542 (viminalis); 15596 (bakhuizenii); 16545, 18213 (divaricata); 18739 (gracilis); 19233 (divaricata); 19788 (gracilis); 20642, 20738 (viminalis); 21214 (gracilis); 21355 (biligulata).

Derry, R. 59 (divaricata).

Diepenhorst, H. HB1323 (fallax).

Ding Hou 839 (bracteata).

Docters van Leeuwen, W.M. 479, 1264 (gracilis); 2301 (leprosa); 2301a (intermedia); 7904 (fallax); 11423 (intermedia); 11487 (leprosa); 11487a (vacillans); 14130 (gracilis).

Dransfield, J. 4414 (pulchra); 7392 (beccariana).

Du 3009 (barbata).

Duaneh, J. 85 (biligulata); 88 (divaricata).

Edwards, P.J. 2110 (densiflora); 2111 (glabra var. glabra).

Elmer, A.D.E. 8236 (acmura); 10577 (celebica var. celebica); 13352 (divaricata); 15257, 16844 (acmura); 20106 (axillaris); 20259 (glandulosa); 20333 (divaricata); 20794 (glabra var. glabra); 21187 (divaricata); 21291 (stipularis); 21305 (viminalis); 21426 (porphyrocarpa); 21427 (gracilis); 21649 (viminalis); 21840 (biligulata).

Endert, F.H. 1593 (biligulata); 2270 (divaricata); 2275 (glabra var. glabra); 2304 (densiflora); 2425 (pulchra); 2552 (beccariana); 2559 (viminalis); 2933 (pulchra); 3015 (gracilis); 3054 (divaricata); 3060 (pubescens); 3126 (glandulosa); 3127 (laevis); 3201 (viminalis); 3294 (gracilis); 3351 (viminalis); 3358 (gracilis); 3360 (hirsutoidea); 3572 (beccariana); 4660, 4754 (porphyrocarpa); 4767 (beccariana); 5174 (gracilis).

Enggoh 7270 (axillaris); 7246 (glabra var. glabra).

Enoh 181 (vacillans).

Erlo 32 (floccosa).

Eyma, P.J. 1167, 1656 (celebica var. celebica); 2692, 2770 (annulata); 2771 (angiensis); 2788 (divaricata); 4168 (celebica var. celebica).

Forbes, H.O. 459 (angiensis); 460 (fallax); 1047 (intermedia); 2882a (fallax); 3287 (annulata).

Forest Research Institute Malaysia Number (FRI) 242 (inappendiculata); 1827 (divaricata); 5423 (stipularis); 5739 (divaricata); 7132 (gracilis); 7154, 7247 (viminalis); 11163 (stipularis); 11222 (divaricata); 12371 (annulata); 12509 (stipularis); 12786, 12794 (punctulata); 13138, 13278 (pallida); 13499 (gracilis); 13637, 14154 (divaricata); 17660 (punctulata); 17707 (stipularis); 17837 (annulata); 18475, 29182 (divaricata); 34336 (punctulata); 36361 (johorensis); 37342 (pallida); 44615 (annulata); 44735 (gracilis); 51500 (biligulata); 51741 (gracilis); 52047 (bakhuizenii); 52081 (spectabilis); 53289 (gracilis); 54870, 58596, 59167 (divaricata); 59338 (stipularis).

Forestry Bureau Number (FB) 17872 (axillaris).

Forman, L.L. & Blewett, J.B.J. 1034 (porphyrocarpa).

Forsten E.A. 305 (celebica var. celebica).

Franck, C.W. 1135 (divaricata).

Fuchs, H.P. 21150 (biligulata); 21352 (glandulosa).

Gardette, E.C. 1850, 1850B (punctulata); 1886 (divaricata); 1989 (griffithii); 2010 (divaricata); 2046 (pallida); 2279 (stipularis); 2280 (gracilis); 2574 (pubescens).

Gaudichaud-Beaupré, C. 80 (biligulata); 93, 94 (pallida); 95 (divaricata); 96 (pallida); 97 (bracteata).

Geesink, R. 5098 (barbata); 5336, 6262, 6394 (divaricata); 8926 (glabra var. glabra); 9295 (biligulata).

Gibbs, L.S. 3951 (macrosepala); 3977 (rubiginosa).

Giesen, W. 69 (divaricata).

Girmansyah, D. 1187 (celebica var. celebica); 1847 (punctulata).

Göring, P.F.W. 331 (leprosa); 6093 (inappendiculata).

Griffith, W. 2268 (annulata); 2269 (griffithii); 2288 (divaricata); 2291 (punctulata); 2292 (pallida).

Gushilman I. et al. 382 (annulata).

Haegens, R.M.A.P. 346 (biligulata); 360 (annulata).

Hallier, J.G. 334 (gracilis); 432, 626 (intermedia); 626a (leprosa); 2013 (beccariana).

Haniff, M. 349 (pallida).

Hansen, C. 244 (pubescens); 645 (hirsutoidea); 670 (pulchra); 705 (porphyrocarpa); 707 (divaricata); 878, 954 (hirsutoidea); 999 (annulata); 1158 (viminalis); 1212 (divaricata); 1213, 1226 (biligulata); 1366 (rostrata); 1579 (porphyrocarpa); 1593 (pulchra).

Hardial Singh & Samsuri 181 (pallida); 237 (biligulata); 278 (inappendiculata); 295 (pallida); 306 (gracilis).

Hardial Singh & Sidek 396 (divaricata).

Hartley, T.G. 9702 (annulata); 11395 (brassii).

Hassan & Kadim H.10 (divaricata).

Haviland, G.D. 68 (biligulata); 69 (sarawakensis); 78 (annulata); 144, 144E (pubescens); 151 (bracteata); 159 (viminalis); 545 (divaricata); 546 (annulata); 549 (gracilis); 862 (hirsutoidea); 863 (divaricata); 971 (viminalis); 1287, 1288 (porphyrocarpa); 1385 (axillaris); 1508 (glandulosa); 1550 (densiflora); 1901 (biligulata); 2036 (rostrata); 2207 (gracilis); 3143 (divaricata); 3144 (pallida); 3387 (gracilis); 3628 (beccariana).

Hayata, B. 337 (barbata).

Helfer, J.W. 2286 (biligulata); 2290 (barbata).

Hellendoorn, E.R. 3 (inappendiculata); 4 (gracilis); 5 (vacillans); 8 (sagittata); 9 (fallax).

Hidayat, A. 4205 (annulata).

Hidink 4765 (divaricata).

Hochreutiner, B.P.G. 159 (gracilis).

Hollrung, U.M. 656 (schumannii).

Hoogland, R.D. & Craven, L.A. 10180, 10514 (schumannii).

Hoover, W.S. 36 (fallax); 473 (schumannii); 820 (inappendiculata); 5502 (intermedia); 20323 (inappendiculata); 30486 (gracilis); 30504, 30621 (inappendiculata); 30691 (gracilis); 31877 (inappendiculata); 31909 (bakhuizenii); 31994 (inappendiculata); 32172, 32564 (vacillans); 32663 (leprosa); 32767 (inappendiculata).

Horsfield, T. 11 (inappendiculata); 12 (gracilis); 15 (pallida); 19 (biligulata); 438 (divaricata).

Horst, W.A. 2 (leprosa).

Hose, C. 181 (divaricata); 478 (pulchra).

Hotta, M. 12 (fallax); 56 (gracilis); 429, 1637 (fallax); 6113 (pulchra).

Hue, R. 462 (biligulata).

Hullet, R.W. 179 (pallida); 505 (punctulata).

Iboet 12 (annulata); 46 (bakhuizenii); 276 (viminalis); 423 (divaricata); 432 (bakhuizenii).

Irawati & Sadili, A. 201 (bakhuizenii).

Ismael 83 (viminalis).

Ismail & Arifin, Z. BRF1714 (pulchra).

Isman, A. 5 (bakhuizenii).

Iwatsuki, K. S-401 (biligulata); S-405 (inappendiculata).

Izu 150 (viminalis).

Jack, W. 49 (divaricata); 55 (pallida).

Jacobs, M. 4517 (glandiformis); 4550 (inappendiculata); 4596 (viminalis); 5361 (glandulosa); 8213 (fallax); 9676 (annulata).

Jacobson, E. 2412 (bracteata); 2426 (biligulata); 2679 (gracilis).

Jaheri 330 (pulchra); 704 (biligulata); 1104 (pulchra); 1297, 1631 (gracilis).

Janowsky, R.F. 132 (papuana).

Johns, R.J. 7286 (pulchra); 7389 (porphyrocarpa); 8887 (schumannii).

Junghuhn, F.W. 13, 196, 198 (leprosa); 201 (inappendiculata).

Kadim & Noor, M. 357 (punctulata).

Kakah 92 (leprosa).

Kanehira, R. & Hatusima, S. 11999 (annulata); 13374 (angiensis).

Karsten, B.J. 66 (leprosa).

Kartawinata, K. 996 (densiflora); 1185 (rostrata).

Kartonegoro, A. 314 (gracilis); 318 (vacillans); 737 (fallax); 779 (annulata); 1056 (bracteata); 1058 (pallida); 1069 (divaricata); 1073 (horrida); 1074 (bracteata); 1075 (viminalis); 1078 (conica); 1079 (viminalis); 1088 (biligulata); 1095 (viminalis); 1096 (divaricata); 1100 (spectabilis); 1101 (conica); 1103 (bakhuizenii); 1104 (inappendiculata); 1105 (vacillans); 1106 (fallax); 1107 (vacillans); 1110 (fallax); 1113 (gracilis); 1114 (bakhuizenii); 1115 (inappendiculata); 1116 (bakhuizenii); 1117 (vacillans).

Kato, M. B-5202 (pulchra); B-6995 (axillaris); B-7131 (pulchra); B-7957 (viminalis); B-9017 (annulata); B-9030 (viminalis); B-10052 (pulchra); B-10105 (micrantha).

Kaudern, W. 6 (divaricata).

Keng, H. 20 (inappendiculata); 122 (viminalis).

Kepong Serial Number (KEP) 55818 (divaricata); 95044 (stipularis); 97965 (johorensis); 97980 (biligulata); 98073 (annulata); 98504 (divaricata); 98980 (gracilis); 99079 (divaricata); 99109 (pallida); 104410 (gracilis); 104685 (biligulata); 104968 (gracilis).

Kerr, A.F.G. 5870 (barbata); 7215 (divaricata); 7433, 7433A, 7812 (gracilis); 10075 (divaricata); 13232 (barbata); 14461 (viminalis); 14470 (gracilis); 14587 (viminalis); 15310 (gracilis); 15580, 16529 (divaricata); 16573, 17113 (barbata); 17904, 19087 (divaricata); 19192 (bracteata); 19233 (gracilis).

Kessler, P.J.A. 913 (densiflora); 919 (gracilis); 1461 (rostrata); 2032 (divaricata); 2070 (biligulata); 2111 (viminalis); 2240, 2657 (biligulata); B-1585, Berau-25 (divaricata); Berau-128A (pulchra).

King’s Collector 224 (annulata); 369 (divaricata); 655 (viminalis); 657 (gracilis); 2258 (viminalis); 2468 (gracilis); 2570 (pallida); 2911 (biligulata); 3965 (pallida); 5284 (bakhuizenii); 8499 (pallida); 10290 (biligulata); 10468, 10864 (viminalis).

Kirkup, D.W. et al. 430 (biligulata); 578 (pubescens).

Kjellberg, G.K. 599 (annulata); 1820a (celebica var. celebica).

Klackenberg, J. & Lundin, R. 673 (bakhuizenii).

Klein 8218 (divaricata).

Kleinhoonte, A. 584 (fallax).

Koizumi, M. 317 (pulchra).

Kokawa, S. & Hotta, M. 892 (stipularis); 2089 (rubiginosa); 2603 (pulchra); 2696 (biligulata); 5190 (rubiginosa); 5222 (gracilis); 5798 (micrantha).

Koorders, S.H. 14934β (gracilis); 14991β (inappendiculata); 15149β (gracilis); 22330β (pallida); 24270β (fallax); 25927β (intermedia); 25949β (leprosa); 26306β (vacillans); 27106β (gracilis); 27844β, 27846β. 29308β (leprosa); 30690β (gracilis); 31506β, 31523β (leprosa); 31670β (vacillans); 32905β, 33313β (inappendiculata); 33314β, 33315β (vacillans); 33358β (fallax); 33833β, 34045β, 34611β (gracilis); 34613β (fallax); 35839β (gracilis); 35840β, 35841β (leprosa); 40659β, 40727β (vacillans); 40738β (fallax); 40911β, 40927β, 41065β, 41417β (gracilis); 42153β (intermedia); 47851β (vacillans).

Kornassi 1403 (angiensis).

Kostermans, A.J.G.H. 632 (annulata); 1322 (angiensis); 4386 (annulata); 4960 (biligulata); 4961 (divaricata); 5085 (biligulata); 5452 (axillaris); 5845 (gracilis); 6846 (axillaris); 6965 (divaricata); 7595 (annulata); 8045 (rostrata); 13690 (pulchra); 19164 (vacillans); 21364, 21644 (divaricata); 23846 (gracilis).

Kuhl, H. & Van Hasselt, J.C. 12, 48 (gracilis).

Kunstler, H.H. 170 (biligulata); 197 (johorensis).

Kurz, S. 1011 (gracilis).

Lae Forestry Number (LAE) 51727 (divaricata); 57470 (angiensis); 77282 (annulata).

Lakshnakara, H.S.H. 679 (divaricata); 739 (barbata).

Lam, H.J. 65, 141 (inappendiculata); 230 (intermedia); 935 (schumannii); 2205 (inappendiculata).

Laman, T.G. et al. 110 (biligulata); 708 (viminalis); 1018 (divaricata); 1357 (annulata); 1396 (viminalis).

Langlasse, E. 83 (gracilis); 187 (biligulata).

Lanjouw, L.R. 179 (gracilis).

Larsen, K. & Larsen, S.S. 32731 (gracilis); 32923 (biligulata); 33279 (gracilis); 33470 (barbata).

Larsen, K. et al. 42845 (gracilis); 42921 (divaricata); 42922, 45732 (gracilis).

Latiff, A. 859 (inappendiculata).

Ledermann, C.L. 6654 (divaricata).

Leeuwenberg, A.J.M. & Rudjiman 13087 (gracilis); 13432 (stipularis); 14505 (divaricata); 14534 (gracilis).

Leg. Ign. FS48 (leprosa).

Leighton, R. 418 (gracilis); 433 (rostrata).

Leong, P. 459 (divaricata).

Lörzing, J.A. 1493 (intermedia); 4332 (fallax); 4349 (bakhuizenii); 4607 (fallax); 5198, 5724 (divaricata); 7351 (inappendiculata); 7351a (fallax); 11439 (bracteata); 12317, 12318 (divaricata); 13673 (bakhuizenii); 14075 (fallax); 17296 (stipularis).

Lucas, E.J. 24 (schumannii).

Lugas, L. 232, 258 (divaricata); 1121 (gracilis); 1846 (biligulata); 2303 (divaricata).

Machado, A.D. 11580 (gracilis).

Mahyar, U.W. et al. 1229 (rostrata); 1373 (annulata).

Maingay, A.C. 784 (griffithii); 788 (annulata); 789 (punctulata); 790 (biligulata); 791 (bracteata); 792 (pallida); 793 (divaricata); 793[2227], 793[2685] (pallida); 794 (divaricata); 795 (viminalis).

Maradjo, S. 102 (biligulata); 350 (floccosa).

Martin 348 (biligulata).

Maskuri 282 (fallax).

Maxwell, J.F. 01-274, 11-25 (divaricata); 77-88 (punctulata); 77-181 (johorensis); 77-359 (annulata); 78-23 (punctulata); 78-24 (biligulata); 78-31 (johorensis); 78-76 (divaricata); 78-81 (fallax); 78-81a (annulata); 78-83 (bakhuizenii); 78-115 (divaricata); 78-133 (spectabilis); 78-139 (inappendiculata); 78-197 (bakhuizenii); 78-207 (annulata); 78-211 (gracilis); 78-296 (divaricata); 78-297 (gracilis); 78-307 (rectandra); 78-308 (bracteata); 78-310 (bakhuizenii); 78-312 (rectandra); 78-323 (spectabilis); 78-324 (biligulata); 78-336 (punctulata); 78-366 (annulata); 78-367 (inappendiculata); 78-368 (bakhuizenii); 78-370 (divaricata); 79-48 (johorensis); 79-50 (gracilis); 80-210 (viminalis); 81-9 (gracilis); 81-15, 81-91 (punctulata); 81-158 (viminalis); 81-179 (gracilis); 81-222 (viminalis); 82-3, 82-194 (biligulata); 86-45 (stipularis); 86-552 (biligulata); 86-905 (divaricata); 87-254 (gracilis); 99-179 (divaricata).

McDonald, J.A. & Ismail 3600 (pulchra).

Meijer, W. 105 (vacillans); 1363 (intermedia); 1866, 1908, 2575 (biligulata); 3007 (bakhuizenii); 4271 (horrida); 4289 (biligulata); 4329 (viminalis); 5002 (biligulata); 5253 (viminalis); 7024, 7026a (pallida); 7138 (viminalis); 7180 (bracteata); 7245 (fallax); 7282 (glandiformis).

Mogea, J.P. & De Wilde, W.J.J.O. 3669 (gracilis); 3716 (rostrata); 4339 (densiflora); 4376 (rostrata).

Mondi 252 (pallida).

Mori et al. 17853 (biligulata).

Moulton, J.C. 6711 (divaricata).

Moulon’s Native Collector 40 (viminalis).

Murata, g. et al. B-459 (rostrata); B-586 (divaricata); B-587, B-600 (pulchra); B-3041 (biligulata); J-875 (gracilis).

Nagamasu, H. 3325 (fallax); 3773 (biligulata); 3782 (bakhuizenii).

Nangkat, N. 89 (latifolia); 251 (rostrata); 265 (biligulata).

Native Collector 85, 96 (viminalis); 388 (bracteata); 580, 1798 (viminalis); 2477 (annulata); 2819 (pulchra).

Nedi 28 (angiensis).

New Guinea Forestry Number (NGF) 3751 (schumannii); 5815 (angiensis); 14452 (annulata); 14886, 21952 (brassii); 22928 (angiensis); 23241 (annulata); 23243 (angiensis); 25730 (annulata); 26113 (angiensis); 31814, 31912 (viminalis); 33015, 46798 (schumannii).

Nimanong, B. 89 (barbata).

Niyomdham, C. 868, 6228 (divaricata).

Noor, M. & Samsuri 66 (divaricata).

Nooteboom, H.P. 1138, 1198 (pulchra); 1664 (viminalis); 4370 (rostrata).

Nurainas 71 (stipularis).

Nurta et al. 154 (inappendiculata).

Ogata, K. 10040a (divaricata); 10513 (annulata); 11037 (divaricata); 11671 (biligulata); Og-B94 (stipularis); Og-B320 (biligulata).

Olsen, S.-E.S. 989 (axillaris).

Orolfo, P. 8 (glabra var. glabra); 22 (bracteata).

Parkinson, C.E. 1691, 1953 (barbata).

Pawanche & Awang Lela 13652 (stipularis).

Pearce, K.G. et al. ITTO/BB 431 (rostrata).

Pereira, J.T. 111 (glabra var. kinabaluensis); 246 (pulchra).

Philippine National Herbarium Number (PNH) 964, 1008 (divaricata); 1042 (celebica var. celebica); 13453, 14463, 14514, 21567 (acmura); 38277 (celebica var. celebica); 41847 (acmura); 41871, 42216 (divaricata); 42217, 98404 (acmura); 108992 (celebica var. celebica); 119985 (acmura).

Philippine Plant Inventory Number (PPI) 368 (biligulata); 1317, 2037, 2212, 2425 (acmura); 2635 (celebica var. celebica); 4378 (divaricata); 4855 (acmura); 5245 (celebica var. celebica); 7448, 8440, 9293, 13023, 18314, 18494, 21956 (acmura); 24289, 26128 (divaricata); 37255, 37459 (acmura).

Pierre, L. 3032 (inappendiculata).

Pleyte, D.R. 299 (angiensis); 705 (papuana).

Podzorski, A.C. SMHI-906 (divaricata); SMHI-944 (stipularis).

Poilane, E. 10854, 12234, 22026, 22291, 27539, 31700, 31855, 35651 (barbata).

Pooma, R. 4520 (viminalis); 5136 (divaricata); 5259 (barbata).

Poore, M.E.D. 143 (divaricata); 144 (biligulata); 535 (divaricata).

Posthumus, O. 687, 837 (biligulata); 937 (divaricata); 987 (viminalis).

Prance, G.T. & Dransfield, J. 30592, 30623 (biligulata).

Prawiroatmodjo, S. 1984 (celebica var. celebica); 2386 (biligulata).

Project Barito Ulu Number (PBU) 81 (atrobrunnea); 185 (pubescens); 187, 226 (gracilis); 229 (atrobrunnea); 456 (divaricata); 647 (latifolia); 692 (biligulata).

Promchua, M. 48 (gracilis).

Puasa Angian 1404 (divaricata).

Pule, A.A. 4073 (intermedia).

Pullen, R. 8424 (schumannii).

Purseglove, J.W. 4112 (rectandra); 4114 (divaricata); 4353 (bracteata); 5401 (densiflora).

Put, P. 778, 3635 (biligulata).

Putri et al. 63 (pallida).

Raap, H. 158 (intermedia); 667 (leprosa); 695A (sagittata).

Rabil 268 (divaricata).

Rachmat 397 (celebica var. celebica).

Raes, N. 720 (viminalis).

Rahayu, M. & Maskuri 473 (biligulata).

Rahmat Si Boeea 7781 (conica); 9041 (viminalis); 9075 (bracteata); 9241 (biligulata); 9464 (gracilis); 9964 (biligulata).

Rahmat Si Toroes 3294, 3837 (pallida); 4634 (biligulata); 5326 (gracilis).

Ramlanto 461 (annulata).

Ramos, M. 437 (acmura); 1207 (annulata); 1292 (divaricata); 1585 (pubescens); 1758 (axillaris).

Rant, A. 881 (annulata).

Regalado, J.C. & Takeuchi, W.N. 1426 (brassii).

Richards, P.W. 1301 (pubescens); 1401 (stipularis); 1818 (biligulata).

Ridley, H.N. 349 (biligulata); 548 (punctulata); 1574 (stipularis); 2012 (divaricata); 2014 (viminalis); 2015 (pallida); 2016 (punctulata); 2017, 2017a (pallida); 2025 (biligulata); 3858, 3918 (punctulata); 4185 (johorensis); 5087 (annulata); 7332 (gracilis); 8464 (viminalis); 11103 (divaricata); 11105, 11444 (viminalis); 11595 (divaricata); 12259 (pubescens); 13686 (inappendiculata); 14639 (gracilis).

Ridsdale, C.E. 1935 (viminalis); 1961 (glabra var. glabra); 1981 (divaricata); 2017 (gracilis); 2327 (schumannii).

Riswan, S. ML051 (stipularis).

Robinson, C.B. 2024 (annulata).

Royal Society North Borneo Number (RSNB) 68 (biligulata); 69 (annulata); 528 (stipularis); 2647 (glabra var. kinabaluensis).

Rumutom, M. 305 (divaricata); 306 (annulata); 473 (pulchra).

Rus, Y. 408183 (bakhuizenii).

Rutten, L.M.R. 103 (pulchra); 170 (biligulata); 535 (rostrata); 547 (gracilis); 580 (biligulata); 1904 (divaricata).

Sambuling, S. 244, 262, 468 (biligulata); 596 (gracilis).

Samsuri 352 (gracilis).

Sandakan Forestry Series Number (SAN) 19243 (stipularis); 19525 (biligulata); 24253 (viminalis); 26097 (divaricata); 27732 (annulata); 28030 (biligulata); 28134 (stipularis); 28959 (micrantha); 30555 (beccariana); 31570 (axillaris); 34332 (hirsutoidea); 34540 (beccariana); 35395, 36928 (glabra var. glabra); 38278 (divaricata); 40929 (gracilis); 41194, 41260, 41606 (divaricata); 41905 (stipularis); 46742 (glabra var. kinabaluensis); 50522 (stipularis); 54413 (densiflora); 54477 (stipularis); 56381 (rubiginosa); 57228 (densiflora); 57379 (biligulata); 58046 (glabra var. glabra); 60183 (divaricata); 60895 (annulata); 64762 (divaricata); 66574 (glabra var. glabra); 66948 (bracteata); 66950, 67100 (biligulata); 67567 (pubescens); 67969 (viminalis); 69037 (gracilis); 69084 (viminalis); 69335, 70032 (annulata); 70925 (porphyrocarpa); 71095 (divaricata); 71173 (axillaris); 72083 (viminalis); 72234 (pulchra); 73375 (biligulata); 74187 (hirsutoidea); 74345 (viminalis); 74415 (divaricata); 74565 (pubescens); 75319 (divaricata); 75633 (glabra var. glabra); 75687 (pulchra); 77090, 77566 (divaricata); 77594 (pulchra); 77821 (biligulata); 77955 (porphyrocarpa); 77963 (gracilis); 78319 (pulchra); 78421 (bracteata); 78447 (viminalis); 79188 (divaricata); 79751 (viminalis); 79761 (annulata); 80819 (divaricata); 81101 (pulchra); 81113 (divaricata); 81490 (hirsutoidea); 81978 (axillaris); 82620 (biligulata); 83025 (viminalis); 83210 (glabra var. glabra); 83349 (annulata); 83571 (axillaris); 83878 (stipularis); 83879 (annulata); 84312, 84799 (divaricata); 85351 (stipularis); 85753 (biligulata); 85957 (porphyrocarpa); 87884 (hirsutoidea); 88408 (biligulata); 89206 (pulchra); 89899, 90255 (biligulata); 90697, 91256 (densiflora); 91275 (annulata); 91283 (hirsutoidea); 91826 (stipularis); 92962 (biligulata); 93072 (divaricata); 94467 (pulchra); 94559 (glabra var. glabra); 94758, 94790 (divaricata); 94808 (viminalis); 94866 (annulata); 95456 (biligulata); 95585 (porphyrocarpa); 95687, 96488 (divaricata); 97451, 101240 (biligulata); 102705 (annulata); 103629 (stipularis); 103669 (glabra var. glabra); 103670, 106138, 107148 (divaricata); 110070 (gracilis); 110178 (stipularis); 110269 (pulchra); 110362 (beccariana); 110449 (porphyrocarpa); 112059 (glabra var. glabra); 113060 (stipularis); 113224 (gracilis); 113279, 114076 (porphyrocarpa); 114333 (bracteata); 114565 (axillaris); 114572 (gracilis); 114944 (viminalis); 115845 (axillaris); 115864 (pulchra); 116163 (divaricata); 116348 (biligulata); 116362, 116412 (divaricata); 116427 (annulata); 116711 (divaricata); 116983 (pubescens); 116989, 117976 (divaricata); 118472 (biligulata); 118688, 119836, 119938 (divaricata); 120183 (viminalis); 120365 (glabra var. glabra); 121169, 121484, 121990, 123600 (divaricata); 123756 (pulchra); 124418 (glabra var. glabra); 124683 (stipularis); 125310 (biligulata); 125639 (gracilis); 125645 (biligulata); 125669 (divaricata); 127929 (biligulata); 127960 (gracilis); 127973 (viminalis); 128066 (pubescens); 128076, 128120 (biligulata); 128382 (porphyrocarpa); 128385 (glandulosa); 128874 (densiflora); 129145 (annulata); 129794 (gracilis); 129999 (divaricata); 130786 (viminalis); 131046 (axillaris); 132639, 132681, 133876 (pulchra); 133936 (viminalis); 134068, 134269 (divaricata); 135689 (annulata); 135746 (divaricata); 136035 (glandulosa); 136931 (biligulata); 136959 (viminalis); 139507 (pulchra); 143515 (divaricata); 143924 (pulchra); 143959 (porphyrocarpa); 143985 (divaricata); 144145 (pulchra); 145986 (biligulata); 145988 (porphyrocarpa); 146062 (glandulosa); 146067 (divaricata); 146068 (porphyrocarpa); 146684 (pubescens); 146905 (bracteata); 147687 (glabra var. glabra); 147913 (divaricata); 148885 (biligulata); 149046 (viminalis); 151210 (divaricata); 152101 (bracteata).

Sands, M.J.S. & Johns, R.J. 5445 (porphyrocarpa).

Sangkhachand, B. & Phusomsaeng, S. 1539 (gracilis).

Santos, J.K. 4267 (divaricata).

Sapiin 57 (intermedia); 1115 (leprosa).

Sarawak Forestry Series Number (S.) 3004 (biligulata); 4071 (pubescens); 4367, 5922 (latifolia); 8460 (biligulata); 8462 (glandulosa); 11981 (rostrata); 13494 (bracteata); 13984 (divaricata); 16326 (beccariana); 17254 (biligulata); 17256 (latifolia); 17786 (hirsutoidea); 19481 (sarawakensis); 19751 (viminalis); 19868 (hirsutoidea); 19873 (porphyrocarpa); 19946 (pubescens); 21105 (pulchra); 21200 (pubescens); 21304 (divaricata); 21636 (annulata); 21720 (densiflora); 21910 (gracilis); 22896 (viminalis); 23062 (annulata); 23280 (pulchra); 23617 (annulata); 23863 (hirsutoidea); 23880 (divaricata); 24301 (viminalis); 24556 (annulata); 25075 (biligulata); 25084 (rostrata); 26207 (beccariana); 27040 (sarawakensis); 27216 (pubescens); 27219 (glandulosa); 28175 (pubescens); 31103 (beccariana); 31862 (rostrata); 33173 (glandulosa); 33794 (viminalis); 33819 (porphyrocarpa); 33998, 34082 (densiflora); 34270 (hirsutoidea); 34495 (bracteata); 34713 (sarawakensis); 34771 (porphyrocarpa); 35025 (pubescens); 35320 (annulata); 35335 (biligulata); 35384 (viminalis); 36793 (biligulata); 36955 (pubescens); 37213 (viminalis); 37410 (porphyrocarpa); 37642 (latifolia); 39326 (laevis); 39492 (glandulosa); 40000 (beccariana); 40124 (rostrata); 40214 (densiflora); 40230 (pubescens); 40264 (rostrata); 40332 (biligulata); 40438 (porphyrocarpa); 41368 (beccariana); 41775 (pubescens); 41990 (glandulosa); 42599 (sarawakensis); 42845 (beccariana); 42897 (pubescens); 45085 (biligulata); 45092 (pubescens); 45094 (annulata); 45415 (pulchra); 45446, 45555, 45974 (viminalis); 46305 (biligulata); 47543 (viminalis); 47645 (biligulata); 48017 (annulata); 48265 (sarawakensis); 48814 (biligulata); 48867 (gracilis); 48949 (pubescens); 50457 (viminalis); 50537 (beccariana); 51005 (viminalis); 52539 (annulata); 52727 (gracilis); 53018 (bracteata); 53694 (divaricata); 53947 (glabra var. glabra); 53996 (porphyrocarpa); 56049 (beccariana); 56359 (divaricata); 56571 (pulchra); 56745, 56746, 56899 (pubescens); 57198 (biligulata); 58220 (densiflora); 58277 (beccariana); 59909 (viminalis); 61328 (annulata); 61445 (porphyrocarpa); 62014 (laevis); 62032 (porphyrocarpa); 63011 (gracilis); 63137 (maxwellii); 63183 (pulchra); 63213 (glandulosa); 64404 (pubescens); 64697, 64698 (gracilis); 65744 (biligulata); 65799 (porphyrocarpa); 66939 (annulata); 68539, 68540 (biligulata); 68562 (viminalis); 69527 (pubescens); 71615, 71651 (biligulata); 72466 (pulchra); 73359 (pallida); 73711, 73791 (annulata); 74133 (biligulata); 74524 (latifolia); 79258 (viminalis); 80966 (annulata); 81367 (viminalis); 82832 (gracilis); 84494 (pulchra); 91083 (hirsutoidea); 91085 (biligulata); 92155 (annulata); 95559 (biligulata); 97859 (pulchra); 97994 (biligulata); 98016 (porphyrocarpa); 98377 (biligulata).

Sarip 219 (fallax); 465 (leprosa).

Schiffner, V.F. 2291 (leprosa); 2293 (inappendiculata).

Schlechter, R.F.F. 15159 (fallax).

Schodde, R. 2189 (brassii).

Scortechini, B. 22 (pallida); 23 (biligulata); 28 (divaricata); 127 (gracilis); 226 (biligulata); 235 (annulata); 371 (pallida); 1469 (biligulata); 1650 (pallida); 2106 (stipularis).

Seeman, B.C. 2369 (biligulata).

Seren 65 (spectabilis).

Setiabudi 143 (fallax).

Shah, M. 621 (bakhuizenii); 129 (biligulata); 133 (divaricata); 176 (stipularis); 431 (punctulata); 680A (biligulata); 1163 (viminalis); 1308 (gracilis); 1162 (inappendiculata); 2265 (gracilis); 4944 (annulata); 4954 (biligulata).

Shea G. 23289, 26643 (annulata); 26846 (laevis); 27033 (annulata); 27039 (viminalis); 27146 (pubescens).

Shimizu, T. et al. M-13467 (bakhuizenii); 13927 (annulata).

Sidek bin Kiah 229 (divaricata); 276 (pallida); 288 (divaricata).

Sidin 214 (biligulata).

Sidiyasa, K. 606 (gracilis); 719, 2010 (pulchra); 2536 (viminalis); 3349 (gracilis).

Siew, W.H. 95, 201 (spectabilis).

Simaga, S. 1836 (schumannii).

Simpson, D.A. & Marsh, M. 2115 (glandulosa); 2502 (biligulata); 2503 (gracilis); 2600 (stipularis).

Sinclair, J. 9235 (micrantha).

Singapore Field Number (SFN) 2297 (divaricata); 2302 (viminalis); 2774 (divaricata); 4081, 6841 (pallida); 6959 (gracilis); 7799, 7849 (punctulata); 8077 (pallida); 8628, 10657 (annulata); 11529 (pallida); 11708 (biligulata); 12120 (gracilis); 12282 (bakhuizenii); 13082 (divaricata); 14661 (gracilis); 15654 (biligulata); 15726 (gracilis); 19156 (hirsutoidea); 19363 (biligulata); 19975 (johorensis); 21404 (divaricata); 23973 (gracilis); 24057 (annulata); 25848 (biligulata); 28776 (gracilis); 29377 (punctulata); 29646 (stipularis); 30081 (biligulata); 30207 (divaricata); 30223 (gracilis); 30381 (malayana); 30666 (viminalis); 31645 (divaricata); 31912 (biligulata); 32357 (johorensis); 32600 (divaricata); 33853 (stipularis); 33858 (malayana); 34345 (gracilis); 34429, 34586, 35137 (divaricata); 38484 (annulata); 38817, 38984 (divaricata); 40491 (stipularis); 40492 (malayana); 40649 (punctulata).

Smith, J.J. 125, 719 (leprosa); 822 (vacillans).

Soedarsono 244 (viminalis); 432 (inappendiculata).

Soegandiredja 82 (gracilis).

Soegeng Reksodihardjo, W. 6 (intermedia); 295 (viminalis); 481 (schumannii).

Soejarto, D.D. 6344 (divaricata); 7458 (biligulata); 13310 (barbata).

Soepadmo, E. 653 (viminalis).

Sorensen, T. et al. 7175 (divaricata).

Stone, B.C. 13535, 14131 (spectabilis); 15244 (pallida).

Strugnell, E.J. 13618 (stipularis); 13965 (griffithii).

Sugau, J.B. 165B (pulchra).

Sulistyaningsih, L.D. 5 (inappendiculata).

Sun, H.F. 226 (biligulata); 475 (gracilis); 9778 (pallida).

Susanti, R. et al. 149 (biligulata); 264 (rostrata); 276 (annulata).

Suzuki, E. K-13181 (stipularis).

Tadong 190 (divaricata).

Tagawa, K. et al. 77, 587 (pulchra); 798 (biligulata).

Takeuchi, W. 5668 (brassii); 6192 (annulata); 7040, 7040A (brassii); 10198, 10790, 11200 (angiensis); 11438 (brassii); 16004 (angiensis); 18228, 18685 (biligulata); 18771 (divaricata); 18792 (biligulata); 18794 (conica).

Tamin, R. 591 (viminalis).

Teijsmann, J.E. 1860 (fallax); 3197, 3424 (pallida); 3634 (divaricata).

Teo, Y. & Purseglove, J.W. 1 (gracilis); 23 (viminalis); 165 (biligulata).

Tjoa, A. A14 (annulata).

Tokuoka, T. et al. T-322 (fallax).

Tuke P1 1000, P5 1010 (rostrata).

Treub, M. 564 (angiensis).

Uce 106 (horrida).

Uchida, H. 20 (leprosa); 90 (fallax).

Ueda, K. & Darnaedi, D. B-8515 (divaricata).

Ultée, A.J. s.n. (fallax).

Umbol 6201 (divaricata).

University of Papua New Guinea Number (UPNG) 4849 (annulata); 20172 (schumannii).

University of San Carlos 819 (acmura).

Utteridge, T.M.A. 312 (schumannii).

Van Balgooy, M.M.J. 3205 (celebica var. longilobata); 5161 (bakhuizenii); 5179 (gracilis); 5840 (pubescens); 7114 (johorensis); 7219 (divaricata).

Van Balgooy, M.M.J. & Wiriadinata, H. 2889 (intermedia); 2921 (gracilis); 2922 (vacillans).

Van Balgooy, M.M.J. & Mogea, J.P. 4284 (inappendiculata).

Van Beusekom, C.F. & Phengkhlai, C. 880 (fallax).

Van Borssum-Waalkes, J. 1532 (fallax); 1533 (bakhuizenii); 1871 (gracilis); 1888 (biligulata); 1890 (fallax); 1893 (bakhuizenii); 2659 (gracilis).

Van Heel, W.A. 338 (gracilis).

Van Ooststroom, S.J. 12833 (inappendiculata); 13840 (leprosa).

Van Royen, P. 4081 (annulata); 4216 (brassii).

Van Royen, P. & Sleumer, H.O. 7574, 7646 (angiensis).

Van Slooten, D.F. 282 (divaricata).

Van Steenis, C.G.G.J. 2300 (fallax); 2486 (leprosa); 2712, 3538, 4002 (gracilis); 4023, 5685 (inappendiculata); 9411 (divaricata); 9478 (gracilis); 9972 (fallax); 10074, 11756 (gracilis); 11782 (fallax); 12735 (intermedia); 17373 (fallax); 17412 (conica).

Van Valkenburg, J. 1041 (stipularis); 1252 (annulata); 1255 (divaricata).

Vanpruk, P. 697 (gracilis).

Veldkamp, J.F. 8076 (rostrata); 8077 (densiflora); 8297 (biligulata); 8326 (gracilis).

Vermeulen, J.J. & Duistermaat, H. 2023 (inappendiculata).

Villamil, A. 242 (glabra var. glabra).

Walker, G.W. 23 (viminalis); 28 (pallida); 30 (punctulata); 39 (divaricata).

Wallich, N. 4044 (bracteata); 4049 (pallida); 4050 (biligulata); 4050a (gracilis); 4051 (divaricata); 4052 (biligulata); 4053 (viminalis); 4054, 4055 (divaricata); 4082 (barbata).

Wanariset Number (AA) 283 (divaricata); 468 (biligulata); 763 (viminalis); 1280, 1667 (axillaris); 1719, 1764 (biligulata); 1921 (pulchra); 3115 (densiflora).

Wanariset Number (W) 728 (pubescens); 747 (divaricata).

Wardi BOHK 390 (biligulata).

Wenzel, C.A. 414, 662, 3043 (acmura).

Whitmore, T.C. & Kalima, T. 3245 (viminalis).

Widjaja, E.A. 1616 (annulata); 3220 (vacillans); 6894 (annulata); 6930 (schumannii); 9400 (celebica var. celebica); 9718 (annulata); 9846a (celebica var. celebica).

Wilkie, P. 93360 (gracilis); 93374 (rostrata).

Williams, K. 1148 (barbata).

Williams, R.S. 2571 (celebica var. celebica).

Winckel, W.F. 710, 959 (fallax); 1176 (vacillans).

Winda 19 (fallax).

Winkler, H.J.P. 1752 (divaricata); 2627 (biligulata); 2809 (gracilis); 2970 (divaricata); 3033 (axillaris); 3272 (viminalis).

Winkler, J. 590 (annulata); 1167 (rostrata).

Wirawan, N. 265 (gracilis).

Wiriadinata, H. 691 (pulchra); 1151 (divaricata); 3385 (gracilis); 3600 (biligulata).

Wiriadinata, H. & Hoover, W.S. 31188 (vacillans); 31204 (bakhuizenii).

Wiriadinata, H. & Maskuri 538 (gracilis); 658 (viminalis).

Wisse, C.A. 1188 (intermedia).

Wong, K.M. 347 (viminalis); 1434 (annulata); 1439 (beccariana); 1869 (pubescens).

Wood, D.D. 2389 (divaricata).

Worthington, R.D. 12804 (spectabilis).

Wray, L. 571 (divaricata); 641 (viminalis); 1194 (divaricata); 1370 (pallida); 3610 (divaricata).

Yates, H.S. 1271 (gracilis); 1424 (inappendiculata); 1599 (gracilis); 2012 (nodosa).

Yunnas 34 (gracilis).

Zahro 69, 113 (bakhuizenii).

Zainudin, A. et al. 4535 (rostrata); 5131 (bracteata).

Zeijlstra, H.H. 19 (leprosa).

Zhang, X.C. et al. 613 (intermedia).

Zollinger, H. 769 (gracilis); 1288, 1489 (inappendiculata); 3044 (divaricata); 3511 (gracilis); 3661 (divaricata).

Zul et al. 43 (spectabilis).
